# Tandem sequential catalytic enantioselective synthesis of highly-functionalised tetrahydroindolizine derivatives†‡

**DOI:** 10.1039/d0sc00432d

**Published:** 2020-03-12

**Authors:** Shuyue Zhang, Mark D. Greenhalgh, Alexandra M. Z. Slawin, Andrew D. Smith

**Affiliations:** EaStCHEM, School of Chemistry, University of St Andrews North Haugh St Andrews KY16 9ST UK ads10@st-andrews.ac.uk

## Abstract

An isothiourea-catalysed enantioselective synthesis of novel tetrahydroindolizine derivatives is reported through a one-pot tandem sequential process. The application of 2-(pyrrol-1-yl)acetic acid in combination with either a trifluoromethyl enone or an α-keto-β,γ-unsaturated ester in an enantioselective Michael addition–lactonisation process, followed by *in situ* ring-opening and cyclisation, led to a range of 24 tetrahydroindolizine derivatives containing three stereocentres in up to >95 : 5 dr and >99 : 1 er.

## Abstract 2

The isothiourea-catalysed enantioselective synthesis of tetrahydroindolizine derivatives containing three stereocentres is reported through a one-pot tandem sequential process.

## Graphical abstract

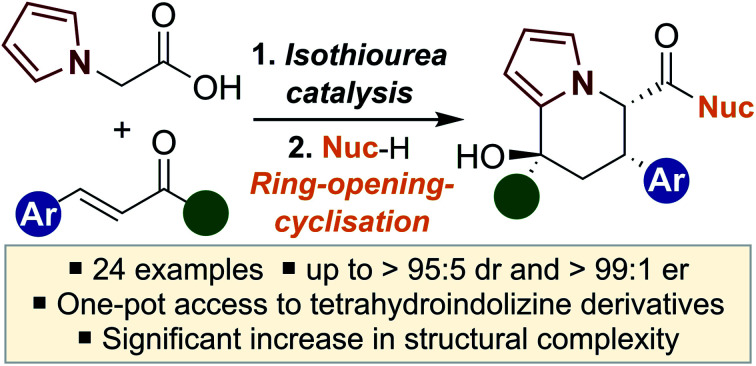

## 1. Introduction

**Fig. 1:**
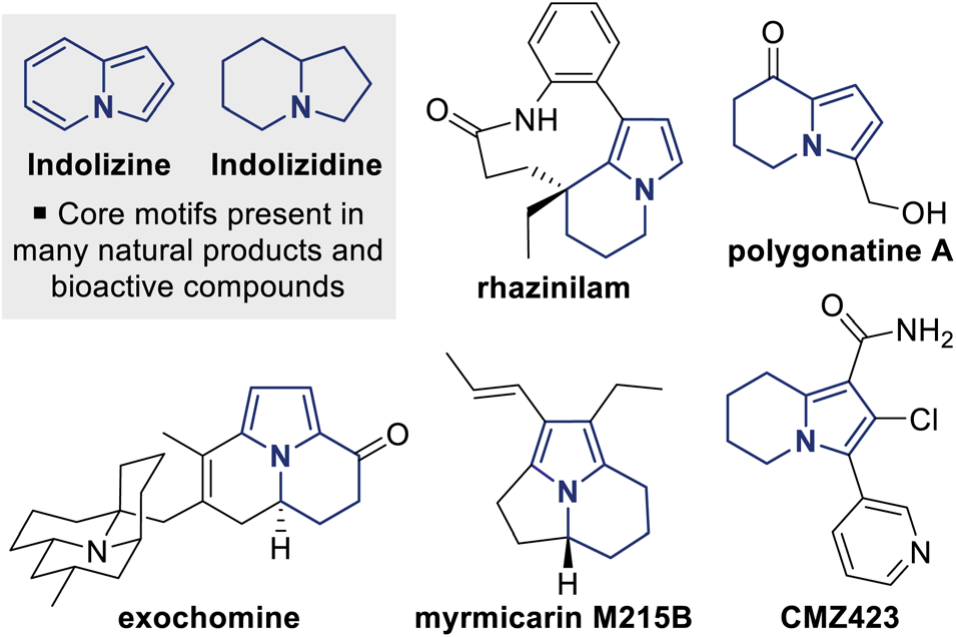
Tetrahydroindolizine cores present within natural products and bioactive compounds.

Pyrrole derivatives are found as a common structural motif within many natural products that can display a wide range of biological activities.^1^ The related but structurally more complex tetrahydroindolizine framework, which contains a fused pyrrole–piperidine core, is present in a range of naturally-occurring and synthetic bioactive molecules, and has also been utilised within key intermediates for the synthesis of indolizidine alkaloids (Fig. 1).^2^ A number of catalytic enantioselective methods for synthesising tetrahydroindolizine derivatives have been developed,^3^ with the majority of enantioselective syntheses focusing on applications towards rhazinilam.^2*b*,3*a*,*b*,*h*,*i*^ Catalytic enantioselective methodologies that provide access to polyfunctionalised tetrahydroindolizine architectures from simple starting materials are much less developed, but have the potential to allow greater exploration of 3D chemical space around the tetrahydroindolizine core.^4^

**Scheme 1:**
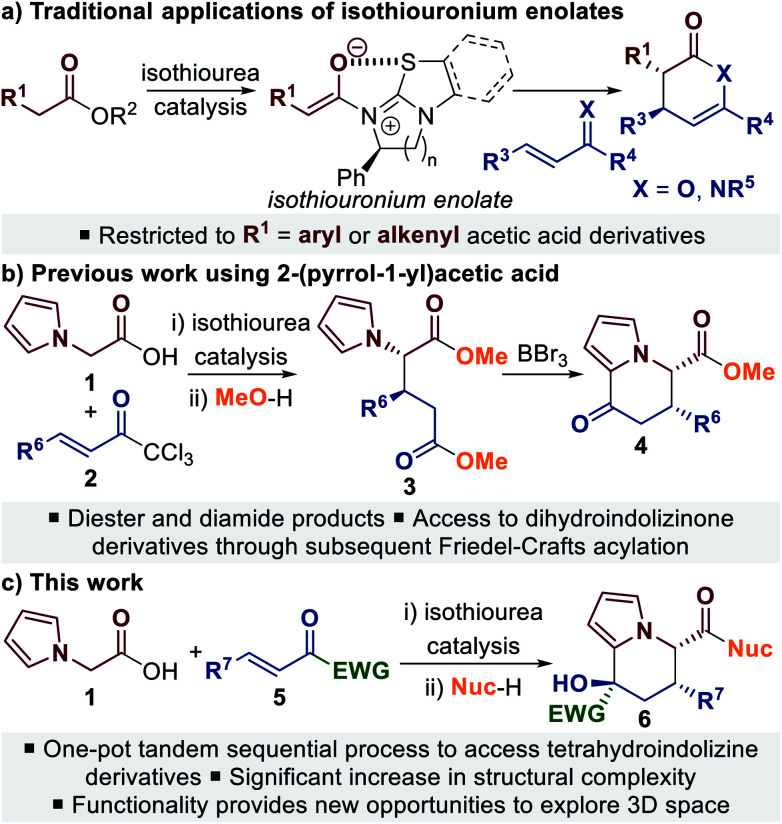
Applications of isothiouronium enolate catalysis in Michael addition–lactonisation/lactamisation processes.

C(1)-Ammonium enolate catalysis has emerged as a powerful method for the stereocontrolled synthesis of carbocyclic and heterocyclic compounds, through both intra- and intermolecular processes.^5^ C(1)-Ammonium enolates can be generated *in situ* from a range of carboxylic acid derivatives and have been most widely applied in formal cycloaddition processes, in which an aldol or Michael addition is followed by a lactonisation or lactamisation event.^5*a*,*b*^ Within this field, C(1)-isothiouronium enolates, generated from Lewis basic isothiourea catalysts,^6^ have been shown to be particularly effective for Michael addition–lactonisation/lactamisation processes (Scheme 1a).^7,8^ Using either enones or α,β-unsaturated ketimines as the Michael acceptor provides access to dihydropyranone or dihydropyridinone derivatives with excellent stereocontrol. Although a wide variety of Michael acceptors have been applied,^7^ a limitation for intermolecular processes is the common requirement for the isothiouronium enolate precursor to be an acetic acid derivative bearing an α-aryl or α-alkenyl substituent.

To address this limitation we recently reported the use of 2-(pyrrol-1-yl)acetic acid **1** in an isothiourea-catalysed Michael addition–lactonisation process using trichloromethyl enones **2** (Scheme 1b).^9^ The dihydropyranone products were unstable, however ring-opening using an alcohol or amine allowed isolation of diester **3** or diamide products with excellent stereoselectivity. Taking advantage of the inherent reactivity of pyrroles, the diester products could be further transformed in a separate reaction step through BBr_3_-promoted Friedel–Crafts acylation.^9,10^ Building upon this work, in this manuscript we report the development of a one-pot tandem sequential process^11^ to generate highly-functionalised tetrahydroindolizine derivatives **6** bearing three stereocentres. It was envisaged that the use of alternative Michael acceptors in an enantioselective Michael addition–lactonisation process, followed by ring-opening of the dihydropyranone to reveal a suitably electrophilic ketone, would allow a spontaneous cyclisation reaction with the pendant pyrrole to take place. Herein we describe the use of trifluoromethyl enones and α-keto-β,γ-unsaturated esters in this process, leading to a range of tetrahydroindolizine derivatives in up to >95 : 5 dr and >99 : 1 er (Scheme 1c).

## 2. Results and discussion

### 2.1 Reaction optimisation

Initial studies focussed on optimising the isothiourea-catalysed Michael addition–lactonisation process using 2-(pyrrol-1-yl)acetic acid **1** and trifluoromethyl enone **7** (Table 1).^12^ Treatment of 2-(pyrrol-1-yl)acetic acid **1** with pivaloyl chloride, to give a mixed anhydride *in situ*, followed by addition of HyperBTM **11** and enone **7** in MeCN at room temperature gave full conversion to a mixture of three compounds **8–10** (entry 1). Surprisingly, the expected dihydropyranone product **8** was only a minor component (∼20%) and was accompanied by the isomeric dihydropyranone **10** (65%) as the major component as well as β-lactone **9** (∼15%). In previous work using trifluoromethyl enone **7** in combination with aryl acetic acid derivatives,^7*d*,*i*,*n*^ no analogous β-lactone or isomeric dihydropyranone products were observed, indicating significantly different reaction pathways are accessible when using 2-(pyrrol-1-yl)acetic acid **1** as the isothiouronium enolate precursor. Separation of the three compounds was very challenging and therefore conditions for the selective generation of dihydropyranone **8** were targeted. An extensive solvent screen of 25 solvents was conducted, with only selected results summarised in Table 1 (see ESI‡ for full details). Conducting the reaction in DMF provided complete conversion to the isomeric dihydropyranone **10**, which was isolated in 97% and 80 : 20 er (entry 2).^13^ The use of CH_2_Cl_2_ at room temperature gave a 20 : 50 : 30 mixture of the three compounds, however conducting the reaction at −60 °C suppressed formation of the isomeric dihydropyranone **10** to give only **8** and **9** in a 50 : 50 ratio (entries 3 and 4). β-Lactone **9** was obtained from this mixture in 40% yield, 95 : 5 dr and 96 : 4 er,^14^ however dihydropyranone **8** could not be isolated. Ethereal solvents provided significantly enhanced selectivity for dihydropyranone **8** (entries 5–7), with the use of cyclopentylmethyl ether (CPME) giving **8** and **9** in an 85 : 15 ratio (entry 7). From this mixture, dihydropyranone **8** was isolated for the first time, albeit in low yield (18%), but with highly promising stereoselectivity (80 : 20 dr, 94 : 6 er). The low isolated yield was attributed to instability of the product on silica. At this point alternative isothiourea catalysts **12** and **13** were tested, however lower conversion and product selectivity was observed (entries 8 and 9). Further solvent screening using HyperBTM **11** as catalyst identified that acetates were also good solvents (entries 10 and 11), with the use of *i*-PrOAc providing a 90 : 5 : 5 ratio of **8** : **9** : **10** and allowing isolation of **8** in 20% yield, 80 : 20 dr and 95 : 5 er (entry 11). Although the precise effect of solvent in this reaction cannot be definitively stated, the formation of isomeric dihydropyranone **10** appears to be favoured in solvents with higher dielectric constants (*ε* ≥ 10).

**Table 1:** Reaction optimisation I: selectivity for formation of dihydropyranone **8**

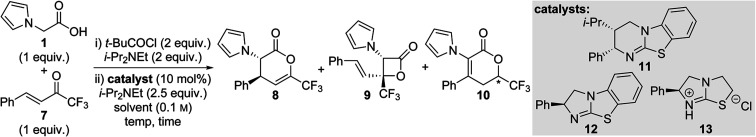							
Entry	Solvent	Catalyst	Conversion (%)	Ratio^a^ (**8** : **9** : **10**)	Yield^b^ (%)	dr^a^	er^c^
1	MeCN	**11**	100	20 : 15 : 65	—		—
2	DMF	**11**	100	0 : 0 : 100	**10**: 97		80 : 20
3	CH_2_Cl_2_	**11**	100	20 : 50 : 30			
4^d^	CH_2_Cl_2_	**11**	95	50 : 50 : 0	**9**: 40	95 : 5	96 : 4
5	Et_2_O	**11**	90	75 : 15 : 10			
6	MTBE	**11**	63	65 : 30 : 5			
7	CPME	**11**	97	85 : 15 : 0	**8**: 18	80 : 20	94 : 6
8	CPME	**12**	68	60 : 15 : 25			
9	CPME	**13**	70	60 : 15 : 25			
10	EtOAc	**11**	90	75 : 5 : 20			
11	*i*-PrOAc	**11**	98	90 : 5 : 5	**8**: 20	80 : 20	95 : 5

a Determined by ^1^H NMR spectroscopic analysis of the crude reaction product mixture, with values rounded to nearest 5.

b Isolated yield of specified product.

c Determined by chiral stationary phase HPLC analysis.

d Reaction conducted at −60 °C, DMF = dimethylformamide, MTBE = methyl *t*-butyl ether, CPME = cyclopentyl methyl ether.

**Scheme 2:**
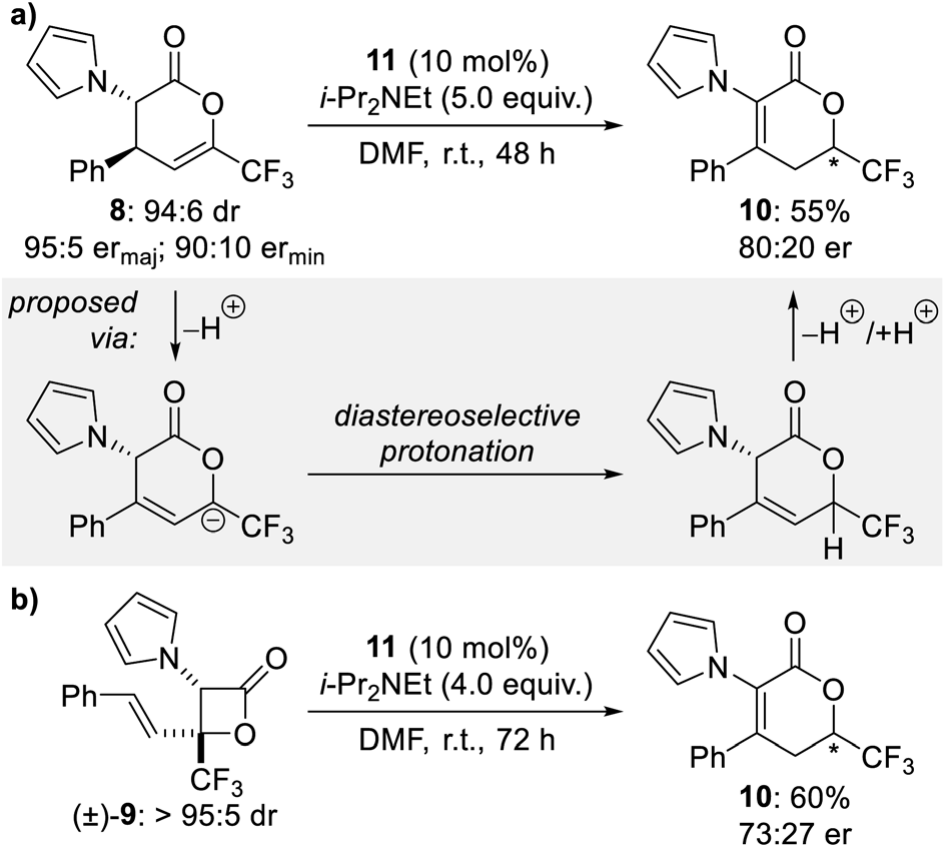
Control experiments.

Intrigued by the formation of β-lactone **9** and isomeric dihydropyranone **10** in these reactions, further studies were conducted to investigate their formation and stability under the reaction conditions. First, a diastereo- and enantioenriched sample of dihydropyranone **8** was treated with HyperBTM **11** and *i*-Pr_2_NEt in DMF at room temperature. The isomeric dihydropyranone **10** was obtained as the sole product and isolated in 55% yield and 80 : 20 er (Scheme 2a). Formation of this product can be rationalised by isomerisation of dihydropyranone **8** through a series of tautomerisations. The enantioenriched nature of isomeric dihydropyranone **10** indicates that protonation at C(6) is diastereoselective, with control presumably imparted by the stereocentre at C(3) (Scheme 2a, grey box). This isomerisation process would be expected to be more facile in solvents with higher dielectric constants, consistent with the trends in product ratio observed during reaction optimisation (see Table 1). Next, an isolated sample of racemic β-lactone (±)-**9** was treated with HyperBTM **11** and *i*-Pr_2_NEt in DMF at room temperature. Once again the isomeric dihydropyranone **10** was obtained as the sole product and isolated in 60% yield and 73 : 27 er (Scheme 2b). Generation of the isomeric dihydropyranone **10** in enantioenriched form may be rationalised by initial transformation of β-lactone (±)-**9** to an enantioenriched sample of dihydropyranone **8**, followed by the proposed tautomerisation process. The proposed isomerisation of racemic β-lactone (±)-**9** to enantioenriched dihydropyranone **8** may be explained by a HyperBTM-promoted formal retro-[2 + 2] cycloaddition to regenerate the isothiouronium enolate and enone **7**, which may then recombine to give enantioenriched dihydropyranone **8**.

Having identified CPME and *i*-PrOAc as the optimal solvents for the generation of enantioenriched dihydropyranone **8**, optimisation of the one-pot tandem sequential process to give tetrahydroindolizine derivatives was targeted. Upon completion of the isothiourea-catalysed reaction, methanol and catalytic DMAP was added to initiate the proposed ring-opening–cyclisation process (Table 2). Pleasingly, tetrahydroindolizine **14** was obtained in both solvents in good yield (71–88%) and with high enantioselectivity for the major diastereoisomer (entries 1 and 2). Only two diastereoisomers were observed, indicating high diastereocontrol in the generation of the new stereocentre at C(8). Conducting the reaction at −40 °C provided enhanced stereoselectivity (entries 3 and 4), with the use of *i*-PrOAc proving optimal to give tetrahydroindolizine **14** in 83% yield, 90 : 10 dr, 98 : 2 er for the major diastereoisomer and 96 : 4 er for the minor diastereoisomer (entry 4).

**Table 2:** Reaction optimisation II: formation of tetrahydroindolizine **14**

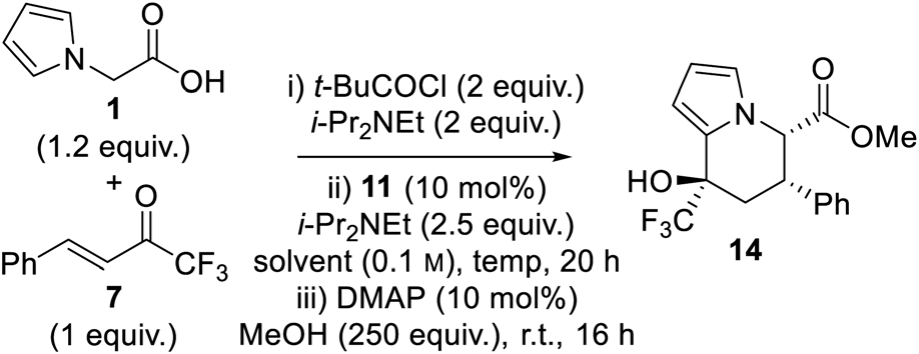						
Entry	Solvent	Temp.	Yield (%)	dr^a^	er_maj_^b^	er_min_^b^
1	CPME	r.t.	88	75 : 25	93 : 7	81 : 19
2	*i*-PrOAc	r.t.	71	70 : 30	97 : 3	68 : 32
3	CPME	−40 °C	76	90 : 10	94 : 6	92 : 8
4	*i*-PrOAc	−40 °C	83	90 : 10	98 : 2	96 : 4

a Determined by ^1^H and ^19^F NMR spectroscopic analysis of the crude reaction product mixture, with values rounded to nearest 5.

b Determined by chiral stationary phase HPLC analysis. CPME = cyclopentyl methyl ether. DMAP = 4-(dimethylamino)pyridine.

**Fig. 2:**
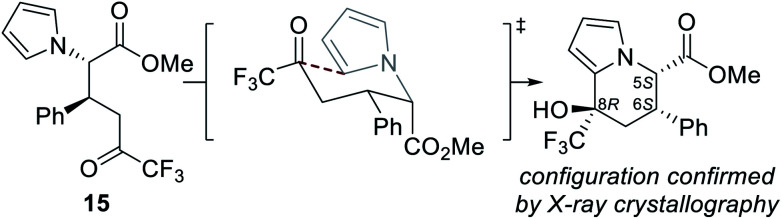
Rationale for configuration of the major diastereoisomer of tetrahydroindolizine.

The absolute (5*S*,6*S*,8*R*)-configuration of the major diastereoisomer of tetrahydroindolizine was confirmed by single crystal X-ray crystallographic analysis of derivative **21**.^15^ Based on previous literature precedent, the observed configurations at C(5) and C(6) are consistent with those expected to be generated in the isothiourea-catalysed Michael addition–lactonisation process.^7*d*,*i*,*n*^ The absolute configuration generated at C(8) may be rationalised by cyclisation of the ring-opened product **15** proceeding through a chair-like transition state, in which both the phenyl and CF_3_ groups occupy pseudo-equatorial positions (Fig. 2).^16^

### 2.2 Reaction scope and limitations

#### 2.2.1 Trifluoromethyl enone Michael acceptors

The scope of the transformation was investigated using different nucleophiles to promote ring-opening–cyclisation, and by applying a range of aryl- and heteroaryl-substituted trifluoromethyl enones (Table 3). Variation of the nucleophile demonstrated that amines were equally applicable, with amide-functionalised tetrahydroindolizines **16** and **17** obtained in good yield and with excellent stereoselectivity. The scope with respect to the trifluoromethyl enone was investigated using methanol as nucleophile for the ring-opening–cyclisation sequence. Incorporation of both electron-withdrawing and -donating groups at the 4- and 3-positions of the β-aryl ring of the trifluoromethyl enone was tolerated, with tetrahydroindolizines **18–22** obtained in 40–68% yield, and with good diastereoselectivity and excellent enantioselectivity (96 : 4 to 99 : 1 er). Incorporation of a sterically-imposing *ortho*-tolyl substituent at the β-position of the enone resulted in a significantly lower yield of tetrahydroindolizine **23** (25%), however excellent enantioselectivity (>99 : 1 er) for the major diastereoisomer was still observed. Electron neutral 1-naphthyl- and 2-naphthyl-substituted enones were also tolerated, giving tetrahydroindolizines **24** and **25** in good yield and excellent er (both 98 : 2 er). Incorporation of a 2-furyl group at the β-position of the enone also led to tetrahydroindolizine **26** in excellent yield and er (95%, 97 : 3 er_major_).

**Table 3:** Reaction scope I: trifluoromethyl enone Michael acceptors^a^

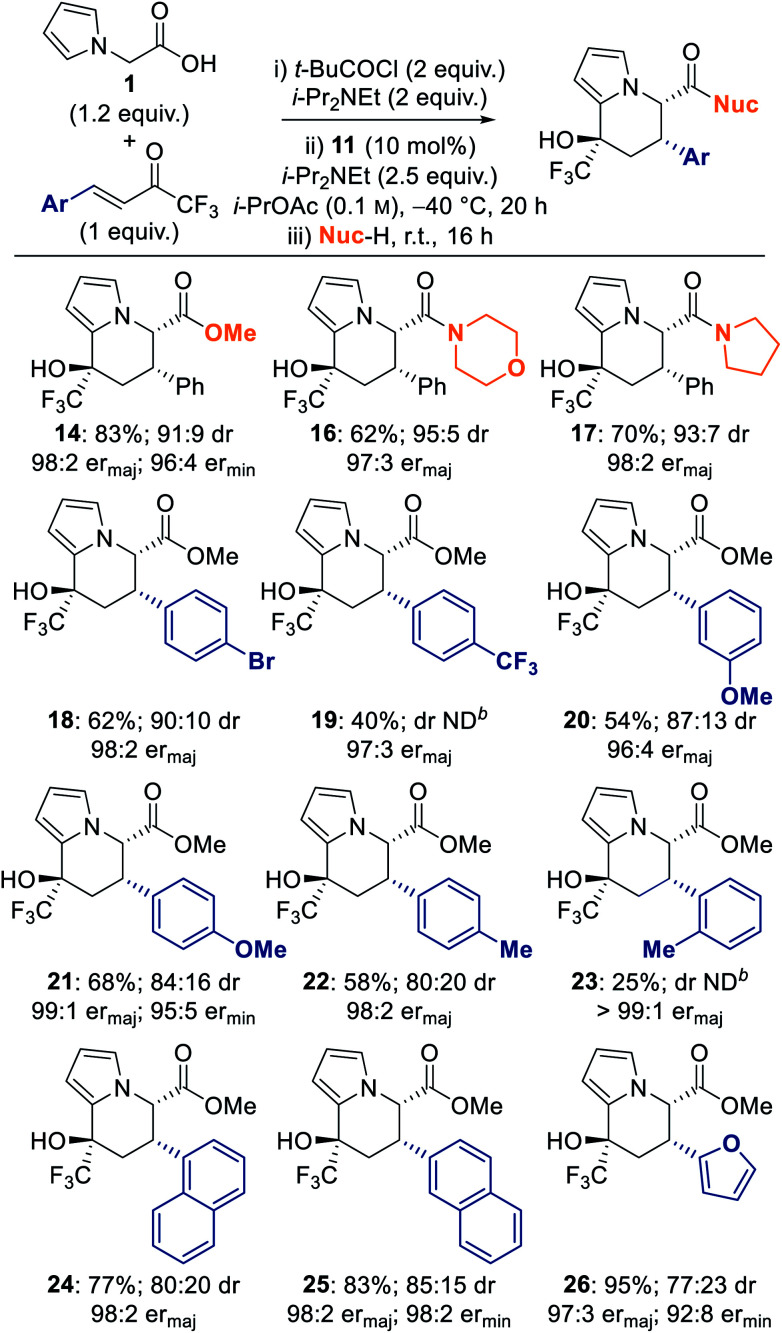

a dr determined by ^1^H and ^19^F NMR spectroscopic analysis of the crude reaction product mixture, with values rounded to nearest 5; er determined by chiral stationary phase HPLC analysis.

b ND = could not be determined.

#### 2.2.2 α-Keto-β,γ-unsaturated ester Michael acceptors

**Scheme 3:**
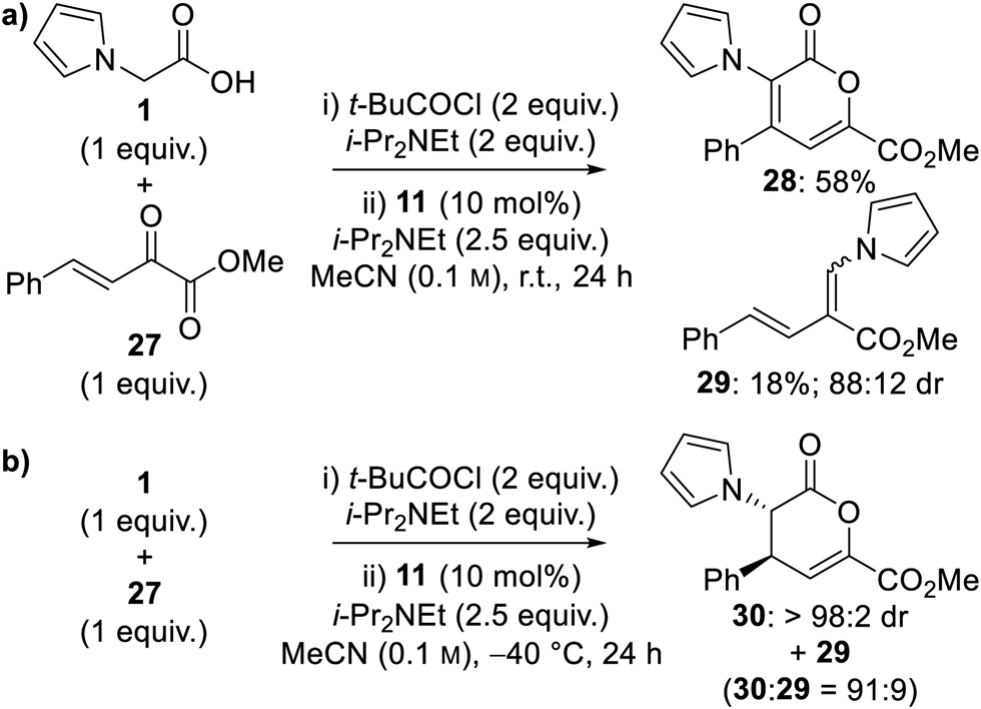
Initial optimisation using α-keto-β,γ-unsaturated ester **27**.

**Scheme 4:**
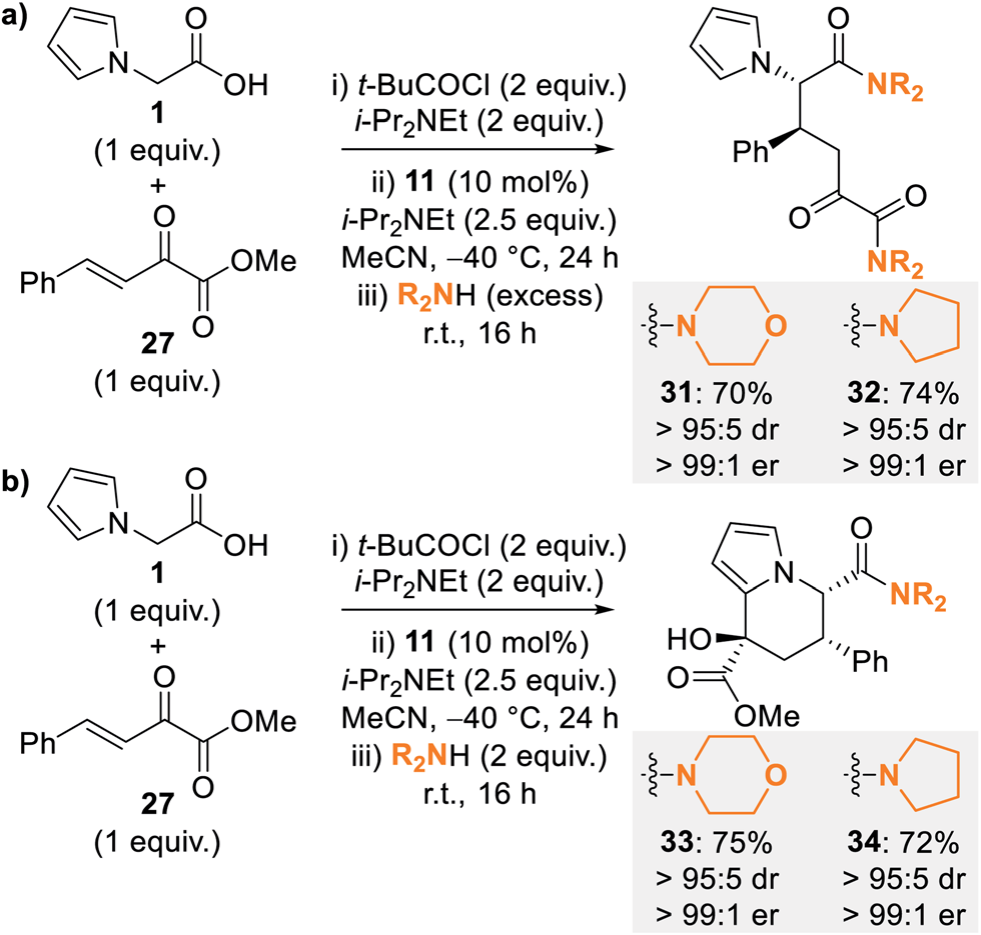
Optimisation of ring-opening–cyclisation process using amines.

Extension of this catalytic tandem sequential approach to tetrahydroindolizine derivatives was targeted by using α-keto-β,γ-unsaturated esters as the Michael acceptor.^7*a*,*i*^ A brief optimisation was first conducted (Schemes 3 and 4). Treatment of 2-(pyrrol-1-yl)acetic acid **1** with pivaloyl chloride, to give a mixed anhydride *in situ*, followed by addition of HyperBTM **11** and α-keto-β,γ-unsaturated ester **27** in MeCN at room temperature led to full conversion to pyranone **28** and diene **29** (Scheme 3a). In contrast to the analogous experiment using trifluoromethyl enone **7** (Table 1, entry 1), no dihydropyranone or β-lactone products were observed. It was suspected that pyranone **28** arose from oxidation of a transiently-formed dihydropyranone, whilst diene **29** most likely originated from decarboxylation of a β-lactone. Conducting the reaction at −40 °C suppressed formation of pyranone **28**, with dihydropyranone **30** and diene **29** obtained in a 91 : 9 ratio, however dihydropyranone **30** proved unstable to chromatographic purification (Scheme 3b). The use of alternative solvents provided no further improvement in product distribution.

Due to the difficulties in isolating dihydropyranone **30**, optimisation of the nucleophile-promoted ring-opening–cyclisation process to give tetrahydroindolizine derivatives was probed. Following completion of the isothiourea-catalysed reaction, addition of methanol as nucleophile provided a complex mixture of products. In contrast, the use of excess secondary amines (morpholine and pyrrolidine) provided diamides **31** and **32** as the sole products in high yield and with excellent stereoselectivity (>95 : 5 dr, >99 : 1 er) (Scheme 4a). To favour cyclisation to give tetrahydroindolizine derivatives, the equivalents of amine nucleophile were reduced, with the use of 2 equivalents proving optimal to give tetrahydroindolizines **33** and **34** in high yield and excellent stereocontrol (>95 : 5 dr, >99 : 1 er) (Scheme 4b).

The scope of the transformation was probed using a range of α-keto-β,γ-unsaturated esters, and employing morpholine (2 equiv.) as the nucleophile in the ring-opening–cyclisation process (Table 4). In all cases the tetrahydroindolizine products were obtained with consistently excellent diastereo- and enantioselectivity (>95 : 5 dr and >99 : 1 er in each case). Variation of the ester of the α-keto-β,γ-unsaturated ester to ethyl and isopropyl ester derivatives was tolerated, giving tetrahydroindolizines **35** and **36** in good yield (57% and 64%). Variation of the γ-aryl substituent demonstrated that the incorporation of both electron-donating and -withdrawing groups was well tolerated and provided tetrahydroindolizines **37–43** in moderate to excellent yield (50–98%). The incorporation of *ortho*-substituted and heteroaromatic γ-aryl substituents was also successful to give **44** and **45** in 58% and 62% yield, respectively. Suitable single crystals could not be obtained to unambiguously assign the configuration of these tetrahydroindolizine products, however based on literature precedent^7*a*,*i*^ and similarities in ^1^H coupling constants,^12^ the absolute and relative configurations were assigned by analogy to the trifluoromethyl-substituted tetrahydroindolizine series.

**Table 4:** Reaction scope II: α-keto-β,γ-unsaturated ester Michael acceptors^a^

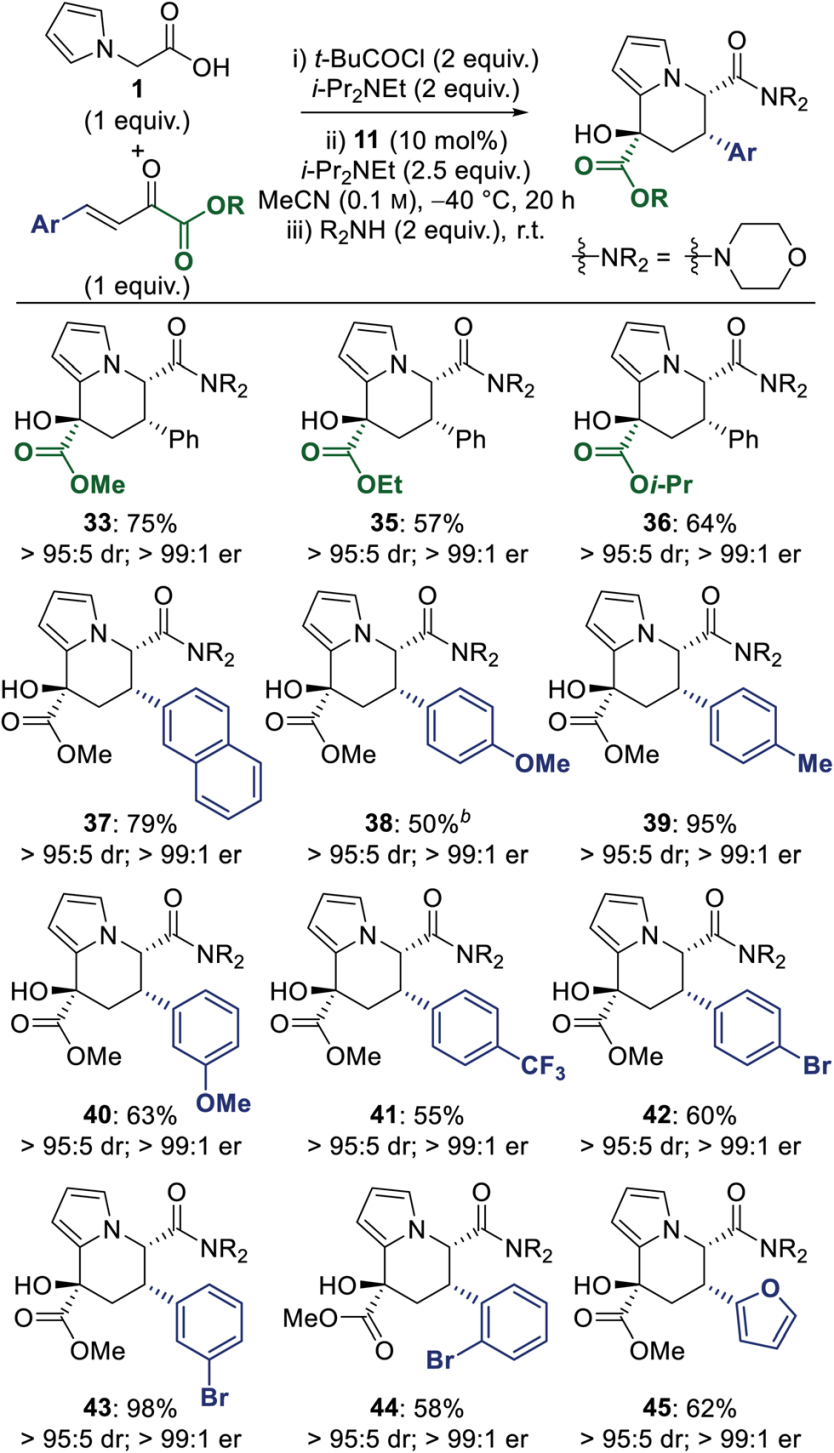

a dr determined by ^1^H NMR spectroscopic analysis of the crude reaction product mixture, with values rounded to nearest 5; er determined by chiral stationary phase HPLC analysis.

b CH_2_Cl_2_ used as solvent due to low solubility of α-keto-β,γ-unsaturated ester in MeCN.

### 2.3 Proposed mechanism

**Scheme 5:**
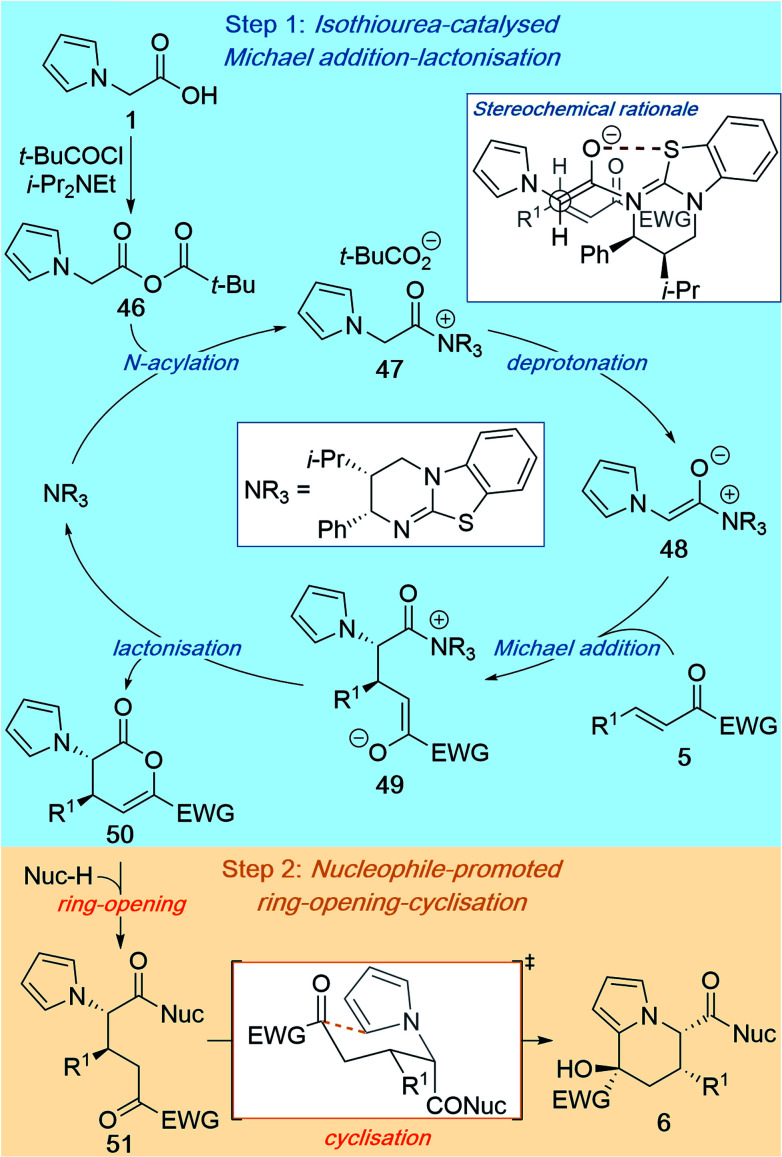
Proposed mechanism.

The mechanism of this reaction can be proposed based on previous related studies^7^ and the results presented in this manuscript (Scheme 5). The isothiourea-catalysed Michael addition–lactonisation is initiated by *N*-acylation of the Lewis basic isothiourea by mixed anhydride **46**, which is generated *in situ* from 2-(pyrrol-1-yl)acetic acid **1** and pivaloyl chloride, to give acyl ammonium **47** (Scheme 5, Step 1). Deprotonation to give ammonium enolate **48** is most likely facilitated by the pivalate counterion,^7*d*,14^ with *i*-Pr_2_NEt operating as an auxiliary base to buffer the pivalic acid by-product. Ammonium enolate **48** then undergoes stereoselective Michael addition to enone **5** to give zwitterionic intermediate **49**, which following lactonisation gives dihydropyranone **50** and regenerates the isothiourea catalyst. The stereochemical outcome of the reaction is determined in the Michael addition step, and can be rationalised through C–C bond formation between the *Si* face of the ammonium enolate and the *Si* face of the enone (Scheme 5, top right box). Reaction on the *Si* face of the ammonium enolate can be explained through three factors: (i) generation of the (*Z*)-ammonium enolate; (ii) a stabilising 1,5-O⋯S interaction favouring a *syn*-coplanar orientation between the enolate O and the S embedded within the catalyst;^17,18^ and (iii) the pseudo-axial phenyl substituent of the catalyst blocking the *Re* face of the enolate. The diastereoselectivity can be explained by adoption of a staggered transition state in which non-bonding steric contacts are minimised (Scheme 5, top right box). Following the catalytic reaction, the ring-opening–cyclisation process is initiated through addition of a nucleophilic amine or alcohol to give intermediate **51** (Scheme 5, Step 2). Spontaneous cyclisation of the pyrrole onto the electrophilic ketone within intermediate **51** is proposed to proceed through a chair-like transition state, in which both the R^1^ group and ketone substituent occupy pseudo-equatorial positions, to give the final tetrahydroindolizine product **6**.

## 3. Conclusions

In conclusion, an isothiourea-catalysed one-pot tandem sequential process has been developed for the enantioselective synthesis of highly-functionalised tetrahydroindolizine derivatives. The application of 2-(pyrrol-1-yl)acetic acid, with a suitable Michael acceptor, in an enantioselective Michael addition–lactonisation process was followed by ring-opening with an alcohol or amine. The transient ring-opened intermediate underwent spontaneous cyclisation of the pyrrole onto the pendant ketone to give tetrahydroindolizine products bearing 3 stereogenic centres. The use of trifluoromethyl enones as the Michael acceptor led to 12 tetrahydroindolizine products, bearing a CF_3_ group at a stereogenic centre, in 25–95% yield and up to 95 : 5 dr and 99 : 1 er. The use of α-keto-β,γ-unsaturated esters provided a further 12 tetrahydroindolizines in 50–98% yield, with each product obtained essentially as a single stereoisomer (>95 : 5 dr, >99 : 1 er). Current work is focused on further applications using 2-(pyrrol-1-yl)acetic acid, and other α-amino acetic acid derivatives, in isothiourea-catalysed enantioselective transformations.

## Author contributions

S. Z. conducted all synthetic work. M. D. G. prepared graphics and wrote the manuscript. A. M. Z. S. conducted X-ray crystal structure analysis and refinement. A. D. S. conceptualised and supervised the project, and provided critical feedback during manuscript preparation.

## Conflicts of interest

There are no conflicts to declare.

## Supplementary Material

SC-011-D0SC00432D-s001

SC-011-D0SC00432D-s002
