# Supplementary material for: Tandem sequential catalytic enantioselective synthesis of highly-functionalised tetrahydroindolizine derivatives
Source: Chem Sci. 2020 Mar 12;11(15):3885–92. doi: 10.1039/d0sc00432d (PMC8152628; doi:10.1039/d0sc00432d)
Supplement: SC-011-D0SC00432D-s001 [file SC-011-D0SC00432D-s001.pdf]

Electronic Supplementary Material (ESI) for Chemical Science.
This journal is © The Royal Society of Chemistry 2020

Tandem sequential catalytic enantioselective synthesis of highly-functionalised tetrahydroindolizine derivatives

Shuyue Zhang, Mark D. Greenhalgh, Alexandra M. Z. Slawin and Andrew D. Smith*
EaStCHEM, School of Chemistry, University of St Andrews, North Haugh, St Andrews, KY16 9ST, U.K.
e-mail: ads10@st-andrews.ac.uk

Supporting Information

Table of Contents

General Experimental S2
General Procedures S4
Synthesis of Substrates S5
Reaction Optimisation S10
Compound Data for Products of Catalysis S12
X-Ray Data and Stereochemical Rationale S38
NMR Spectral Traces for Novel Compounds S39
HPLC Traces S119
References S149

General Experimental

Reactions involving moisture sensitive reagents were carried out in flame-dried glassware under an N_2 atmosphere using standard vacuum line techniques. Anhydrous MeCN and DMF was purchased from Acros Organics. Petrol is defined as petroleum ether 40–60 °C. All other solvents and commercial reagents were used as received without further purification unless stated.

Room temperature (rt) refers to 20–25 °C. Temperatures of 0 °C and –78 °C were obtained using ice/water and CO_2 (s)/acetone baths, respectively. Temperatures of 0 °C to –78 °C for overnight reactions were obtained using an immersion cooler (HAAKE EK 90). Reactions involving heating were performed using DrySyn blocks and a contact thermocouple.

Under reduced pressure refers to the use of either a Büchi Rotavapor R-200 with a Büchi V-491 heating bath and Büchi V-800 vacuum controller, a Büchi Rotavapor R-210 with a Büchi V-491 heating bath and Büchi V-850 vacuum controller, a Heidolph Laborota 4001 with vacuum controller, an IKA RV10 rotary evaporator with an IKA HB10 heating bath and ILMVAC vacuum controller, or an IKA RV10 rotary evaporator with a IKA HB10 heating bath and Vacuubrand CVC3000 vacuum controller. Rotary evaporator condensers were fitted to Julabo FL601 Recirculating Coolers filled with ethylene glycol and set to –5 °C.

Analytical thin layer chromatography was performed on pre-coated aluminium plates (Kieselgel 60 F254 silica) and visualisation was achieved using ultraviolet light (254 nm) and/or staining with aqueous $KMnO_4$ solution followed by heating. Manual column chromatography was performed in glass columns fitted with porosity 3 sintered discs over Kieselgel 60 silica using the solvent system stated.

Melting points were recorded on an Electrothermal 9100 melting point apparatus.

Optical rotations were measured on a Perkin Elmer Precisely/Model-341 polarimeter operating at the sodium D line with a 100 mm path cell at 20 °C.

HPLC analyses were obtained on a Shimadzu HPLC consisting of a DGU-20A$_5$ degassing unit, LC-20AT liquid chromatography pump, SIL-20AHT autosampler, CMB-20A communications bus module, SPD-M20A diode array detector and a CTO-20A column oven. Separation was achieved using either DAICEL CHIRALCEL OD-H or DAICEL CHIRALPAK AD-H and IB columns using the method stated. HPLC

traces of enantiomerically enriched compounds were compared with authentic racemic spectra.

Infrared spectra were recorded on a Shimadzu IRAffinity-1 Fourier transform IR spectrophotometer fitted with a Specac Quest ATR accessory (diamond puck). Spectra were recorded of thin films, with characteristic absorption wavenumbers (ν_{max}) reported in cm^{-1}.

^{1}H, $^{13}C\{^{1}H\}$, ^{19}F and $^{19}F\{^{1}H\}$ NMR spectra were acquired on either a Bruker AV400 with a BBFO probe (^{1}H 400 MHz; ^{13}C 101 MHz; ^{19}F 377 MHz), a Bruker AVII 400 with a BBFO probe (^{1}H 400 MHz; ^{13}C 101 MHz; ^{19}F 377 MHz), a Bruker AVIII-HD 500 with a SmartProbe BBFO+ probe (^{1}H 500 MHz; ^{13}C 126 MHz; ^{19}F 471 MHz) or a Bruker AVIII 500 with a CryoProbe Prodigy BBO probe (^{1}H 500 MHz; ^{13}C 126 MHz; ^{19}F 471 MHz) in the deuterated solvent stated. All chemical shifts are quoted in parts per million (ppm) relative to the residual solvent peak. All coupling constants, *J*, are quoted in Hz. Multiplicities are indicated as s (singlet), d (doublet), t (triplet), q (quartet), p (pentet), m (multiplet), and multiples thereof. The abbreviation Ar denotes aromatic and app denotes apparent. NMR peak assignments were confirmed using 2D ^{1}H correlated spectroscopy (COSY), 2D ^{1}H-^{13}C heteronuclear multiple-bond correlation spectroscopy (HMBC), and 2D ^{1}H-^{13}C heteronuclear single quantum coherence (HSQC) where necessary.

Mass spectrometry (*m/z*) data were acquired by either electrospray ionisation (ESI), atmospheric solids analysis probe (ASAP) or nanospray ionisation (NSI) at either the University of St Andrews Mass Spectrometry Facility or at the EPSRC UK National Mass Spectrometry Facility at Swansea University.

General Procedures

General Procedure A: Synthesis of α-keto-β,γ-unsaturated ester

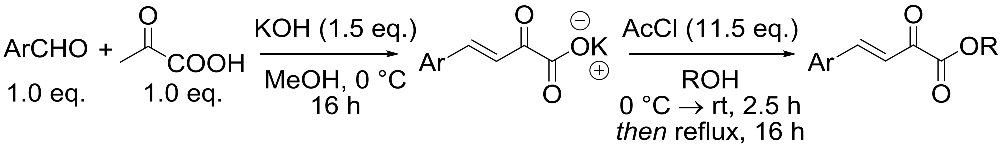

Following literature procedures,[1] to a solution of pyruvic acid (50 mmol, 1.0 eq.) and aldehyde (50 mmol, 1.0 eq.) in MeOH (5 mL) at 0 °C was added a solution of KOH (75 mmol, 1.5 eq.) in MeOH (15 mL). The first 1 eq. of the KOH solution was added dropwise over 30 minutes. The last 0.5 eq. of the KOH solution was added as one portion and the reaction mixture was stirred at 40 °C for 1 h followed by 0 °C for 16 h. The precipitate was collected by filtration, washed twice with cold MeOH, once with Et_2O and dried under vacuum to furnish the potassium salt that was used directly in the next step.

Acetyl chloride (400 mmol, 11.5 eq.) was added to the requisite alcohol (200 mL) at 0 °C to generate HCl. Potassium salt obtained from last step was added and the mixture stirred at 0 °C for 30 min then warmed to rt for 2 h before heating at reflux for 16 h. Concentration *in vacuo* gave a sticky solid which was dissolved in H_2O (50 mL) and extracted with CH_2Cl_2 (50 mL × 3). The combined organics were washed with saturated aq. $NaHCO_3$ (25 mL), H_2O (25 mL) and brine (25 mL) before being dried with $MgSO_4$. Concentration *in vacuo* afforded the crude reaction mixture, which was purified by flash column chromatography (EtOAc/petrol).

General Procedure B: Enantioselective synthesis of tetrahydroindolizine derivatives

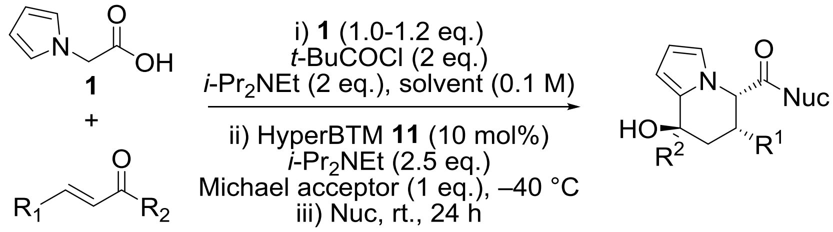

i-Pr_2NEt (2 eq.) and pivaloyl chloride (2 eq.) were added sequentially to a solution of 2-(1*H*-pyrrol-1-yl)acetic acid **1** (1.0-1.2 eq.) in the solvent stated (0.1 M) under an N_2 atmosphere at 0 °C. The reaction was stirred at 0 °C for 20 min then cooled to –40 °C. HyperBTM **11** (10 mol%), the required Michael acceptor (1 eq.), and *i*-Pr_2NEt (2.5 eq.) were added sequentially and the reaction stirred at –40 °C for the time stated. The reaction was quenched with the nucleophile stated at –40 °C, and stirred at rt for 24 h before being concentrated under reduced pressure to give the crude product, which was purified by flash silica column chromatography.

Synthesis of Substrates

Ethyl 2-(1*H*-pyrrol-1-yl)acetate (S1)

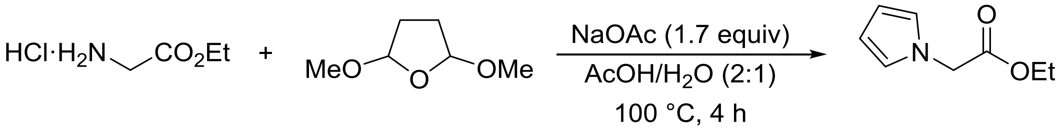

Glycine ethyl ester hydrochloride (2.50 g, 17.9 mmol) and NaOAc (2.45 g, 29.9 mmol) were suspended in H_2O (12.5 mL) and AcOH (25 mL) before 2,5-dimethoxytetrahydrofuran (2.32 mL, 17.9 mmol) was added. The reaction was heated at 100 °C for 4 h before being cooled to rt, poured into water (50 mL) and extracted with EtOAc (30 mL). The aqueous phase was neutralized with solid Na_2CO_3 and further extracted with EtOAc (2 × 30 mL). The combined organics were washed with water (50 mL), dried over $MgSO_4$, filtered, and concentrated under reduced pressure. The crude material was purified by column chromatography (Petrol/EtOAc 80:20, R_f 0.56) to give **S1** (1.80 g, 66%) as a colourless oil.

^{1}H NMR (400 MHz, $CDCl_3$) δ_H: 1.31 (3H, t, *J* 7.2, CH_2CH_3), 4.25 (2H, q, *J* 7.2, CH_2CH_3), 4.65 (2H, s, CH_2), 6.23 (2H, t, *J* 2.1, ArC(3,4)*H*), 6.69 (2H, t, *J* 2.1, ArC(2,5)*H*).

Spectroscopic data were in accordance with the literature.[2]

2-(1*H*-Pyrrol-1-yl)acetic acid (1)

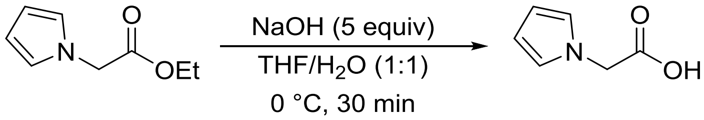

NaOH pellets (2.36 g, 59.0 mmol) were added to a solution of ester **S1** (1.80 g, 11.8 mmol) in 1:1 THF/H_2O (35 mL) at 0 °C. The reaction was stirred at 0 °C for 30 min before being washed with CH_2Cl_2 (40 mL). The aqueous phase was acidified with concentrated 12 M HCl to *ca.* pH 1 and extracted with CH_2Cl_2 (3 × 40 mL) before being dried over $MgSO_4$, filtered, and concentrated under reduced pressure to give **1** (1.43 g, 97%) as a colourless solid.

mp 90-92 °C{Lit.[3] 84-87 °C}; **^{1}H NMR** (500 MHz, $CDCl_3$) δ_H: 4.73 (2H, s, CH_2), 6.25 (2H, t, *J* 2.1, ArC(3,4)*H*), 6.69 (2H, t, *J* 2.1, ArC(2,5)*H*).

Spectroscopic data were in accordance with the literature.[2]

CF$_3$ enones

All substrates were synthesized according to a literature procedure,[4] and matched characterization data previously reported in the literature:

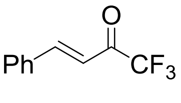

7[4]

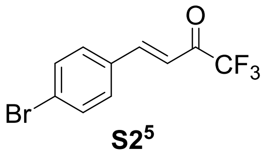

S2[5]

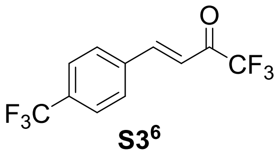

S3[6]

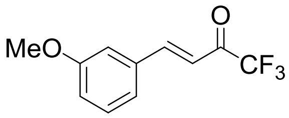

S4[5]

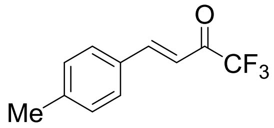

S5[5]

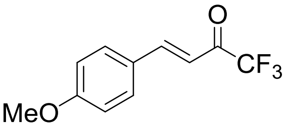

S6[4]

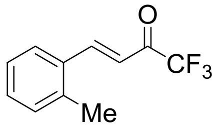

S7[4]

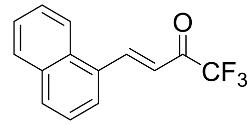

S8[7]

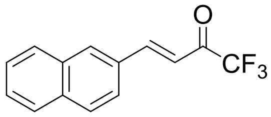

S9[4]

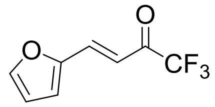

S10[5]

α-Keto-β,γ-unsaturated esters

Methyl (*E*)-2-oxo-4-phenylbut-3-enoate (27)

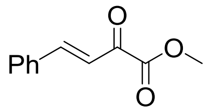

Following **General Procedure A**, the title compound was obtained as a yellow solid (35% yield over two steps). Spectroscopic data were in accordance with the literature.[1]

mp 68–70 °C {Lit.[1] 69–70 °C}; **^{1}H NMR** (400 MHz, CDCl$_3$) δ_H: 3.89 (3H, s, C*H*$_3$), 7.34 (1H, d, *J* 16.1, C(3)*H*), 7.34–7.44 (3H, m, ArC(3,5)*H* and ArC(4)*H*), 7.56–7.61 (2H, m, ArC(2,6)*H*), 7.82 (1H, d, *J* 16.1, C(4)*H*).

Ethyl (*E*)-2-oxo-4-phenylbut-3-enoate (S11)

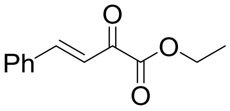

Following **General Procedure A**, the title compound was obtained as a yellow oil (25% yield over two steps). Spectroscopic data were in accordance with the literature.[1]

^{1}H NMR (400 MHz, CDCl$_3$) δ_H: 1.43 (3H, t, *J* 7.2, CH$_3$), 4.42 (2H, q, *J* 7.1, CH$_2$), 7.30 (1H, d, *J* 16.1, C(3)*H*), 7.38–7.46 (3H, m, Ar*H*), 7.62–7.69 (2H, m, Ar*H*), 7.83 (1H, d, *J* 16.1, C(4)*H*).

Isopropyl (*E*)-2-oxo-4-phenylbut-3-enoate (S12)

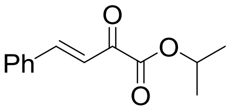

Following **General Procedure A**, the title compound was obtained as a yellow oil (21% yield over two steps). Spectroscopic data were in accordance with the literature.[1]

^{1}H NMR (400 MHz, $CDCl_3$) δ_H: 1.41 (6H, d, *J* 6.1, C*H*$_3$), 5.22 (1H, septet, *J* 6.1, CH), 7.34 (1H, d, *J* 16.2, C(3)*H*), 7.40–7.52 (3H, m, ArC*H*), 7.62–7.69 (2H, m, ArC*H*), 7.82 (1H, d, *J* 16.1, C(4)*H*).

Methyl (*E*)-4-(naphthalen-2-yl)-2-oxobut-3-enoate (S13)

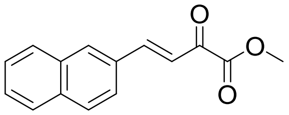

Following **General Procedure A**, the title compound was obtained as a yellow solid (40% yield over two steps). Spectroscopic data were in accordance with the literature.[1]

mp 67–69 °C {Lit.[1] 70–72 °C}; **^{1}H NMR** (400 MHz, $CDCl_3$) δ_H: 4.01 (3H, s, CH_3), 7.51 (1H, d, *J* 16.0, C(3)*H*), 7.52–7.61 (2H, m, ArC*H*), 7.75–7.84 (1H, m, ArC*H*), 7.87–7.95 (3H, m, ArC*H*), 8.03–8.12 (2H, m, C(4)*H* and ArC*H*).

Methyl (*E*)-2-oxo-4-(*p*-tolyl)but-3-enoate (S14)

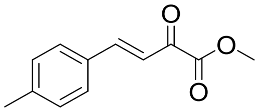

Following **General Procedure A**, the title compound was obtained as a yellow solid (41% yield over two steps). Spectroscopic data were in accordance with the literature.[8]

mp 80–81 °C {Lit.[9] 70–72 °C}; **^{1}H NMR** (400 MHz, $CDCl_3$) δ_H: 2.41 (3H, s, C*H*$_3$), 3.94 (3H, s, OCH_3), 7.22 (2H, d, *J* 7.8, ArC*H*), 7.32 (1H, d, *J* 16.1, C(3)*H*), 7.55 (2H, d, *J* 7.9, ArC*H*), 7.88 (1H, d, *J* 16.2, C(4)*H*).

Methyl (*E*)-4-(4-methoxyphenyl)-2-oxobut-3-enoate (S15)

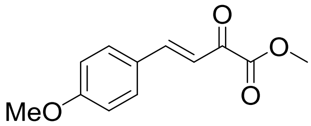

Following **General Procedure A**, the title compound was obtained as a yellow solid (25% yield over two steps). Spectroscopic data were in accordance with the literature.[1]

mp 84–86 °C {Lit.[1] 86–88 °C}; **^{1}H NMR** (400 MHz, $CDCl_3$) δ_H: 3.87 (3H, s, OC*H*$_3$), 3.96 (3H, s, OC*H*$_3$), 6.92–7.00 (2H, m, ArC*H*), 7.28 (1H, d, *J* 16.0, C(3)*H*), 7.58–7.64 (2H, m, ArC*H*), 7.88 (1H, d, *J* 16.0, C(4)*H*).

Methyl (*E*)-4-(3-methoxyphenyl)-2-oxobut-3-enoate (S16)

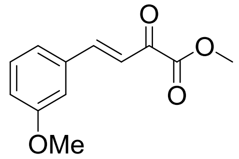

Following **General Procedure A**, the title compound was obtained as a yellow solid (25% yield over two steps). Spectroscopic data were in accordance with the literature.[8]

mp 83–84 °C {Lit.[9] 100–102 °C}; **^{1}H NMR** (400 MHz, $CDCl_3$) δ_H: 3.85 (3H, s, C*H*$_3$), 3.93 (3H, s, C*H*$_3$), 6.92–7.06 (1H, m, ArC*H*), 7.11–7.12 (1H, m, C(3)*H*), 7.19–7.23 (1H, m, ArC*H*), 7.26–7.36 (2H, m, ArC*H*), 7.82 (1H, d, *J* 16.1, C(4)*H*).

Methyl (*E*)-2-oxo-4-(4-(trifluoromethyl)phenyl)but-3-enoate (S17)

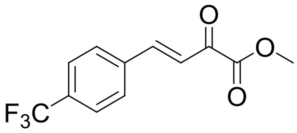

Following **General Procedure A**, the title compound was obtained as a yellow solid (30% yield over two steps). Spectroscopic data were in accordance with the literature.[10]

mp 116–117 °C {Lit.[10] 122–123 °C}; **^{1}H NMR** (400 MHz, $CDCl_3$) δ_H: 3.95 (3H, s, C*H*$_3$), 7.37 (1H, d, *J* 16.2, C(3)*H*), 7.71 (2H, d, *J* 8.2, C(4)ArC(2,6)*H*), 7.76 (2H, d, *J* 8.2, C(4)ArC(3,5)*H*), 7.89 (1H, d *J* 16.2, C(4)*H*).

Methyl (*E*)-4-(4-bromophenyl)-2-oxobut-3-enoate (S18)

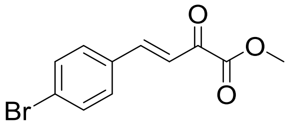

Following **General Procedure A**, the title compound was obtained as a yellow solid (28% yield over two steps). Spectroscopic data were in accordance with the literature.[1]

mp 115–116 °C {Lit.[1] 116–118 °C}; **^{1}H NMR** (400 MHz, $CDCl_3$) δ_H: 3.98 (3H, s, C*H*$_3$), 7.42 (1H, d, *J* 15.9, C(3)*H*), 7.48–7.54 (2H, m, ArC*H*), 7.55–7.64 (2H, m, ArC*H*), 7.81 (1H, d, *J* 15.9, C(4)*H*).

Methyl (*E*)-4-(3-bromophenyl)-2-oxobut-3-enoate (S19)

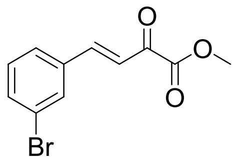

Following **General Procedure A**, the title compound was obtained as a yellow solid (34% yield over two steps). Spectroscopic data were in accordance with the literature.[1]

mp 103–104 °C {Lit.[1] 96–98 °C}; **^{1}H NMR** (400 MHz, $CDCl_3$) δ_H: 3.94 (3H, s, C*H*$_3$), 7.30 (1H, t, *J* 7.9,

ArC*H*), 7.37 (1H, d, *J* 16.2, C(3)*H*), 7.53–7.59 (2H, m, ArC*H*), 7.78 (1H, t, *J* 1.8, ArC*H*), 7.78 (1H, d, *J* 16.1, C(4)*H*).

Methyl (*E*)-4-(2-bromophenyl)-2-oxobut-3-enoate (S20)

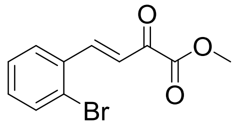

Following **General Procedure A**, the title compound was obtained as a yellow solid (26% yield over two steps). Spectroscopic data were in accordance with the literature.[10]

mp 50–52 °C {Lit.[10] 54–57 °C}; **^{1}H NMR** (400 MHz, $CDCl_3$) δ_H: 3.94 (3H, s, C*H*$_3$), 7.28–7.40 (3H, m, C(3)*H* + ArC*H*), 7.62–7.79 (2H, m, ArC*H*), 8.25 (1H, d, *J* 16.1, C(4)*H*).

Methyl (*E*)-4-(furan-2-yl)-2-oxobut-3-enoate (S21)

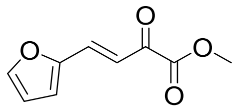

Following literature procedure,[1] to a solution of pyruvic acid (3.52 g, 50 mmol, 1.0 eq.) and furan-2-carbaldehyde (4.14 mL, 50 mmol, 1.0 eq.) in MeOH (5 mL) at 0 °C was added a solution of KOH (4.21 g, 75 mmol, 1.5 eq.) in MeOH (15 mL). The first 1 eq. of the KOH solution was added dropwise over 30 minutes. The last 0.5 eq. was added as one portion and the reaction mixture was stirred at 40 °C for 1 h followed by 0 °C for 16 h. The precipitate was collected by filtration, washed twice with cold MeOH, once with Et_2O and dried under vacuum to furnish the crude potassium salt, which was then all dissolved in DMF (40 mL). Methyl iodide (3.4 mL, 55 mmol, 1.1 eq.) was added and the reaction mixture was heated at 75 °C for 4 h. Once the reaction mixture was cooled to room temperature, H_2O (40 mL) was added and the organic layer was extracted with DCM (3 × 20 mL). The combined organic extracts were washed with H_2O (3 × 30 mL) and brine (3 × 30 mL), dried, filtered and concentrated under reduced pressure. Chromatographic purification (15:85 EtOAc/petrol) gave the title compound as a dark yellow solid (1.62 g, 18% over two steps). Spectroscopic data were in accordance with the literature.[1]

mp 51–53 °C; {Lit.[1] 56–58 °C}; **^{1}H NMR** (400 MHz, $CDCl_3$) δ_H: 3.92 (3H, s, C*H*$_3$), 6.54 (1H, dd, *J* 3.5, 1.8, ArC*H*), 6.82–6.84 (1H, m, ArC*H*), 7.23 (1H, d, *J* 15.7, C(3)*H*), 7.57–7.58 (1H, m, ArC*H*), 7.63 (1H, d, *J* 15.7, C(4)*H*).

Reaction Optimisation

Table S1: Solvent screening at room temperature

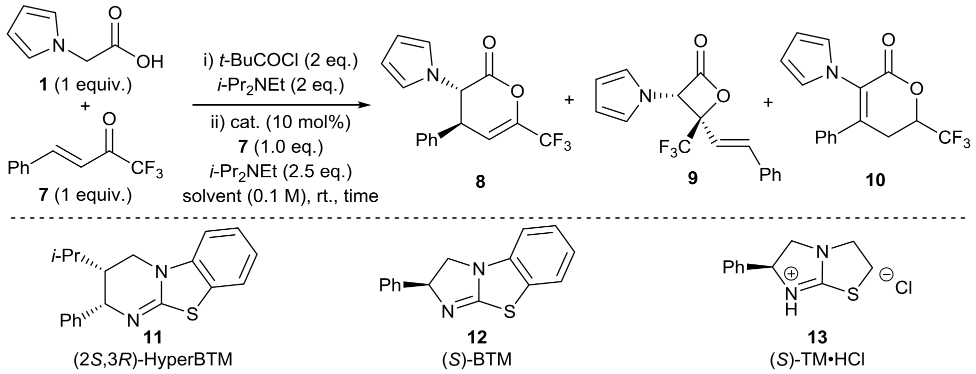

Entry	Cat.	Solvent	Time/ h	Conv.[a]	Product ratio 8:9:10[a,b]
**1**	13	DMF	24	100%	0:0:100 (95%, 20:80 er)
**2**	12	DMF	24	100%	0:0:100 (96%, 25:75 er)
**3**	11	DMF	24	100%	0:0:100 (97%, 80:20 er)
**4**	11	DMA	18	100%	0:0:100
**5**	11	DMSO	18	0%	-
**6**	11	$MeNO_2$	18	0%	-
**7**	11	cyclohexanone	18	100%	0:0:100
**8**	11	DCE	18	95%	5:5:90
**9**	11	MeCN	4	100%	20:15:65
**10**	11	CH_2Cl_2	18	100%	20:50:30
**11**	11	$CHCl_3$	18	<5%	n/d.
**12**	11	CCl_4	18	95%	60:15:25
**13**	11	DMC	18	85%	65 (15%, 96:4 er):25:10
**14**	11	2-MeTHF	18	85%	70:15:15
**15**	11	MTBE	18	63%	65:30:5
**16**	11	1,4-dioxane	18	83%	75:20:5
**17**	11	Et_2O	18	90%	75:15:10
**18**	11	THF	18	86%	75:15:10
**19**	11	1,2-dimethoxyethane	18	0%	-
**20**	11	2,2-dimethoxypropane	18	0%	-
**21**	11	diglyme	18	0%	-
**22**	11	Toluene	18	92%	70:15:15
**23**	11	EtOAc	18	90%	75:10:20
**24**	11	*tert*-Amyl alcohol	18	88%	85 (21%, 60:40 er):15:0
**25**	11	hexafluoroisopropanol	18	0%	
**26**	11	CPME	18	97%	85 (18%, 94:6 er):15:0
**27**	11	*i*-PrOAc	18	98%	90 (20%, 95:5 er):5:5

a. Determined by ^1H NMR analysis of crude reaction mixture. b. Isolated yield and er for product in parentheses

Table S2: Solvent screening at low temperature

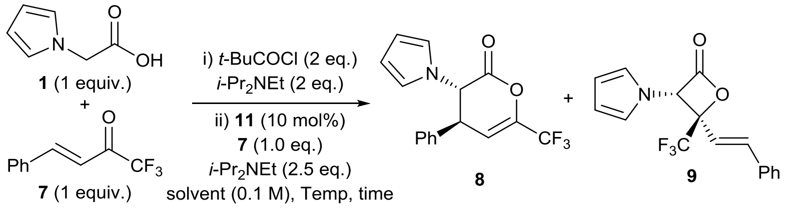

Entry	Solvent	Temp / °C	Time / h	Conv.[a]	Product ratio 8:9[a,b]
**1**	DMF	−60	18	70%	85:15
**2**	CH_2Cl_2	−60	15	95%	50:50 (40%, 96:4 er)
**3**	CH_2Cl_2	−78	24	85%	55:45
**4**	MeCN	−40	20	100%	85:15
**5**	CPME	−40	20	97%	84:16
**6**	*i*-PrOAc	−40	20	98%	90:10

a. Determined by ¹H NMR analysis of crude reaction mixture. b. Isolated yield and er for product in parentheses

Compound Data for Products of Catalysis

(3*S*,4*S*)-4-Phenyl-3-(1*H*-pyrrol-1-yl)-6-(trifluoromethyl)-3,4-dihydro-2*H*-pyran-2-one (8)

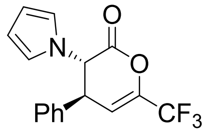

2-(1*H*-Pyrrol-1-yl)acetic acid **1** (38 mg, 0.30 mmol, 1.2 eq.) was dissolved in *i*-PrOAc (0.1 M) under an atmosphere of N_2 and cooled to 0 °C before *i*-Pr_2NEt (87 μL, 0.5 mmol, 2 eq.) and pivaloyl chloride (62 μL, 0.5 mmol, 2 eq.) were added. The reaction was stirred at 0 °C for 20 min before warming to rt. HyperBTM **11** (7.7 mg, 10 mol%), CF_3 enone **7** (50 mg, 0.25 mmol, 1 eq.) and *i*-Pr_2NEt (110 μL, 0.63 mmol, 2.5 eq.) were added sequentially and the reaction stirred at rt for 18 h. Then the mixture was concentrated under reduced pressure to give the crude product (82:18 dr) which was purified by flash silica column chromatography (5:95 Et_2O/petrol) and recrystallization from hexane to give the title compound as fine colourless needles (15 mg, 20%).

mp 83–85 °C; **Chiral HPLC analysis**, Chiralpak AD-H (97.5:2.5 hexane/*i*-PrOH, flow rate 1 mLmin^{-1}, 211 nm, 30 °C) t_R (major): 13.6 min, t_R (minor): 19.1 min, 95:5 er; **IR** ν_{max} (film, cm^{-1}) 1782 (C=O), 1701; **^{1}H NMR** (400 MHz, $CDCl_3$) δ_H: 4.26 (1H, app dp, *J* 12.4, 2.8, C(4)*H*), 4.85 (1H, d, *J* 12.4, C(3)*H*), 6.12 (2H, t, *J* 2.2, C(3)Ar(3,4)*H*), 6.15 (1H, dq, *J* 2.4, 0.8, C(5)*H*), 6.48 (2H, t, *J* 2.2, C(3)Ar(2,5)*H*), 6.98–7.01 (2H, m, PhC(2,6)*H*), 7.27–7.31 (3H, m, PhC(3,4,5)*H*); **^{13}C{^{1}H} NMR** (126 MHz, $CDCl_3$) δ_C: 44.7 (*C*(4)H), 62.7 (*C*(3)H), 110.0 (C(3)Ar*C*(2,5)H), 110.7 (q, $^3J_{CF}$ 3.4, *C*(5)H), 118.3 (q, $^1J_{CF}$ 272.0, *C*F_3), 120.3 (C(3)Ar*C*(3,4)H), 127.2 (Ph*C*(2,6)H), 128.7 (Ph*C*(3,5)H), 129.4 (Ph*C*(4)H), 137.0 (Ph*C*(1)), 140.8 (q, $^2J_{CF}$ 38.9, *C*(6)), 163.2 (*C*(2)); **^{19}F{^{1}H} NMR** (377 MHz, $CDCl_3$) δ_F: −72.1 (C*F*$_3$); **HRMS** (NSI$^+$) $C_{16}H_{13}F_3NO_2$ [M+H]$^+$ found 308.0895, requires 308.0893 (+0.7 ppm); $[\alpha]_D^{20}$ not measured due to product instability.

Selected data for minor diastereoisomer: **Chiral HPLC analysis**, Chiralpak AD-H (97.5:2.5 hexane/*i*-PrOH, flow rate 1 mLmin^{-1}, 211 nm, 30 °C) t_R (major): 15.3 min, t_R (minor): 22.3 min, 90:10 er; **^{1}H NMR** (400 MHz, $CDCl_3$) δ_H: 4.06–4.13 (1H, m, C(4)*H*), 5.49 (1H, d, *J* 7.3, C(3)*H*).

(3*S*,4*R*)-3-(1*H*-Pyrrol-1-yl)-4-((*E*)-styryl)-4-(trifluoromethyl)oxetan-2-one (9)

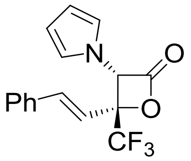

2-(1*H*-Pyrrol-1-yl)acetic acid **1** (31 mg, 0.25 mmol, 1 eq.) was dissolved in CH_2Cl_2 (0.1 M) under an atmosphere of N_2 and cooled to 0 °C before *i*-Pr_2NEt (87 μL, 0.5 mmol, 2 eq.) and pivaloyl chloride (62 μL, 0.5 mmol, 2 eq.) were added. The reaction was stirred at 0 °C for 20 min before cooling to –60 °C. HyperBTM **11** (7.7 mg, 10 mol%), CF_3 enone **7** (50 mg, 0.25 mmol, 1 eq.) and *i*-Pr_2NEt (110 μL,

0.63 mmol, 2.5 eq.) were added sequentially and the reaction stirred at –60 °C for 15 h. The mixture was concentrated under reduced pressure to give the crude product (> 95:5 dr) which was purified by flash silica column chromatography (10:90 Et_2O/petrol) to give the title compound as a colourless oil (31 mg, 40%).

Chiral HPLC analysis, Chiralpak IB (99:1 hexane/*i*-PrOH, flow rate 1 mLmin^{-1}, 211 nm, 30 °C) t_R (major): 9.6 min, t_R (minor): 8.8 min, 96:4 er; **^{1}H NMR** (500 MHz, $CDCl_3$) δ_H: 5.67 (1H, d, *J* 16.2, PhCH=C*H*), 6.06 (1H, s, C(3)*H*), 6.23 (2H, t, *J* 2.2, C(3)Ar(3,4)*H*), 6.63 (2H, t, *J* 2.2, C(3)Ar(2,5)*H*), 6.95 (1H, d, *J* 16.1, PhC*H*=CH), 7.22–7.24 (2H, m, ArC(2,6)*H*), 7.31–7.34 (3H, m, ArC(3,4,5)*H*); **^{13}C{^{1}H} NMR** (126 MHz, $CDCl_3$) δ_C: 70.6 (*C*(3)H), 81.4 (q, $^2J_{CF}$ 33.5, C(4)), 111.3 (C(3)Ar*C*(3,4)H), 113.3 (PhCH=*C*H), 121.0 (C(3)Ar*C*(2,5)H), 127.3 (Ph*C*(2,6)H), 127.8 (q, $^1J_{CF}$ 271.6, *C*F_3), 129.0 (Ph*C*(3,5)H), 129.6 (Ph*C*(4)H), 134.5 (Ph*C*H=CH), 139.1 (Ph*C*(1)), 162.7 (*C*(2)); **^{19}F NMR** (471 MHz, $CDCl_3$) δ_F: −78.8 (CF_3); **HRMS** (ASAP$^+$) $C_{16}H_{13}F_3NO_2$ $[M+H]^+$ found 308.0895, requires 308.0893 (+0.7 ppm); $[\alpha]_D^{20}$ and **IR** not measured due to product instability.

4-Phenyl-3-(1*H*-pyrrol-1-yl)-6-(trifluoromethyl)-5,6-dihydro-2*H*-pyran-2-one (10)

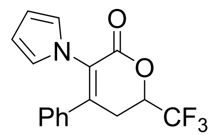

2-(1*H*-Pyrrol-1-yl)acetic acid **1** (31 mg, 0.25 mmol, 1 eq.) was dissolved in anhydrous DMF (0.1 M) under an atmosphere of N_2 and cooled to 0 °C before *i*-Pr_2NEt (87 μL, 0.5 mmol, 2 eq.) and pivaloyl chloride (62 μL, 0.5 mmol, 2 eq.) were added. The reaction was stirred at 0 °C for 20 min before warming to rt. HyperBTM **11** (7.7 mg, 10 mol%), CF_3 enone **7** (50 mg, 0.25 mmol, 1 eq.) and *i*-Pr_2NEt (110 μL, 0.63 mmol, 2.5 eq.) were added sequentially and the reaction stirred at rt for 24 h. Upon completion of the reaction (checked by TLC), it was diluted with CH_2Cl_2 (equal volume) and washed with 1 M HCl (× 2) and brine (× 2) before being dried over $MgSO_4$, filtered, and concentrated under reduced pressure to give the crude product, which was purified by flash silica column chromatography (15:85 EtOAc/petrol, R_f 0.33), to give the title compound as a yellow solid (75 mg, 97%).

mp 65–66 °C; **Chiral HPLC analysis**, Chiralcel OD-H (90:10 hexane/*i*-PrOH, flow rate 1 mLmin^{-1}, 254 nm, 30 °C) t_R (major): 14.4 min, t_R (minor): 10.1 min, 80:20 er; **IR** ν_{max} (film, cm^{-1}) 3102 (C-H stretch), 1728 (C=O stretch), 1614; **^{1}H NMR** (400 MHz, $CDCl_3$) δ_H: 3.14 (1H, dd, *J* 17.7, 4.3, C(5)H_AH_B), 3.33 (1H, dd, *J* 17.7, 11.3, C(5)H_AH_B), 5.04 (1H, dqd, *J* 11.3, 5.6, 4.3, C(6)*H*), 6.17 (2H, t, *J* 2.2, C(3)Ar(3,4)*H*), 6.48 (2H, t, *J* 2.2, C(3)Ar(2,5)*H*), 6.96–6.99 (2H, m, PhC(2,6)*H*), 7.27–7.37 (3H, m, PhC(3,4,5)*H*); **^{13}C{^{1}H} NMR** (101 MHz, $CDCl_3$) δ_C: 28.3 (*C*(5)H_2), 72.9 (q, $^2J_{CF}$ 34.4, *C*(6)H), 110.2 (C(3)Ar*C*(3,4)H), 122.1 (C(3)Ar*C*(2,5)H), 122.6 (q, $^1J_{CF}$ 280.2, *C*F_3), 125.3 (*C*(3)), 127.4 (Ph*C*(2,6)H), 128.9 (Ph*C*(3,5)H), 130.5

(Ph*C*(4)H), 134.4 (Ph*C*(1)), 144.6 (*C*(4)), 159.9 (*C*(2)); **^{19}F{^{1}H} NMR** (377 MHz, $CDCl_3$) δ_F: −78.0 (CF_3); **HRMS** (NSI^+) $C_{16}H_{13}F_3NO_2$ $[M+H]^+$ found 308.0894, requires 308.0893 (+0.4 ppm).

Methyl (5*S*,6*S*,8*R*)-8-hydroxy-6-phenyl-8-(trifluoromethyl)-5,6,7,8-tetrahydroindolizine-5-carboxylate (14)

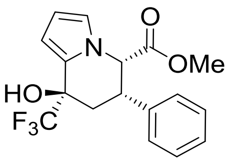

Following **General Procedure B**, 2-(1*H*-pyrrol-1-yl)acetic acid **1** (30 mg, 0.24 mmol, 1.2 eq.), *i*-Pr_2NEt (70 μL, 0.40 mmol), pivaloyl chloride (50 μL, 0.40 mmol) in *i*-PrOAc (2 mL) at 0 °C for 20 min followed by HyperBTM **11** (6.2 mg, 10 mol%), CF_3 enone **7** (40 mg, 0.20 mmol, 1.0 eq.) and *i*-Pr_2NEt (89 μL, 0.5 mmol) at –40 °C for 20 h. Ring-opening with MeOH (2 mL) and DMAP (4.9 mg, 20 mol%) at rt for 24 h gave crude product (91:9 dr) that was purified by column chromatography (10:90 EtOAc/petrol) to give the combined diastereoisomers (90:10 dr) (56 mg, 83%) as a light yellow oil.

$[\alpha]_D^{20}$ +159 (*c* 0.8 in $CHCl_3$); **IR** ν_{max} (film, cm^{-1}) 3447 (br, O-H), 2953 (C-H), 1740 (C=O); **HRMS** ($ASAP^+$) $C_{17}H_{17}F_3NO_3$ $[M+H]^+$ found 340.1161, requires 340.1155 (+1.8 ppm).

Data for major diastereoisomer: **Chiral HPLC analysis**, Chiralpak AD-H (95:5 hexane/*i*-PrOH, flow rate 1 $mLmin^{-1}$, 220 nm, 30 °C) t_R (major): 19.5 min, t_R (minor): 28.4 min, 98:2 er; **^{1}H NMR** (400 MHz, $CDCl_3$) δ_H: 2.26 (1H, dd, *J* 13.6, 2.7, C(7)H^AH^B), 2.32 (1H, d, *J* 1.8, O*H*), 3.06 (1H, app td, *J* 13.6, 1.9, C(7)H^AH^B), 3.40 (3H, s, OCH_3), 4.04 (1H, ddd, *J* 13.6, 6.0, 2.7, C(6)*H*), 4.99 (1H, d, *J* 6.0, C(5)*H*), 6.27 (1H, dd, *J* 3.8, 2.8, C(2)*H*), 6.48 (1H, dq, *J* 3.4, 1.7, C(1)*H*), 6.64 (1H, dd, *J* 2.8, 1.6, C(3)*H*), 7.25–7.27 (2H, m, PhC(2,6)*H*), 7.30–7.40 (3H, m, PhC(3,4,5)*H*); **^{13}C{^{1}H} NMR** (126 MHz, $CDCl_3$) δ_C: 28.8 (*C*(7)H_2), 37.9 (*C*(6)H), 52.2 (OCH_3), 62.3 (*C*(5)H), 70.7 (q, $^2J_{CF}$ 30.4, *C*(8)), 107.8 (*C*(1)H), 110.4 (*C*(2)H), 121.4 (*C*(3)H), 125.5 (q, $^1J_{CF}$ 284.2, CF_3), 125.9 (*C*(8a)), 127.7 (Ph*C*(2,6)H), 128.1 (Ph*C*(4)H), 128.9 (Ph*C*(3,5)H), 137.9 (Ph*C*(1)), 169.4 (*C*=O); **^{19}F{^{1}H} NMR** (377 MHz, $CDCl_3$) δ_F: −80.6 (CF_3).

Selected data for minor diastereoisomer: **Chiral HPLC analysis**, Chiralpak AD-H (95:5 hexane:*i*-PrOH, flow rate 1 $mLmin^{-1}$, 220 nm, 30 °C) t_R (major): 14.7 min, t_R (minor): 24.8 min, 96:4 er; **^{1}H NMR** (400 MHz, $CDCl_3$) δ_H: 3.64 (3H, s, OCH_3), 3.91 (1H, td, *J* 11.1, 4.8, C(6)*H*), 4.76 (1H, d, *J* 11.0, C(5)*H*), 6.60 (1H, dd, *J* 3.0, 1.5, C(1)*H*); **^{19}F{^{1}H} NMR** (377 MHz, $CDCl_3$) δ_F: −80.3 (CF_3).

((5*S*,6*S*,8*R*)-8-Hydroxy-6-phenyl-8-(trifluoromethyl)-5,6,7,8-tetrahydroindolizin-5-yl)(morpholino)methanone (16)

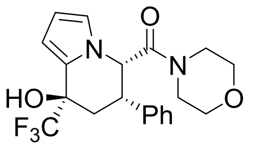

Following **General Procedure B**, 2-(1*H*-pyrrol-1-yl)acetic acid **1** (30 mg, 0.24 mmol, 1.2 eq.), *i*-Pr_2NEt (70 μL, 0.40 mmol), pivaloyl chloride (50 μL, 0.40 mmol) in *i*-PrOAc (2 mL) at 0 °C for 20 min followed by HyperBTM **11** (6.2 mg, 10 mol%), CF_3 enone **7** (40 mg, 0.20 mmol, 1.0 eq.) and *i*-Pr_2NEt (89 μL, 0.5 mmol) at –40 °C for 20 h. Ring-opening with morpholine (300 eq.) at rt for 24 h gave crude product (95:5 dr) that was purified by column chromatography (30:70 EtOAc/petrol) to give the title compound as a colourless solid (49 mg, 62%).

mp 174–175 °C; $[\alpha]_D^{20}$ +116 (*c* 3.0 in $CHCl_3$); **Chiral HPLC analysis**, Chiralcel OD-H (80:20 hexane/*i*-PrOH, flow rate 1 mLmin^{-1}, 211 nm, 30 °C) t_R (major): 18.5 min, t_R (minor): 26.5 min, 97:3 er; **IR** ν_{max} (film, cm^{-1}) 3391 (O-H), 2995 (C-H), 2873 (C-H), 1636 (amide C=O); **^{1}H NMR** (400 MHz, $CDCl_3$) δ_H: 2.12 (1H, dd, *J* 13.4, 2.8, C(7)H^AH^B), 2.29 (1H, d, *J* 1.8, O*H*), 2.46–2.54 (2H, m, morphC*H*), 3.14 (1H, ddd, *J* 13.0, 7.4, 3.0, morphC*H*), 3.27–3.40 (4H, m, morphC*H* + C(7)H^AH^B), 3.44–3.54 (2H, m, morphC*H*), 4.03 (1H, ddd, *J* 13.5, 5.9, 2.8, C(6)*H*), 5.33 (1H, d, *J* 5.9, C(5)*H*), 6.26 (1H, dd, *J* 3.8, 2.8, C(2)*H*), 6.46–6.50 (2H, m, C(1)*H* + C(3)*H*), 7.32–7.41 (5H, m, PhC*H*); **^{13}C{^{1}H} NMR** (126 MHz, $CDCl_3$) δ_C: 29.3 (*C*(7)H_2), 39.1 (*C*(6)H), 42.2 (morph*C*H_2), 46.0 (morph*C*H_2), 56.3 (*C*(5)H), 65.8 (morph*C*H_2), 66.4 (morph*C*H_2), 70.9 (q, $^2J_{CF}$ 30.2, *C*(8)), 107.3 (*C*(1)H), 110.4 (*C*(2)H), 120.5 (*C*(3)H), 125.6 (q, $^1J_{CF}$ 284.5, *C*F_3), 127.1 (*C*(8a)), 128.5 (Ph*C*(4)H), 128.7 (2 x Ph*C*H), 129.2 (2 x Ph*C*H), 138.4 (Ph*C*(1)), 166.8 (*C*=O); **^{19}F NMR** (471 MHz, $CDCl_3$) δ_F: −80.4 (C*F*$_3$); **HRMS** (ESI$^+$) $C_{20}H_{21}F_3N_2O_3Na$ $[M+Na]^+$ found 417.1386, requires 417.1396 (−2.4 ppm).

((5*S*,6*S*,8*R*)-8-Hydroxy-6-phenyl-8-(trifluoromethyl)-5,6,7,8-tetrahydroindolizin-5-yl)(pyrrolidin-1-yl)methanone (17)

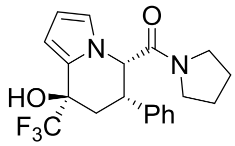

Following General Procedure **B**, 2-(1*H*-pyrrol-1-yl)acetic acid **1** (30 mg, 0.24 mmol, 1.2 eq.), *i*-Pr_2NEt (70 μL, 0.40 mmol), pivaloyl chloride (50 μL, 0.40 mmol) in *i*-PrOAc (2 mL) at 0 °C for 20 min followed by HyperBTM **11** (6.2 mg, 10 mol%), CF_3 enone **7** (40 mg, 0.20 mmol, 1.0 eq.) and *i*-Pr_2NEt (89 μL, 0.5 mmol) at –40 °C for 20 h. Ring-opening with pyrrolidine (300 eq.) at rt for 24 h gave crude product (93:7 dr) that was purified by column chromatography (30:70 EtOAc/petrol) to give the title compound as a colourless solid (53 mg, 70%).

mp 170–172 °C; $[\alpha]_D^{20}$ +175 (*c* 0.6 in $CHCl_3$); **Chiral HPLC analysis**, Chiralcel OD-H (80:20 hexane/*i*-PrOH, flow rate 1 $mLmin^{-1}$, 211 nm, 30 °C) t_R (major): 7.7 min, t_R (minor): 14.5 min, 98:2 er; **IR** ν_{max} (film, cm^{-1}) 3318 (O-H), 2982 (C-H), 1636 (amide CO); **^{1}H NMR** (500 MHz, $CDCl_3$) δ_H: 1.24–1.30 (1H, m, pyrrolidineC*H*), 1.46–1.52 (1H, m, pyrrolidineC*H*), 1.59–1.70 (2H, m, pyrrolidineC*H*), 2.09 (1H, dd, *J* 13.3, 2.7, C(7)H^AH^B), 2.14–2.19 (1H, m, pyrrolidineC*H*), 2.28 (1H, d, *J* 1.8, O*H*), 3.13–3.20 (2H, m, pyrrolidineC*H*), 3.33–3.39 (1H, m, pyrrolidineC*H*), 3.43 (1H, app td, *J* 13.4, 1.7, C(7)H^AH^B), 3.99 (1H, ddd, *J* 13.5, 5.8, 2.8, C(6)*H*), 5.10 (1H, d, *J* 5.8, C(5)*H*), 6.25 (1H, dd, *J* 3.8, 2.8, C(2)*H*), 6.46 (1H, dt, *J* 3.6, 1.7, C(1)*H*), 6.52 (1H, dd, *J* 2.8, 1.6, C(3)*H*), 7.29–7.35 (5H, m, PhC*H*); **^{13}C{^{1}H} NMR** (126 MHz, $CDCl_3$) δ_C: 23.9 (pyrrolidine*C*H_2), 26.1 (pyrrolidine*C*H_2), 29.5 (*C*(7)H_2), 39.4 (*C*(6)H), 45.9 (pyrrolidine*C*H_2), 46.4 (pyrrolidine*C*H_2), 59.6 (*C*(5)H), 71.0 (q, $^2J_{CF}$ 30.7, *C*(8)), 107.1 (app. d, *J* 2.7, *C*(1)H), 110.2 (*C*(2)H), 120.6 (*C*(3)H), 125.6 (q, $^1J_{CF}$ 284.8, *C*F_3), 126.9 (*C*(8a)), 128.2 (Ph*C*(4)H), 128.5 (2 x Ph*C*H), 128.8 (2 x Ph*C*H), 138.5 (Ph*C*(1)), 166.4 (*C*=O); **^{19}F NMR** (471 MHz, $CDCl_3$) δ_F: −80.4 (C*F*$_3$); **HRMS** (ESI^+) $C_{20}H_{21}F_3N_2O_2Na$ $[M+Na]^+$ found 401.1436, requires 401.1447 (−2.7 ppm).

Methyl (5*S*,6*S*,8*R*)-6-(4-bromophenyl)-8-hydroxy-8-(trifluoromethyl)-5,6,7,8-tetrahydroindolizine-5-carboxylate (18)

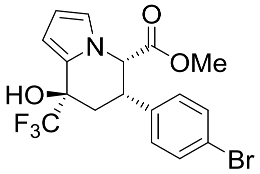

Following **General Procedure B**, 2-(1*H*-pyrrol-1-yl)acetic acid **1** (30 mg, 0.24 mmol, 1.2 eq.), *i*-Pr_2NEt (70 μL, 0.40 mmol), pivaloyl chloride (50 μL, 0.40 mmol) in *i*-PrOAc (2 mL) at 0 °C for 20 min followed by HyperBTM **11** (6.2 mg, 10 mol%), (*E*)-4-(4-bromophenyl)-1,1,1-trifluorobut-3-en-2-one **S2** (56 mg, 0.20 mmol, 1.0 eq.) and *i*-Pr_2NEt (89 μL, 0.5 mmol) at –40 °C for 20 h. Ring-opening with MeOH (2 mL) and DMAP (4.9 mg, 20 mol%) at rt for 24 h gave crude product (90:10 dr) that was purified by column chromatography (15:85 to 20:80 EtOAc/petrol) to give the title compound as a light yellow oil (52 mg, 62%).

$[\alpha]_D^{20}$ +117 (*c* 1.0 in $CHCl_3$); **Chiral HPLC analysis**, Chiralpak AD-H (97.5: 2.5 hexane/*i*-PrOH, flow rate 1 $mLmin^{-1}$, 220 nm, 30 °C) t_R (major): 52.0 min, t_R (minor): 73.7 min, 98:2 er; **IR** ν_{max} (film, cm^{-1}) 3433 (O-H stretch), 2953 (C-H stretch), 1740 (C=O), 1157; **^{1}H NMR** (400 MHz, $CDCl_3$) δ_H: 2.23 (1H, dd, *J* 13.4, 2.7, C(7)H^AH^B), 2.30 (1H, d, *J* 1.8, O*H*), 3.01 (1H, app td, *J* 13.5, 1.9, C(7)H^AH^B), 3.44 (3H, s, OC*H*$_3$), 4.00 (1H, ddd, *J* 13.5, 6.0, 2.7, C(6)*H*), 4.96 (1H, d, *J* 6.0, C(5)*H*), 6.27 (1H, dd, *J* 3.7, 2.9, C(2)*H*), 6.48 (1H, dt, *J* 3.7, 1.7, C(1)*H*), 6.64 (1H, dd, *J* 2.9, 1.6, C(3)*H*), 7.12–7.16 (2H, m, C(6)ArC(2,6)*H*), 7.49–7.52 (2H, m, C(6)ArC(3,5)*H*); **^{13}C{^{1}H} NMR** (126 MHz, $CDCl_3$) δ_C: 28.8 (*C*(7)H_2), 37.5 (*C*(6)H), 52.4 (O*C*H_3), 62.0 (C(5)H), 70.6 (q, $^2J_{CF}$ 30.6, *C*(8)), 108.0 (app. d, *J* 2.7, *C*(1)H), 110.5 (*C*(2)H), 121.4 (*C*(3)H), 122.1

(C(6)Ar*C*(4)), 125.4 (q, $^{1}J_{CF}$ 284.1, *C*F_3), 125.8 (*C*(8a)), 129.5 (C(6)Ar*C*(2,6)H), 132.1 (C(6)Ar*C*(3,5)H), 137.0 (C(6)Ar*C*(1)), 169.1 (*C*=O); **^{19}F NMR** (471 MHz, $CDCl_3$) δ_F: −80.6 (C*F*$_3$); **HRMS** (ASAP$^+$) $C_{17}H_{16}F_3NO_3{}^{79}Br$ [M+H]$^+$ found 418.0266, requires 418.0260 (+1.4 ppm).

Methyl (5*S*,6*S*,8*R*)-8-hydroxy-8-(trifluoromethyl)-6-(4-(trifluoromethyl)phenyl)-5,6,7,8-tetrahydroindolizine-5-carboxylate (19)

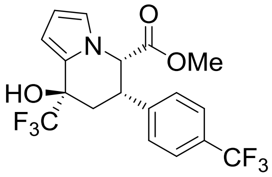

Following **General Procedure B**, 2-(1*H*-pyrrol-1-yl)acetic acid **1** (30 mg, 0.24 mmol, 1.2 eq.), *i*-Pr_2NEt (70 μL, 0.40 mmol), pivaloyl chloride (50 μL, 0.40 mmol) in *i*-PrOAc (2 mL) at 0 °C for 20 min followed by HyperBTM **11** (6.2 mg, 10 mol%), (*E*)-1,1,1-trifluoro-4-(4-(trifluoromethyl)phenyl)but-3-en-2-one **S3** (54 mg, 0.20 mmol, 1.0 eq.) and *i*-Pr_2NEt (89 μL, 0.5 mmol) at –40 °C for 20 h. Ring-opening with MeOH (2 mL) and DMAP (4.9 mg, 20 mol%) at rt for 24 h gave crude product that was purified by column chromatography (15:85 EtOAc/petrol) to give the title compound as a light pink oil (33 mg, 40%).

$[\alpha]_D^{20}$ +129 (*c* 0.5 in $CHCl_3$); **Chiral HPLC analysis**, Chiralpak AD-H (97.5:2.5 hexane/IPA, flow rate 1 mLmin^{-1}, 211 nm, 30 °C) t_R(major): 45.6 min, t_R(minor): 62.9 min, 97:3 er; **IR** ν_{max}(film, cm^{-1}) 3472 (O-H stretch), 2955 (C-H stretch), 1742 (C=O), 1325, 1161; **^{1}H NMR** (400 MHz, $CDCl_3$) δ_H: 2.30 (1H, dd, *J* 13.5, 2.7, C(7)H^AH^B), 2.37 (1H, d, *J* 1.9, O*H*), 3.09 (1H, app td, *J* 13.5, 1.9, C(7)H^AH^B), 3.44 (3H, s, OCH_3), 4.14 (1H, ddd, *J* 13.5, 5.9, 2.7, C(6)*H*), 5.03 (1H, d, *J* 5.9, C(5)*H*), 6.31 (1H, dd, *J* 3.8, 2.8, C(2)*H*), 6.52 (1H, dp, *J* 3.7, 1.7, C(1)*H*), 6.68 (1H, dd, *J* 2.8, 1.6, C(3)*H*), 7.42 (2H, d, *J* 8.4, C(6)ArC(2,6)*H*), 7.67 (2H, d, *J* 8.1, C(6)ArC(3,5)*H*); **^{13}C{^{1}H} NMR** (126 MHz, $CDCl_3$) δ_C: 28.7 (*C*(7)H_2), 37.9 (*C*(6)H), 52.4 (OCH_3), 61.9 (*C*(5)H), 70.6 (q, $^{2}J_{CF}$ 30.8, *C*(8)), 108.0 (*C*(1)H), 110.6 (*C*(2)H), 121.5 (*C*(3)H), 124.0 (q, $^{1}J_{CF}$ 272.1, C(6)ArC(4)*C*F_3), 125.4 (q, $^{1}J_{CF}$ 284.1, C(8)*C*F_3), 125.7 (*C*(8a)), 125.9 (q, $^{3}J_{CF}$ 3.8, C(6)Ar*C*(3,5)H), 128.3 (C(6)Ar*C*(2,6)H), 130.4 (q, $^{2}J_{CF}$ 32.6, C(6)Ar*C*(4)), 142.0 (C(6)Ar*C*(1)), 169.0 (*C*=O); **^{19}F NMR** (471 MHz, $CDCl_3$) δ_F: −80.6 (C(8)C*F*$_3$), −62.6 (ArC*F*$_3$); HRMS (ESI$^+$) $C_{18}H_{15}F_6NO_3Na$ [M+Na]$^+$ found 430.0837, requires 430.0848 (−2.6 ppm).

Methyl (5*S*,6*S*,8*R*)-8-hydroxy-6-(3-methoxyphenyl)-8-(trifluoromethyl)-5,6,7,8-tetrahydroindolizine-5-carboxylate (20)

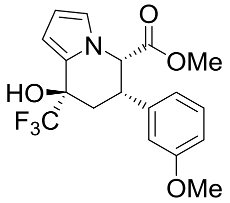

Following **General Procedure B**, 2-(1*H*-pyrrol-1-yl)acetic acid **1** (30 mg, 0.24 mmol, 1.2 eq.), *i*-Pr_2NEt (70 μL, 0.40 mmol), pivaloyl chloride (50 μL, 0.40 mmol) in *i*-PrOAc (2 mL) at 0 °C for 20 min followed by HyperBTM **11** (6.2 mg, 10 mol%), (*E*)-1,1,1-trifluoro-4-(3-methoxyphenyl)but-3-en-2-one **S4** (46 mg, 0.20 mmol, 1.0 eq.) and *i*-Pr_2NEt (89 μL, 0.5 mmol) at –40 °C for 20 h. Ring-opening with MeOH (2 mL) and DMAP (4.9 mg, 20 mol%) at rt for 24 h gave crude product (87:13 dr) that was purified by column chromatography (15:85 EtOAc/petrol) to give the title compound as a light yellow oil (40 mg, 54%).

$[\alpha]_D^{20}$ +153 (*c* 0.3 in $CHCl_3$); **Chiral HPLC analysis**, Chiralpak IB (85:15 hexane/IPA, flow rate 1 mLmin^{-1}, 211 nm, 30 °C) t_R(major): 9.0 min, t_R(minor): 23.6 min, 96:4 er; **IR** ν_{max}(film, cm^{-1}) 3447 (O-H stretch), 2953 (C-H stretch), 2837 (C-H stretch), 1744 (C=O), 1165; **^{1}H NMR** (400 MHz, $CDCl_3$) δ_H: 2.25 (1H, dd, *J* 13.6, 2.7, C(7)H^AH^B), 2.31 (1H, s, O*H*), 3.03 (1H, app td, *J* 13.6, 1.9, C(7)H^AH^B), 3.43 (3H, s, OCH_3), 3.82 (3H, s, OCH_3), 4.00 (1H, ddd, *J* 13.5, 6.0, 2.7, C(6)*H*), 4.98 (1H, d, *J* 6.0, C(5)*H*), 6.26 (1H, dd, *J* 3.8, 2.8, C(2)*H*), 6.48 (1H, app dt, *J* 3.7, 1.7, C(1)*H*), 6.63 (1H, dd, *J* 2.9, 1.6, C(3)*H*), 6.79 (1H, app t, *J* 2.1, C(6)ArC(2)*H*), 6.83–6.87 (2H, m, C(6)ArC(4,6)*H*), 7.29 (1H, app t, *J* 7.9, C(6)ArC(5)*H*); **^{13}C{^{1}H} NMR** (126 MHz, $CDCl_3$) δ_C: 28.9 (*C*(7)H_2), 38.0 (*C*(6)H), 52.3 (OCH_3), 55.4 (OCH_3), 62.3 (*C*(5)H), 70.7 (q, $^2J_{CF}$ 30.4, *C*(8)), 107.8 (app d, *J* 2.8, *C*(1)H), 110.4 (*C*(2)H), 113.2 (C(6)Ar*C*(6)H), 113.9 (C(6)Ar*C*(2)H), 119.9 (C(6)Ar*C*(4)H), 121.4 (*C*(3)H), 125.5 (q, $^1J_{CF}$ 284.0, CF_3), 125.9 (*C*(8a)), 129.9 (C(6)Ar*C*(5)H), 139.5 (C(6)Ar*C*(1)), 160.0 (C(6)Ar*C*(3)), 169.4 (*C*=O); **^{19}F NMR** (471 MHz, $CDCl_3$) δ_F: –80.6 (CF_3); **HRMS** (ASAP$^+$) $C_{18}H_{19}F_3NO_4$ $[M+H]^+$ found 370.1261, requires 370.1261 (+0.0 ppm).

Methyl (5*S*,6*S*,8*R*)-8-hydroxy-6-(4-methoxyphenyl)-8-(trifluoromethyl)-5,6,7,8-tetrahydroindolizine-5-carboxylate (21)

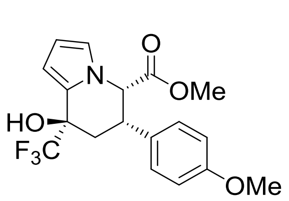

Following **General Procedure B**, 2-(1*H*-pyrrol-1-yl)acetic acid **1** (30 mg, 0.24 mmol, 1.2 eq.), *i*-Pr_2NEt (70 μL, 0.40 mmol), pivaloyl chloride (50 μL, 0.40 mmol) in *i*-PrOAc (2 mL) at 0 °C for 20 min followed by HyperBTM **11** (6.2 mg, 10 mol%), (*E*)-1,1,1-trifluoro-4-(4-methoxyphenyl)but-3-en-2-one **S6** (46

mg, 0.20 mmol, 1.0 eq.) and *i*-Pr_2NEt (89 μL, 0.5 mmol) at –40 °C for 20 h. Ring-opening with MeOH (2 mL) and DMAP (4.9 mg, 20 mol%) at rt for 24 h gave crude product (84:16 dr) that was purified by column chromatography (15:85 EtOAc/petrol) to give the title compound as a light orange solid (50 mg, 68%).

mp 114–116 °C; $[\alpha]_D^{20}$ +167 (*c* 0.5 in $CHCl_3$); **Chiral HPLC analysis**, Chiralpak AD-H (97.5:2.5 hexane/IPA, flow rate 1 $mLmin^{-1}$, 211 nm, 30 °C) t_R (major): 59.1 min, t_R (minor): 104.6 min, 99:1 er; **IR** ν_{max} (film, cm^{-1}) 3460 (O-H stretch), 2953 (C-H stretch), 2814 (C-H stretch), 1744 (C=O), 1514, 1259, 1160; **^{1}H NMR** (500 MHz, $CDCl_3$) δ_H: 2.22 (1H, dd, *J* 13.6, 2.7, C(7)H^AH^B), 2.33 (1H, d, *J* 1.8, O*H*), 3.02 (1H, app td, *J* 13.6, 1.9, C(7)H^AH^B), 3.43 (3H, s, OCH_3), 3.82 (3H, s, OCH_3), 3.98 (1H, ddd, *J* 13.6, 6.0, 2.7, C(6)*H*), 4.95 (1H, d, *J* 6.0, C(5)*H*), 6.26 (1H, dd, *J* 3.8, 2.8, C(2)*H*), 6.48 (1H, dt, *J* 3.6, 1.7, C(1)*H*), 6.63 (1H, dd, *J* 2.8, 1.6, C(3)*H*), 6.88–6.91 (2H, m, C(6)ArC(3,5)*H*), 7.15–7.18 (2H, m, C(6)ArC(2,6)*H*); **^{13}C{^{1}H} NMR** (126 MHz, $CDCl_3$) δ_C: 29.1 (*C*(7)H_2), 37.1 (*C*(6)H), 52.3 (O*C*H_3), 55.4 (O*C*H_3), 62.4 (*C*(5)H), 70.7 (q, $^2J_{CF}$ 30.3, *C*(8)), 107.8 (app. d, *J* 2.7, *C*(1)H), 110.3 (*C*(2)H), 114.2 (C(6)Ar*C*(3,5)H), 121.3 (*C*(3)H), 125.5 (q, $^1J_{CF}$ 284.0, *C*F_3), 125.8 (*C*(8a)), 128.7 (C(6)Ar*C*(2,6)H), 129.9 (C(6)Ar*C*(1)), 159.3 (C(6)Ar*C*(4)), 169.5 (*C*=O); **^{19}F NMR** (471 MHz, $CDCl_3$) δ_F: –80.6 (*C*F_3); **HRMS** (NSI$^+$) $C_{18}H_{19}F_3NO_4$ $[M+H]^+$ found 370.1262, requires 370.1261 (+0.4 ppm).

Selected data for the minor diastereoisomer: **Chiral HPLC analysis**, Chiralpak AD-H (97.5:2.5 hexane/IPA, flow rate 1 $mLmin^{-1}$, 211 nm, 30 °C) t_R (major): 37.2 min, t_R (minor): 95.3 min, 95:5 er; **^{1}H NMR** (500 MHz, $CDCl_3$) δ_H: 2.27–2.36 (2H, m, C(7)H_2), 2.56 (1H, s, O*H*), 3.64 (3H, s, OCH_3), 3.80–3.86 (4H, m, OCH_3 + C(6)*H*), 4.68 (1H, d, *J* 11.1, C(5)*H*), 6.25 (1H, dd, *J* 3.8, 2.9, C(2)*H*), 6.46 (1H, dt, *J* 3.7, 1.7, C(1)*H*), 6.57 (1H, dd, *J* 2.9, 1.6, C(3)*H*), 6.87–6.90 (2H, m, C(6)ArC(3,5)*H*), 7.17–7.20 (2H, m, C(6)ArC(2,6)*H*); **^{13}C{^{1}H} NMR** (126 MHz, $CDCl_3$) δ_C: 35.1 (C(7)H_2), 39.0 (*C*(6)H), 52.8 (O*C*H_3), 55.4 (O*C*H_3), 64.9 (*C*(5)H), 70.3 (q, $^2J_{CF}$ 30.4, *C*(8)), 107.8 (*C*(1)H), 110.1 (*C*(2)H), 114.5 (C(6)Ar*C*(3,5)H), 120.9 (C(3)H), 125.4 (q, $^1J_{CF}$ 284.2, *C*F_3), 125.4 (*C*(8a)), 128.8 (C(6)Ar*C*(2,6)H), 130.8 (C(6)Ar*C*(1)), 159.3 (C(6)Ar*C*(4)), 170.5 (*C*=O); **^{19}F NMR** (471 MHz, $CDCl_3$) δ_F: –80.3 (C*F*$_3$).

Methyl (5*S*,6*S*,8*R*)-8-hydroxy-6-(*p*-tolyl)-8-(trifluoromethyl)-5,6,7,8-tetrahydroindolizine -5-carboxylate (22)

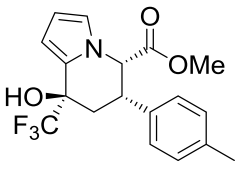

Following **General Procedure B**, 2-(1*H*-pyrrol-1-yl)acetic acid **1** (30 mg, 0.24 mmol, 1.2 eq.), *i*-Pr_2NEt (70 μL, 0.40 mmol), pivaloyl chloride (50 μL, 0.40 mmol) in *i*-PrOAc (2 mL) at 0 °C for 20 min followed by HyperBTM **11** (6.2 mg, 10 mol%), (*E*)-1,1,1-trifluoro-4-(*p*-tolyl)but-3-en-2-one **S5** (43 mg, 0.20 mmol, 1.0 eq.) and *i*-Pr_2NEt (89 μL, 0.5 mmol) at –40 °C for 20 h. Ring-opening with MeOH (2 mL) and

DMAP (4.9 mg, 20 mol%) at rt for 24 h gave crude product (80:20 dr) that was purified by column chromatography (15:85 EtOAc/petrol) to give the title compound as a light pink oil (41 mg, 58%).

$[\alpha]_D^{20}$ +132 (*c* 0.4 in $CHCl_3$); **Chiral HPLC analysis**, Chiralpak AD-H (97.5: 2.5 hexane/*i*-PrOH, flow rate 1 mLmin^{-1}, 220 nm, 30 °C) t_R (major): 38.7 min, t_R (minor): 59.7 min, 98:2 er; **IR** ν_{max} (film, cm^{-1}) 3458 (O-H stretch), 2953 (C-H stretch), 1744 (C=O), 1161; **^{1}H NMR** (400 MHz, $CDCl_3$) δ_H: 2.23 (1H, dd, *J* 13.6, 2.8, C(7)H^AH^B), 2.28–2.29 (1H, m, O*H*), 2.35 (3H, s, CH_3), 3.04 (1H, app td, *J* 13.6, 1.9, C(7)H^AH^B), 3.42 (3H, s, OCH_3), 3.99 (1H, ddd, *J* 13.6, 6.0, 2.8, C(6)*H*), 4.97 (1H, d, *J* 6.0, C(5)*H*), 6.26 (1H, dd, *J* 3.8, 2.8, C(2)*H*), 6.48 (1H, app dt, *J* 3.6, 1.7, C(1)*H*), 6.63 (1H, dd, *J* 2.9, 1.6, C(3)*H*), 7.12–7.18 (4H, m, ArC*H*); **^{13}C{^{1}H} NMR** (126 MHz, $CDCl_3$) δ_C: 21.3 (CH_3), 29.0 (*C*(7)H_2), 37.6 (*C*(6)H), 52.2 (OCH_3), 62.4 (*C*(5)H), 70.7 (q, $^2J_{CF}$ 30.5, *C*(8)), 107.8 (*C*(1)H), 110.4 (*C*(2)H), 121.4 (*C*(3)H), 125.5 (q, $^1J_{CF}$ 284.1, CF_3), 126.0 (*C*(8a)), 127.6 (C(6)Ar*C*(3,5)H), 129.6 (C(6)Ar*C*(2,6)H), 134.9 (C(6)Ar*C*(4)), 137.8 (C(6)Ar*C*(1)), 169.4 (*C*=O); **^{19}F NMR** (471 MHz, $CDCl_3$) δ_F: −80.6 (CF_3); **HRMS** (ESI$^+$) $C_{18}H_{18}F_3NO_3Na$ $[M+Na]^+$ found 376.1129, requires 376.1131 (−0.5 ppm).

Methyl (5*S*,6*S*,8*R*)-8-hydroxy-6-(*o*-tolyl)-8-(trifluoromethyl)-5,6,7,8-tetrahydroindolizine-5-carboxylate (23)

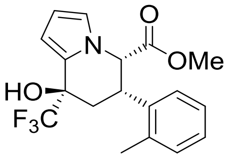

Following **General Procedure B**, 2-(1*H*-pyrrol-1-yl)acetic acid **1** (30 mg, 0.24 mmol, 1.2 eq.), *i*-Pr_2NEt (70 μL, 0.40 mmol), pivaloyl chloride (50 μL, 0.40 mmol) in *i*-PrOAc (2 mL) at 0 °C for 20 min followed by HyperBTM **11** (6.2 mg, 10 mol%), (*E*)-1,1,1-trifluoro-4-(*o*-tolyl)but-3-en-2-one **S7** (43 mg, 0.20 mmol, 1.0 eq.) and *i*-Pr_2NEt (89 μL, 0.5 mmol) at –40 °C for 20 h. Ring-opening with MeOH (2 mL) and DMAP (4.9 mg, 20 mol%) at rt for 24 h gave crude product that was purified by column chromatography (15:85 EtOAc/petrol) to give the title compound as a colourless solid (18 mg, 25%).

mp 154–156 °C; $[\alpha]_D^{20}$ +180 (*c* 0.3 in $CHCl_3$); **Chiral HPLC analysis**, Chiralpak AD-H (97.5:2.5 hexane : IPA, flow rate 1 mLmin^{-1}, 211 nm, 30 °C) t_R (major): 17.6 min, t_R (minor): 77.3 min, > 99:1 er; **IR** ν_{max} (film, cm^{-1}) 3300 (O-H stretch), 2953 (C-H stretch), 1746 (C=O), 1152; **^{1}H NMR** (400 MHz, $CDCl_3$) δ_H: 2.18 (1H, dd, *J* 13.5, 2.6, C(7)H^AH^B), 2.31 (1H, d, *J* 1.9, O*H*), 2.47 (3H, s, CH_3), 3.10 (1H, app td, *J* 13.4, 1.8, C(7)H^AH^B), 3.37 (3H, s, OCH_3), 4.23 (1H, ddd, *J* 13.4, 6.0, 2.6, C(6)*H*), 4.98 (1H, d, *J* 6.0, C(5)*H*), 6.28 (1H, dd, *J* 3.8, 2.8, C(2)*H*), 6.49 (1H, dt, *J* 3.6, 1.7, C(1)*H*), 6.63 (1H, dd, *J* 2.8, 1.6, C(3)*H*), 7.06–7.09 (1H, m, C(6)ArC*H*), 7.16–7.25 (3H, m, C(6)ArC*H*); **^{13}C{^{1}H} NMR** (126 MHz, $CDCl_3$) δ_C: 19.5 (CH_3), 29.3 (*C*(7)H_2), 34.0 (*C*(6)H), 52.2 (OCH_3), 60.1 (*C*(5)H), 70.8 (q, $^2J_{CF}$ 30.3, *C*(8)), 107.8 (*C*(1)H), 110.4 (*C*(2)H), 121.4 (*C*(3)H), 125.5 (q, $^1J_{CF}$ 284.2, CF_3), 126.0 (*C*(8a)), 126.1 (C(6)ArC*H*), 126.5 (C(6)ArC*H*), 127.9 (C(6)ArC*H*), 131.1 (C(6)ArC*H*), 136.0 (C(6)Ar*C*(2)), 136.4 (C(6)Ar*C*(1)), 169.5 (*C*=O);

^{19}**F NMR** (471 MHz, $CDCl_3$) δ_F: −80.6 (CF_3); **HRMS** (ASAP$^+$) $C_{18}H_{19}F_3NO_3$ $[M+H]^+$ found 354.1315, requires 354.1312 (+0.8 ppm).

Methyl (5*S*,6*S*,8*R*)-8-hydroxy-6-(naphthalen-1-yl)-8-(trifluoromethyl)-5,6,7,8-tetrahydroindolizine-5-carboxylate (24)

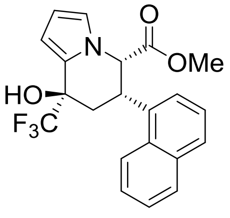

Following **General Procedure B**, 2-(1*H*-pyrrol-1-yl)acetic acid **1** (30 mg, 0.24 mmol, 1.2 eq.), *i*-Pr_2NEt (70 μL, 0.40 mmol), pivaloyl chloride (50 μL, 0.40 mmol) in *i*-PrOAc (2 mL) at 0 °C for 20 min followed by HyperBTM **11** (6.2 mg, 10 mol%), (*E*)-1,1,1-trifluoro-4-(naphthalen-1-yl)but-3-en-2-one **S8** (50 mg, 0.20 mmol, 1.0 eq.) and *i*-Pr_2NEt (89 μL, 0.5 mmol) at –40 °C for 20 h. Ring-opening with MeOH (2 mL) and DMAP (4.9 mg, 20 mol%) at rt for 24 h gave crude product (80:20 dr) that was purified by column chromatography (15:85 EtOAc/petrol) to give the title compound as a colourless solid (60 mg, 77%).

mp 138–140 °C; $[\alpha]_D^{20}$ +193 (*c* 2.9 in $CHCl_3$); **Chiral HPLC analysis**, Chiralpak AD-H (97.5:2.5 hexane/IPA, flow rate 1 mLmin^{-1}, 211 nm, 30 °C) t_R (major): 52.9 min, t_R (minor): 45.2 min, 98:2 er; **IR** ν_{max} (film, cm^{-1}) 3466 (O-H stretch), 2961 (C-H stretch), 1744 (C=O), 1163; ^{1}**H NMR** (400 MHz, $CDCl_3$) δ_H: 2.35 (1H, dd, *J* 13.3, 2.5, C(7)H^AH^B), 2.39 (1H, d, *J* 1.8, O*H*), 3.24–3.31 (4H, m, C(7)H^AH^B + OCH_3), 4.88 (1H, ddd, *J* 13.3, 5.9, 2.5, C(6)*H*), 5.26 (1H, d, *J* 5.8, C(5)*H*), 6.30 (1H, dd, *J* 3.8, 2.8, C(2)*H*), 6.54 (1H, dt, *J* 3.7, 1.7, C(1)*H*), 6.64 (1H, dd, *J* 2.8, 1.6, C(3)*H*), 7.32 (1H, d, *J* 7.2, C(6)ArC*H*), 7.45 (1H, dd, *J* 8.2, 7.2, C(6)ArC*H*), 7.56 (1H, ddd, *J* 8.0, 6.8, 1.2, C(6)ArC*H*), 7.62 (1H, ddd, *J* 8.4, 6.8, 1.5, C(6)ArC*H*), 7.84 (1H, d, *J* 8.2, C(6)ArC*H*), 7.92–7.95 (1H, m, C(6)ArC*H*), 8.17 (1H, d, *J* 8.4, C(6)ArC*H*); ^{13}**C{^{1}H} NMR** (126 MHz, $CDCl_3$) δ_C: 29.4 (*C*(7)H_2), 33.3 (*C*(6)H), 52.0 (OCH_3), 61.0 (*C*(5)H), 71.0 (q, $^2J_{CF}$ 30.2, *C*(8)), 107.9 (app d, *J* 2.9, *C*(1)H), 110.5 (*C*(2)H), 121.5 (*C*(3)H), 122.3 (C(6)Ar*C*H), 123.9 (C(6)Ar*C*H), 125.3 (C(6)Ar*C*H), 125.6 (q, $^1J_{CF}$ 283.9, CF_3), 126.0 (*C*(8a)), 126.1 (C(6)Ar*C*H), 127.0 (C(6)Ar*C*H), 128.8 (C(6)Ar*C*H), 129.5 (C(6)Ar*C*H), 131.6 (C(6)Ar*C*), 133.9 (C(6)Ar*C*), 134.1 (C(6)Ar*C*(1)), 169.3 (*C*=O); ^{19}**F NMR** (471 MHz, $CDCl_3$) δ_F: −80.5 (CF_3); **HRMS** (NSI$^+$) $C_{21}H_{19}F_3NO_3$ $[M+H]^+$ found 390.1302, requires 390.1312 (−2.6 ppm).

Methyl (5*S*,6*S*,8*R*)-8-hydroxy-6-(naphthalen-2-yl)-8-(trifluoromethyl)-5,6,7,8-tetrahydroindolizine-5-carboxylate (25)

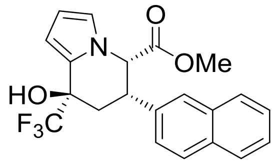

Following **General Procedure B**, 2-(1*H*-pyrrol-1-yl)acetic acid **1** (30 mg, 0.24 mmol, 1.2 eq.), *i*-Pr_2NEt (70 μL, 0.40 mmol), pivaloyl chloride (50 μL, 0.40 mmol) in *i*-PrOAc (2 mL) at 0 °C for 20 min followed by HyperBTM **11** (6.2 mg, 10 mol%), (*E*)-1,1,1-trifluoro-4-(naphthalen-2-yl)but-3-en-2-one **S9** (50 mg, 0.20 mmol, 1.0 eq.) and *i*-Pr_2NEt (89 μL, 0.5 mmol) at –40 °C for 20 h. Ring-opening with MeOH (2 mL) and DMAP (4.9 mg, 20 mol%) at rt for 24 h gave crude product (85:15 dr) that was purified by column chromatography (20:80 EtOAc/petrol) to give the title compound as a light yellow oil (65 mg, 83%).

$[\alpha]_D^{20}$ +119 (*c* 2.7 in $CHCl_3$); **Chiral HPLC analysis**, Chiralpak AD-H (97.5:2.5 hexane/IPA, flow rate 1 mLmin^{-1}, 254 nm, 30 °C) t_R (major): 69.1 min, t_R (minor): 91.9 min, 98:2 er; **IR** ν_{max} (film, cm^{-1}) 3472 (O-H stretch), 3055 (C-H stretch), 2903 (C-H stretch), 1742 (C=O), 1171; **^{1}H NMR** (500 MHz, $CDCl_3$) δ_H: 2.37 (1H, dd, *J* 13.5, 2.7, C(7)H^AH^B), 2.45 (1H, s, O*H*), 3.20 (1H, t, *J* 13.5, C(7)H^AH^B), 3.31 (3H, s, OCH_3), 4.20 (1H, ddd, *J* 13.5, 6.0, 2.7, C(6)*H*), 5.10 (1H, d, *J* 6.0, C(5)*H*), 6.30 (1H, dd, *J* 3.8, 2.8, C(2)*H*), 6.52 (1H, dt, *J* 3.6, 1.7, C(1)*H*), 6.67 (1H, dd, *J* 2.8, 1.6, C(3)*H*), 7.39 (1H, dd, *J* 8.5, 1.9, C(6)ArC*H*), 7.50–7.53 (2H, m, C(6)ArC*H*), 7.70 (1H, app. s, C(6)ArC*H*), 7.82–7.87 (3H, m, C(6)ArC*H*); **^{13}C{^{1}H} NMR** (126 MHz, $CDCl_3$) δ_C: 29.0 (*C*(7)H_2), 38.1 (*C*(6)H), 52.2 (OCH_3), 62.2 (*C*(5)H), 70.8 (q, $^2J_{CF}$ 30.5, *C*(8)), 107.9 (app. d, *J* 2.5, *C*(1)H), 110.4 (*C*(2)H), 121.4 (*C*(3)H), 125.5 (q, $^1J_{CF}$ 280.9, CF_3), 125.9 (*C*(8a)), 125.9 (C(6)Ar*C*H), 126.4 (C(6)Ar*C*H), 126.4 (C(6)Ar*C*H), 126.6 (C(6)Ar*C*H), 127.8 (C(6)Ar*C*H), 128.0 (C(6)Ar*C*H), 128.6 (C(6)Ar*C*H), 133.0 (C(6)Ar*C*), 133.5 (C(6)Ar*C*), 135.4 (C(6)Ar*C*(1)), 169.4 (*C*=O); **^{19}F NMR** (471 MHz, $CDCl_3$) δ_F: −80.5 (CF_3); **HRMS** (ASAP$^+$) $C_{21}H_{19}F_3NO_3$ $[M+H]^+$ found 390.1314, requires 390.1312 (+0.5 ppm).

Selected data for minor diastereoisomer: **Chiral HPLC analysis**, Chiralpak AD-H (97.5:2.5 hexane:IPA, flow rate 1 mLmin^{-1}, 211 nm, 30 °C) t_R (major): 40.5 min, t_R (minor): 75.5 min, 98:2 er; **^{1}H NMR** (500 MHz, $CDCl_3$) δ_H: 2.43–2.49 (2H, m, C(7)H_2), 2.62 (1H, s, O*H*), 3.58 (3H, s, OCH_3), 4.07 (1H, app td, *J* 10.7, 5.8, C(6)*H*), 4.87 (1H, d, *J* 11.0, C(5)*H*), 6.28 (1H, dd, *J* 3.9, 2.9, C(2)*H*), 6.50 (1H, dd, *J* 3.7, 1.8, C(1)*H*), 6.61 (1H, dd, *J* 2.9, 1.6, C(3)*H*), 7.39 (1H, dd, *J* 8.5, 1.9, C(6)ArC*H*), 7.49–7.53 (2H, m, C(6)ArC*H*), 7.75 (1H, d, *J* 1.8, C(6)ArC*H*), 7.81–7.87 (3H, m, C(6)ArC*H*); **^{13}C{^{1}H} NMR** (126 MHz, $CDCl_3$) δ_C (selected): 35.0 (*C*(7)H_2), 39.9 (*C*(6)H), 52.9 (OCH_3), 64.4 (*C*(5)H), 70.3 (d, $^2J_{CF}$ 30.5, *C*(8)), 107.9 (*C*(1)H), 110.1 (*C*(2)H), 121.0 (*C*(3)H), 125.5 (*C*(8a)), 126.4 (C(6)Ar*C*H), 126.6 (C(6)Ar*C*H), 127.0 (C(6)Ar*C*H), 127.9 (C(6)Ar*C*H), 128.0 (C(6)Ar*C*H), 129.1 (C(6)Ar*C*H), 133.1 (C(6)Ar*C*), 133.6 (C(6)Ar*C*), 136.3 (C(6)Ar*C*(1)), 170.5 (*C*=O); **^{19}F NMR** (471 MHz, $CDCl_3$) δ_F: −80.2 (CF_3).

Methyl (5*S*,6*R*,8*R*)-6-(furan-2-yl)-8-hydroxy-8-(trifluoromethyl)-5,6,7,8-tetrahydroindolizine -5-carboxylate (26)

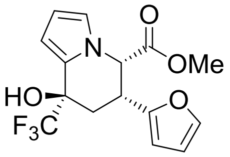

Following **General Procedure B**, 2-(1*H*-pyrrol-1-yl)acetic acid **1** (30 mg, 0.24 mmol, 1.2 eq.), *i*-Pr_2NEt (70 μL, 0.40 mmol), pivaloyl chloride (50 μL, 0.40 mmol) in *i*-PrOAc (2 mL) at 0 °C for 20 min followed by HyperBTM **11** (6.2 mg, 10 mol%), (*E*)-1,1,1-trifluoro-4-(furan-2-yl)but-3-en-2-one **S10** (38 mg, 0.20 mmol, 1.0 eq.) and *i*-Pr_2NEt (89 μL, 0.5 mmol) at –40 °C for 20 h. Ring-opening with MeOH (2 mL) and DMAP (4.9 mg, 20 mol%) at rt for 24 h gave crude product (77:23 dr) that was purified by column chromatography (20:80 EtOAc/petrol) to give the combined diastereoisomers (76:24 dr) as a light yellow oil (62 mg, 95%).

$[\alpha]_D^{20}$ +81.5 (*c* 2.2 in $CHCl_3$); **IR** ν_{max} (film, cm^{-1}) 3456 (O-H stretch), 2957 (C-H stretch), 2361, 1746 (C=O), 1277, 1163; **HRMS** (ESI^+) $C_{15}H_{14}F_3NO_4Na$ $[M+Na]^+$ found 352.0763, requires 352.0767 (−1.1 ppm).

Data for major diastereoisomer: **Chiral HPLC analysis**, Chiralpak AD-H (97.5:2.5 hexane/IPA, flow rate 1 $mLmin^{-1}$, 211 nm, 30 °C) t_R (major): 40.4 min, t_R (minor): 44.9 min, 97:3 er; **^{1}H NMR** (400 MHz, $CDCl_3$) δ_H: 2.31 (1H, dd, *J* 13.8, 2.9, C(7)H^AH^B), 2.36 (1H, s, O*H*), 2.80 (1H, app t, *J* 13.6, C(7)H^AH^B), 3.50 (3H, s, OCH_3), 4.08 (1H, ddd, *J* 13.5, 6.0, 2.8, C(6)*H*), 5.15 (1H, d, *J* 5.9, C(5)*H*), 6.19 (1H, dt, *J* 3.2, 0.9, C(6)Ar*H*), 6.26 (1H, dd, *J* 3.9, 2.9, C(2)*H*), 6.35 (1H, dd, *J* 3.3, 1.9, C(6)Ar*H*), 6.47 (1H, dq, *J* 3.7, 1.7, C(1)*H*), 6.65 (1H, dd, *J* 2.8, 1.6, C(3)*H*), 7.42 (1H, dd, *J* 1.9, 0.8, C(6)Ar*H*); **^{13}C{^{1}H} NMR** (126 MHz, $CDCl_3$) δ_C: 28.1 (*C*(7)H_2), 32.6 (*C*(6)H), 52.6 (O*C*H_3), 60.0 (*C*(5)H), 70.3 (q, $^2J_{CF}$ 30.6, *C*(8)), 106.8 (C(6)Ar*C*H), 107.9 (app d, *J* 2.4, *C*(1)H), 110.4 (*C*(2)H), 110.5 (C(6)Ar*C*H), 121.7 (*C*(3)H), 125.2 (q, $^1J_{CF}$ 284.2, *C*F_3), 125.8 (*C*(8a)), 142.6 (C(6)Ar*C*H), 151.9 (C(6)Ar*C*(2)), 169.3 (*C*=O); **^{19}F NMR** (471 MHz, $CDCl_3$) δ_F: −80.7 (CF_3).

Data for minor diastereoisomer: **Chiral HPLC analysis**, Chiralpak AD-H (97.5:2.5 hexane/IPA, flow rate 1 $mLmin^{-1}$, 211 nm, 30 °C) t_R (major): 25.0 min, t_R (minor): 31.8 min, 92:8 er; **^{1}H NMR** (400 MHz, $CDCl_3$) δ_H (selected): 2.62 (1H, s, O*H*), 3.75 (3H, s, OCH_3), 4.77 (1H, d, *J* 10.9, C(5)*H*), 6.20–6.21 (1H, m, C(6)ArC(3)*H*), 6.24–6.25 (1H, m, C(2)*H*), 6.33–6.34 (1H, m, C(6)ArC(4)*H*), 6.43–6.46 (1H, m, C(1)*H*), 6.59 (1H, dd, *J* 2.9, 1.5, C(3)*H*), 7.39 (1H, dd, *J* 1.8, 0.9, C(6)ArC(5)*H*); **^{19}F NMR** (471 MHz, $CDCl_3$) δ_F: −80.3 (CF_3).

Methyl 2-oxo-4-phenyl-3-(1*H*-pyrrol-1-yl)-2*H*-pyran-6-carboxylate (28)

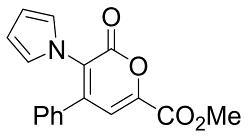

2-(1*H*-Pyrrol-1-yl)acetic acid **1** (31.3 mg, 0.25 mmol, 1 eq.) was dissolved in anhydrous MeCN (0.1 M) under an atmosphere of N_2 and cooled to 0 °C before *i*-Pr_2NEt (87 μL, 0.5 mmol, 2 eq.) and pivaloyl chloride (62 μL, 0.5 mmol, 2 eq.) were added. The reaction was stirred at 0 °C for 20 min before warming to rt. HyperBTM **11** (7.7 mg, 10 mol%), α-keto-β,γ-unsaturated ester **27** (48 mg, 0.25 mmol, 1 eq.) and *i*-Pr_2NEt (110 μL, 0.63 mmol, 2.5 eq.) were added sequentially and the reaction stirred at rt for 24 h. Upon completion of the reaction (checked by TLC), the solvent was concentrated under reduced pressure and the crude was purified by flash silica column chromatography (15:85 EtOAc/petrol) to give the title compound as a yellow oil (34 mg, 58%).

IR ν_{max} (film, cm^{-1}) 3107 (C-H), 2955 (C-H), 1736 (C=O), 1726 (C=O), 1643 (C=C), 1244; **^{1}H NMR** (500 MHz, $CDCl_3$) δ_H: 3.97 (3H, s, OC*H*$_3$), 6.18 (2H, t, *J* 2.2, C(3)Ar(3,4)*H*), 6.56 (2H, t, *J* 2.2, C(3)Ar(2,5)*H*), 7.06–7.08 (2H, m, PhC(2,6)*H*), 7.32–7.38 (4H, m, PhC(3,4,5)*H* + C(5)*H*); **^{13}C{^{1}H} NMR** (126 MHz, $CDCl_3$) δ_C: 53.4 (*C*H$_3$), 110.6 (C(3)Ar*C*(3,4)H), 113.8 (*C*(5)H), 121.9 (C(3)Ar*C*(2,5)H), 127.8 (Ph*C*(2,6)H), 129.1 (Ph*C*(3,5)H), 130.4 (Ph*C*(4)H), 133.8 (Ph*C*(1)), 144.9 (*C*(4)), 145.8 (*C*(3)), 158.5 (*C*(2)=O), 159.7 (C(6)*C*O$_2$Me); **HRMS** (ESI$^+$) $C_{17}H_{14}NO_4$ $[M+H]^+$ found 296.0918, requires 296.0917 (+0.2 ppm).

Methyl (3*E*)-2-((1*H*-pyrrol-1-yl)methylene)-4-phenylbut-3-enoate (29)

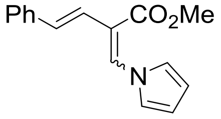

In the above experiment, the title compound was also obtained following column chromatography as a colourless oil (11 mg, 18%, 88:12 mixture of stereoisomers).

IR ν_{max} (film, cm^{-1}) 3026 (C-H stretch), 2951 (C-H stretch), 1713 (C=C), 1225; **^{1}H NMR** (500 MHz, $CDCl_3$) δ_H: 3.86 (3H, s, OC*H*$_3$), 6.32 (2H, t, *J* 2.2, pyrroleC(3,4)*H*), 6.97 (1H, dd, *J* 16.4, 1.0, C*H*=CHPh), 7.05 (2H, t, *J* 2.2, pyrroleC(2,5)*H*), 7.18 (1H, d, *J* 16.4, CH=C*H*Ph), 7.28–7.29 (1H, m, PhC(4)*H*), 7.34–7.37 (2H, m, PhC(3,5)*H*), 7.46–7.47 (2H, m, PhC(2,6)*H*), 7.84 (1H, s, C(2)=C*H*); **^{13}C{^{1}H} NMR** (126 MHz, $CDCl_3$) δ_C: 52.3 (O*C*H$_3$), 112.0 (pyrrole*C*(3,4)H), 115.6 (*C*(2)), 120.0 (*C*H=CHPh), 122.9 (pyrrole*C*(2,5)H), 126.8 (Ar*C*(2,6)H), 128.2 (Ar*C*(4)H), 128.9 (Ar*C*(3,5)H), 135.4 (CH=*C*HPh), 136.2 (C(2)=*C*H), 137.3 (Ph*C*(1)), 167.7 (*C*O$_2$Me); **HRMS** (NSI$^+$) $C_{16}H_{16}NO_2$ $[M+H]^+$ found 254.1177, requires 254.1176 (+0.4 ppm).

(4*S*,5*S*)-1,6-Dimorpholino-4-phenyl-5-(1*H*-pyrrol-1-yl)hexane-1,2,6-trione (31)

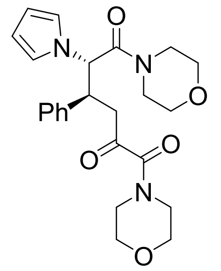

Following **General Procedure B**, 2-(1*H*-pyrrol-1-yl)acetic acid **1** (31 mg, 0.25 mmol, 1 eq.), *i*-Pr_2NEt (87 μL, 0.5 mmol), pivaloyl chloride (62 μL, 0.5 mmol) in anhydrous MeCN (2.5 mL) at 0 °C for 20 min followed by HyperBTM **11** (7.7 mg, 10 mol%), α-keto-β,γ-unsaturated ester **27** (48 mg, 0.25 mmol, 1 eq.) and *i*-Pr_2NEt (110 μL, 0.63 mmol) at –40 °C for 24 h. Ring-opening with morpholine (300 eq.) at rt for 24 h gave crude product (> 95:5 dr) that was purified by column chromatography (30:70 EtOAc/petrol) to give the title compound as a colourless solid (77 mg, 70%).

mp 154–156 °C; $[\alpha]_D^{20}$ −50.7 (*c* 1.1 in $CHCl_3$); **Chiral HPLC analysis**, Chiralcel OD-H (80:20 hexane/*i*-PrOH, flow rate 1 mLmin^{-1}, 211 nm, 30 °C) t_R (major): 25.2 min, t_R (minor): 33.3 min, > 99:1 er; **IR** ν_{max} (film, cm^{-1}) 2967 (C-H), 2920 (C-H), 2857 (C-H), 1713 (C=O), 1638 (amide C=O), 1441, 1271, 1113; **^{1}H NMR** (500 MHz, $CDCl_3$) δ_H: 2.91 (1H, ddd, *J* 11.2, 7.8, 3.1, morphC*H*), 3.06–3.08 (2H, m, morphC*H*), 3.25–3.40 (7H, m, morphC*H* + C(3)H_2), 3.45–3.69 (7H, m, morphC*H*), 3.85–3.89 (1H, m, morphC*H*), 4.08 (1H, app td, *J* 9.7, 4.6, C(4)*H*), 4.78 (1H, d, *J* 10.0, C(5)*H*), 5.90 (2H, t, *J* 2.1, C(5)Ar(3,4)*H*), 6.33 (2H, t, *J* 2.1, C(5)Ar(2,5)*H*), 7.00–7.02 (2H, m, PhC*H*), 7.12–7.17 (3H, m, PhC*H*); **^{13}C{^{1}H} NMR** (126 MHz, $CDCl_3$) δ_C: 42.0 (morph*C*H_2), 42.8 (*C*(3)H_2), 43.0 (morph*C*H_2), 44.4 (*C*(4)H), 45.8 (morph*C*H_2), 46.4 (morph*C*H_2), 63.6 (*C*(5)H), 66.3 (morph*C*H_2), 66.5 (morph*C*H_2), 66.6 (morph*C*H_2), 66.7 (morph*C*H_2), 109.0 (C(5)Ar*C*(3,4)H), 119.6 (C(5)Ar*C*(2,5)H), 127.5 (Ph*C*(4)H), 128.1 (2 x Ph*C*H), 128.6 (2 x Ph*C*H), 139.3 (Ph*C*(1)), 164.4 (*C*(6)=O), 166.4 (*C*(2)=O), 197.8 (*C*(1)=O); **HRMS** (ESI$^+$) $C_{24}H_{29}N_3O_5Na$ $[M+Na]^+$ found 462.1988, requires 462.1999 (−2.4 ppm).

(4*S*,5*S*)-4-Phenyl-5-(1*H*-pyrrol-1-yl)-1,6-di(pyrrolidin-1-yl)hexane-1,2,6-trione (32)

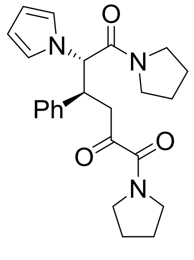

Following **General Procedure B**, 2-(1*H*-pyrrol-1-yl)acetic acid **1** (31 mg, 0.25 mmol, 1 eq.), *i*-Pr_2NEt (87 μL, 0.5 mmol), pivaloyl chloride (62 μL, 0.5 mmol) in anhydrous MeCN (2.5 mL) at 0 °C for 20 min followed by HyperBTM **11** (7.7 mg, 10 mol%), α-keto-β,γ-unsaturated ester **27** (48 mg, 0.25 mmol, 1

eq.) and *i*-Pr_2NEt (110 μL, 0.63 mmol) at –40 °C for 24 h. Ring-opening with pyrrolidine (300 eq.) at rt for 24 h gave crude product (> 95:5 dr) that was purified by column chromatography (30:70 EtOAc/petrol) to give the title compound as a colourless solid (75 mg, 74%).

mp 148–151 °C; $[\alpha]_D^{20}$ +91.0 (*c* 1.5 in $CHCl_3$); **Chiral HPLC analysis**, Chiralcel OD-H (90:10 hexane/*i*-PrOH, flow rate 1 mLmin^{-1}, 211 nm, 30 °C) t_R (major): 21.3 min, t_R (minor): 27.8 min, > 99:1 er; **IR** ν_{max} (film, cm^{-1}) 2974 (C-H), 2878 (C-H), 1715 (C=O), 1638 (amide C=O), 1605 (C=O), 1447; **^{1}H NMR** (500 MHz, $CDCl_3$) δ_H: 1.70–1.98 (8H, m, pyrrolidineC*H*), 3.17–3.30 (4H, m, C(3)H^AH^B + pyrrolidineC*H*), 3.38–3.56 (6H, m, C(3)H^AH^B + pyrrolidineC*H*), 4.07 (1H, app td, *J* 10.2, 4.3, C(4)*H*), 4.69 (1H, d, *J* 10.4, C(5)*H*), 5.90 (2H, t, *J* 2.1, C(5)Ar(3,4)*H*), 6.48 (2H, t, *J* 2.2, C(5)Ar(2,5)*H*), 7.02–7.05 (2H, m, PhC(2,6)*H*), 7.09–7.15 (3H, m, PhC(3,4,5)*H*); **^{13}C{^{1}H} NMR** (126 MHz, $CDCl_3$) δ_C: 23.6 (pyrrolidine*C*H_2), 24.2 (pyrrolidine*C*H_2), 26.2 (pyrrolidine*C*H_2), 26.4 (pyrrolidine*C*H_2), 41.8 (*C*(3)H_2), 44.6 (*C*(4)H), 46.4 (pyrrolidine*C*H_2), 46.5 (pyrrolidine*C*H_2), 46.5 (pyrrolidine*C*H_2), 47.2 (pyrrolidine*C*H_2), 65.7 (*C*(5)H), 108.3 (C(5)Ar*C*(3,4)H), 120.2 (C(5)Ar*C*(2,5)H), 127.1 (Ph*C*(4)H), 128.1 (Ph*C*(3,5)H), 128.4 (Ph*C*(2,6)H), 139.7 (Ph*C*(1)), 162.3 (*C*(6)=O), 166.6 (*C*(2)=O), 197.8 (*C*(1)=O); **HRMS** (ESI$^+$) $C_{24}H_{29}N_3O_3Na$ [M+Na]$^+$ found 430.2091, requires 430.2101 (−2.3 ppm).

Methyl (5*S*,6*S*,8*R*)-8-hydroxy-5-(morpholine-4-carbonyl)-6-phenyl-5,6,7,8-tetrahydroindolizine-8-carboxylate (33)

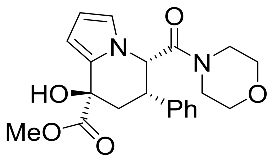

Following **General Procedure B**, 2-(1*H*-pyrrol-1-yl)acetic acid **1** (31 mg, 0.25 mmol, 1 eq.), *i*-Pr_2NEt (87 μL, 0.5 mmol), pivaloyl chloride (62 μL, 0.5 mmol) in anhydrous MeCN (2.5 mL) at 0 °C for 20 min followed by HyperBTM **11** (7.7 mg, 10 mol%), α-keto-β,γ-unsaturated ester **27** (48 mg, 0.25 mmol, 1 eq.) and *i*-Pr_2NEt (110 μL, 0.63 mmol) at –40 °C for 24 h. Ring-opening with morpholine (2 eq.) at rt for 24 h gave crude product (> 95:5 dr) that was purified by column chromatography (30:70 EtOAc/petrol) to give the title compound as a light yellow oil (72 mg, 75%).

$[\alpha]_D^{20}$ +50.2 (*c* 2.4 in $CHCl_3$); **Chiral HPLC analysis**, Chiralpak AD-H (80:20 hexane/IPA, flow rate 1 mLmin^{-1}, 211 nm, 30 °C) t_R (major): 18.8 min, t_R (minor): 31.9 min, > 99:1 er; **IR** ν_{max} (film, cm^{-1}) 3399 (O-H stretch), 2961 (C-H), 2926 (C-H), 2857 (C-H), 1732 (C=O), 1643 (amide C=O); **^{1}H NMR** (500 MHz, $CDCl_3$) δ_H: 2.02 (1H, dd, *J* 13.1, 2.6, C(7)H^AH^B), 2.48–2.54 (2H, m, morphC*H*), 3.16 (1H, ddd, *J* 13.7, 7.5, 3.1, morphC*H*), 3.27–3.36 (3H, m, morphC*H*), 3.48–3.52 (2H, m, morphC*H*), 3.55 (1H, d, *J* 1.3, O*H*), 3.73 (1H, app td, *J* 13.3, 1.3, C(7)H^AH^B), 3.90 (3H, s, OCH_3), 4.10 (1H, ddd, *J* 13.5, 6.0, 2.6, C(6)*H*), 5.34 (1H, d, *J* 6.0, C(5)*H*), 6.10 (1H, dd, *J* 3.7, 1.6, C(2)*H*), 6.21 (1H, dd, *J* 3.7, 2.8, C(1)*H*), 6.41 (1H, dd, *J* 2.8, 1.6, C(3)*H*), 7.31–7.39 (5H, m, PhC*H*); **^{13}C{^{1}H} NMR** (126 MHz, $CDCl_3$) δ_C: 32.8 (*C*(7)H_2), 39.5 (*C*(6)H),

42.1 (morphCH_2), 45.9 (morphCH_2), 53.7 (OCH_3), 56.2 (*C*(5)H), 65.8 (morphCH_2), 66.4 (morphCH_2), 71.2 (C(8)), 105.9 (*C*(2)H), 110.1 (*C*(1)H), 119.5 (*C*(3)H), 128.3 (Ph*C*(4)H), 128.8 (2 × Ph*C*H), 129.1 (2 × Ph*C*H), 130.3 (*C*(8a)), 138.9 (Ph*C*(1)), 167.2 (C(5)*C*=O), 175.1 (C(8)*C*=O); **HRMS** (NSI^+) $C_{21}H_{25}N_2O_5$ $[M+H]^+$ found 385.1760, requires 385.1758 (+0.5 ppm).

Methyl (5*S*,6*S*,8*R*)-8-hydroxy-6-phenyl-5-(pyrrolidine-1-carbonyl)-5,6,7,8-tetrahydroindolizine-8-carboxylate (34)

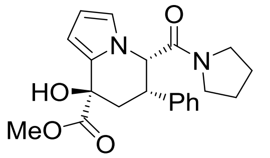

Following **General Procedure B**, 2-(1*H*-pyrrol-1-yl)acetic acid **1** (31 mg, 0.25 mmol, 1 eq.), *i*-Pr_2NEt (87 μL, 0.5 mmol), pivaloyl chloride (62 μL, 0.5 mmol) in anhydrous MeCN (2.5 mL) at 0 °C for 20 min followed by HyperBTM **11** (7.7 mg, 10 mol%), α-keto-β,γ-unsaturated ester **27** (48 mg, 0.25 mmol, 1 eq.) and *i*-Pr_2NEt (110 μL, 0.63 mmol) at –40 °C for 24 h. Ring-opening with pyrrolidine (2 eq.) at rt for 24 h gave crude product (> 95:5 dr) that was purified by column chromatography (30:70 EtOAc/petrol) to give the title compound as a light yellow oil (66 mg, 72%).

$[\alpha]_D^{20}$ +123 (*c* 1.8 in $CHCl_3$); **Chiral HPLC analysis**, Chiralcel OD-H (80:20 hexane/IPA, flow rate 1 $mLmin^{-1}$, 211 nm, 30 °C) t_R(major): 21.2 min, t_R(minor): 29.3 min, > 99:1 er; **IR** ν_{max}(film, cm^{-1}) 3393 (O-H stretch), 2972 (C-H), 2951 (C-H), 1732 (C=O), 1643 (amide C=O); **^{1}H NMR** (500 MHz, $CDCl_3$) δ_H: 1.22–1.29 (1H, m, pyrrolidineC*H*), 1.44–1.52 (2H, m, pyrrolidineC*H*), 1.57–1.70 (1H, m, pyrrolidineC*H*), 1.99 (1H, dd, *J* 13.0, 2.5, C(7)H^AH^B), 2.21–2.25 (1H, m, pyrrolidineC*H*), 3.11–3.22 (2H, m, pyrrolidineC*H*), 3.33–3.38 (1H, m, pyrrolidineC*H*), 3.54 (1H, s, O*H*), 3.78 (1H, app t, *J* 13.3, C(7)H^AH^B), 3.88 (3H, s, OCH_3), 4.07 (1H, ddd, *J* 13.5, 5.9, 2.5, C(6)*H*), 5.11 (1H, d, *J* 5.9, C(5)*H*), 6.09 (1H, dd, *J* 3.7, 1.6, C(2)*H*), 6.19 (1H, dd, *J* 3.7, 2.8, C(1)*H*), 6.44 (1H, dd, *J* 2.8, 1.6, C(3)*H*), 7.27–7.36 (5H, m, PhC*H*); **^{13}C{^{1}H} NMR** (126 MHz, $CDCl_3$) δ_C: 23.9 (pyrrolidineCH_2), 26.1 (pyrrolidineCH_2), 33.0 (*C*(7)H_2), 39.8 (*C*(6)H), 45.8 (pyrrolidineCH_2), 46.4 (pyrrolidineCH_2), 53.5 (OCH_3), 59.5 (*C*(5)H), 71.2 (*C*(8)), 105.7 (*C*(2)H), 109.9 (*C*(1)H), 119.6 (*C*(3)H), 128.0 (Ph*C*(4)H), 128.6 (4 x Ph*C*H), 130.2 (*C*(8a)), 139.1 (Ph*C*(1)), 166.8 (C(5)*C*=O), 175.2 (C(8)*C*=O); **HRMS** (NSI^+) $C_{21}H_{25}N_2O_4$ $[M+H]^+$ found 369.1810, requires 369.1809 (+0.3 ppm).

Ethyl (5*S*,6*S*,8*R*)-8-hydroxy-5-(morpholine-4-carbonyl)-6-phenyl-5,6,7,8-tetrahydroindolizine-8-carboxylate (35)

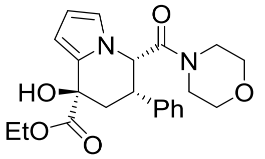

Following **General Procedure B**, 2-(1*H*-pyrrol-1-yl)acetic acid **1** (31 mg, 0.25 mmol, 1 eq.), *i*-Pr_2NEt (87 μL, 0.5 mmol), pivaloyl chloride (62 μL, 0.5 mmol) in anhydrous MeCN (2.5 mL) at 0 °C for 20 min followed by HyperBTM **11** (7.7 mg, 10 mol%), α-keto-β,γ-unsaturated ester **S11** (51 mg, 0.25 mmol, 1 eq.) and *i*-Pr_2NEt (110 μL, 0.63 mmol) at –40 °C for 24 h. Ring-opening with morpholine (2 eq.) at rt for 24 h gave crude product (> 95:5 dr) that was purified by column chromatography (20:80 EtOAc/petrol) to give the title compound as a colourless oil (55 mg, 57%).

$[\alpha]_D^{20}$ +102 (*c* 1.2 in $CHCl_3$); **Chiral HPLC analysis**, Chiralpak AD-H (90:10 hexane/IPA, flow rate 1 $mLmin^{-1}$, 211 nm, 30 °C) t_R(major): 31.4 min, t_R(minor): 63.7 min, > 99:1 er; **IR** ν_{max}(film, cm^{-1}) 3420 (O-H stretch), 2978 (C-H), 2928 (C-H), 2859 (C-H), 1730 (C=O), 1649 (amide C=O); **^{1}H NMR** (500 MHz, $CDCl_3$) δ_H: 1.36 (3H, t, *J* 7.1, CH_3), 2.00 (1H, dd, *J* 13.0, 2.6, C(7)H^AH^B), 2.50–2.55 (2H, m, morphC*H*), 3.14–3.19 (1H, m, morphC*H*), 3.27–3.37 (3H, m, morphC*H*), 3.45–3.51 (2H, m, morphC*H*), 3.57 (1H, d, *J* 1.3, O*H*), 3.71 (1H, app t, *J* 13.3, C(7)H^AH^B), 4.11 (1H, ddd, *J* 13.5, 6.1, 2.6, C(6)*H*), 4.27 (2H, q, *J* 7.1, CH_2CH_3), 5.34 (1H, d, *J* 6.1, C(5)*H*), 6.10 (1H, dd, *J* 3.7, 1.6, C(2)*H*), 6.20 (1H, dd, *J* 3.7, 2.8, C(1)*H*), 6.41 (1H, dd, *J* 2.8, 1.6, C(3)*H*), 7.31–7.38 (5H, m, PhC*H*); **^{13}C{^{1}H} NMR** (126 MHz, $CDCl_3$) δ_C: 14.3 (CH_3), 32.8 ($C(7)H_2$), 39.4 (*C*(6)H), 42.1 (morphCH_2), 45.9 (morphCH_2), 56.2 (*C*(5)H), 62.7 (CH_2CH_3), 65.8 (morphCH_2), 66.4 (morphCH_2), 71.1 (*C*(8)), 105.7 (*C*(2)H), 110.0 (*C*(1)H), 119.5 (*C*(3)H), 128.2 (Ph*C*(4)H), 128.8 (2 × PhCH), 129.0 (2 × PhCH), 130.5 (*C*(8a)), 139.1 (C(6)Ar*C*(1)), 167.3 (C(5)*C*=O), 174.3 (C(8)*C*=O); **HRMS** (ESI^+) $C_{22}H_{26}N_2O_5Na$ $[M+Na]^+$ found 421.1723, requires 421.1734 (−2.6 ppm).

Isopropyl (5*S*,6*S*,8*R*)-8-hydroxy-5-(morpholine-4-carbonyl)-6-phenyl-5,6,7,8-tetrahydroindolizine-8-carboxylate (36)

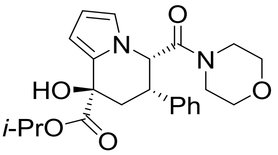

Following **General Procedure B**, 2-(1*H*-pyrrol-1-yl)acetic acid **1** (31 mg, 0.25 mmol, 1 eq.), *i*-Pr_2NEt (87 μL, 0.5 mmol), pivaloyl chloride (62 μL, 0.5 mmol) in anhydrous MeCN (2.5 mL) at 0 °C for 20 min followed by HyperBTM **11** (7.7 mg, 10 mol%), α-keto-β,γ-unsaturated ester **S12** (55 mg, 0.25 mmol, 1 eq.) and *i*-Pr_2NEt (110 μL, 0.63 mmol) at –40 °C for 24 h. Ring-opening with morpholine (2 eq.) at rt for 24 h gave crude product (> 95:5 dr) that was purified by column chromatography (30:70 EtOAc/petrol) to give the title compound as a brown oil (66 mg, 64%).

$[\alpha]_D^{20}$ +77.5 (*c* 1.0 in $CHCl_3$); **Chiral HPLC analysis**, Chiralpak AD-H (90:10 hexane : IPA, flow rate 1 mLmin^{-1}, 211 nm, 30 °C) t_R (major): 27.3 min, t_R (minor): 56.9 min, > 99:1 er; **IR** ν_{max} (film, cm^{-1}) 3414 (O-H stretch), 2974 (C-H), 2936 (C-H), 2922 (C-H), 1713 (C=O), 1649 (amide C=O), 1234, 1111; **^{1}H NMR** (400 MHz, $CDCl_3$) δ_H: 1.33 (3H, d, *J* 6.2, C*H*$_3$), 1.37 (3H, d, *J* 6.3, C*H*$_3$), 1.99 (1H, dd, *J* 13.1, 2.5, C(7)H^AH^B), 2.49–2.56 (2H, m, morphC*H*), 3.14–3.20 (1H, m, morphC*H*), 3.26–3.38 (3H, m, morphC*H*), 3.45–3.51 (2H, m, morphC*H*), 3.58 (1H, d, *J* 1.3, O*H*), 3.68 (1H, td, *J* 13.2, 1.3, C(7)H^AH^B), 4.13 (1H, ddd, *J* 13.2, 6.1, 2.5, C(6)*H*), 5.20 (1H, hept, *J* 6.3, C*H*(CH_3)$_2$), 5.34 (1H, d, *J* 6.1, C(5)*H*), 6.08 (1H, dd, *J* 3.7, 1.6, C(2)*H*), 6.19 (1H, dd, *J* 3.7, 2.8, C(1)*H*), 6.40 (1H, dd, *J* 2.8, 1.6, C(3)*H*), 7.33–7.37 (5H, m, C(6)ArC*H*); **^{13}C{^{1}H} NMR** (126 MHz, $CDCl_3$) δ_C: 21.6 (*C*H$_3$), 21.8 (*C*H$_3$), 32.7 (*C*(7)H$_2$), 39.4 (*C*(6)H), 42.1 (morph*C*H$_2$), 45.9 (morph*C*H$_2$), 56.2 (*C*(5)H), 65.9 (morph*C*H$_2$), 66.5 (morph*C*H$_2$), 70.6 (*C*H(CH_3)$_2$), 71.1 (*C*(8)), 105.6 (*C*(2)H), 110.0 (*C*(3)H), 119.4 (*C*(1)H), 128.2 (Ph*C*(4)H), 128.8 (2 x Ph*C*H), 129.0 (2 x Ph*C*H), 130.7 (*C*(8a)), 139.1 (PhC(1)), 167.3 (C(5)*C*=O), 173.7 (C(8)*C*=O); **HRMS** (ESI$^+$) $C_{23}H_{28}N_2O_5Na$ $[M+Na]^+$ found 435.1882, requires 435.1890 (−1.8 ppm).

Methyl (5*S*,6*S*,8*R*)-8-hydroxy-5-(morpholine-4-carbonyl)-6-(naphthalen-2-yl)-5,6,7,8-tetrahydroindolizine-8-carboxylate (37)

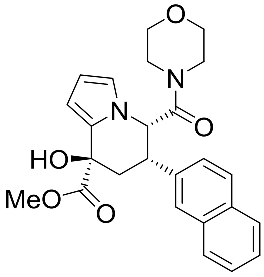

Following **General Procedure B**, 2-(1*H*-pyrrol-1-yl)acetic acid **1** (31 mg, 0.25 mmol, 1 eq.), *i*-Pr$_2$NEt (87 µL, 0.5 mmol), pivaloyl chloride (62 µL, 0.5 mmol) in anhydrous MeCN (2.5 mL) at 0 °C for 20 min followed by HyperBTM **11** (7.7 mg, 10 mol%), α-keto-β,γ-unsaturated ester **S13** (60 mg, 0.25 mmol, 1 eq.) and *i*-Pr$_2$NEt (110 µL, 0.63 mmol) at –40 °C for 24 h. Ring-opening with morpholine (2 eq.) at rt for 24 h gave crude product (> 95:5 dr) that was purified by column chromatography (20:80 EtOAc/petrol) to give the title compound as a colourless oil (86 mg, 79%).

$[\alpha]_D^{20}$ +75.6 (*c* 2.3 in $CHCl_3$); **Chiral HPLC analysis**, Chiralpak IB (80:20 hexane/IPA, flow rate 1 mLmin^{-1}, 211 nm, 30 °C) t_R (major): 50.6 min, t_R (minor): 65.4 min, > 99:1 er; **IR** ν_{max} (film, cm^{-1}) 3402 (O-H stretch), 3055 (C-H), 2955 (C-H), 2857 (C-H), 1719 (C=O), 1645 (amide C=O); **^{1}H NMR** (500 MHz, $CDCl_3$) δ_H: 2.09–2.15 (2H, m, C(7)H^AH^B + morphC*H*), 2.39 (1H, ddd, *J* 13.0, 6.3, 3.0, morphC*H*), 3.04–3.11 (3H, m, morphC*H*), 3.27–3.32 (1H, m, morphC*H*), 3.37–3.45 (2H, m, morphC*H*), 3.63 (1H, s, O*H*), 3.85 (1H, t, *J* 13.3, C(7)H^AH^B), 3.92 (3H, s, OC*H*$_3$), 4.26 (1H, ddd, *J* 13.5, 6.0, 2.6, C(6)*H*), 5.42 (1H, d, *J* 6.0, C(5)*H*), 6.13 (1H, dd, *J* 3.7, 1.6, C(2)*H*), 6.22 (1H, dd, *J* 3.7, 2.7, C(1)*H*), 6.43 (1H, dd, *J* 2.8, 1.6,

C(3)*H*), 7.44 (1H, dd, *J* 8.5, 1.8, C(6)ArC*H*), 7.49–7.53 (2H, m, C(6)ArC*H*), 7.79–7.85 (4H, m, C(6)ArC*H*); **^{13}C{^{1}H} NMR** (126 MHz, $CDCl_3$) δ_C: 33.0 (*C*(7)H_2), 39.6 (*C*(6)H), 42.1 (morph*C*H_2), 45.9 (morph*C*H_2), 53.6 (O*C*H_3), 56.3 (*C*(5)H), 65.6 (morph*C*H_2), 66.3 (morph*C*H_2), 71.2 (*C*(8)), 105.9 (*C*(2)H), 110.1 (*C*(1)H), 119.5 (*C*(3)H), 126.6 (C(6)Ar*C*H), 126.6 (C(6)Ar*C*H), 126.9 (C(6)Ar*C*H), 127.4 (C(6)Ar*C*H), 127.7 (C(6)Ar*C*H), 127.8 (C(6)Ar*C*H), 128.5 (C(6)Ar*C*H), 130.4 (*C*(8a)), 132.9 (C(6)Ar*C*), 133.6 (C(6)Ar*C*), 136.2 (C(6)Ar*C*(1)), 167.2 (C(5)*C*=O), 175.0 (C(8)*C*=O); **HRMS** (ESI^+) $C_{25}H_{26}N_2O_5Na$ $[M+Na]^+$ found 457.1726, requires 457.1734 (−1.7 ppm).

Methyl (5*S*,6*S*,8*R*)-8-hydroxy-6-(4-methoxyphenyl)-5-(morpholine-4-carbonyl)-5,6,7,8-tetrahydroindolizine-8-carboxylate (38)

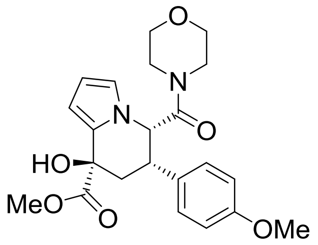

Following **General Procedure B**, 2-(1*H*-pyrrol-1-yl)acetic acid **1** (31 mg, 0.25 mmol, 1 eq.), *i*-Pr_2NEt (87 μL, 0.5 mmol), pivaloyl chloride (62 μL, 0.5 mmol) in anhydrous CH_2Cl_2 (2.5 mL) at 0 °C for 20 min followed by HyperBTM **11** (7.7 mg, 10 mol%), α-keto-β,γ-unsaturated ester **S15** (55 mg, 0.25 mmol, 1 eq.) and *i*-Pr_2NEt (110 μL, 0.63 mmol) at –40 °C for 24 h. Ring-opening with morpholine (2 eq.) at rt for 24 h gave crude product (> 95:5 dr) that was purified by column chromatography (20:80 EtOAc/petrol) to give the title compound as a light yellow oil (52 mg, 50%).

$[\alpha]_D^{20}$ +99.3 (*c* 1.7 in $CHCl_3$); **Chiral HPLC analysis**, Chiralpak AD-H (90:10 hexane/IPA, flow rate 1 $mLmin^{-1}$, 211 nm, 30 °C) t_R (major): 56.4 min, t_R (minor): 80.8 min, > 99:1 er; **IR** ν_{max} (film, cm^{-1}) 3406 (O-H stretch), 2959 (C-H), 2857 (C-H), 1732 (C=O), 1645 (amide C=O); **^{1}H NMR** (500 MHz, $CDCl_3$) δ_H: 1.97 (1H, dd, *J* 13.1, 2.6, C(7)H^AH^B), 2.55 (1H, ddd, *J* 13.2, 6.5, 3.0, morphC*H*), 2.69 (1H, ddd, *J* 11.5, 6.8, 3.0, morphC*H*), 3.20 (1H, ddd, *J* 13.2, 6.9, 3.1, morphC*H*), 3.33–3.52 (5H, m, morphC*H*), 3.55 (1H, d, *J* 1.4, O*H*), 3.66 (1H, t, *J* 13.3, C(7)H^AH^B), 3.81 (3H, s, OCH_3), 3.88 (3H, s, OCH_3), 4.04 (1H, ddd, *J* 13.6, 6.1, 2.6, C(6)*H*), 5.31 (1H, d, *J* 6.1, C(5)*H*), 6.09 (1H, dd, *J* 3.7, 1.6, C(2)*H*), 6.19 (1H, dd, *J* 3.7, 2.7, C(1)*H*), 6.40 (1H, dd, *J* 2.7, 1.6, C(3)*H*), 6.86–6.89 (2H, m, C(6)ArC(3,5)*H*), 7.22–7.25 (2H, m, C(6)ArC(2,6)*H*); **^{13}C{^{1}H} NMR** (126 MHz, $CDCl_3$) δ_C: 33.2 (*C*(7)H_2), 38.6 (*C*(6)H), 42.1 (morph*C*H_2), 45.9 (morph*C*H_2), 53.6 (O*C*H_3), 55.5 (O*C*H_3), 56.3 (*C*(5)H), 65.9 (morph*C*H_2), 66.5 (morph*C*H_2), 71.2 (*C*(8)), 105.8 (*C*(2)H), 110.0 (*C*(1)H), 114.3 (C(6)Ar*C*(3,5)H), 119.5 (*C*(3)H), 129.7 (C(6)Ar*C*(2,6)H), 130.3 (*C*(8a)), 130.9 (C(6)Ar*C*(1)), 159.5 (C(6)Ar*C*(4)), 167.4 (C(5)*C*=O), 175.0 (C(8)*C*=O); **HRMS** (ESI^+) $C_{22}H_{26}N_2O_6Na$ $[M+Na]^+$ found 437.1668, requires 437.1683 (−3.5 ppm).

Methyl (5*S*,6*S*,8*R*)-8-hydroxy-5-(morpholine-4-carbonyl)-6-(*p*-tolyl)-5,6,7,8-tetrahydroindolizine-8-carboxylate (39)

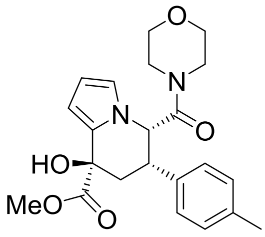

Following **General Procedure B**, 2-(1*H*-pyrrol-1-yl)acetic acid **1** (31 mg, 0.25 mmol, 1 eq.), *i*-Pr_2NEt (87 μL, 0.5 mmol), pivaloyl chloride (62 μL, 0.5 mmol) in anhydrous MeCN (2.5 mL) at 0 °C for 20 min followed by HyperBTM **11** (7.7 mg, 10 mol%), α-keto-β,γ-unsaturated ester **S14** (51 mg, 0.25 mmol, 1 eq.) and *i*-Pr_2NEt (110 μL, 0.63 mmol) at –40 °C for 24 h. Ring-opening with morpholine (2 eq.) at rt for 24 h gave crude product (> 95:5 dr) that was purified by column chromatography (20:80 EtOAc/petrol) to give the title compound as a light yellow oil (95 mg, 95%).

$[\alpha]_D^{20}$ +97.0 (*c* 2.1 in $CHCl_3$); **Chiral HPLC analysis**, Chiralpak AD-H (90:10 hexane/IPA, flow rate 1 mLmin^{-1}, 211 nm, 30 °C) t_R (major): 38.5 min, t_R (minor): 55.0 min, > 99:1 er; **IR** ν_{max} (film, cm^{-1}) 3410 (O-H stretch), 2955 (C-H), 2926 (C-H), 2859 (C-H), 1715 (C=O), 1645 (amide C=O); **^{1}H NMR** (500 MHz, $CDCl_3$) δ_H: 1.98 (1H, dd, *J* 13.1, 2.6, C(7)H^AH^B), 2.36 (3H, s, CH_3), 2.50–2.59 (2H, m, morphC*H*), 3.14–3.19 (1H, m, morphC*H*), 3.30–3.39 (3H, m, morphC*H*), 3.44–3.58 (3H, m, morphC*H* + O*H*), 3.69 (1H, t, *J* 13.3, C(7)H^AH^B), 3.89 (3H, s, OCH_3), 4.05 (1H, ddd, *J* 13.6, 6.0, 2.6, C(6)*H*), 5.31 (1H, d, *J* 6.0, C(5)*H*), 6.09 (1H, dd, *J* 3.7, 1.6, C(2)*H*), 6.20 (1H, dd, *J* 3.7, 2.8, C(1)*H*), 6.41 (1H, dd, *J* 2.8, 1.6, C(3)*H*), 7.15–7.22 (4H, m, C(6)ArC*H*); **^{13}C{^{1}H} NMR** (126 MHz, $CDCl_3$) δ_C: 21.2 (CH_3), 33.0 (*C*(7)H_2), 39.1 (*C*(6)H), 42.2 (morphCH_2), 45.9 (morphCH_2), 53.6 (OCH_3), 56.3 (*C*(5)H), 65.8 (morphCH_2), 66.5 (morphCH_2), 71.2 (*C*(8)), 105.8 (*C*(2)H), 110.1 (*C*(1)H), 119.5 (*C*(3)H), 128.6 (C(6)Ar*C*(3,5)H), 129.6 (C(6)Ar*C*(2,6)H), 130.4 (*C*(8a)), 135.9 (C(6)Ar*C*(4)), 138.0 (C(6)Ar*C*(1)), 167.3 (C(5)*C*=O), 175.1 (C(8)*C*=O); **HRMS** (ESI$^+$) $C_{22}H_{26}N_2O_5Na$ $[M+Na]^+$ found 421.1729, requires 421.1734 (−1.2 ppm).

Methyl (5*S*,6*S*,8*R*)-8-hydroxy-6-(3-methoxyphenyl)-5-(morpholine-4-carbonyl)-5,6,7,8-tetrahydroindolizine-8-carboxylate (40)

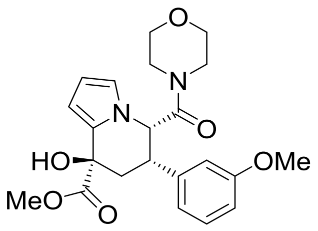

Following **General Procedure B**, 2-(1*H*-pyrrol-1-yl)acetic acid **1** (31 mg, 0.25 mmol, 1 eq.), *i*-Pr_2NEt (87 μL, 0.5 mmol), pivaloyl chloride (62 μL, 0.5 mmol) in anhydrous MeCN (2.5 mL) at 0 °C for 20 min followed by HyperBTM **11** (7.7 mg, 10 mol%), α-keto-β,γ-unsaturated ester **S16** (55 mg, 0.25 mmol, 1 eq.) and *i*-Pr_2NEt (110 μL, 0.63 mmol) at –40 °C for 24 h. Ring-opening with morpholine (2 eq.) at rt for 24 h gave crude product (> 95:5 dr) that was purified by column chromatography (20:80 EtOAc/petrol) to give the titled compound as a light yellow oil (65 mg, 63%).

$[\alpha]_D^{20}$ +84.5 (*c* 1.3 in $CHCl_3$); **Chiral HPLC analysis**, Chiralpak AD-H (90:10 hexane/IPA, flow rate 1 $mLmin^{-1}$, 211 nm, 30 °C) t_R (major): 46.8 min, t_R (minor): 68.2 min, > 99:1 er; **IR** ν_{max} (film, cm^{-1}) 3416 (O-H stretch), 2955 (C-H), 2857 (C-H), 1732 (C=O), 1645 (amide C=O); **^{1}H NMR** (500 MHz, $CDCl_3$) δ_H: 2.01 (1H, dd, *J* 13.1, 2.6, C(7)H^AH^B), 2.57–2.64 (2H, m, morphC*H*), 3.17–3.22 (1H, m, morphC*H*), 3.28–3.37 (3H, m, morphC*H*), 3.49–3.53 (3H, m, O*H* + morphC*H*), 3.68 (1H, app td, *J* 13.3, 1.3, C(7)H^AH^B), 3.81 (3H, s, OCH_3), 3.89 (3H, s, OCH_3), 4.07 (1H, ddd, *J* 13.5, 6.0, 2.6, C(6)*H*), 5.33 (1H, d, *J* 6.0, C(5)*H*), 6.09 (1H, dd, *J* 3.7, 1.6, C(2)*H*), 6.20 (1H, dd, *J* 3.7, 2.8, C(1)*H*), 6.41 (1H, dd, *J* 2.8, 1.6, C(3)*H*), 6.85–6.87 (2H, m, C(6)ArC(4,5)*H*), 6.91–6.93 (1H, m, C(6)ArC(6)*H*), 7.26–7.29 (1H, m, C(6)ArC(2)*H*); **^{13}C{^{1}H} NMR** (126 MHz, $CDCl_3$) δ_C: 32.9 (*C*(7)H_2), 39.6 (*C*(6)H), 42.2 (morphCH_2), 46.0 (morphCH_2), 53.6 (OCH_3), 55.6 (OCH_3), 56.2 (*C*(5)H), 65.9 (morphCH_2), 66.5 (morphCH_2), 71.2 (*C*(8)), 105.9 (*C*(2)H), 110.1 (*C*(1)H), 113.1 (C(6)Ar*C*H), 114.9 (C(6)Ar*C*H), 119.6 (*C*(3)H), 121.0 (C(6)Ar*C*H), 130.0 (C(6)Ar*C*H), 130.4 (*C*(8a)), 140.6 (C(6)Ar*C*(1)), 160.3 (C(6)Ar*C*(3)), 167.2 (C(5)*C*=O), 175.0 (C(8)*C*=O); **HRMS** (ESI^+) $C_{22}H_{26}N_2O_6Na$ $[M+Na]^+$ found 437.1670, requires 437.1683 (−3.0 ppm).

Methyl (5*S*,6*S*,8*R*)-8-hydroxy-5-(morpholine-4-carbonyl)-6-(4-(trifluoromethyl)phenyl)-5,6,7,8-tetrahydroindolizine-8-carboxylate (41)

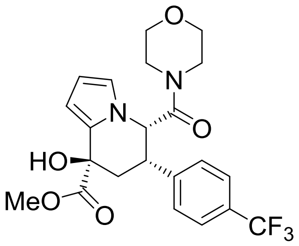

Following **General Procedure B**, 2-(1*H*-pyrrol-1-yl)acetic acid **1** (31 mg, 0.25 mmol, 1 eq.), *i*-Pr_2NEt (87 μL, 0.5 mmol), pivaloyl chloride (62 μL, 0.5 mmol) in anhydrous MeCN (2.5 mL) at 0 °C for 20 min followed by HyperBTM **11** (7.7 mg, 10 mol%), α-keto-β,γ-unsaturated ester **S17** (65 mg, 0.25 mmol, 1 eq.) and *i*-Pr_2NEt (110 μL, 0.63 mmol) at –40 °C for 24 h. Ring-opening with morpholine (2 eq.) at rt for 24 h gave crude product (> 95:5 dr) that was purified by column chromatography (20:80 EtOAc/petrol) to give the title compound as a light yellow oil (62 mg, 55%).

$[\alpha]_D^{20}$ +98.4 (*c* 1.1 in $CHCl_3$); **Chiral HPLC analysis**, Chiralpak AD-H (90:10 hexane/IPA, flow rate 1 $mLmin^{-1}$, 211 nm, 30 °C) t_R (major): 88.3 min, t_R (minor): 134.8 min, > 99:1 er; **IR** ν_{max} (film, cm^{-1}) 3387 (O-H stretch), 2961 (C-H), 2860 (C-H), 1732 (C=O), 1645 (amide C=O); **^{1}H NMR** (500 MHz, $CDCl_3$) δ_H: 2.02 (1H, dd, *J* 13.0, 2.6, C(7)H^AH^B), 2.53–2.62 (2H, m, morphC*H*), 3.18–3.29 (2H, m, morphC*H*), 3.36–3.44 (3H, m, morphC*H*), 3.50–3.54 (1H, m, morphC*H*), 3.56 (1H, s, O*H*), 3.78 (1H, app t, *J* 13.2, C(7)H^AH^B), 3.90 (3H, s, OCH_3), 4.18 (1H, ddd, *J* 13.4, 6.0, 2.6, C(6)*H*), 5.35 (1H, d, *J* 6.0, C(5)*H*), 6.11 (1H, dd, *J* 3.8, 1.6, C(2)*H*), 6.21 (1H, dd, *J* 3.7, 2.8, C(1)*H*), 6.42 (1H, dd, *J* 2.8, 1.6, C(3)*H*), 7.48 (2H, d, *J* 8.0, C(6)ArC(2,6)*H*), 7.64 (2H, d, *J* 8.1, C(6)ArC(3,5)*H*); **^{13}C{^{1}H} NMR** (126 MHz, $CDCl_3$) δ_C: 32.7 (*C*(7)H_2), 39.4 (*C*(6)H), 42.3 (morphCH_2), 46.1 (morphCH_2), 53.7 (OCH_3), 55.7 (*C*(5)H), 65.9 (morphCH_2), 66.5 (morphCH_2), 71.0 (*C*(8)), 106.1 (*C*(2)H), 110.3 (*C*(1)H), 119.6 (*C*(3)H), 123.4 (q, $^1J_{CF}$ 272.0, CF_3), 125.9 (q, $^3J_{CF}$ 4.0, C(6)Ar*C*(3,5)H), 129.2 (C(6)Ar*C*(2,6)H), 130.2 (*C*(8a)), 130.7 (q, $^2J_{CF}$ 33.1, C(6)Ar*C*(4)), 143.2 (C(6)Ar*C*(1)), 166.9 (C(5)*C*=O), 174.7 (C(8)*C*=O); **^{19}F NMR** (471 MHz, $CDCl_3$) δ_F: −62.5 (CF_3); **HRMS** (ESI^+) $C_{22}H_{23}N_2O_5F_3Na$ $[M+Na]^+$ found 475.1451, requires 475.1451 (−0.1 ppm).

Methyl (5*S*,6*S*,8*R*)-6-(4-bromophenyl)-8-hydroxy-5-(morpholine-4-carbonyl)-5,6,7,8-tetrahydroindolizine-8-carboxylate (42)

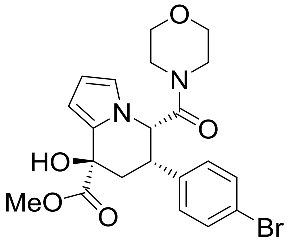

Following **General Procedure B**, 2-(1*H*-pyrrol-1-yl)acetic acid **1** (31 mg, 0.25 mmol, 1 eq.), *i*-Pr_2NEt (87 μL, 0.5 mmol), pivaloyl chloride (62 μL, 0.5 mmol) in anhydrous MeCN (2.5 mL) at 0 °C for 20 min followed by HyperBTM **11** (7.7 mg, 10 mol%), α-keto-β,γ-unsaturated ester **S18** (67 mg, 0.25 mmol, 1 eq.) and *i*-Pr_2NEt (110 μL, 0.63 mmol) at –40 °C for 24 h. Ring-opening with morpholine (2 eq.) at rt for 24 h gave crude product (> 95:5 dr) that was purified by column chromatography (20:80 EtOAc/petrol) to give the title compound as a light yellow oil (70 mg, 60%).

$[\alpha]_D^{20}$ +80.2 (*c* 1.4 in $CHCl_3$); **Chiral HPLC analysis**, Chiralpak AD-H (80:20 hexane/IPA, flow rate 1 mLmin^{-1}, 220 nm, 30 °C) t_R (major): 41.5 min, t_R (minor): 63.8 min, > 99:1 er; **IR** ν_{max} (film, cm^{-1}) 3406 (O-H stretch), 2955 (C-H), 2859 (C-H), 1734 (C=O), 1647 (amide C=O); **^{1}H NMR** (500 MHz, $CDCl_3$) δ_H: 1.98 (1H, dd, *J* 13.0, 2.6, C(7)H^AH^B), 2.58 (1H, ddd, *J* 13.2, 6.5, 3.0, morphC*H*), 2.74 (1H, ddd, *J* 11.5, 6.8, 3.0, morphC*H*), 3.22 (1H, ddd, *J* 13.1, 6.8, 3.1, morphC*H*), 3.34–3.43 (4H, m, morphC*H*), 3.51–3.53 (1H, m, morphC*H*), 3.54 (1H, d, *J* 1.4, O*H*), 3.69 (1H, app td, *J* 13.3, 1.4, C(7)H^AH^B), 3.89 (3H, s, OCH_3), 4.06 (1H, ddd, *J* 13.5, 6.0, 2.6, C(6)*H*), 5.31 (1H, d, *J* 6.0, C(5)*H*), 6.10 (1H, dd, *J* 3.7, 1.6, C(2)*H*), 6.20 (1H, dd, *J* 3.7, 2.8, C(1)*H*), 6.41 (1H, dd, *J* 2.8, 1.6, C(3)*H*), 7.21–7.23 (2H, m, C(6)ArC(2,6)*H*), 7.49–7.52 (2H, m, C(6)ArC(3,5)*H*); **^{13}C{^{1}H} NMR** (126 MHz, $CDCl_3$) δ_C: 32.8 ($C(7)H_2$), 39.0 (*C*(6)H), 42.2 (morphCH_2), 46.1 (morphCH_2), 53.7 (OCH_3), 55.8 (*C*(5)H), 65.9 (morphCH_2), 66.6 (morphCH_2), 71.0 (*C*(8)), 106.0 (*C*(2)H), 110.3 (*C*(3)H), 119.6 (*C*(1)H), 122.2 (C(6)Ar*C*(1)), 130.2 (*C*(8a)), 130.4 (C(6)Ar*C*(2,6)H), 132.1 (C(6)Ar*C*(3,5)H), 138.0 (C(6)Ar*C*(4)), 167.1 (C(5)*C*=O), 174.8 (C(8)*C*=O); **HRMS** (ESI$^+$) $C_{21}H_{23}N_2O_5{}^{79}BrNa$ $[M+Na]^+$ found 485.0675, requires 485.0683 (−1.6 ppm).

Methyl (5*S*,6*S*,8*R*)-6-(3-bromophenyl)-8-hydroxy-5-(morpholine-4-carbonyl)-5,6,7,8-tetrahydroindolizine-8-carboxylate (43)

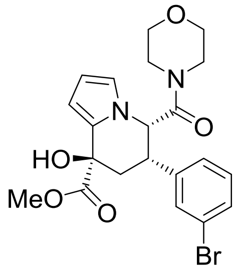

Following **General Procedure B**, 2-(1*H*-pyrrol-1-yl)acetic acid **1** (31 mg, 0.25 mmol, 1 eq.), *i*-Pr_2NEt (87 μL, 0.5 mmol), pivaloyl chloride (62 μL, 0.5 mmol) in anhydrous MeCN (2.5 mL) at 0 °C for 20 min followed by HyperBTM **11** (7.7 mg, 10 mol%), α-keto-β,γ-unsaturated ester **S19** (67 mg, 0.25 mmol, 1 eq.) and *i*-Pr_2NEt (110 μL, 0.63 mmol) at –40 °C for 24 h. Ring-opening with morpholine (2 eq.) at rt for 24 h gave crude product (> 95:5 dr) that was purified by column chromatography (20:80 EtOAc/petrol) to give the title compound as a light yellow oil (114 mg, 98%, isolated in ~95% purity due to a small amount of grease contaminant from petroleum ether).

$[\alpha]_D^{20}$ +72.3 (*c* 1.6 in $CHCl_3$); **Chiral HPLC analysis**, Chiralpak AD-H (90:10 hexane/IPA, flow rate 1 mLmin^{-1}, 211 nm, 30 °C) t_R (major): 63.3 min, t_R (minor): 87.9 min, > 99:1 er; **IR** ν_{max} (film, cm^{-1}) 3422 (O-H stretch), 2955 (C-H), 2855 (C-H), 1734 (C=O), 1647 (amide C=O); **^{1}H NMR** (500 MHz, $CDCl_3$) δ_H: 2.00 (1H, dd, *J* 13.0, 2.6, C(7)H^AH^B), 2.58 (1H, ddd, *J* 10.9, 7.5, 3.0, morphC*H*), 2.64 (1H, ddd, *J* 13.1, 5.7, 3.0, morphC*H*), 3.22 (1H, ddd, *J* 13.1, 7.5, 3.1, morphC*H*), 3.31–3.41 (3H, m, morphC*H*), 3.53–3.61 (3H, m, morphC*H* + O*H*), 3.69 (1H, app td, *J* 13.3, 1.5, C(7)H^AH^B), 3.90 (3H, s, OCH_3), 4.08 (1H, ddd, *J* 13.5, 6.0, 2.6, C(6)*H*), 5.32 (1H, d, *J* 6.0, C(5)*H*), 6.10 (1H, dd, *J* 3.7, 1.6, C(2)*H*), 6.21 (1H, dd, *J* 3.7, 2.8, C(1)*H*), 6.41 (1H, dd, *J* 2.8, 1.6, C(3)*H*), 7.24–7.30 (2H, m, C(6)ArC*H*), 7.48–7.50 (2H, m, C(6)ArC*H*); **^{13}C{^{1}H} NMR** (126 MHz, $CDCl_3$) δ_C: 32.7 (*C*(7)H_2), 39.3 (*C*(6)H), 42.2 (morph*C*H_2), 46.1 (morph*C*H_2), 53.7 (O*C*H_3), 55.9 (*C*(5)H), 65.9 (morph*C*H_2), 66.6 (morph*C*H_2), 71.0 (*C*(8)), 106.0 (*C*(2)H), 110.2 (*C*(1)H), 119.6 (*C*(3)H), 123.2 (C(6)Ar*C*(1)), 127.6 (C(6)Ar*C*H), 130.2 (*C*(8a)), 130.6 (C(6)Ar*C*H), 131.4 (C(6)Ar*C*H), 131.6 (C(6)Ar*C*H), 141.4 (C(6)Ar*C*(3)), 166.9 (C(5)*C*=O), 174.8 (C(8)*C*=O); **HRMS** (ESI$^+$) $C_{21}H_{23}N_2O_5{}^{79}BrNa$ $[M+Na]^+$ found 485.0684, requires 485.0683 (+0.3 ppm).

Methyl (5*S*,6*S*,8*R*)-6-(2-bromophenyl)-8-hydroxy-5-(morpholine-4-carbonyl)-5,6,7,8-tetrahydroindolizine-8-carboxylate (44)

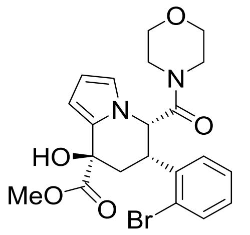

Following **General Procedure B**, 2-(1*H*-pyrrol-1-yl)acetic acid **1** (31 mg, 0.25 mmol, 1 eq.), *i*-Pr_2NEt (87 μL, 0.5 mmol), pivaloyl chloride (62 μL, 0.5 mmol) in anhydrous MeCN (2.5 mL) at 0 °C for 20 min followed by HyperBTM **11** (7.7 mg, 10 mol%), α-keto-β,γ-unsaturated ester **S20** (67 mg, 0.25 mmol, 1 eq.) and *i*-Pr_2NEt (110 μL, 0.63 mmol) at –40 °C for 24 h. Ring-opening with morpholine (2 eq.) at rt for 24 h gave crude product (> 95:5 dr) that was purified by column chromatography (20:80 EtOAc/petrol) to give the title compound as a light yellow oil (67 mg, 58%).

$[\alpha]_D^{20}$ +169 (*c* 1.4 in $CHCl_3$); **Chiral HPLC analysis**, Chiralcel OD-H (90:10 hexane/IPA, flow rate 1 mLmin^{-1}, 220 nm, 30 °C) t_R (major): 37.6 min, t_R (minor): 58.4 min, > 99:1 er; **IR** ν_{max} (film, cm^{-1}) 3424 (O-H), 2959 (C-H), 2926 (C-H), 2859 (C-H), 1717 (C=O), 1651 (C=O); **^{1}H NMR** (500 MHz, $CDCl_3$) δ_H: 1.99 (1H, dd, *J* 12.9, 2.7, C(7)H^AH^B), 2.52 (1H, ddd, *J* 11.2, 7.7, 2.9, morphC*H*), 2.78 (1H, ddd, *J* 13.3, 5.7, 2.9, morphC*H*), 3.13–3.17 (1H, m, morphC*H*), 3.24–3.31 (2H, m, morphC*H*), 3.40–3.53 (4H, m, morphC*H* + O*H*), 3.76–3.81 (1H, m, C(7)H^AH^B), 3.90 (3H, s, OCH_3), 4.51 (1H, ddd, *J* 13.4, 5.9, 2.7, C(6)*H*), 5.59 (1H, d, *J* 5.9, C(5)*H*), 6.12 (1H, dd, *J* 3.7, 1.6, C(2)*H*), 6.22 (1H, dd, *J* 3.7, 2.8, C(1)*H*), 6.44 (1H, dd, *J* 2.8, 1.6, C(3)*H*), 7.20–7.23 (1H, m, C(6)ArC*H*), 7.33–7.34 (2H, m, C(6)ArC*H*), 7.62–7.64 (1H, m, C(6)ArC*H*); **^{13}C{^{1}H} NMR** (126 MHz, $CDCl_3$) δ_C: 32.7 (*C*(7)H_2), 38.4 (*C*(6)H), 42.3 (morph*C*H_2), 46.1 (morph*C*H_2), 53.1 (O*C*H_3), 53.6 (*C*(5)H), 66.2 (morph*C*H_2), 66.6 (morph*C*H_2), 71.1 (*C*(8)), 106.0 (*C*(2)H), 110.2 (*C*(1)H), 119.8 (*C*(3)H), 125.8 (C(6)Ar*C*(5)H), 128.3 (C(6)Ar*C*(6)H), 129.7 (C(6)Ar*C*(2)), 129.8 (C(6)Ar*C*(4)H), 130.2 (*C*(8a)), 133.2 (C(6)Ar*C*(3)H), 138.1 (C(6)Ar*C*(1)), 167.0 (C(5)*C*=O), 174.8 (C(8)*C*=O); **HRMS** (ESI$^+$) $C_{21}H_{23}N_2O_5{}^{79}BrNa$ $[M+Na]^+$ found 485.0681, requires 485.0683 (–0.3 ppm).

Methyl (5*S*,6*R*,8*R*)-6-(furan-2-yl)-8-hydroxy-5-(morpholine-4-carbonyl)-5,6,7,8-tetrahydroindolizine-8-carboxylate (45)

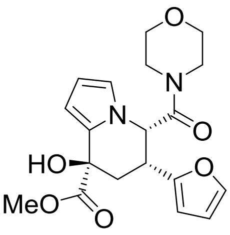

Following **General Procedure B**, 2-(1*H*-pyrrol-1-yl)acetic acid **1** (31 mg, 0.25 mmol, 1 eq.), *i*-Pr_2NEt (87 μL, 0.5 mmol), pivaloyl chloride (62 μL, 0.5 mmol) in anhydrous MeCN (2.5 mL) at 0 °C for 20 min followed by HyperBTM **11** (7.7 mg, 10 mol%), α-keto-β,γ-unsaturated ester **S21** (45 mg, 0.25 mmol, 1 eq.) and *i*-Pr_2NEt (110 μL, 0.63 mmol) at –40 °C for 24 h. Ring-opening with morpholine (2 eq.) at rt for 24 h gave crude product (> 95:5 dr) that was purified by column chromatography (20:80 EtOAc/petrol) to give the title compound as a light yellow oil (58 mg, 62%).

$[\alpha]_D^{20}$ +83.2 (*c* 1.3 in $CHCl_3$); **Chiral HPLC analysis**, Chiralpak AD-H (90:10 hexane/IPA, flow rate 1 mLmin^{-1}, 220 nm, 30 °C) t_R (major): 89.3 min, t_R (minor): 53.1 min, > 99:1 er; **IR** ν_{max} (film, cm^{-1}) 3410 (O-H stretch), 2955 (C-H), 2857 (C-H), 1734 (C=O), 1649 (amide C=O); **^{1}H NMR** (500 MHz, $CDCl_3$) δ_H: 2.08 (1H, dd, *J* 13.2, 2.7, C(7)H^AH^B), 3.03 (1H, ddd, *J* 13.2, 6.6, 3.0, morphC*H*), 3.23 (1H, ddd, *J* 11.6, 6.7, 3.0, morphC*H*), 3.43–3.57 (8H, m, morphC*H* + C(7)H^AH^B + O*H*), 3.88 (3H, s, OCH_3), 4.17 (1H, ddd, *J* 13.5, 5.9, 2.7, C(6)*H*), 5.49 (1H, d, *J* 5.9, C(5)*H*), 6.10 (1H, dd, *J* 3.7, 1.6, C(2)*H*), 6.19–6.22 (2H, m, C(6)ArC*H* + C(1)*H*), 6.39 (1H, dd, *J* 3.4, 1.9, C(6)ArC(3)*H*), 6.44 (1H, dd, *J* 2.8, 1.6, C(3)*H*), 7.40 (1H, dd, *J* 1.9, 0.8, C(6)ArC*H*); **^{13}C{^{1}H} NMR** (126 MHz, $CDCl_3$) δ_C: 31.7 (*C*(7)H_2), 33.5 (*C*(6)H), 42.4 (morph*C*H_2), 45.9 (morph*C*H_2), 53.6 (O*C*H_3), 54.6 (*C*(5)H), 66.5 (morph*C*H_2), 66.8 (morph*C*H_2), 70.8 (*C*(8)), 106.1 (*C*(2)H), 107.5 (C(6)Ar*C*H), 110.2 (*C*(1)H), 111.2 (C(6)Ar*C*H), 119.8 (*C*(3)H), 130.4 (*C*(8a)), 142.1 (C(6)Ar*C*H), 153.3 (C(6)Ar*C*(1)), 167.2 (C(5)*C*=O), 174.7 (C(8)*C*=O); **HRMS** (ESI$^+$) $C_{19}H_{22}N_2O_6Na$ $[M+Na]^+$ found 397.1364, requires 397.1370 (−1.5 ppm).

X-Ray Data and Stereochemical Rationale

X-ray diffraction data were collected at 293 K using a Rigaku MM-007HF High Brilliance RA generator/confocal optics with XtaLAB P200 diffractometer [Cu Kα radiation (λ = 1.54187 Å)]. Data were collected using CrystalClear[11] and processed (including correction for Lorentz, polarization and absorption) using CrysAlisPro.[12] Structures were solved by dual-space (SHELXT[13]), direct (SIR2011[14]) or charge-flipping (Superflip[15]) methods and refined by full-matrix least-squares against F^2 (SHELXL-2018/3[16]). Non-hydrogen atoms were refined anisotropically, and all hydrogen atoms were refined using a riding model. All calculations were performed using the CrystalStructure[17] interface.

	**(5*S*,6*S*,8*R*)-21**
CCDC	1978281
empirical formula	$C_{18}H_{18}F_3NO_4$
fw	369.34
crystal description	colourless prism
crystal size [mm]	0.02×0.03×0.02
space group	$P2_12_12_1$ (#19)
a [Å]	10.18600(6)
b [Å]	11.35580(7)
c [Å]	15.52960(10)
vol [Å]^3	1796.312(19)
Z	4
ρ (calc) [g/cm^3]	1.366
μ [mm^{-1}]	1.007
F(000)	768
reflections collected	20749
independent reflections (R_{int})	3627 (0.0181)
data/parameters	3627/240
GOF on F^2	1.06
R_1 [$I > 2\sigma(I)$]	0.0371
wR_2 (all data)	0.1020
largest diff. peak/hole [e/Å^3]	0.17, −0.16
Flack parameter	0.02(5)

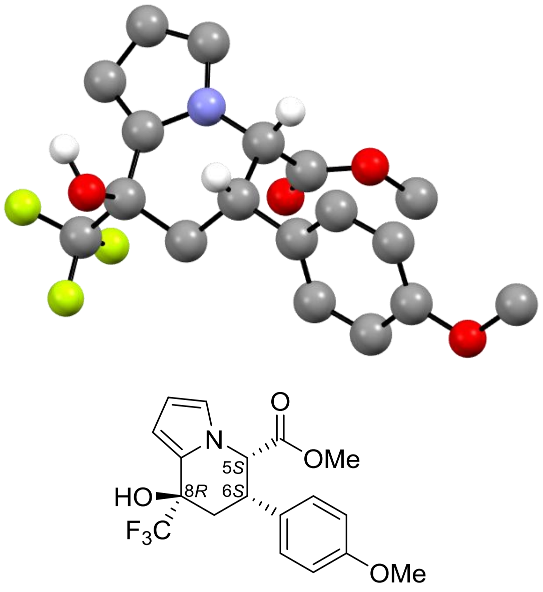

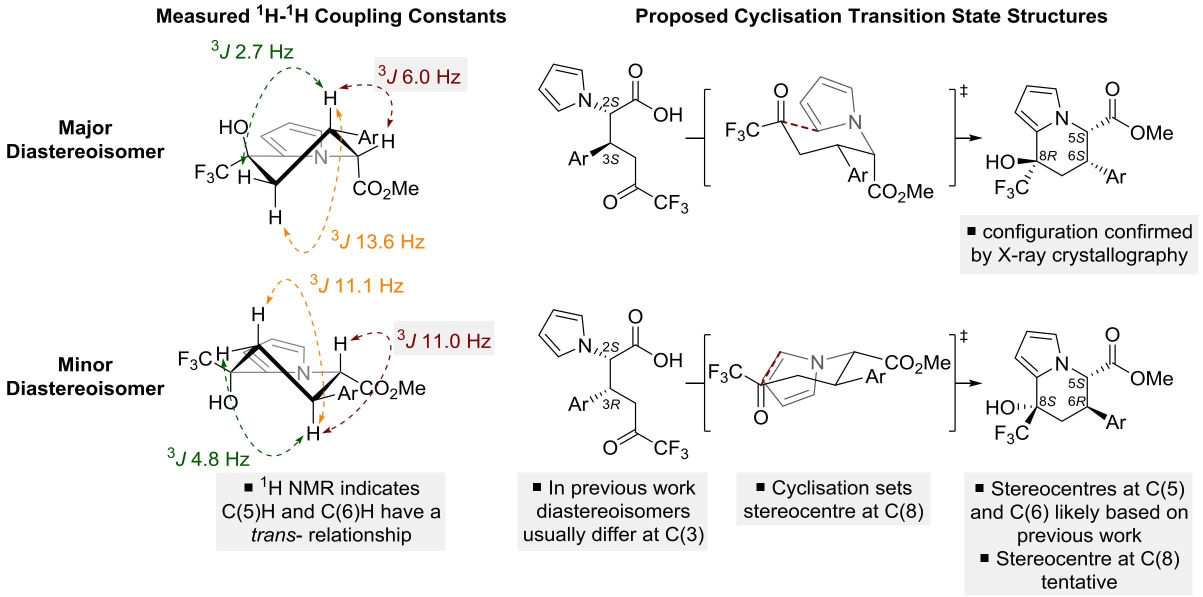

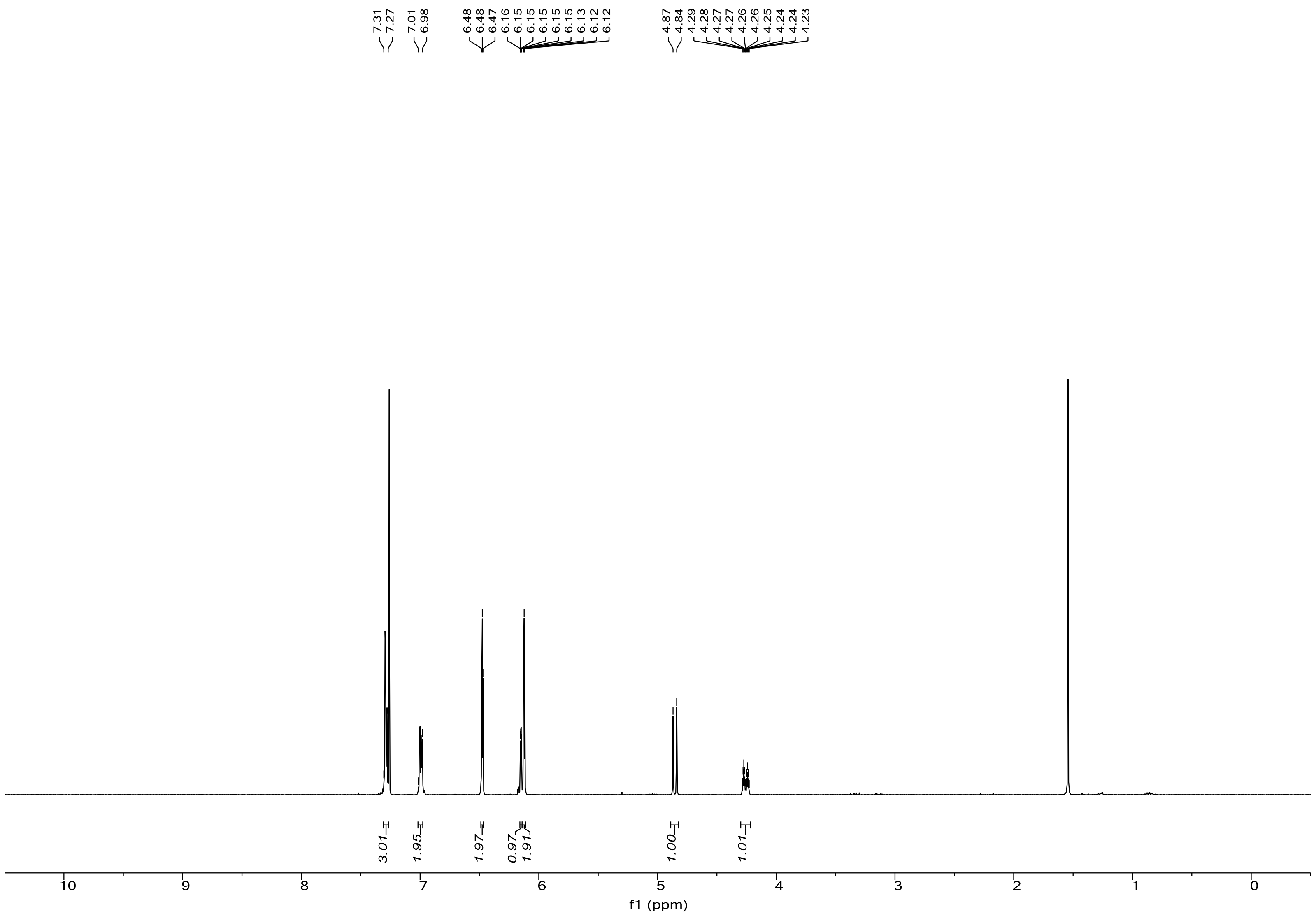

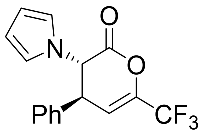

8

^{1}H NMR (400 MHz, $CDCl_3$)

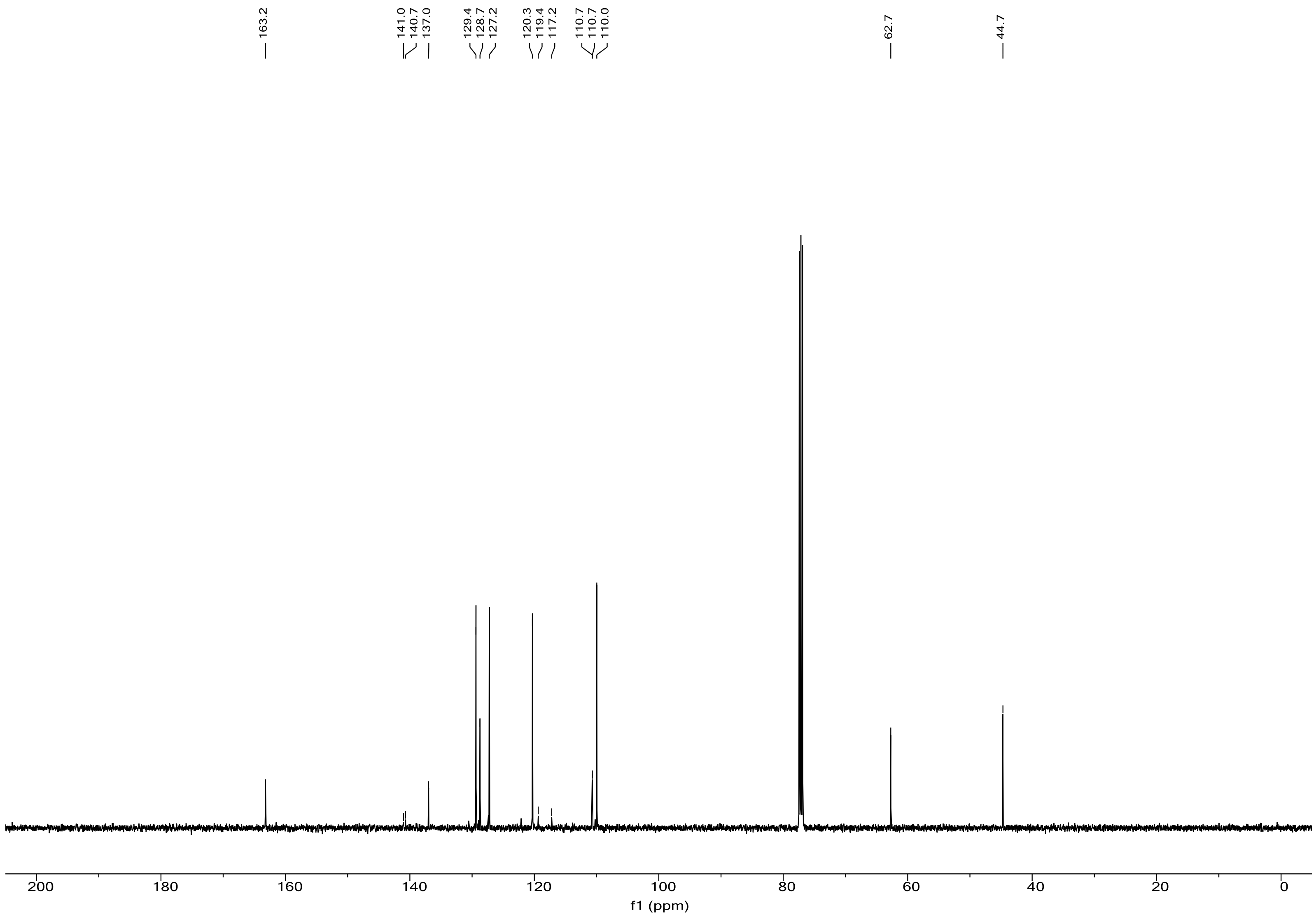

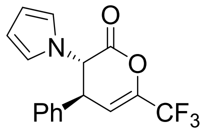

8

$^{13}C\{^{1}H\}$ NMR (126 MHz, $CDCl_3$)

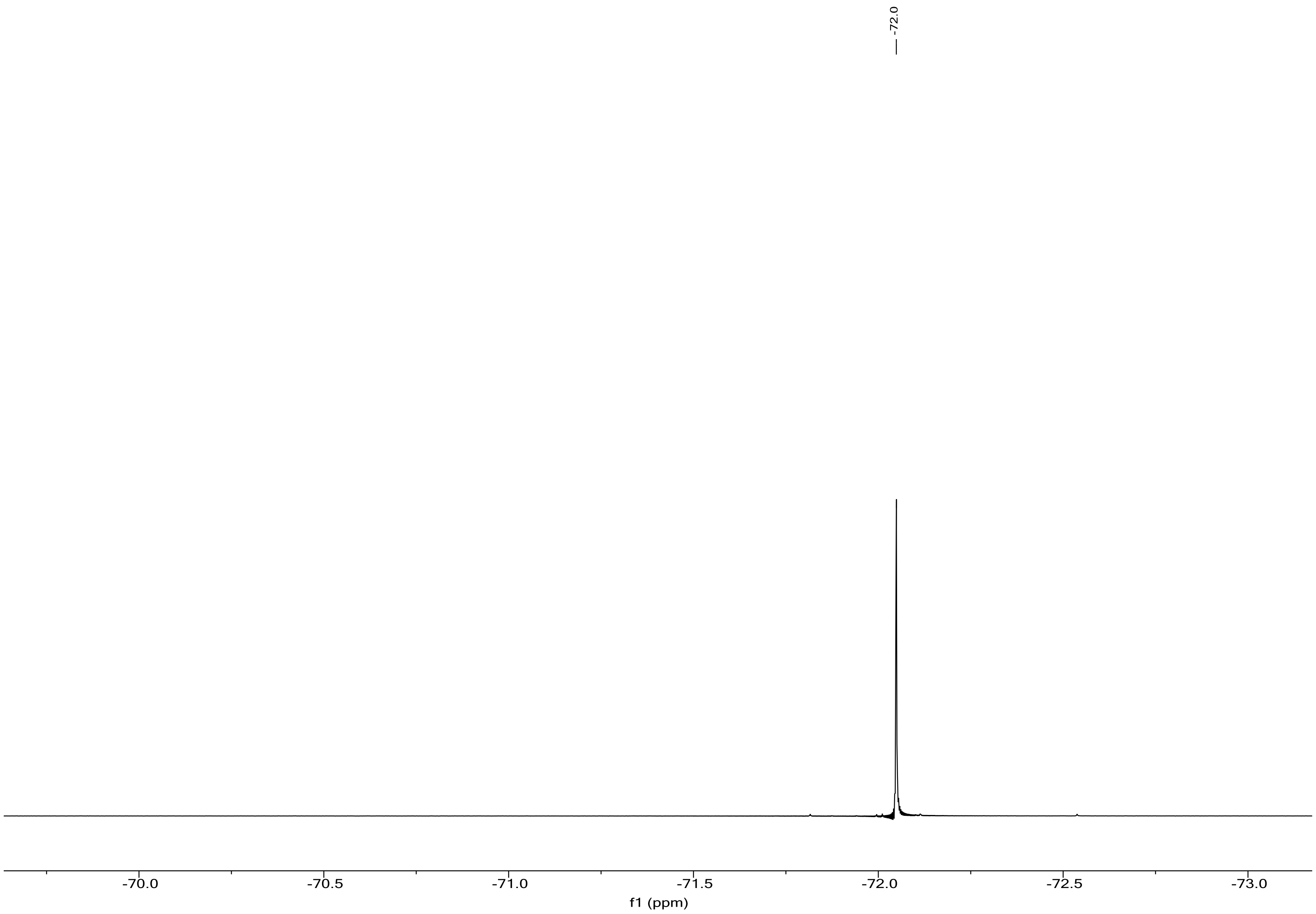

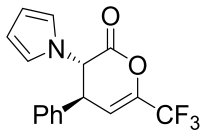

8

$^{19}F\{^{1}H\}$ NMR (377 MHz, $CDCl_3$)

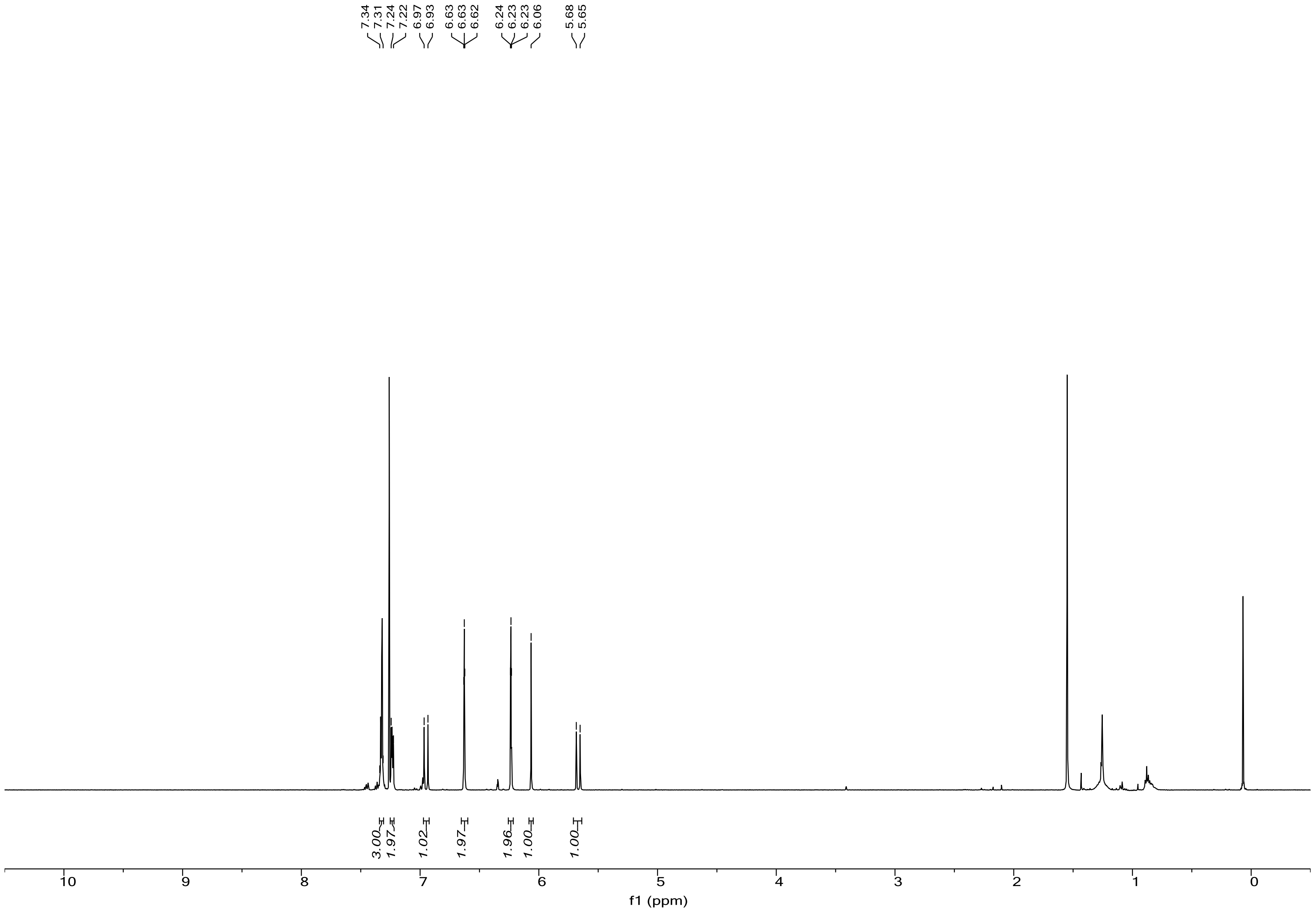

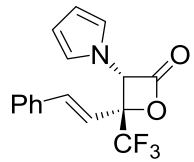

9

^{1}H NMR (500 MHz, $CDCl_3$)

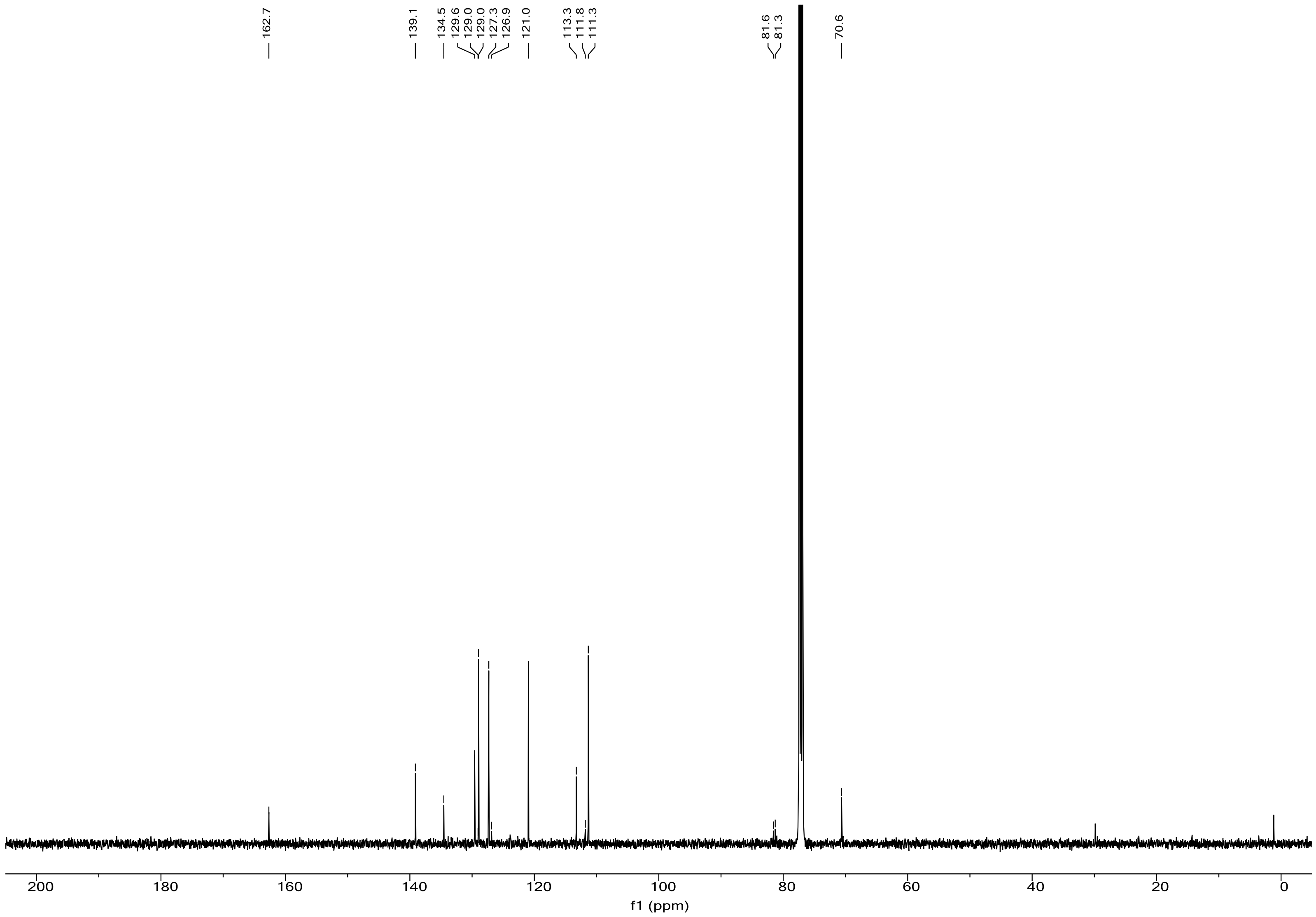

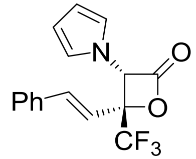

9

$^{13}C\{^{1}H\}$ NMR (126 MHz, $CDCl_3$)

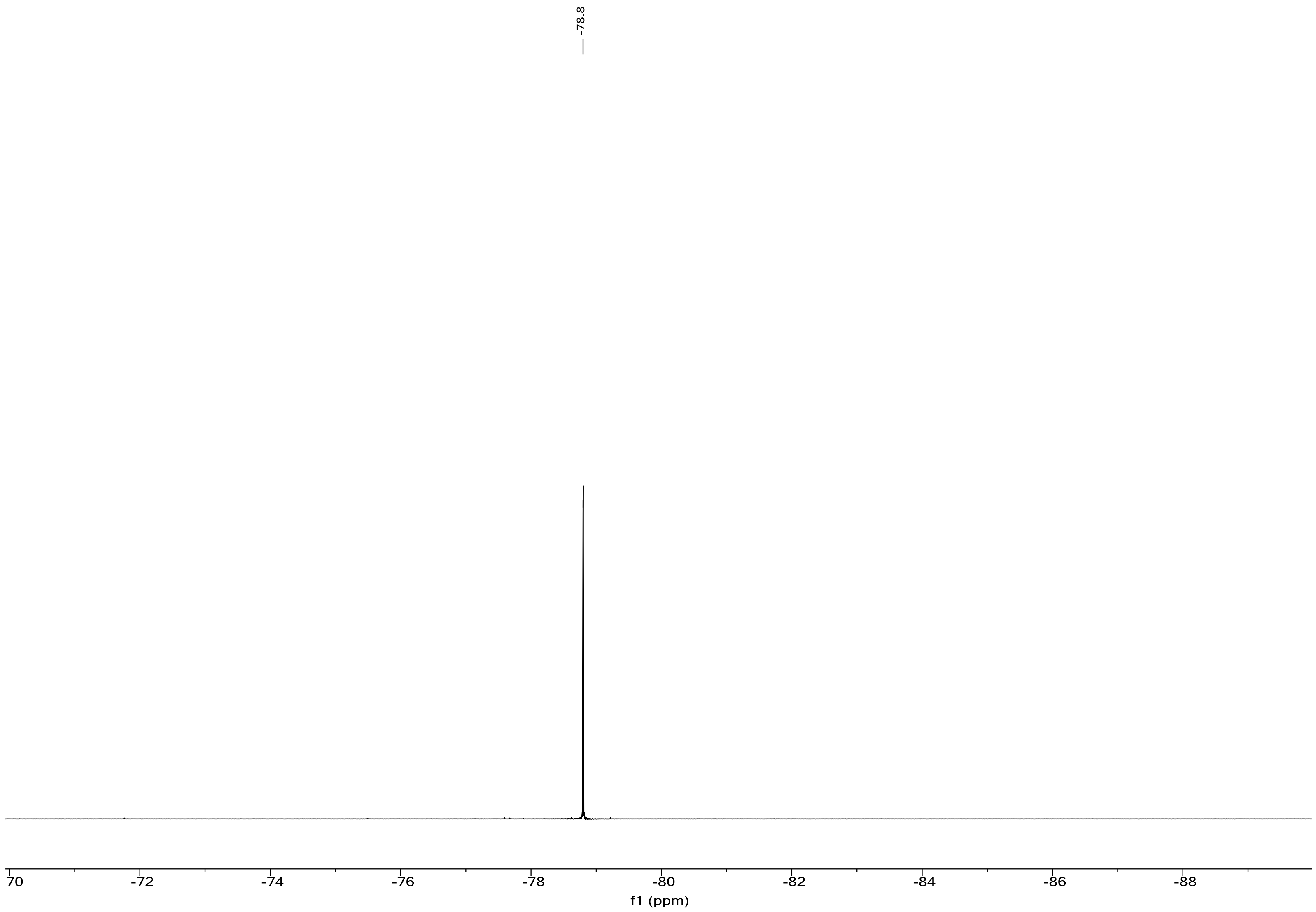

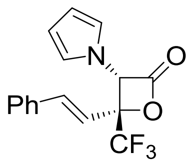

9

^{19}F NMR (471 MHz, $CDCl_3$)

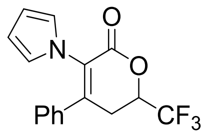

10

^{1}H NMR (400 MHz, $CDCl_3$)

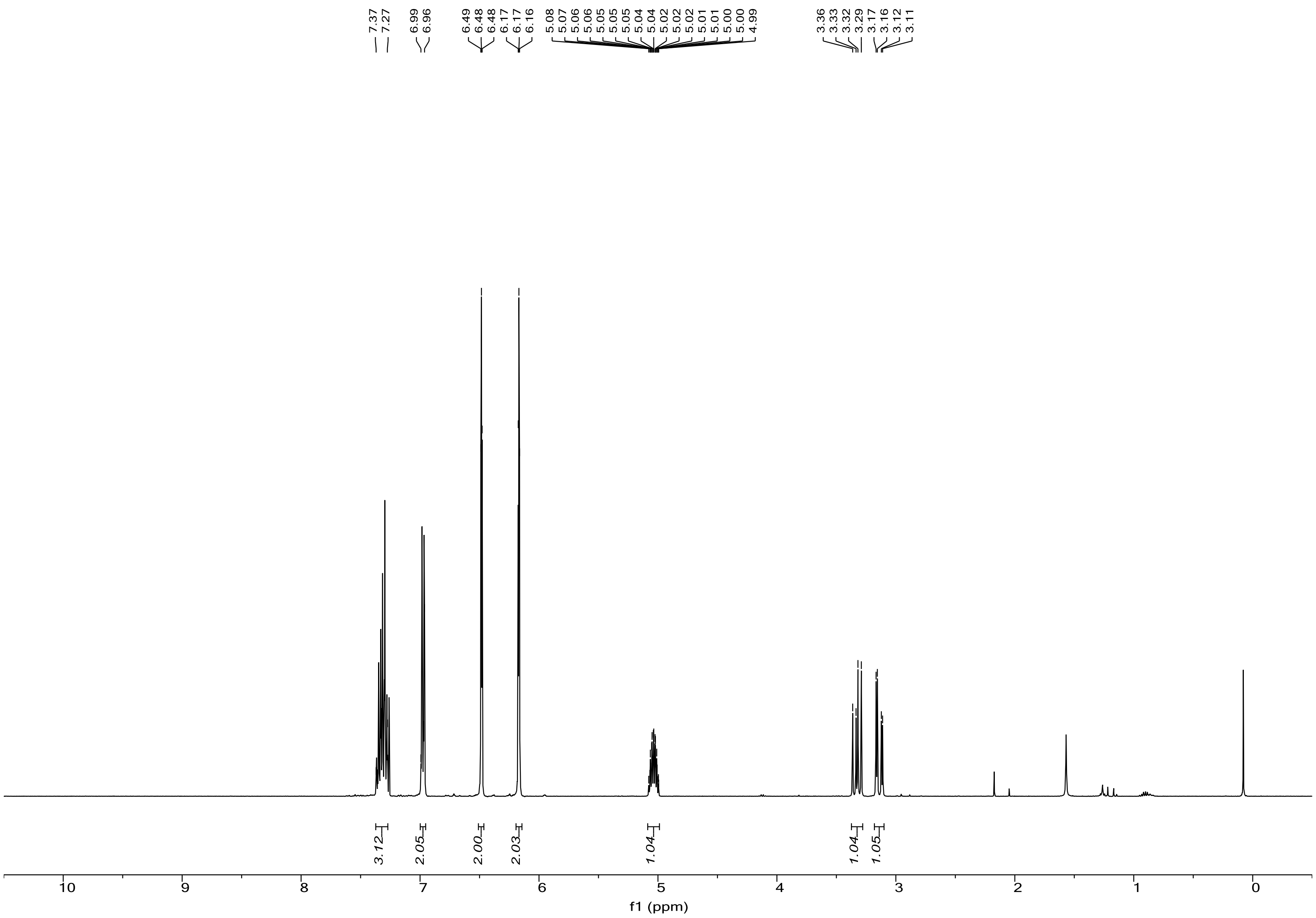

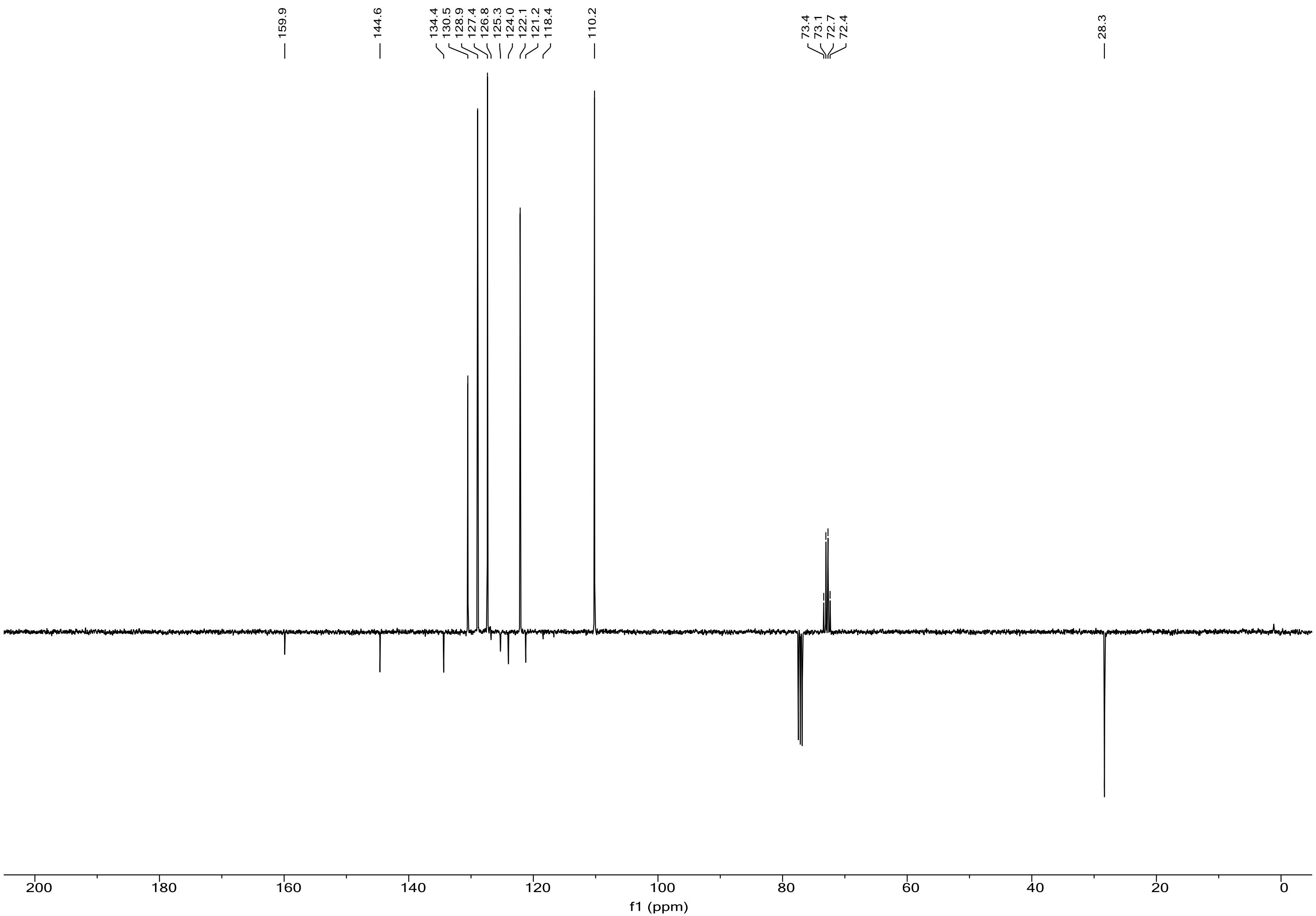

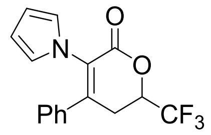

10

$^{13}C\{^{1}H\}$ NMR (101 MHz, $CDCl_3$)

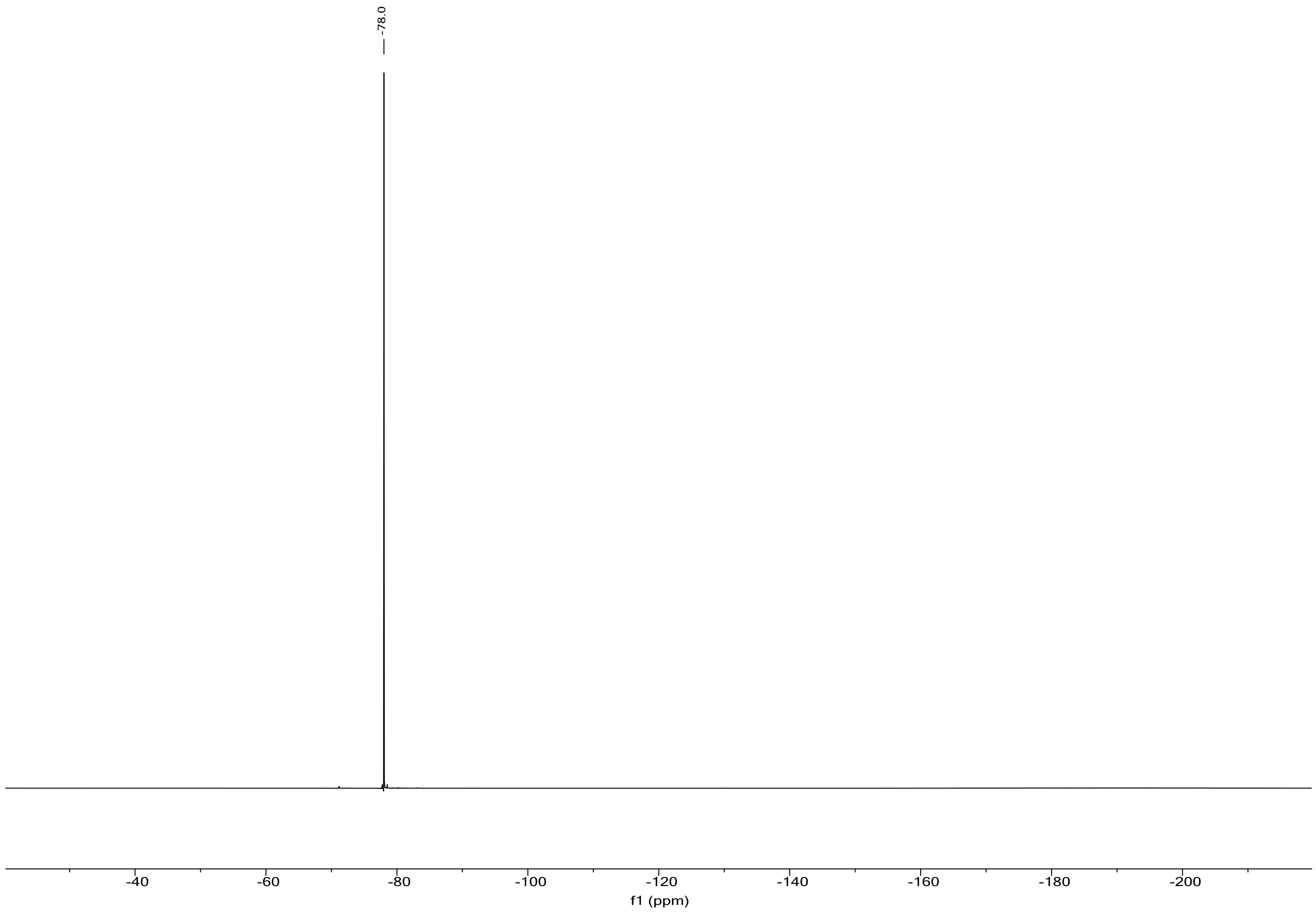

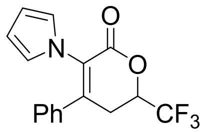

10

^{19}F{^{1}H} NMR (377 MHz, $CDCl_3$)

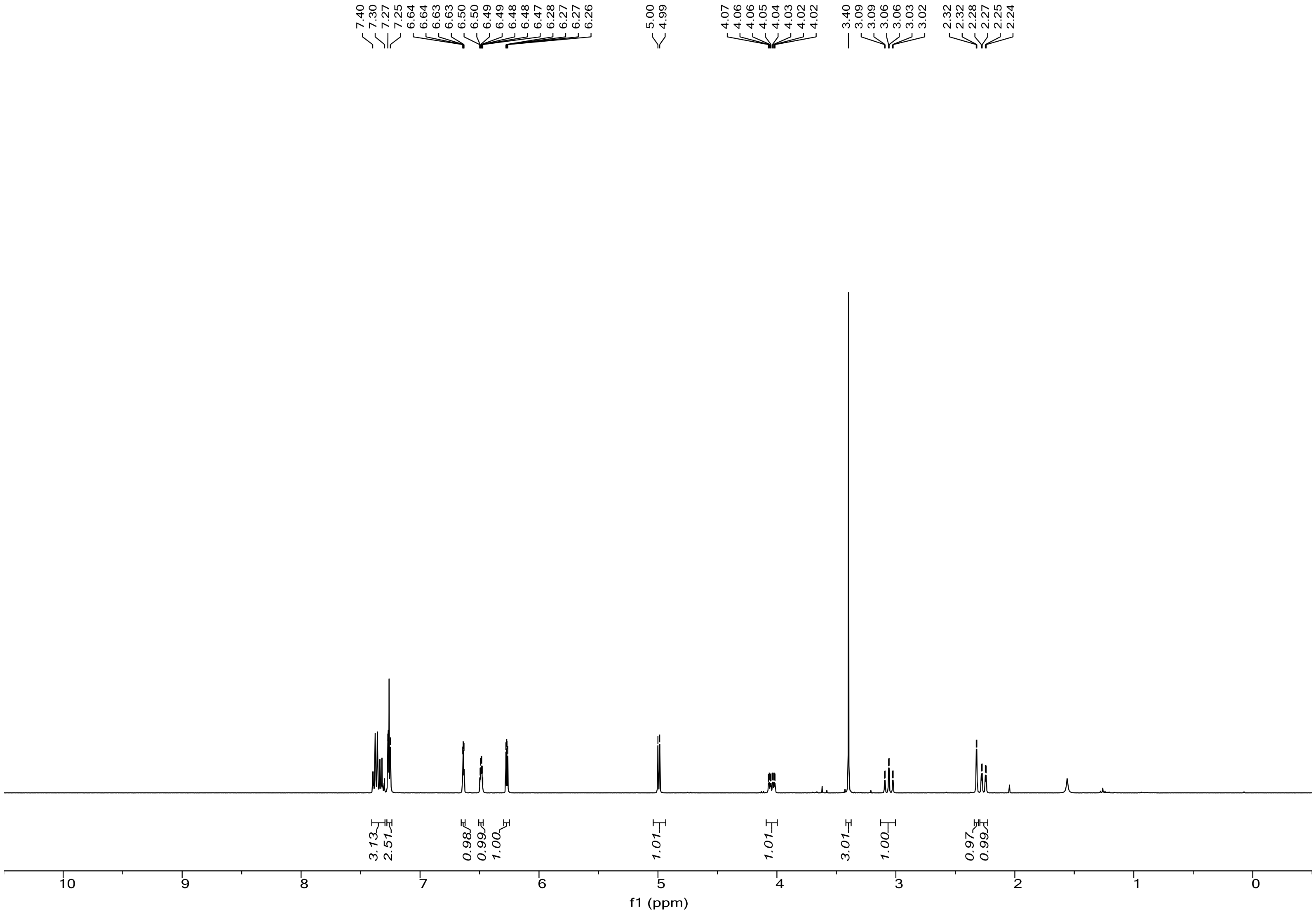

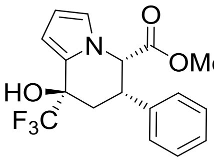

14

^{1}H NMR (400 MHz, $CDCl_3$)

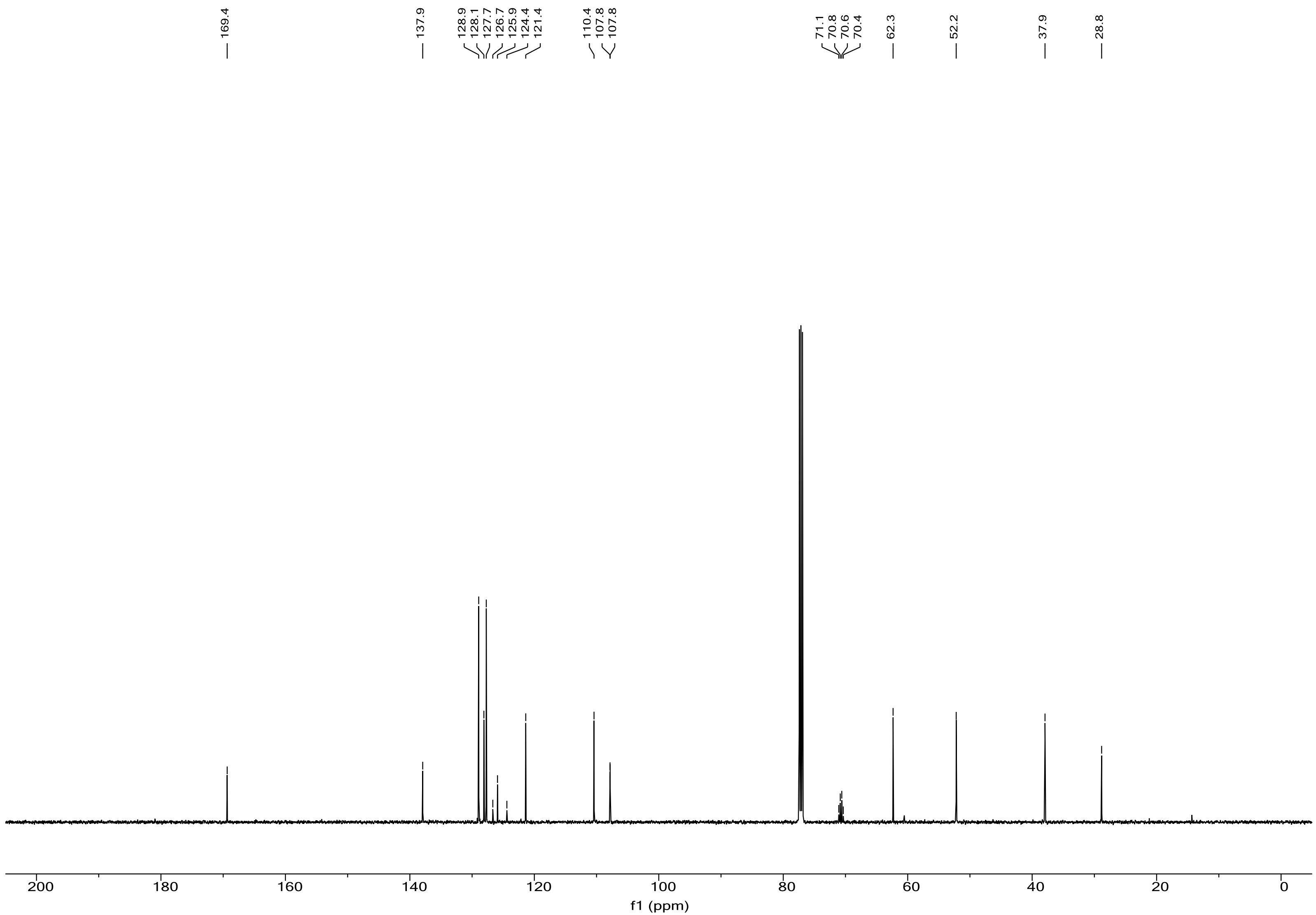

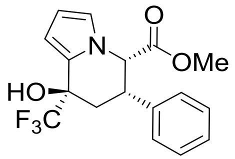

14

$^{13}C\{^{1}H\}$ NMR (126 MHz, $CDCl_3$)

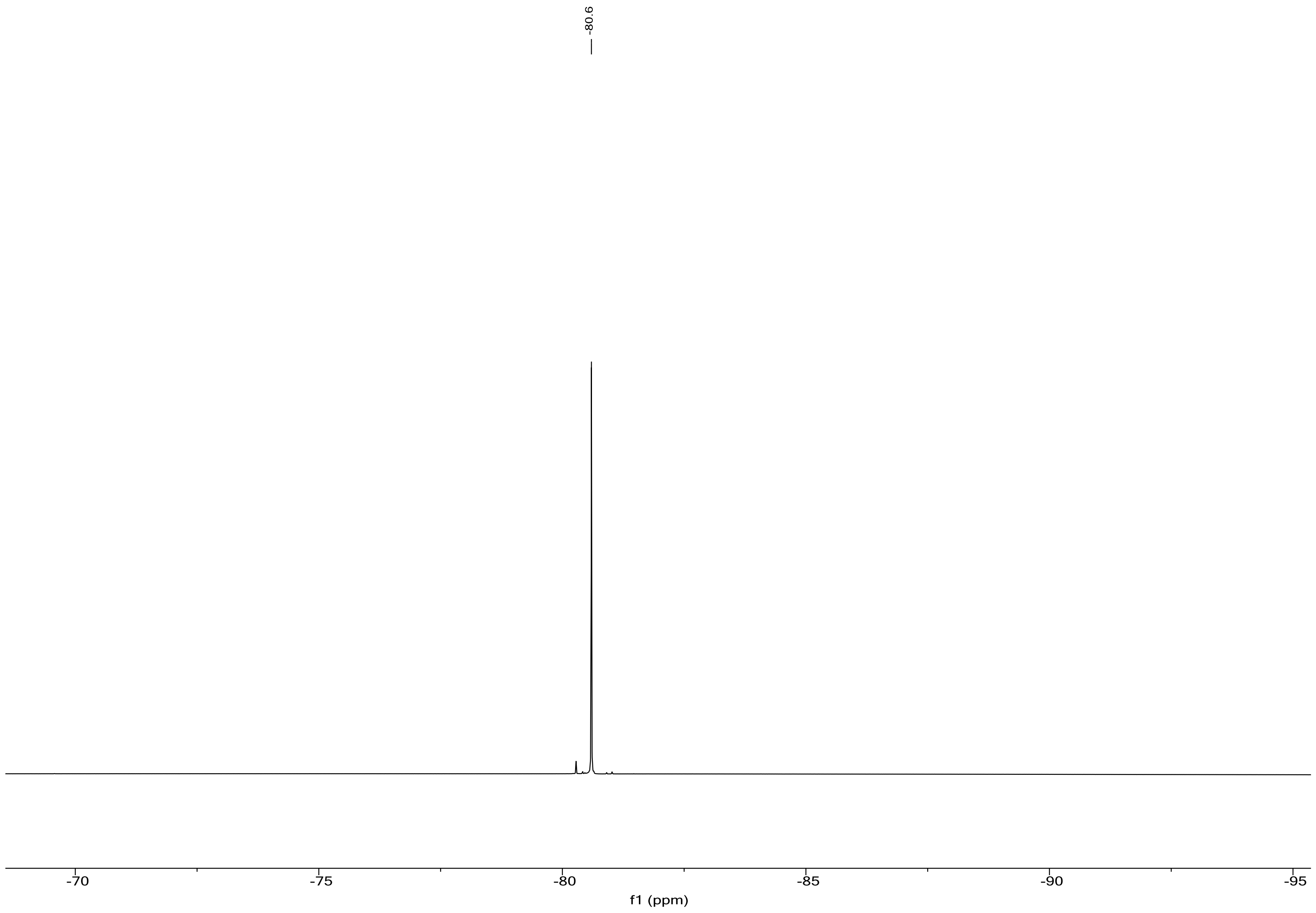

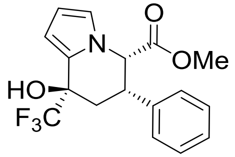

14

$^{19}F\{^1H\}$ NMR (377 MHz, $CDCl_3$)

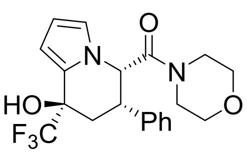

16

^{1}H NMR (400 MHz, $CDCl_3$)

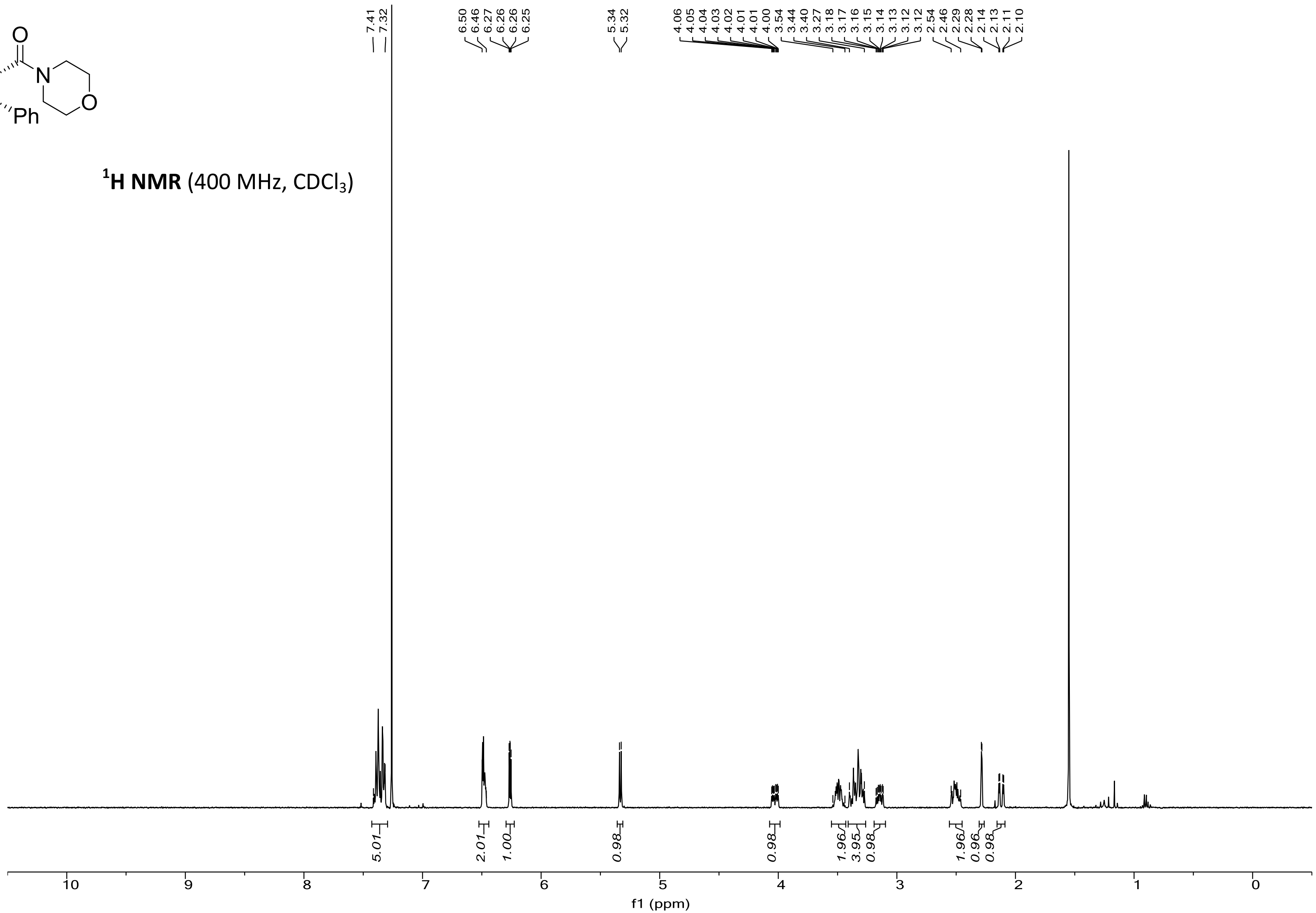

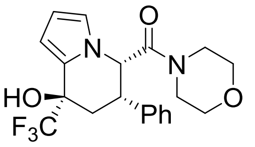

16

^{13}**C{^{1}H} NMR** (126 MHz, $CDCl_3$)

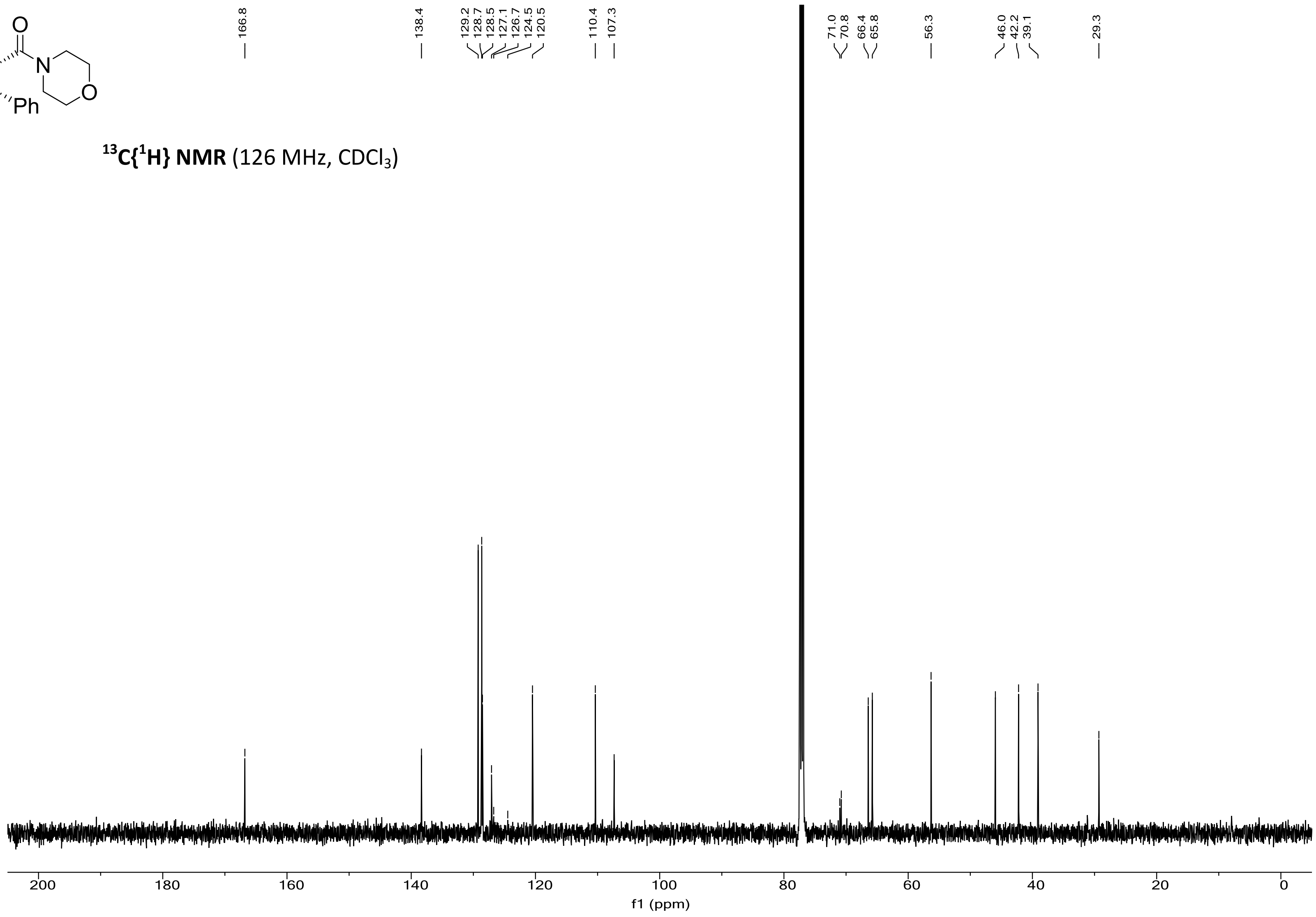

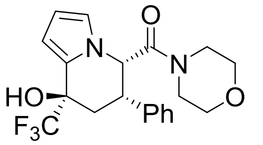

16

^{19}F NMR (471 MHz, $CDCl_3$)

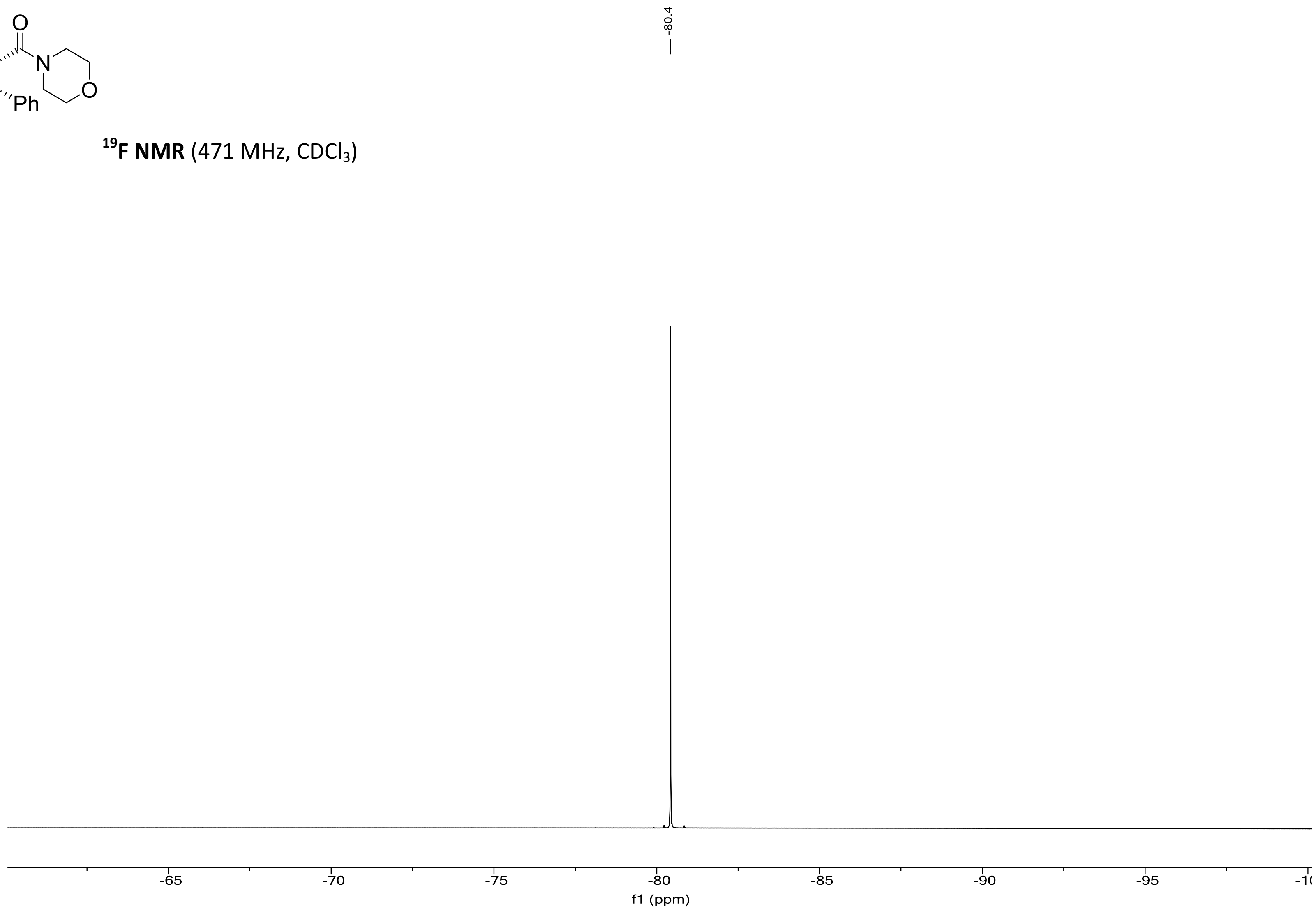

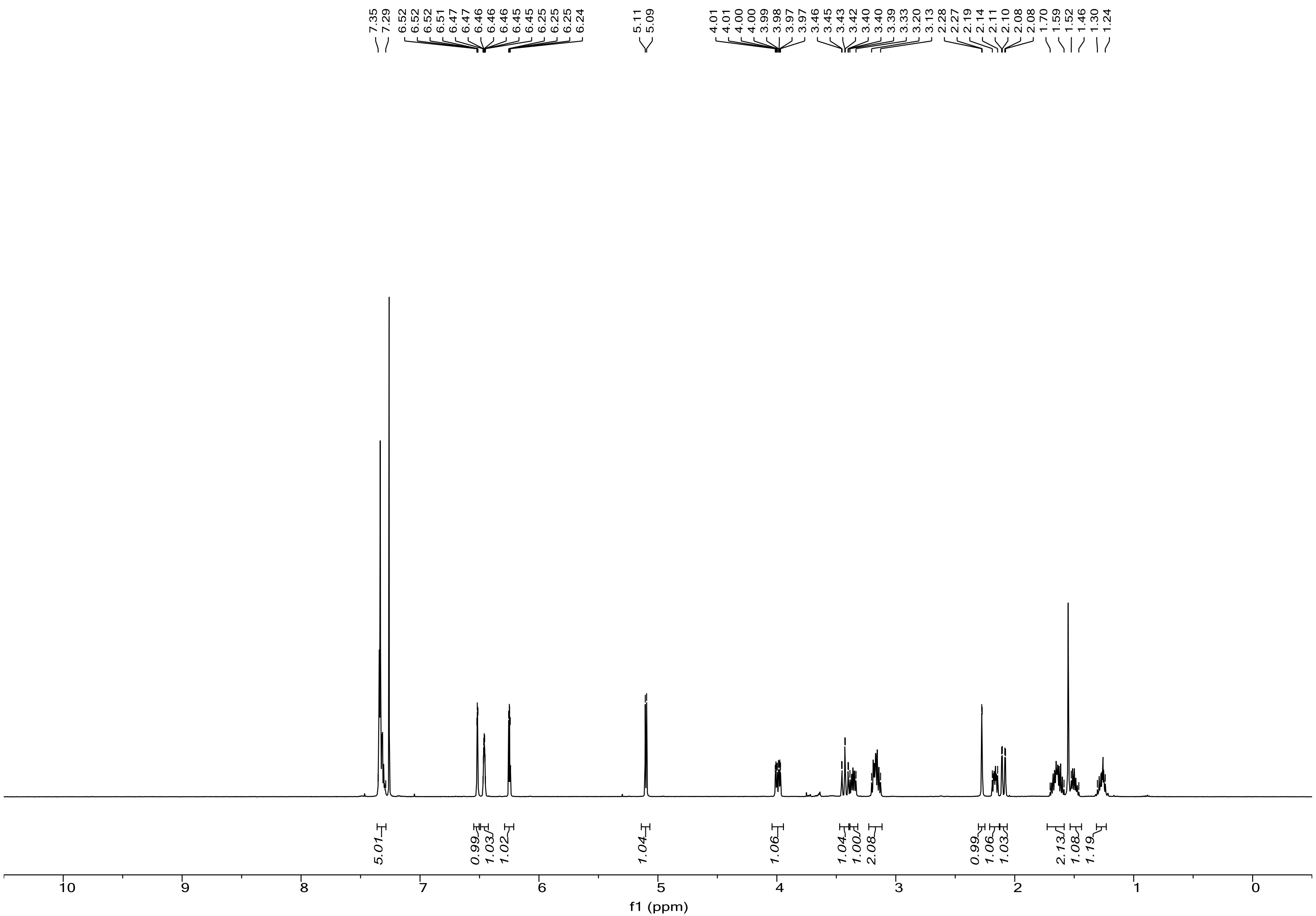

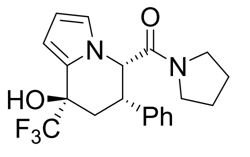

17

^{1}H NMR (500 MHz, $CDCl_3$)

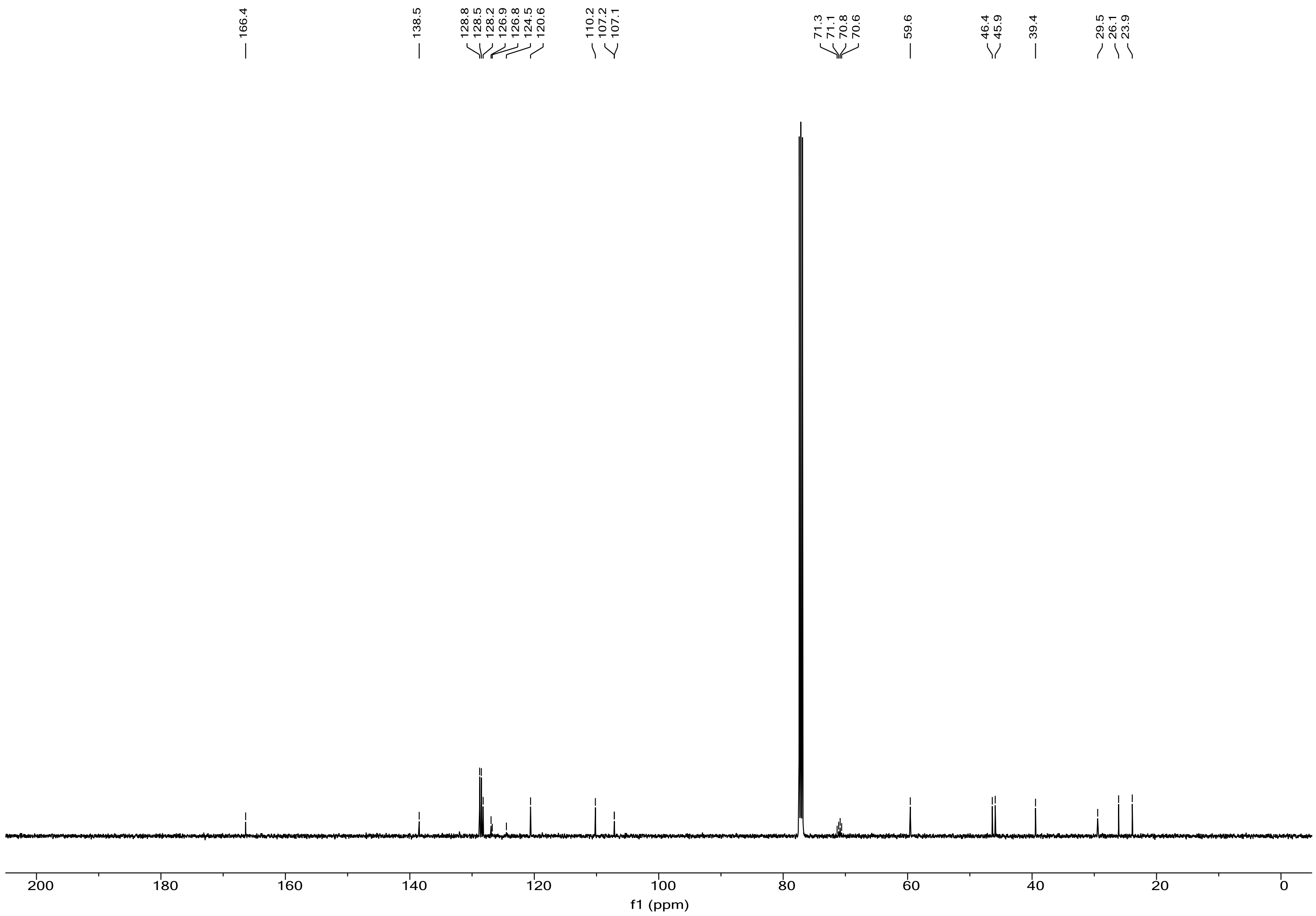

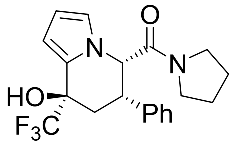

17

$^{13}C\{^{1}H\}$ NMR (126 MHz, $CDCl_3$)

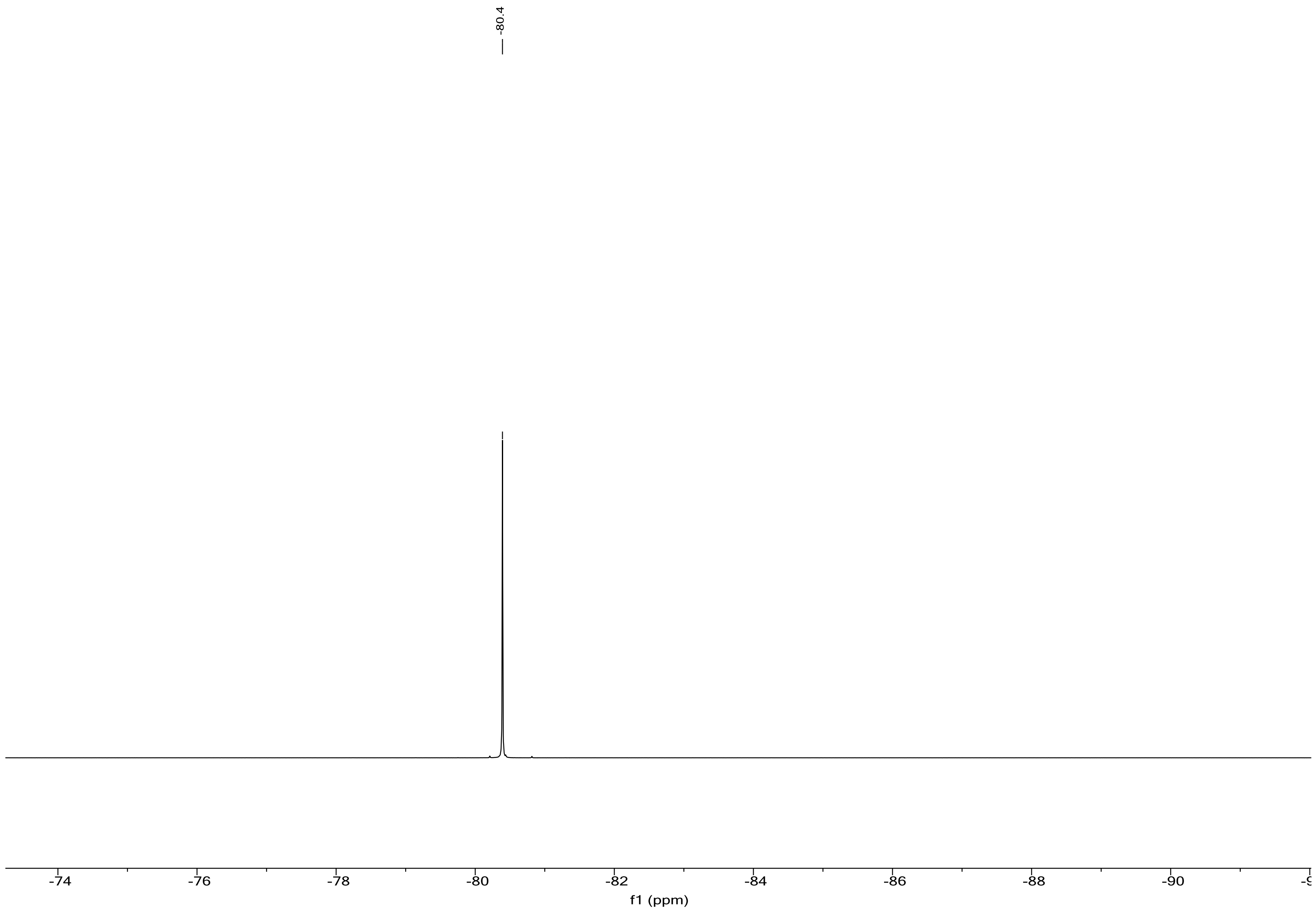

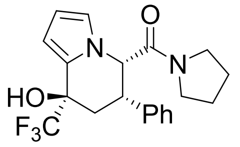

17

^{19}F NMR (471 MHz, $CDCl_3$)

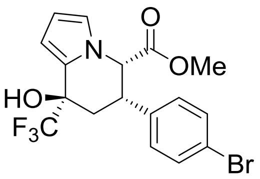

18

^{1}H NMR (400 MHz, $CDCl_3$)

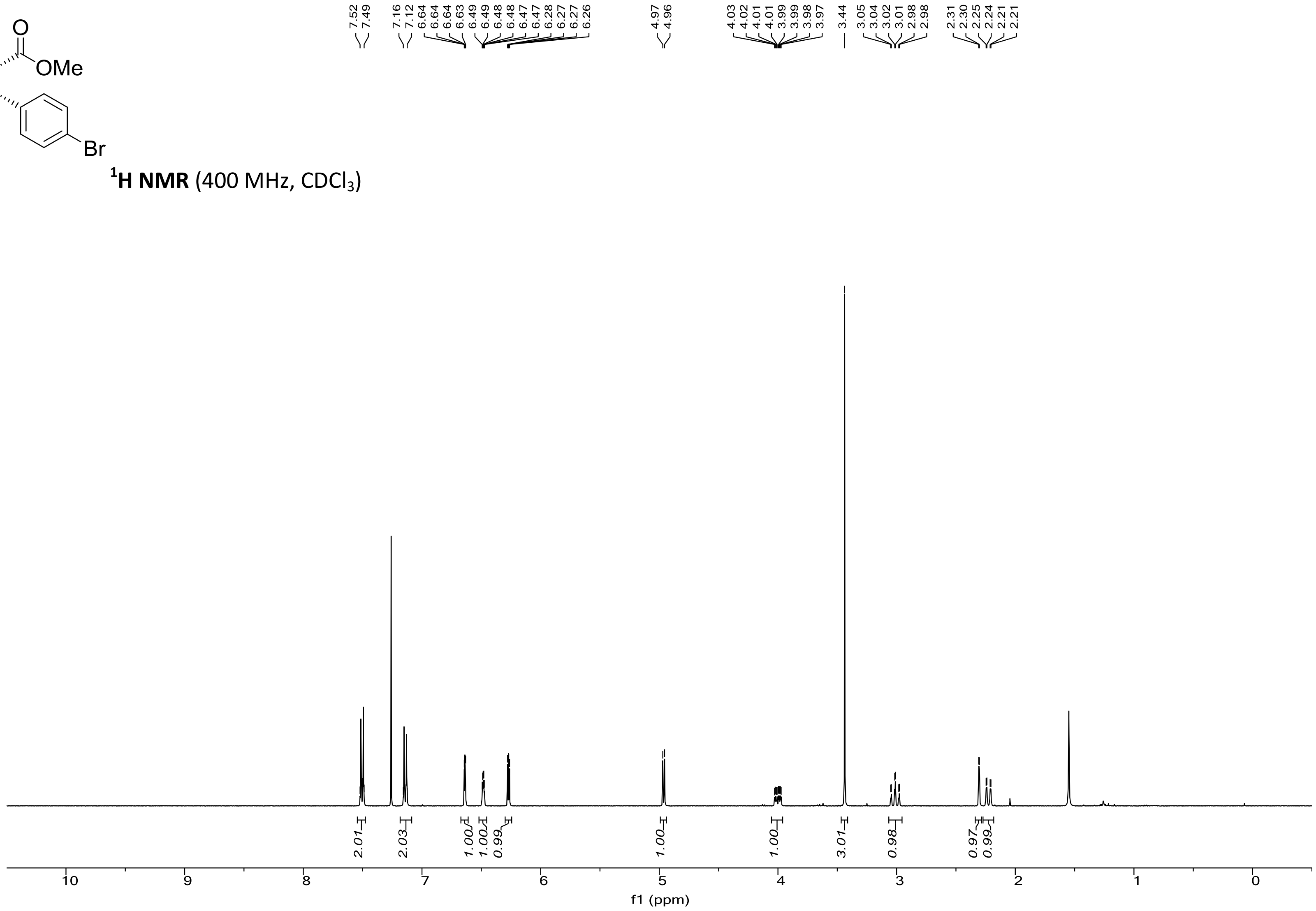

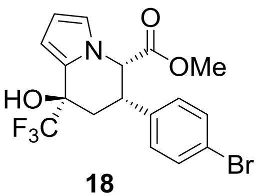

18

$^{13}C\{^1H\}$ NMR (126 MHz, $CDCl_3$)

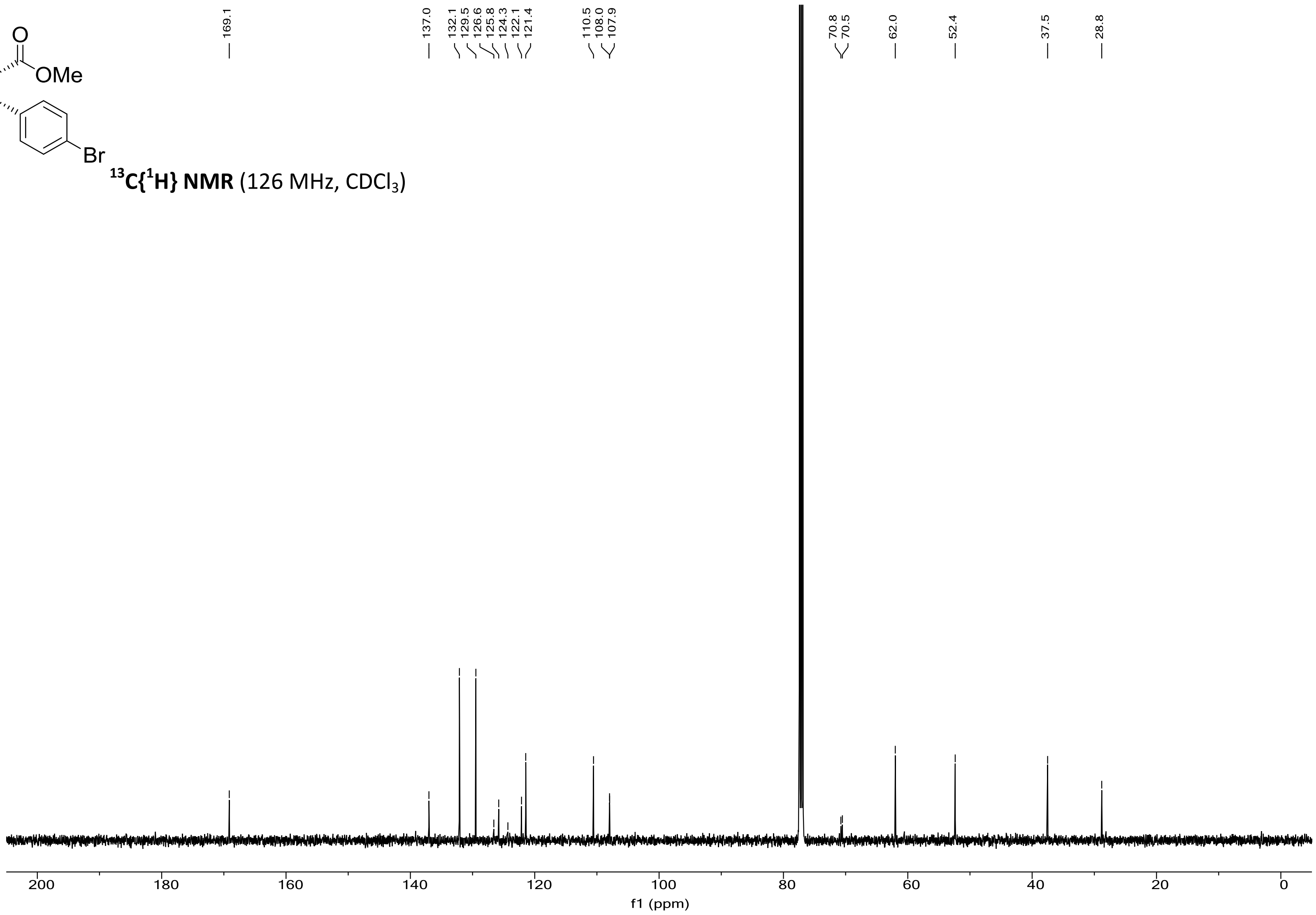

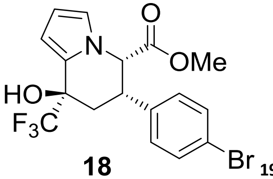

18

^{19}**F NMR** (471 MHz, $CDCl_3$)

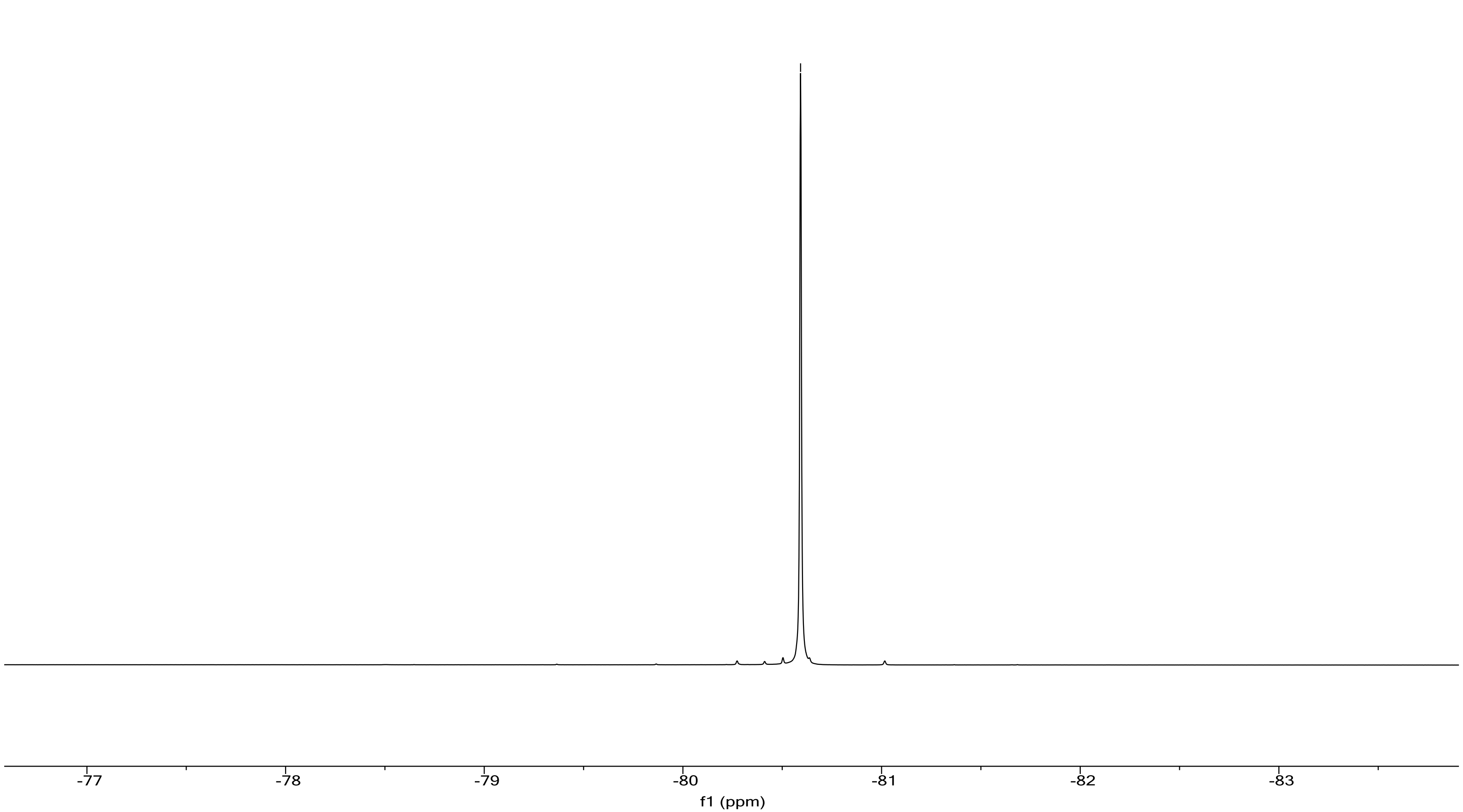

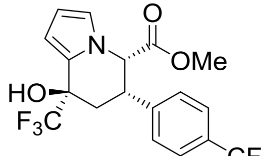

19

¹H NMR (400 MHz, $CDCl_3$)

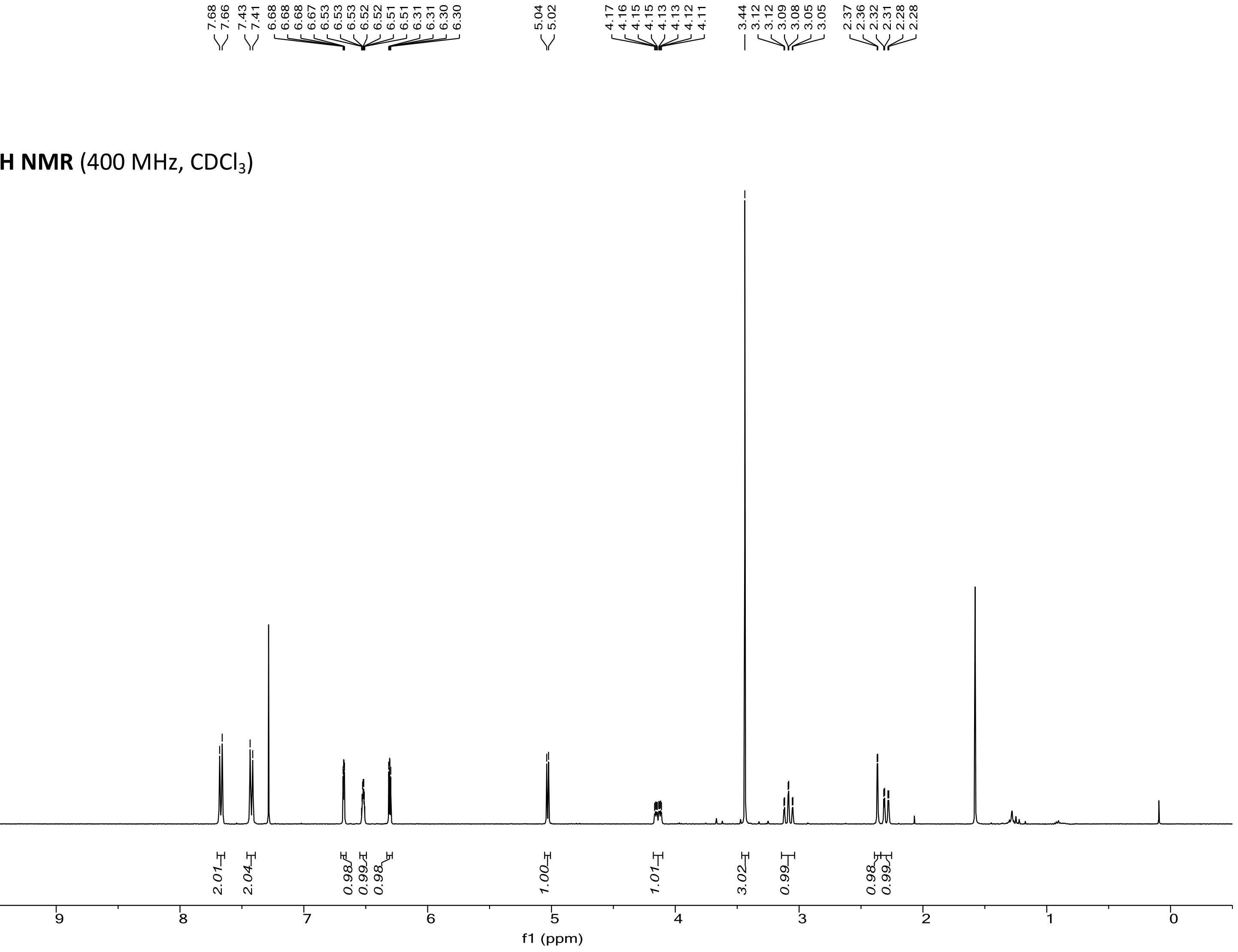

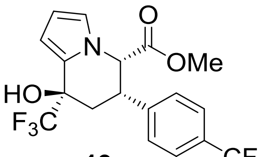

19

$^{13}C\{^{1}H\}$ NMR (126 MHz, $CDCl_3$)

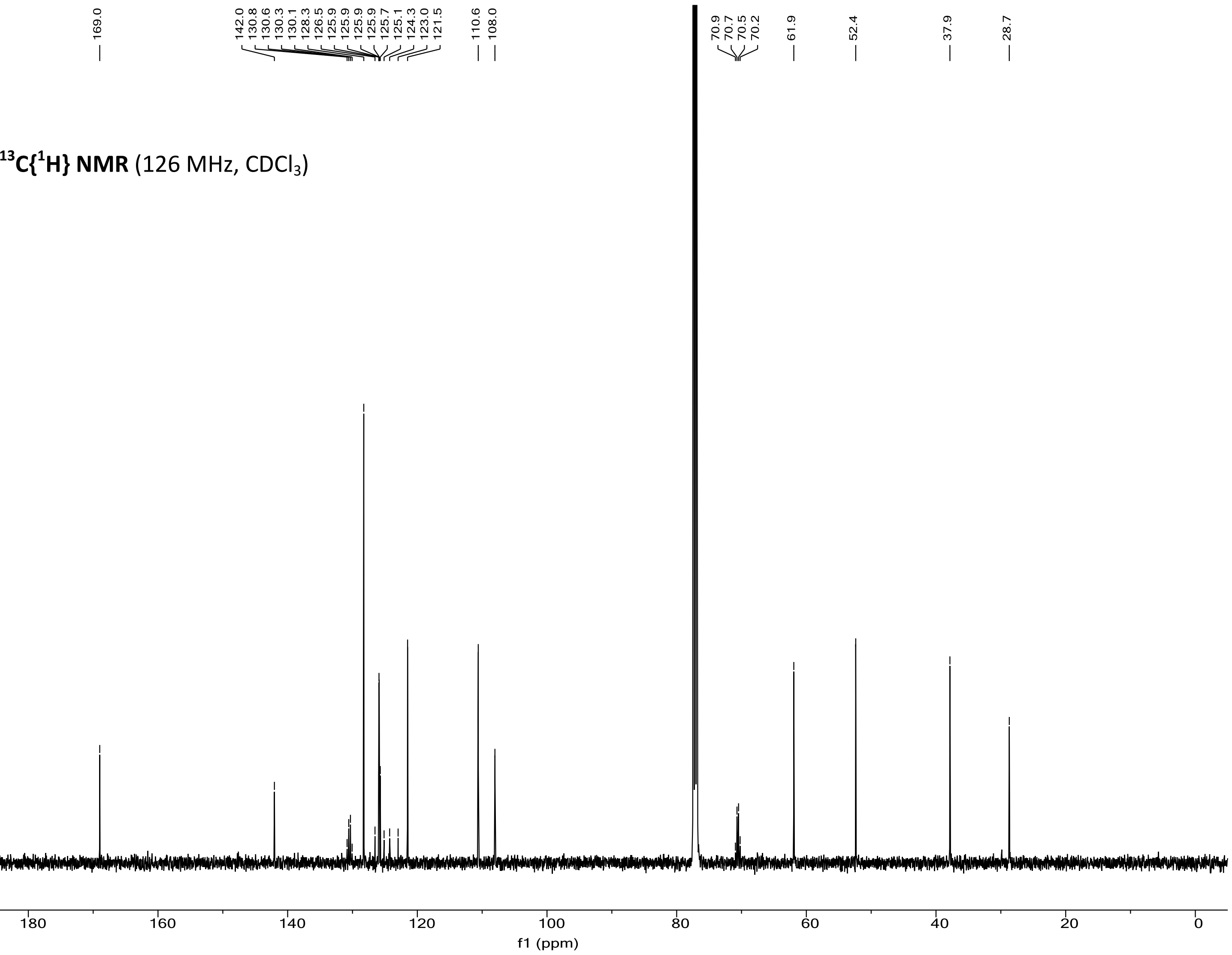

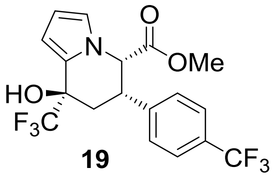

19

^{19}F NMR (471 MHz, $CDCl_3$)

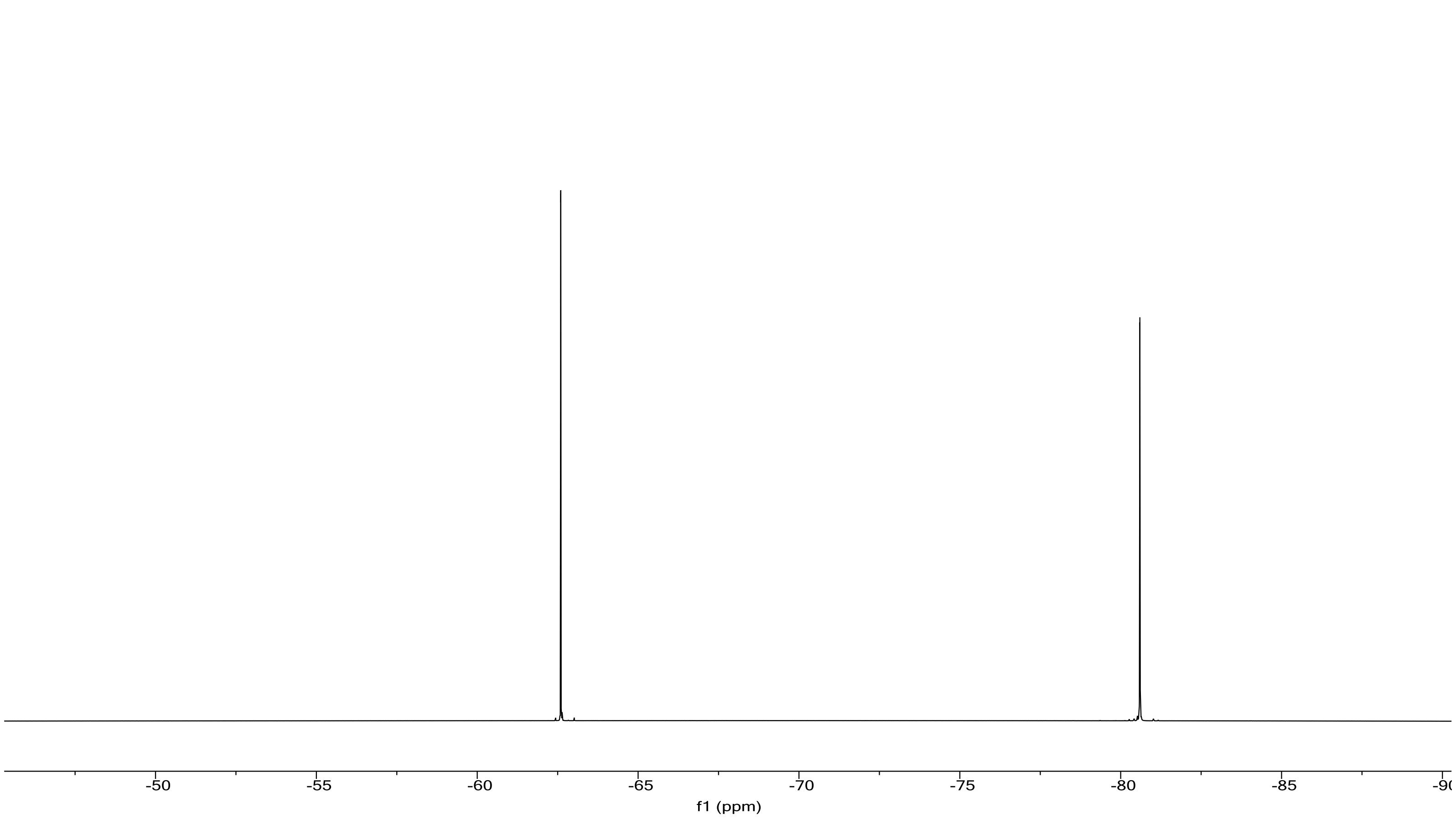

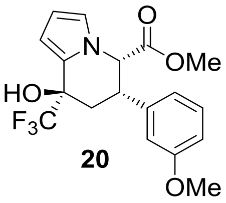

20

^{1}**H NMR** (400 MHz, $CDCl_3$)

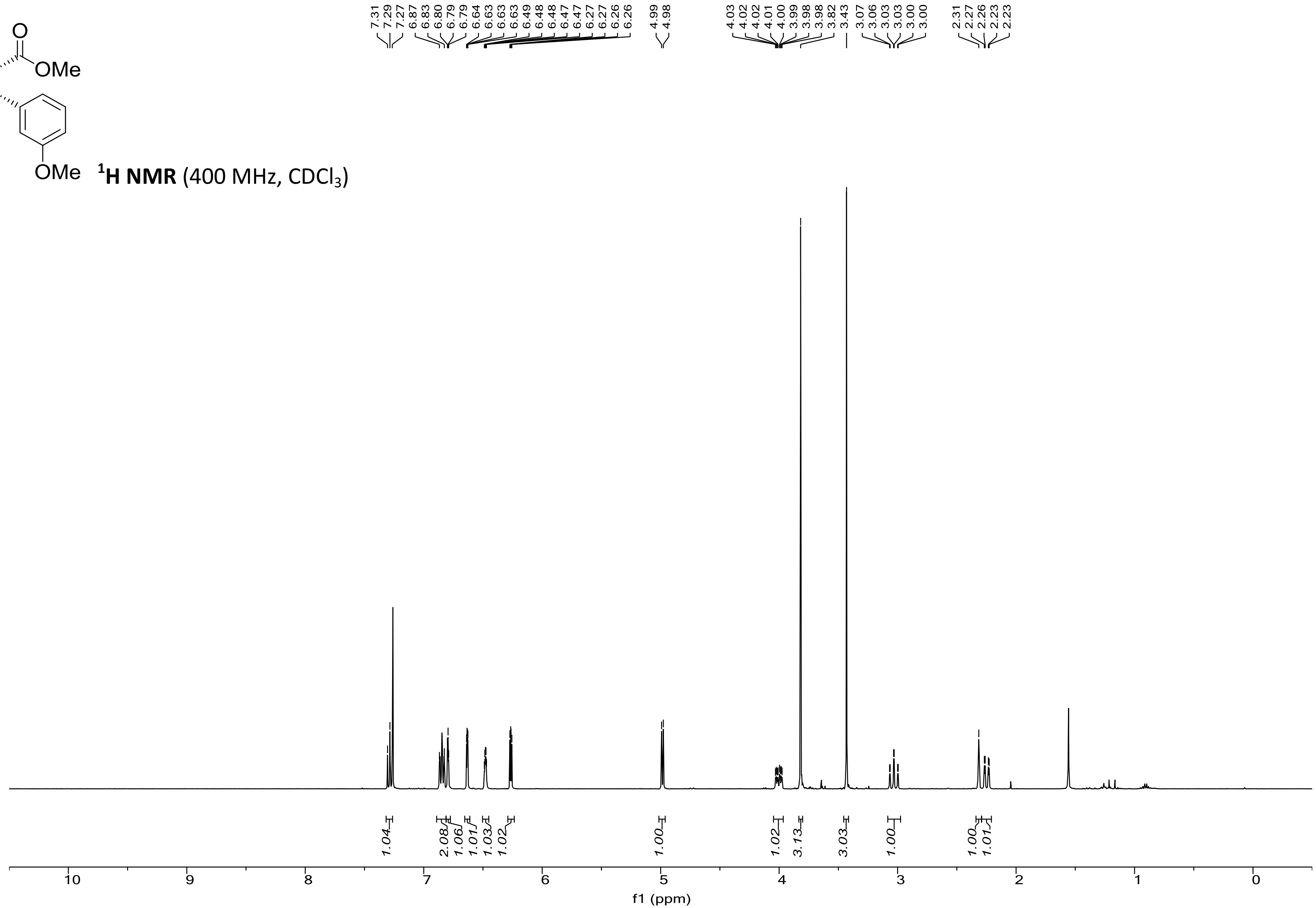

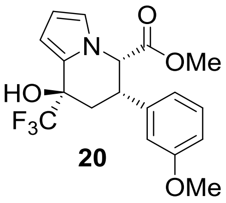

20

^{13}**C{^{1}H} NMR** (126 MHz, $CDCl_3$)

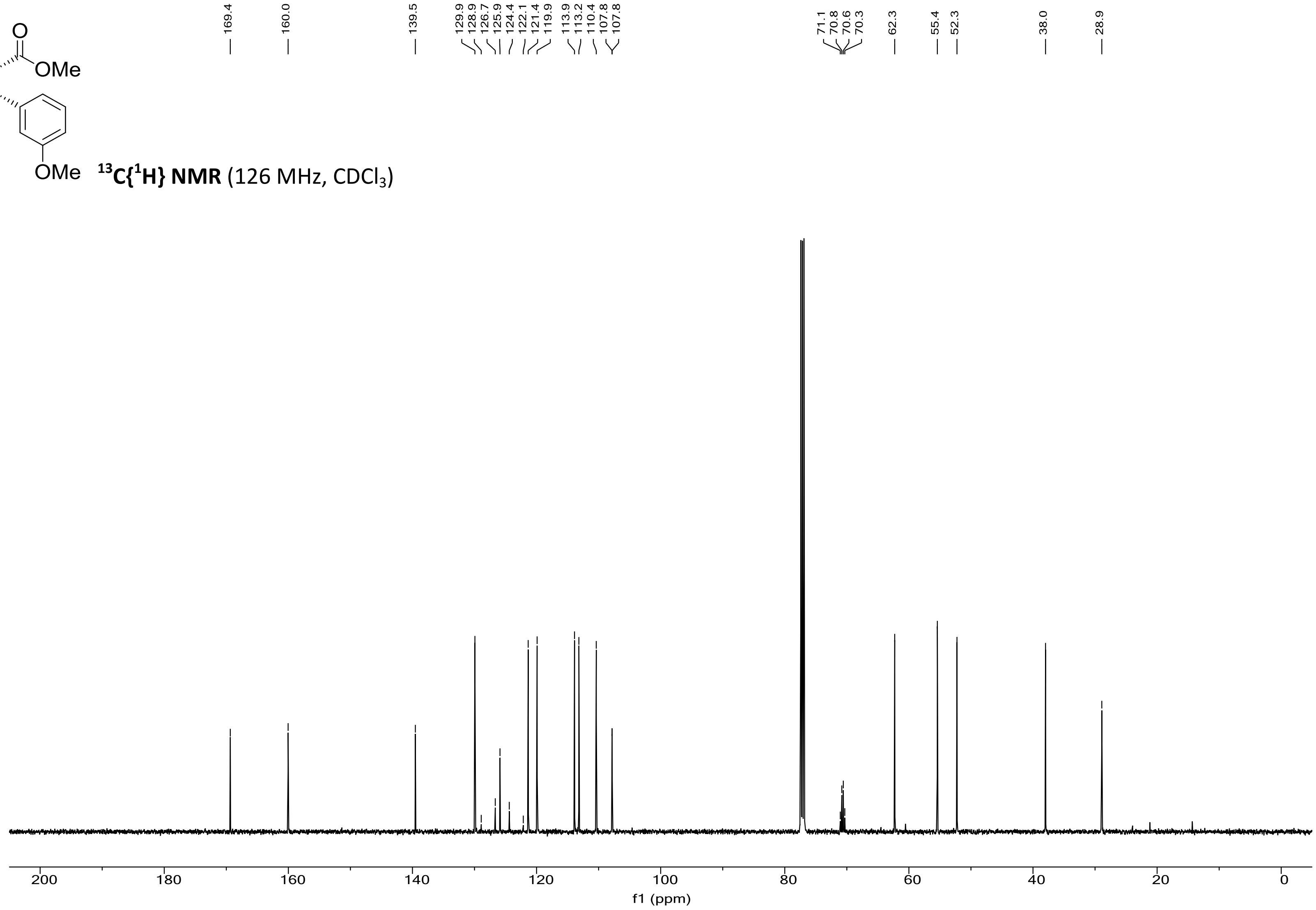

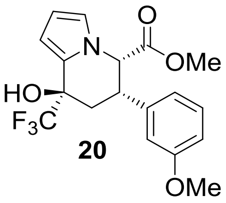

20

^{19}F NMR (471 MHz, $CDCl_3$)

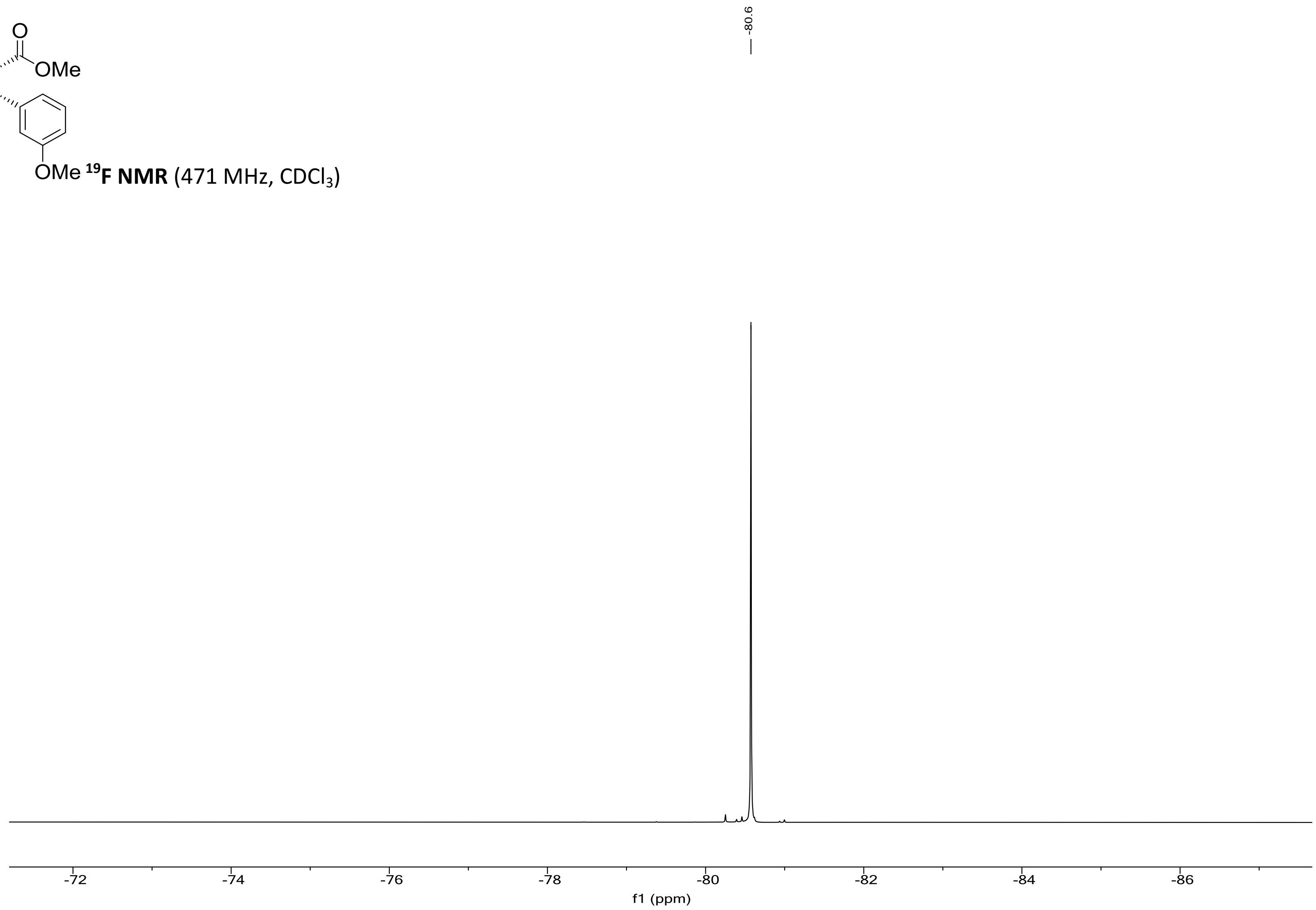

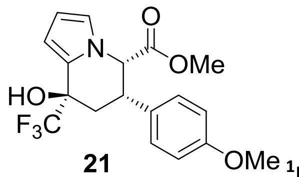

21

^{1}**H NMR** (500 MHz, $CDCl_3$)

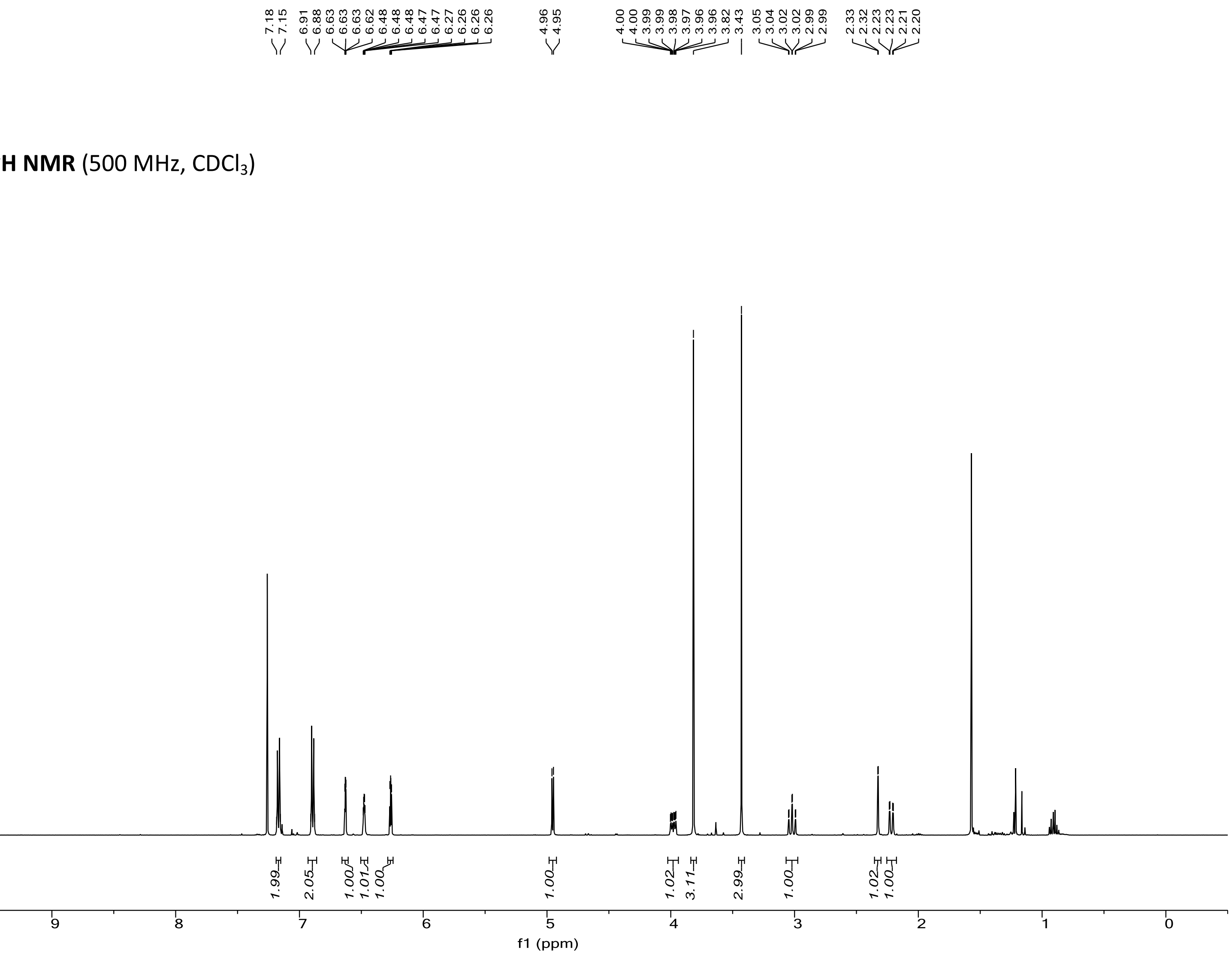

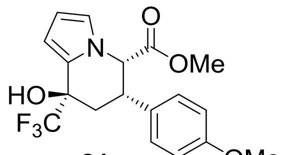

21

$^{13}C\{^1H\}$ NMR (126 MHz, $CDCl_3$)

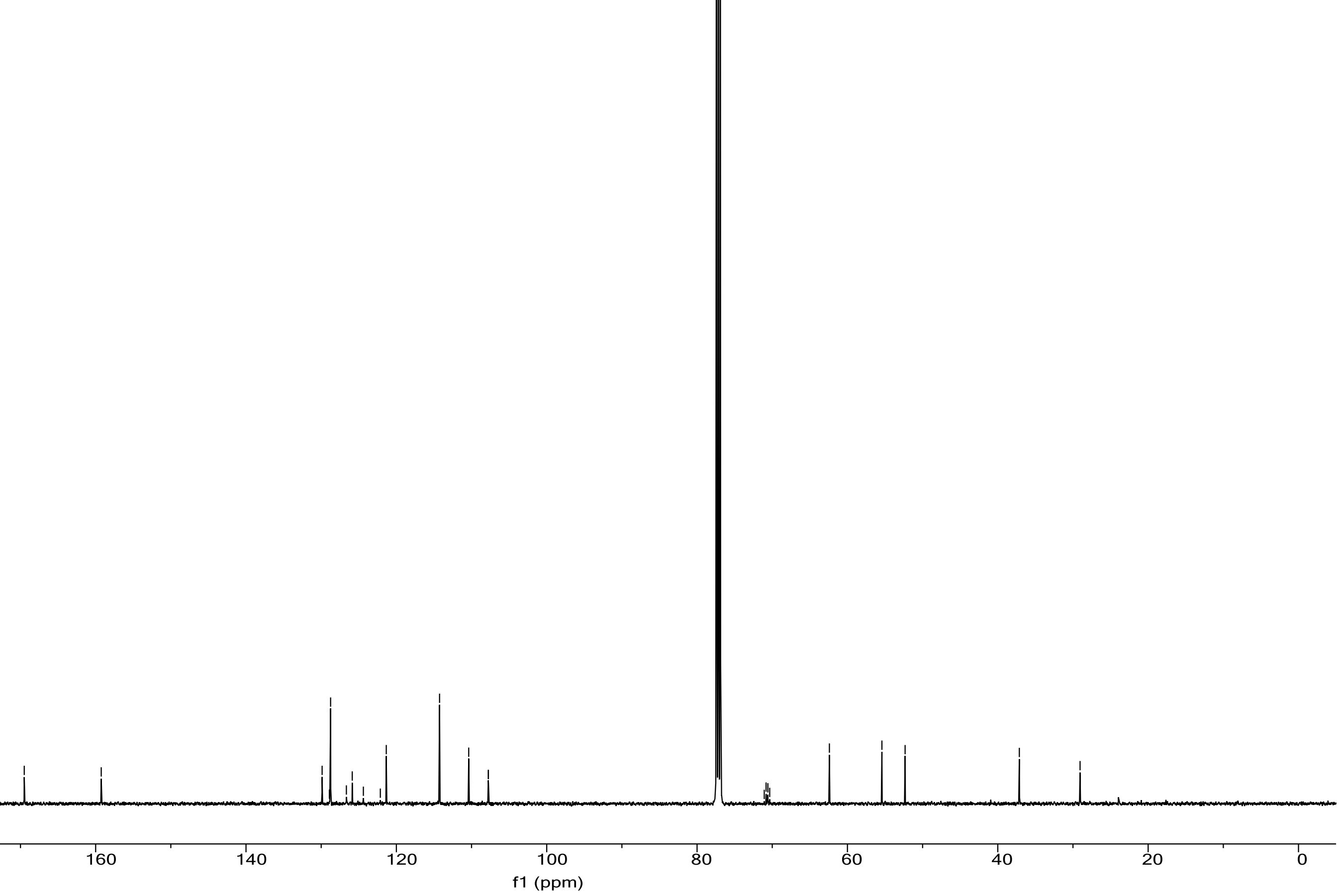

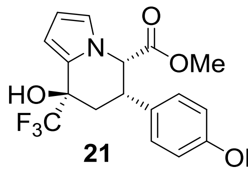

21

^{19}F NMR (471 MHz, $CDCl_3$)

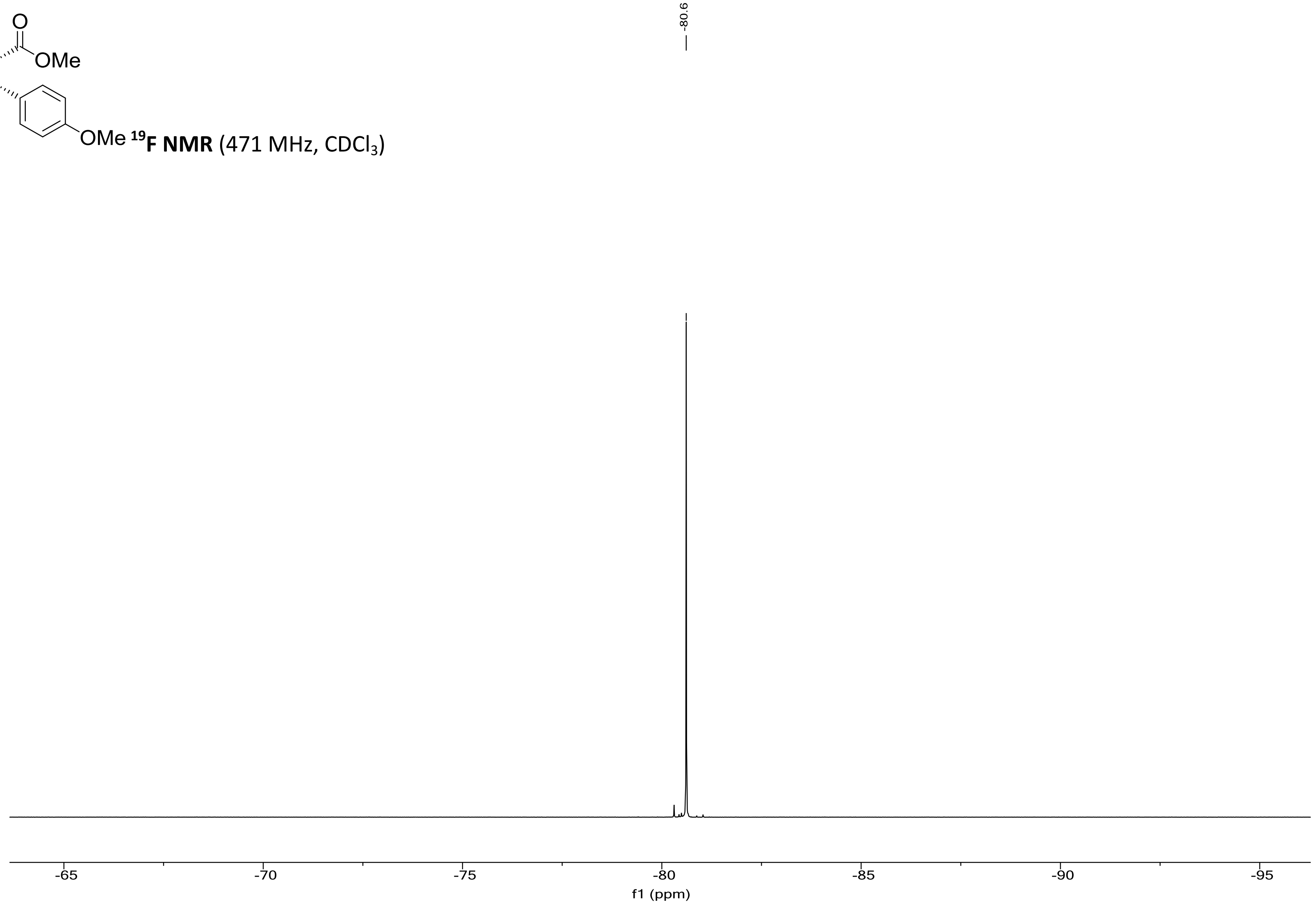

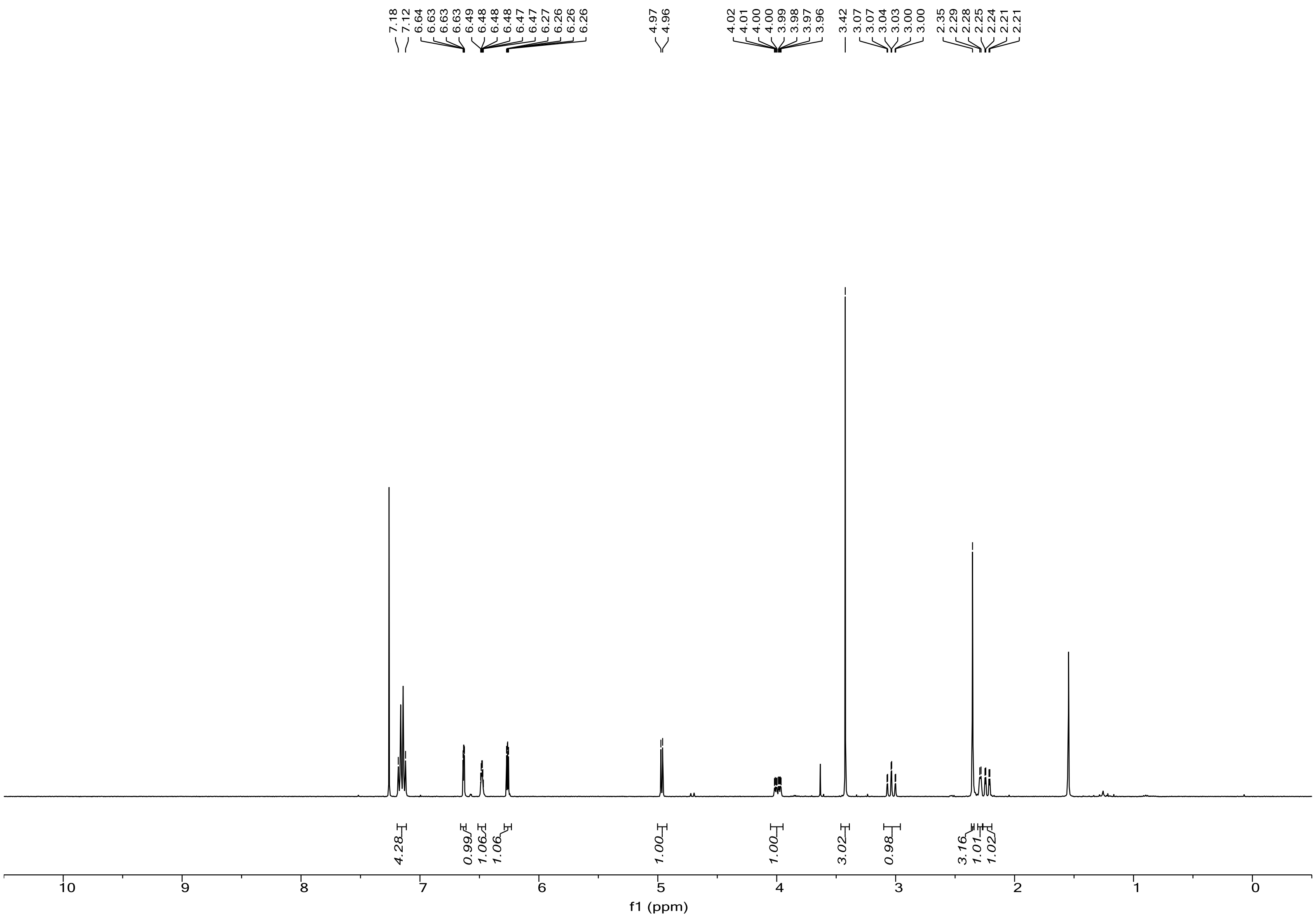

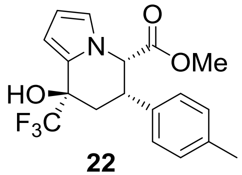

22

^{1}H NMR (400 MHz, $CDCl_3$)

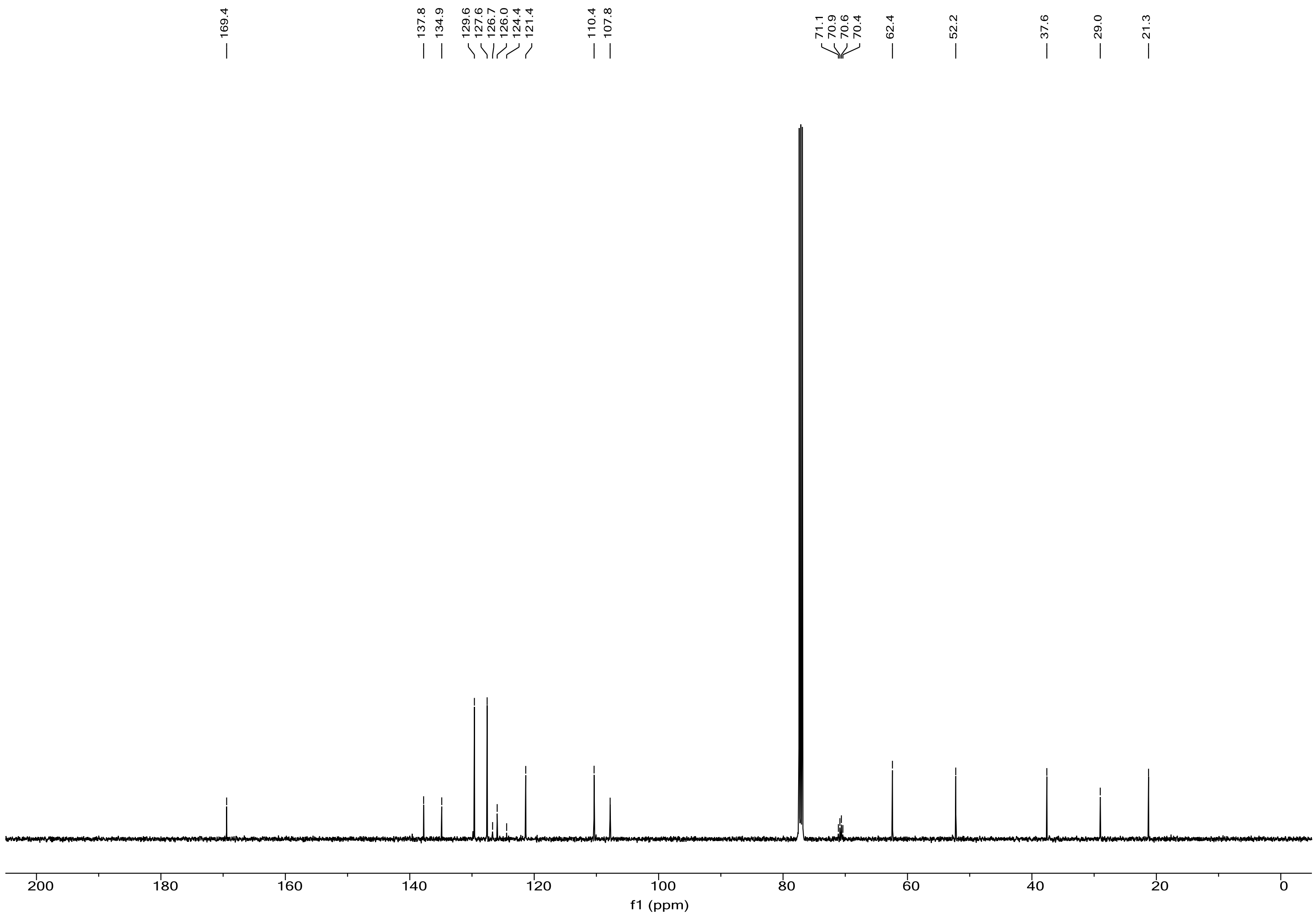

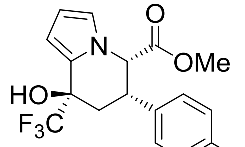

22

^{13}C{^{1}H} NMR (126 MHz, $CDCl_3$)

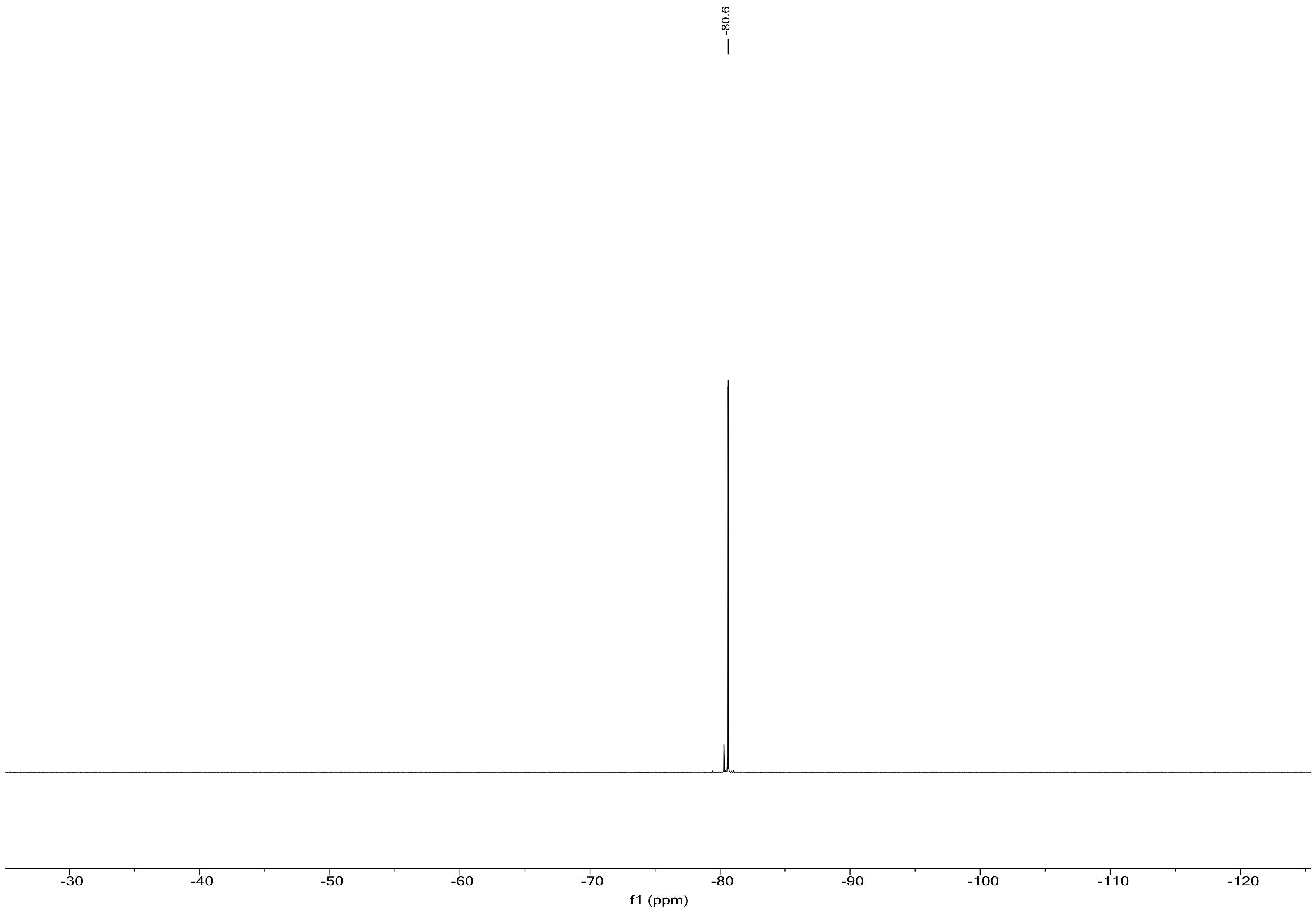

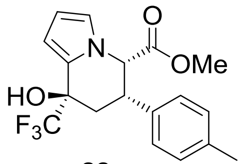

22

^{19}F NMR (471 MHz, $CDCl_3$)

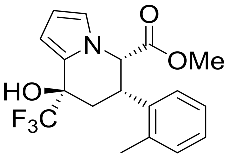

23

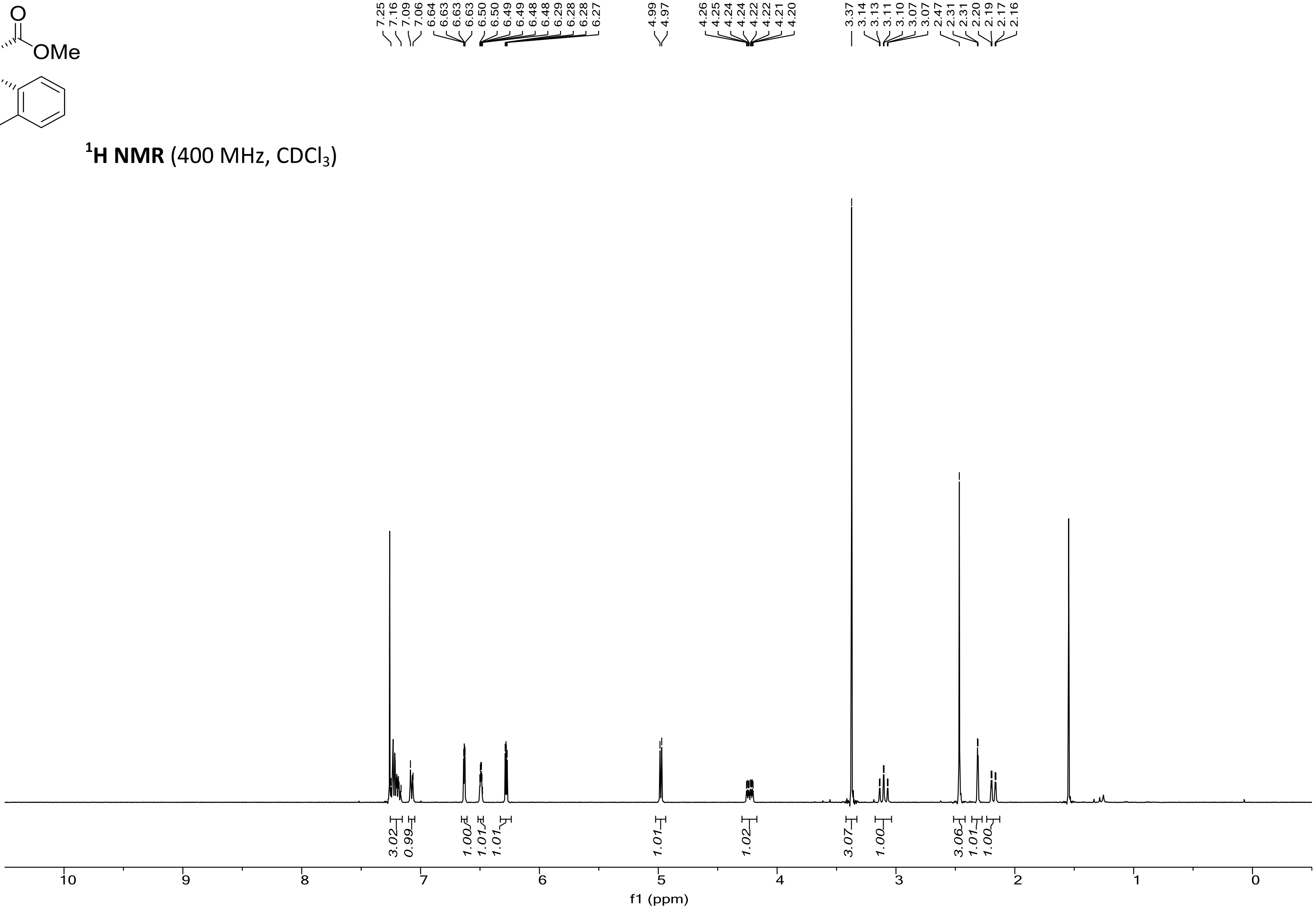

^{1}H NMR (400 MHz, $CDCl_3$)

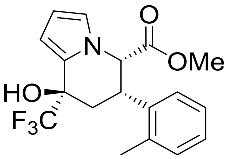

23

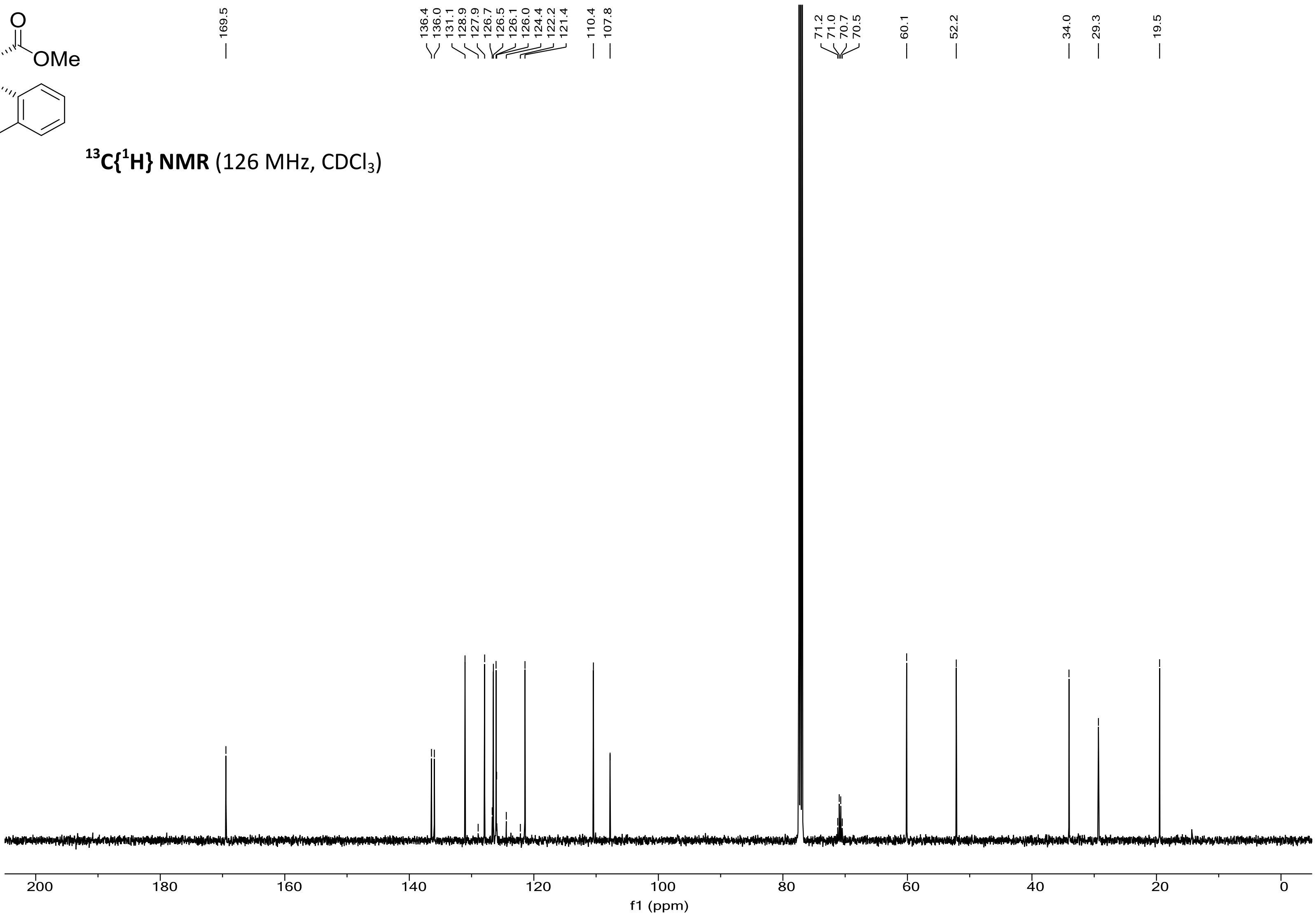

$^{13}C\{^{1}H\}$ NMR (126 MHz, $CDCl_3$)

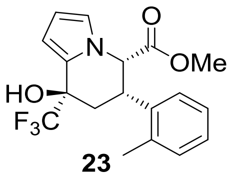

23

^{19}**F NMR** (471 MHz, $CDCl_3$)

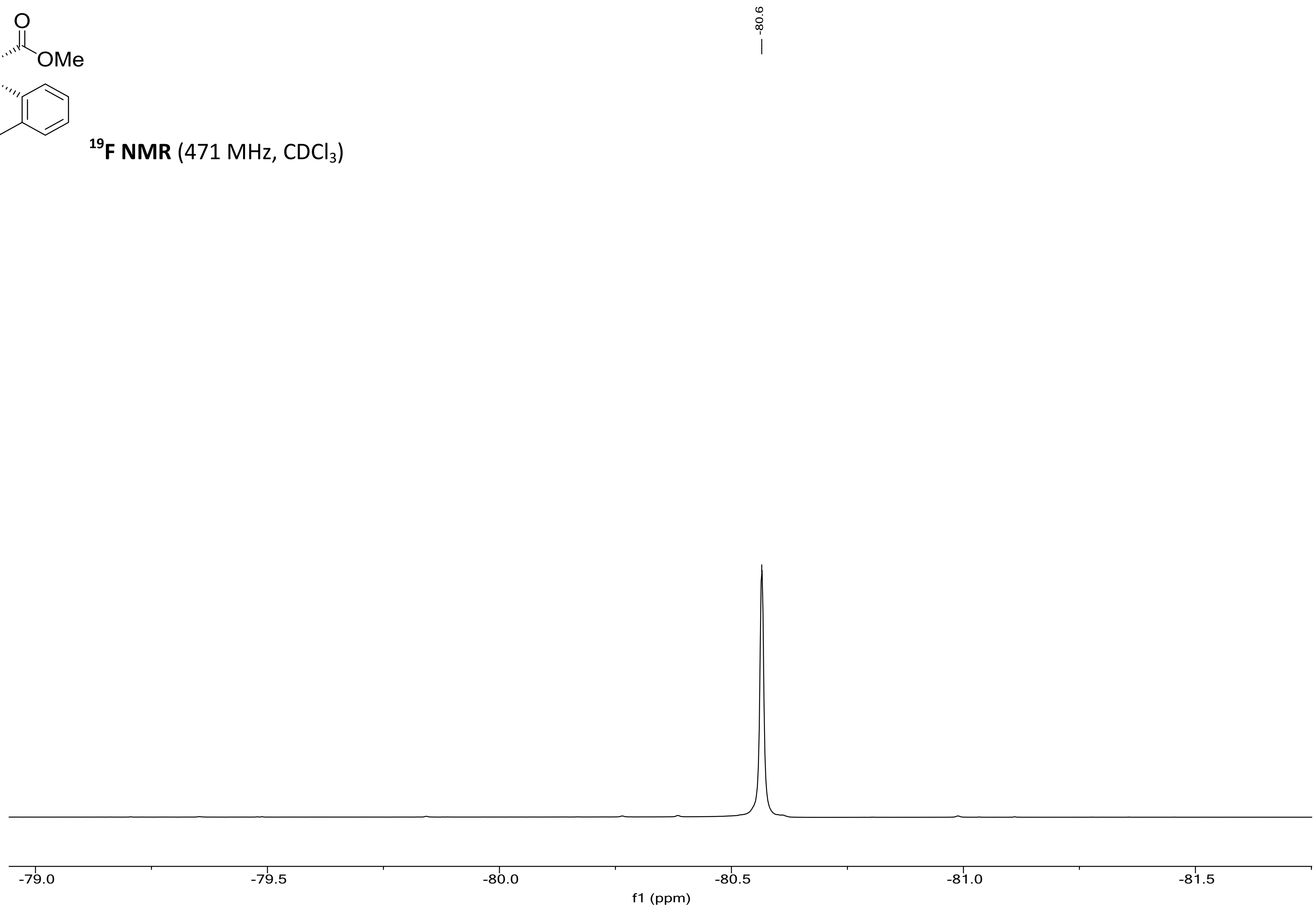

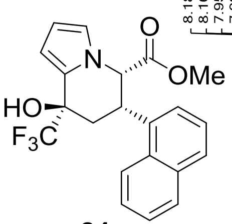

24

^{1}H NMR (400 MHz, $CDCl_3$)

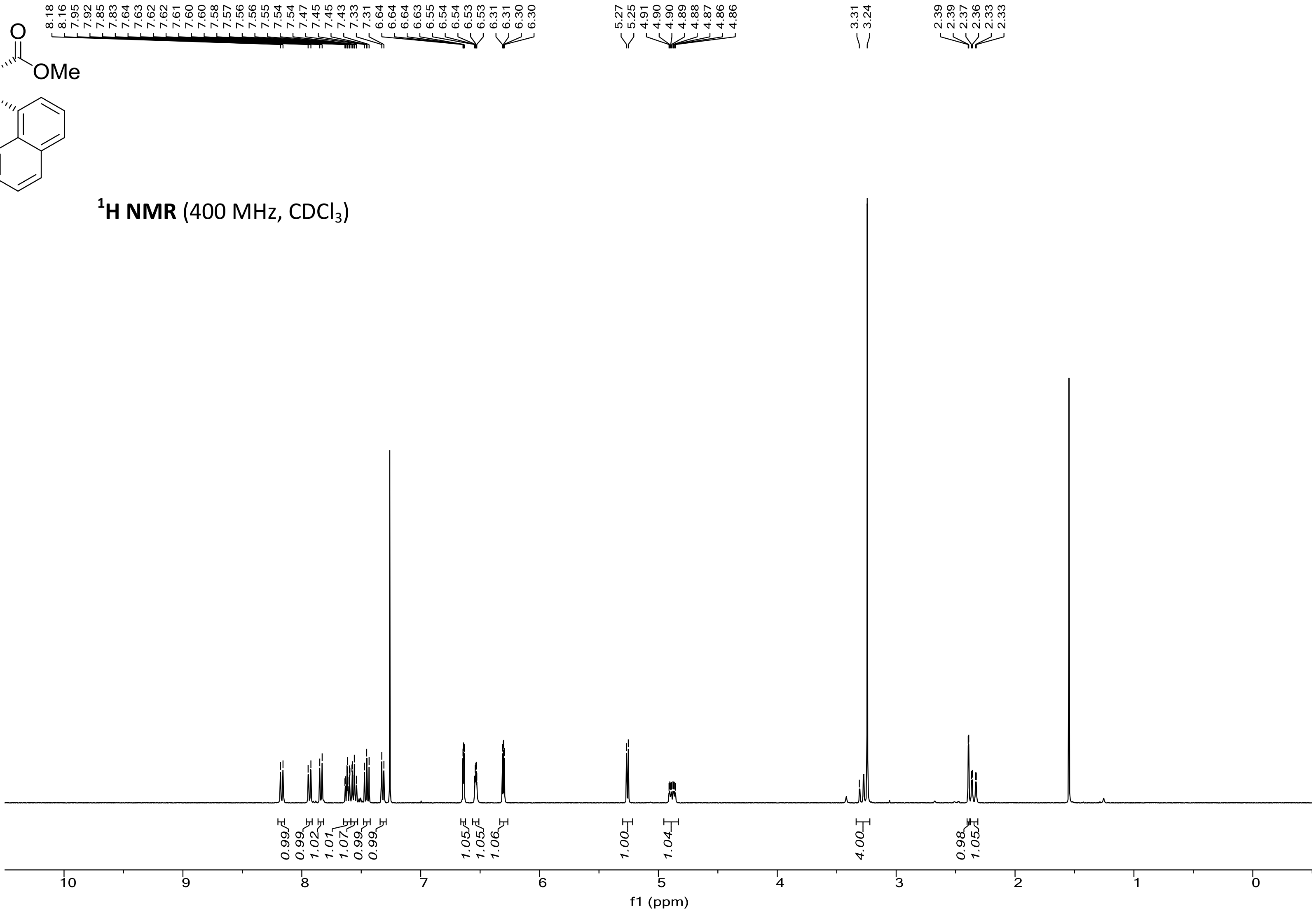

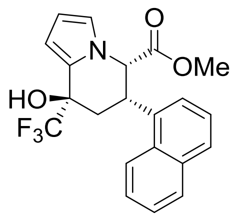

24

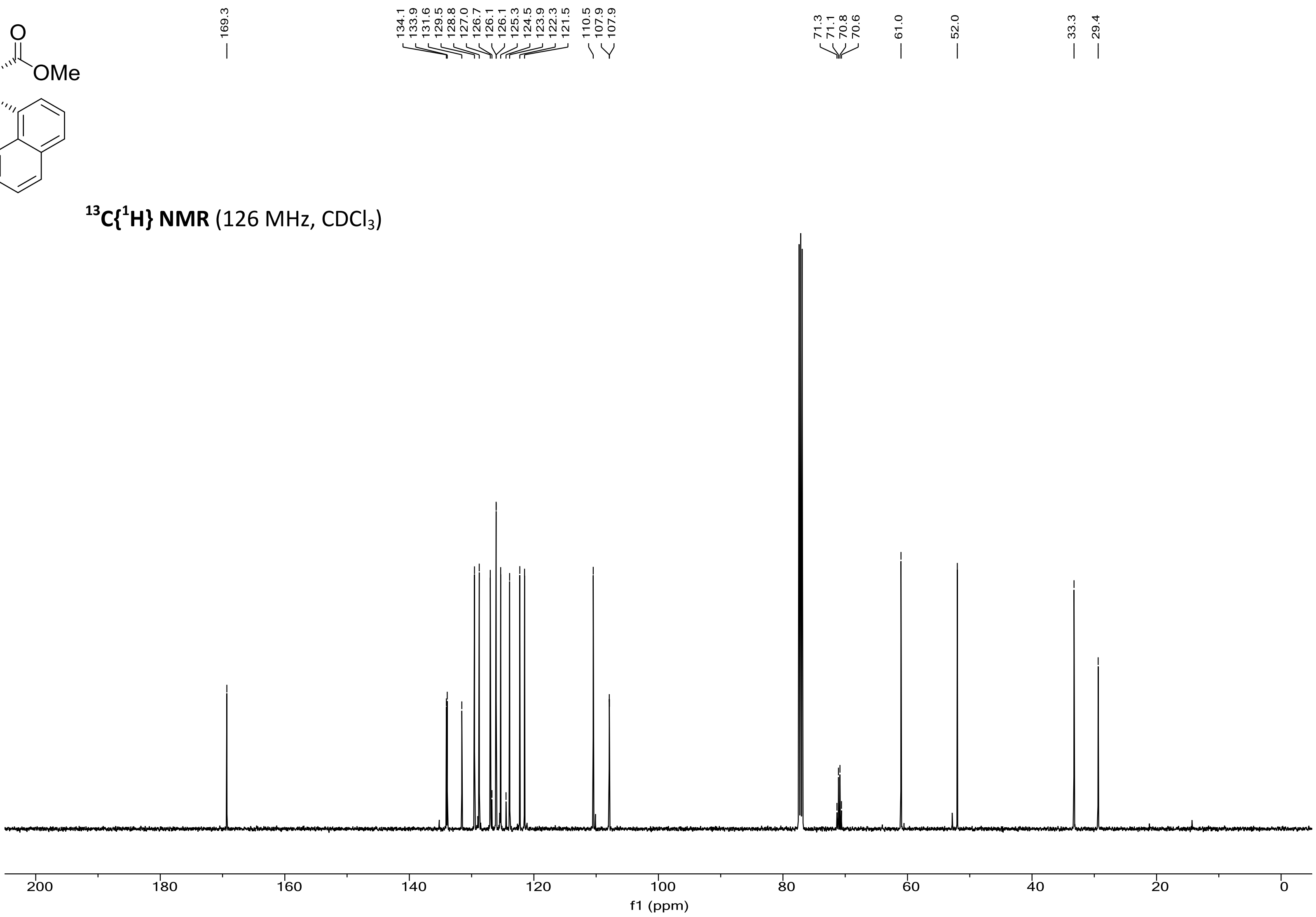

$^{13}C\{^{1}H\}$ NMR (126 MHz, $CDCl_3$)

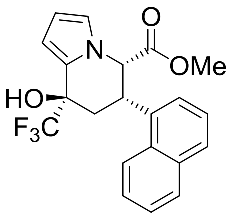

24

^{19}F NMR (471 MHz, $CDCl_3$)

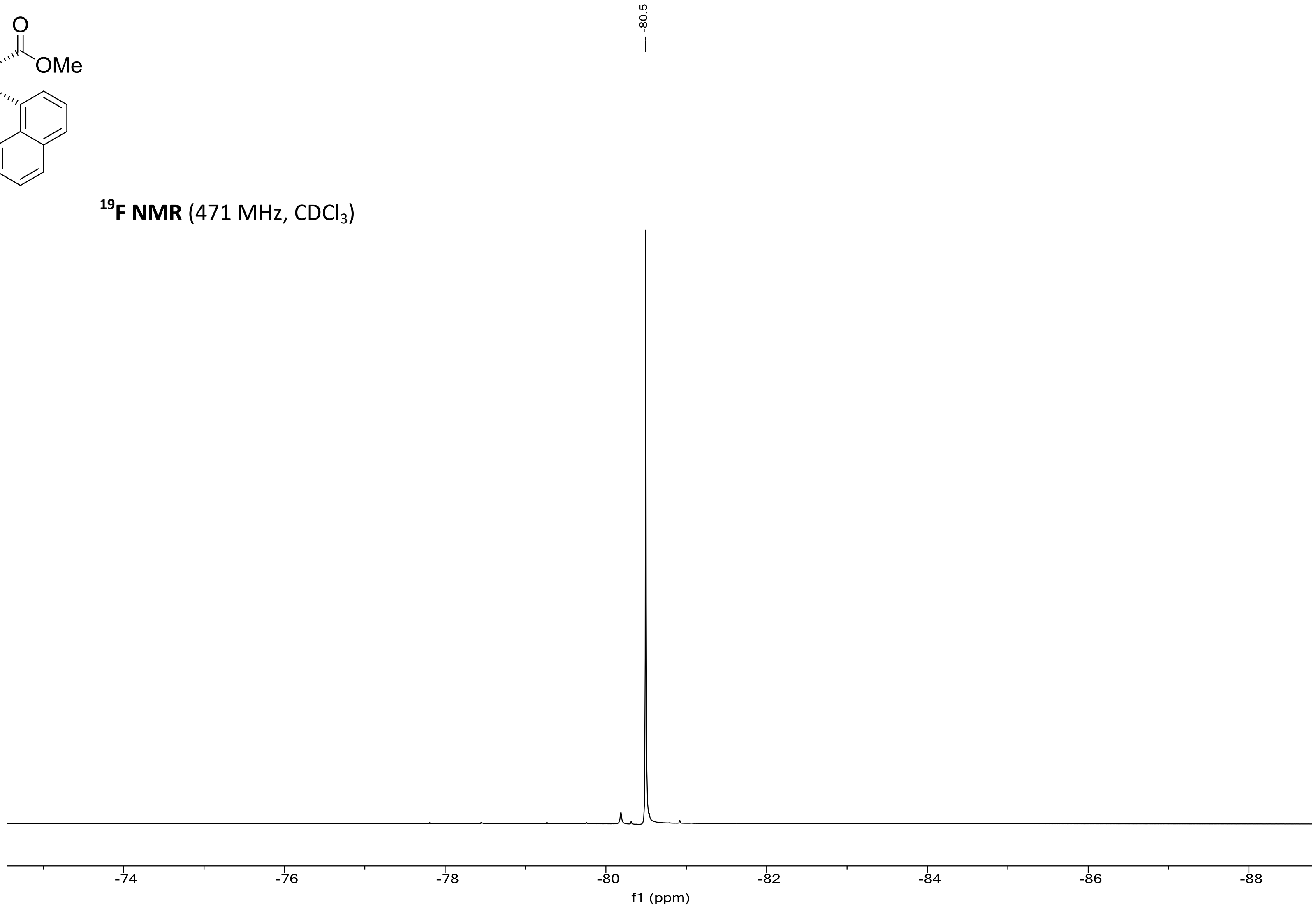

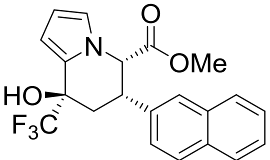

25

^{1}H NMR (500 MHz, $CDCl_3$)

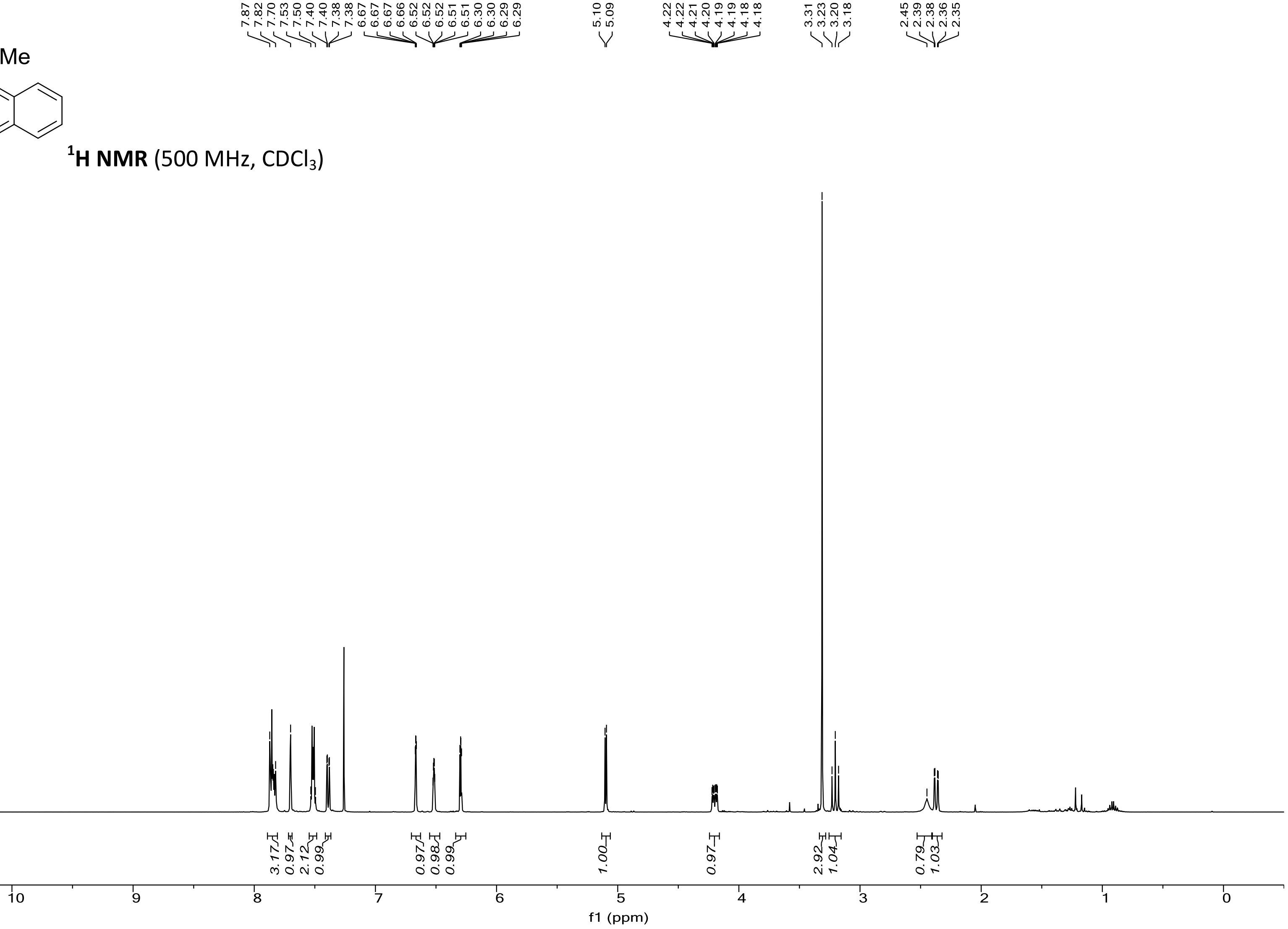

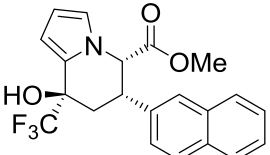

25

^{13}**C{^{1}H} NMR** (126 MHz, $CDCl_3$)

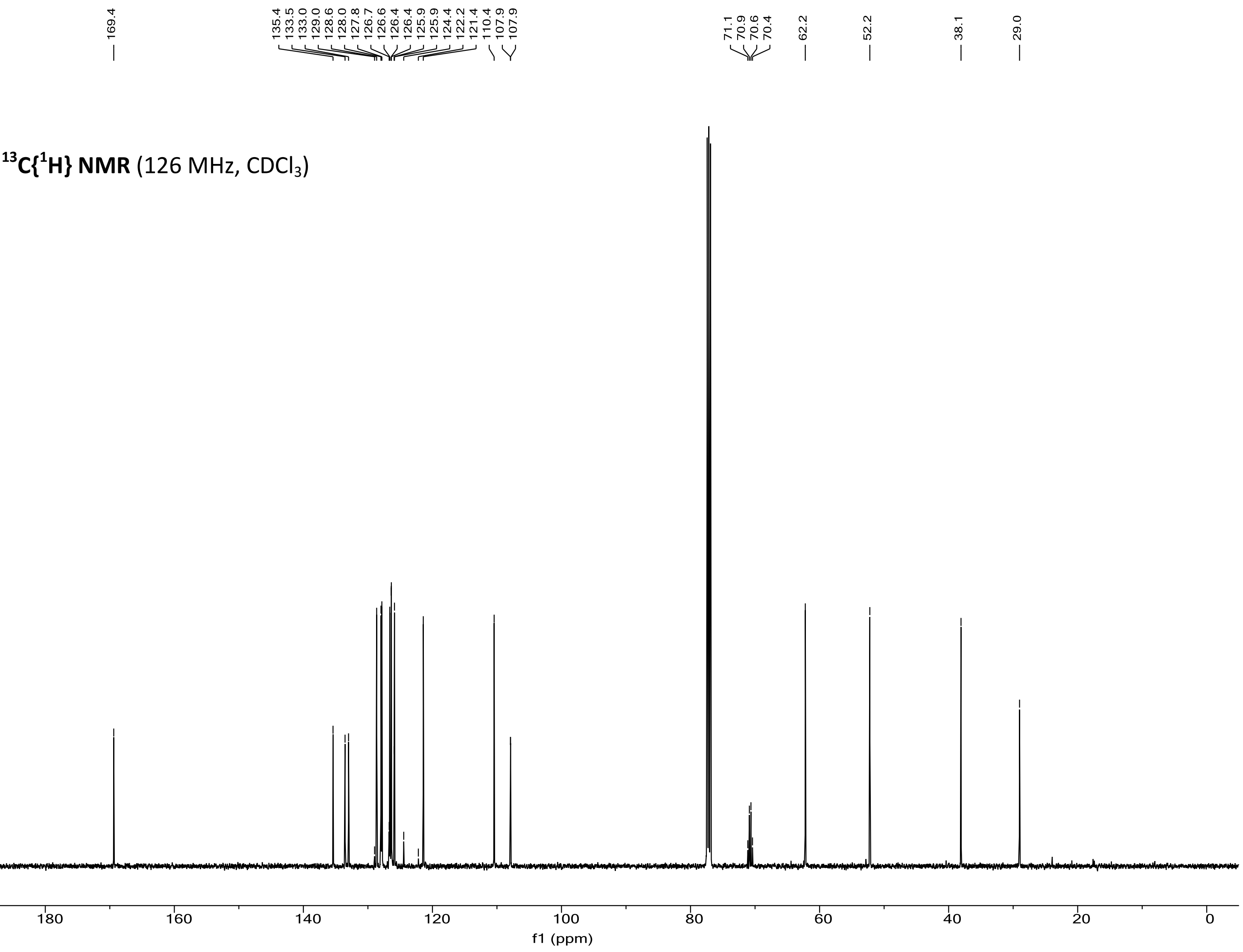

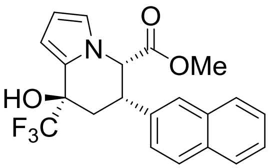

25

^{19}**F NMR** (471 MHz, $CDCl_3$)

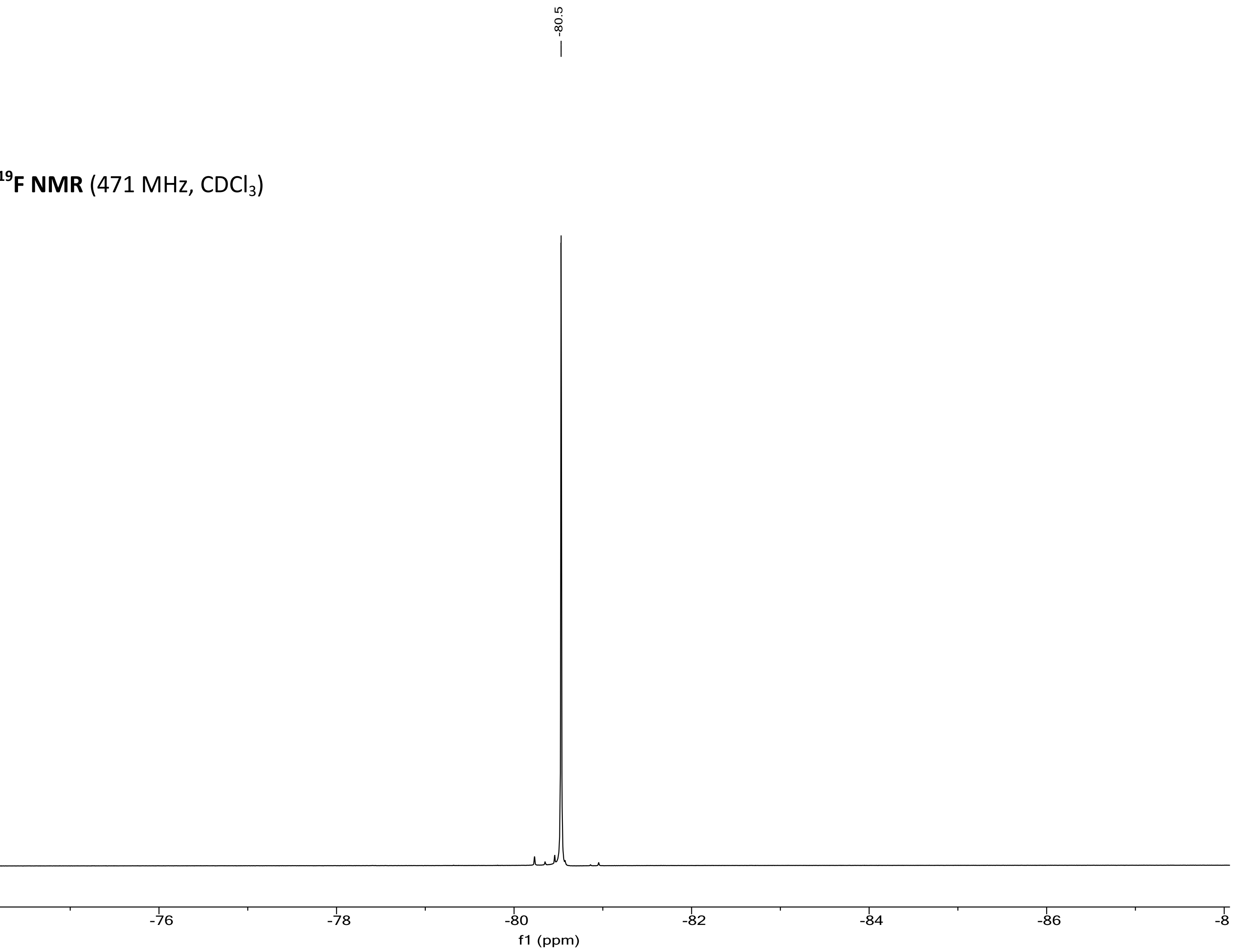

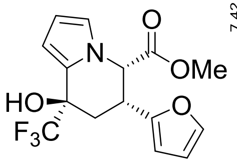

26

^{1}H NMR (400 MHz, $CDCl_3$)

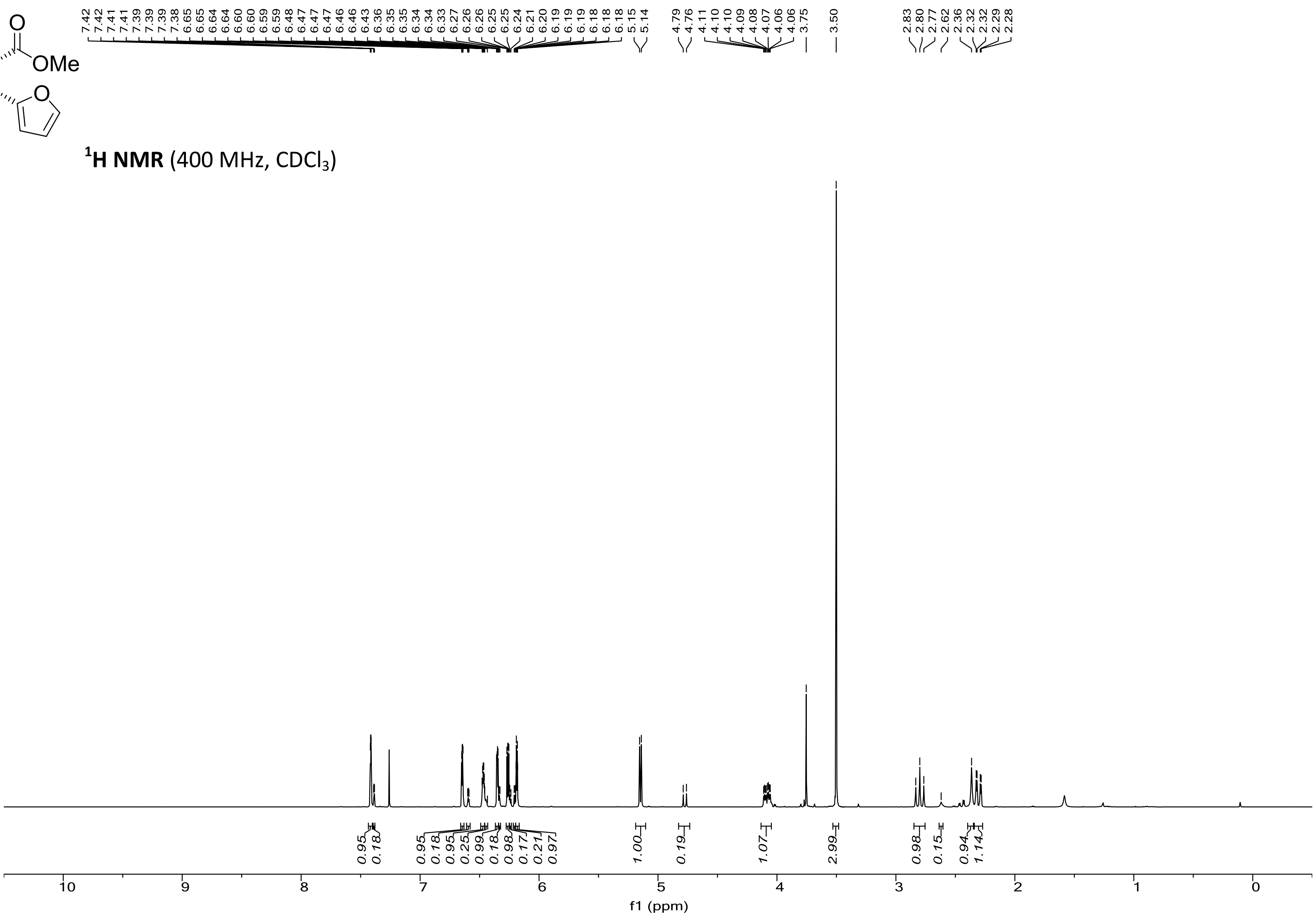

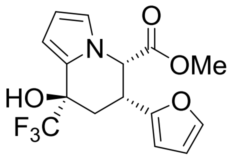

26

^{13}**C{^{1}H} NMR** (126 MHz, $CDCl_3$)

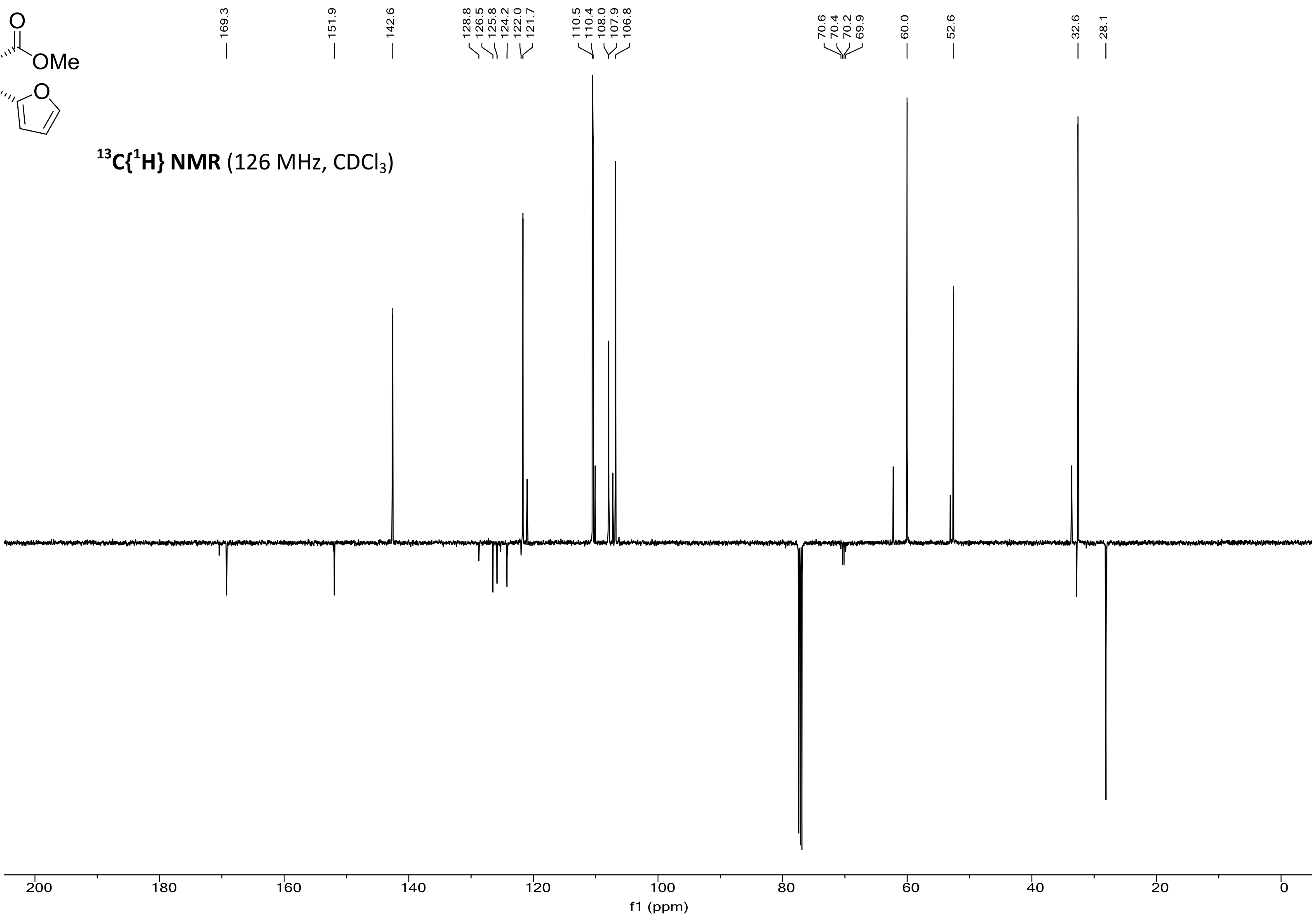

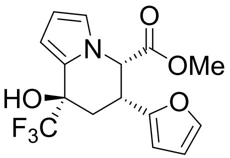

26

^{19}F NMR (471 MHz, $CDCl_3$)

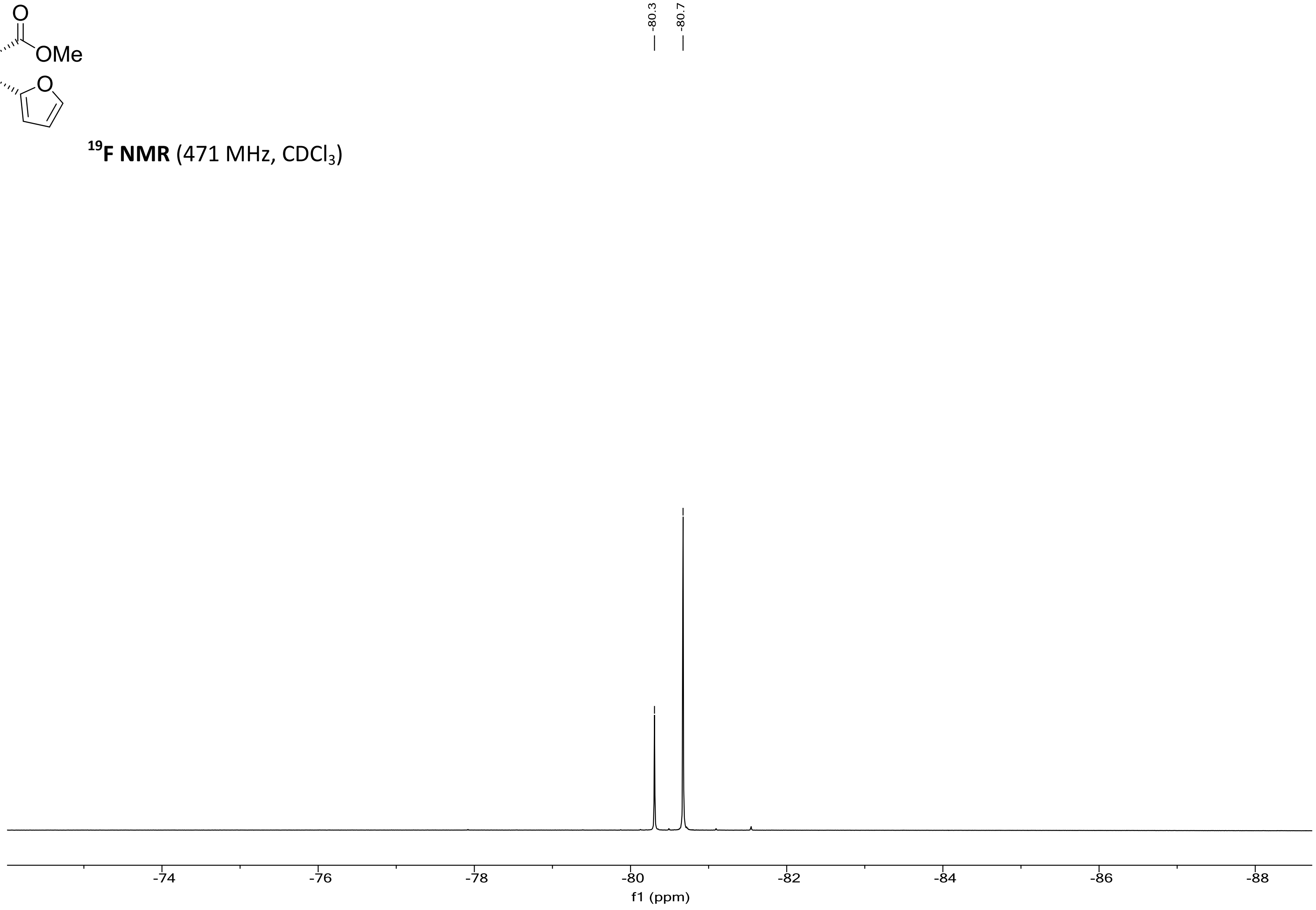

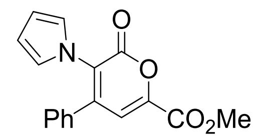

28

^{1}H NMR (500 MHz, CDCl$_3$)

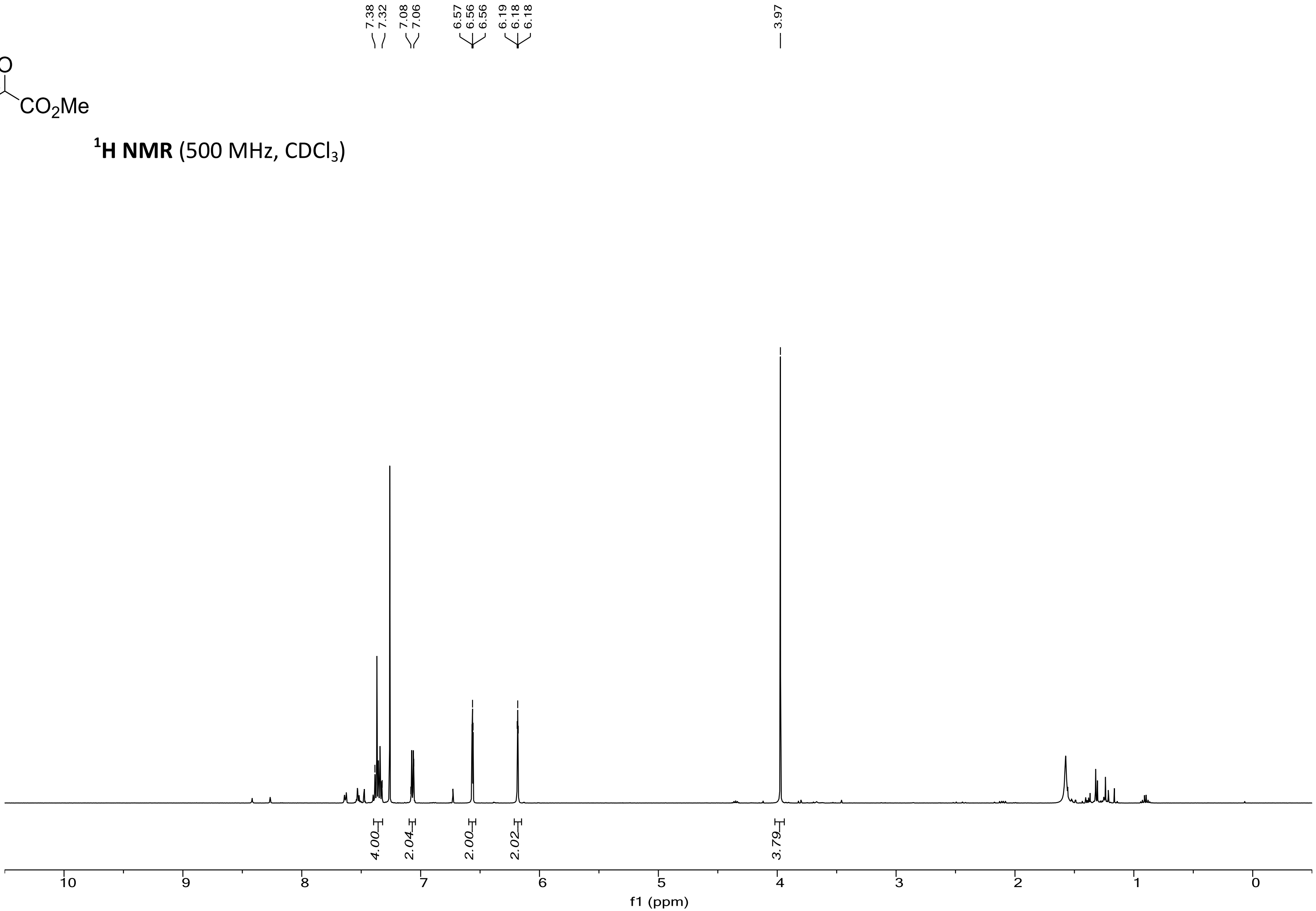

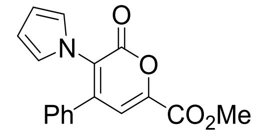

28

$^{13}C\{^{1}H\}$ NMR (126 MHz, $CDCl_3$)

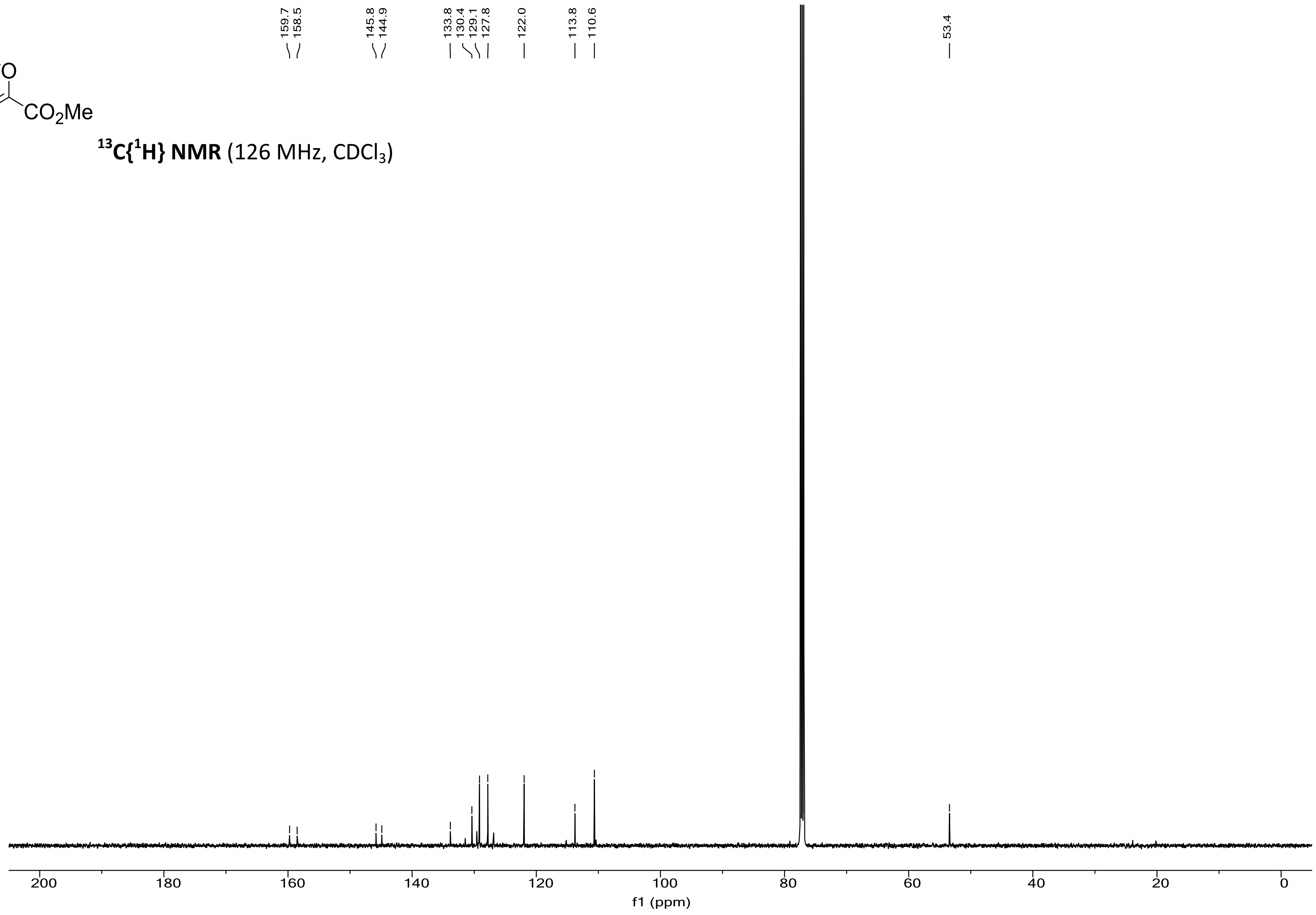

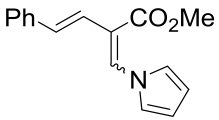

29

^{1}H NMR (500 MHz, $CDCl_3$)

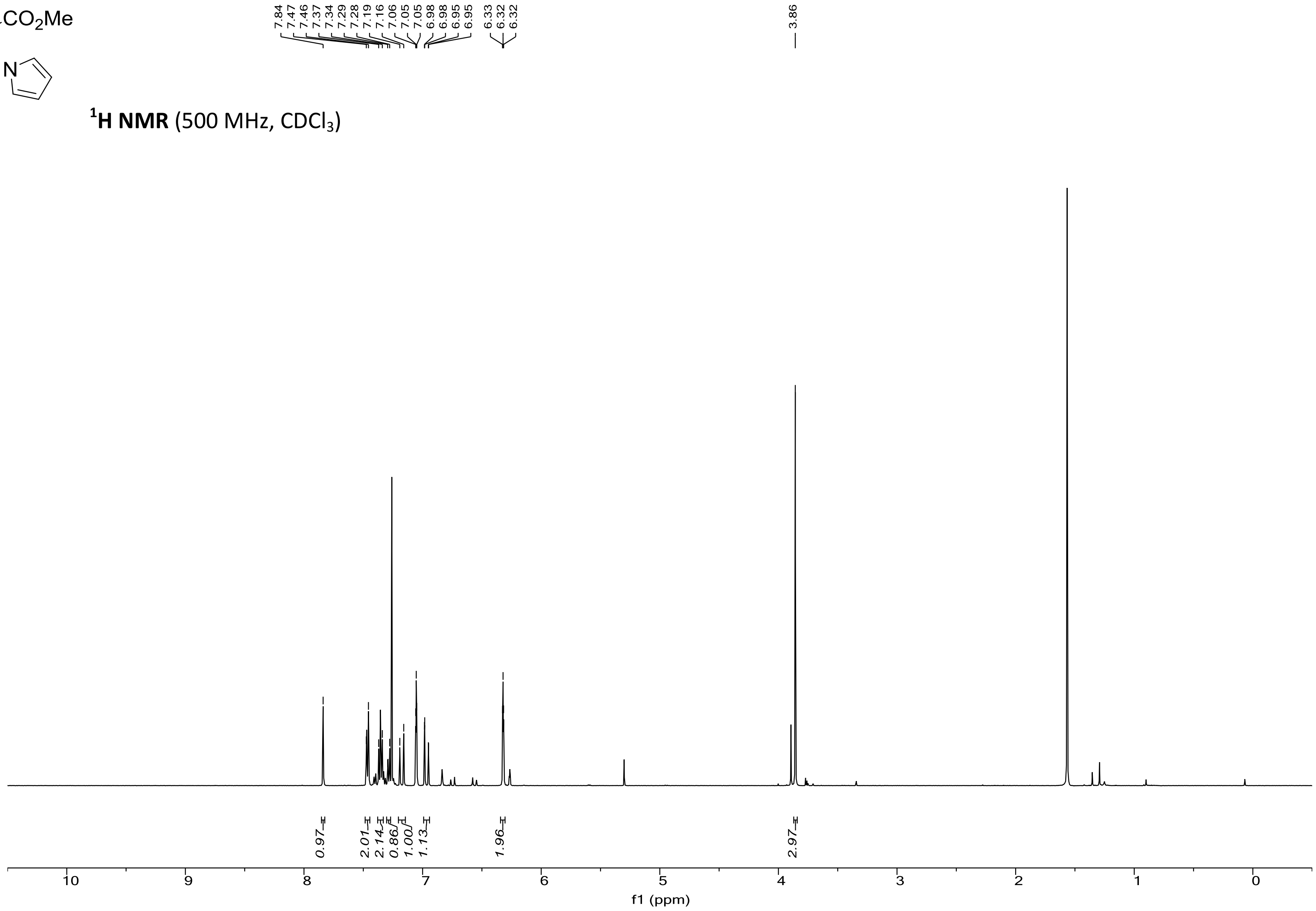

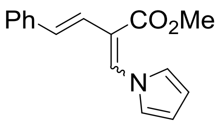

29

^{13}C{^{1}H} NMR (126 MHz, $CDCl_3$)

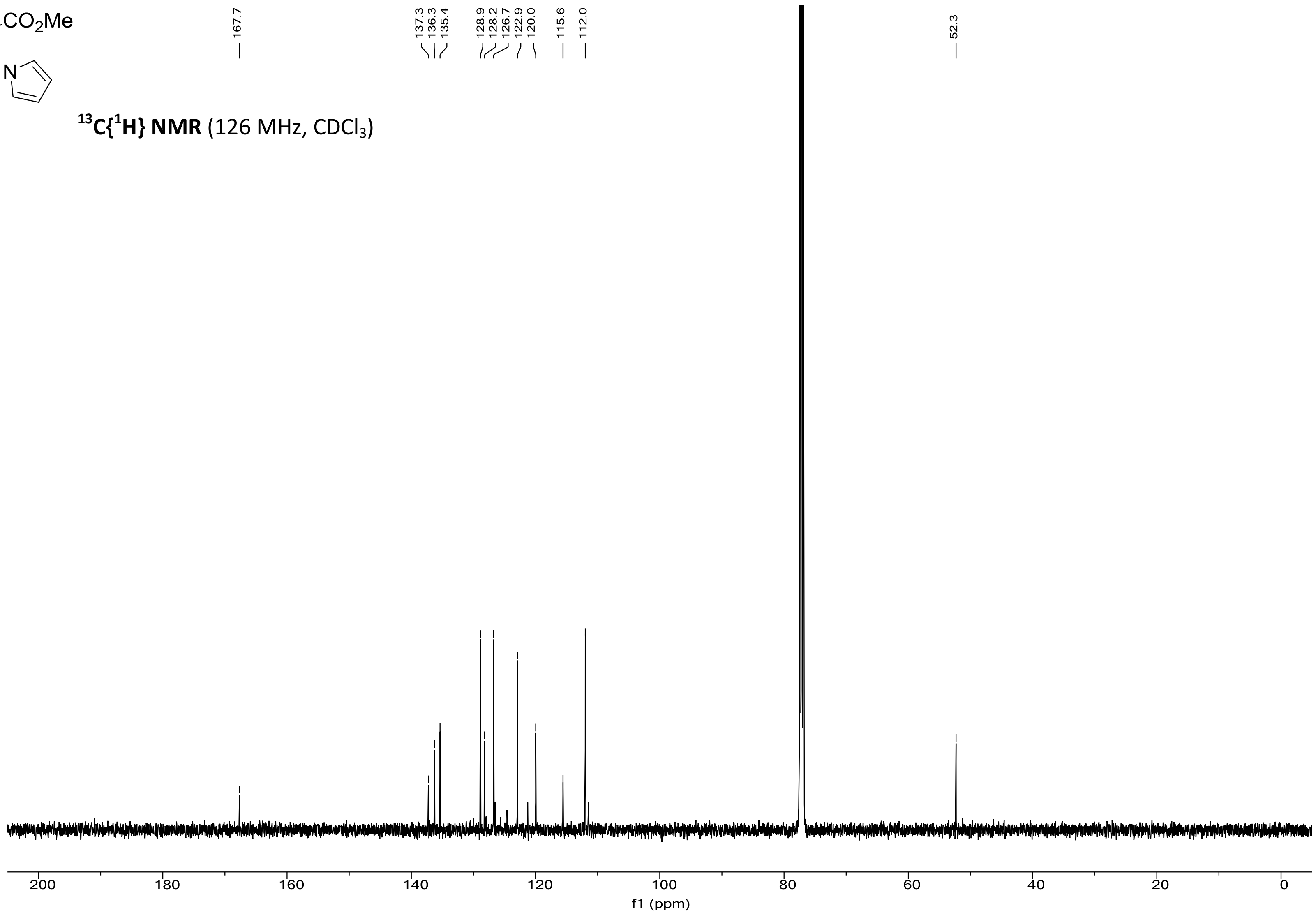

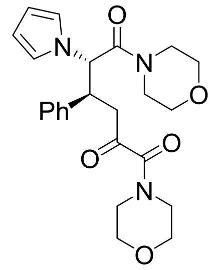

31

^{1}H NMR (500 MHz, $CDCl_3$)

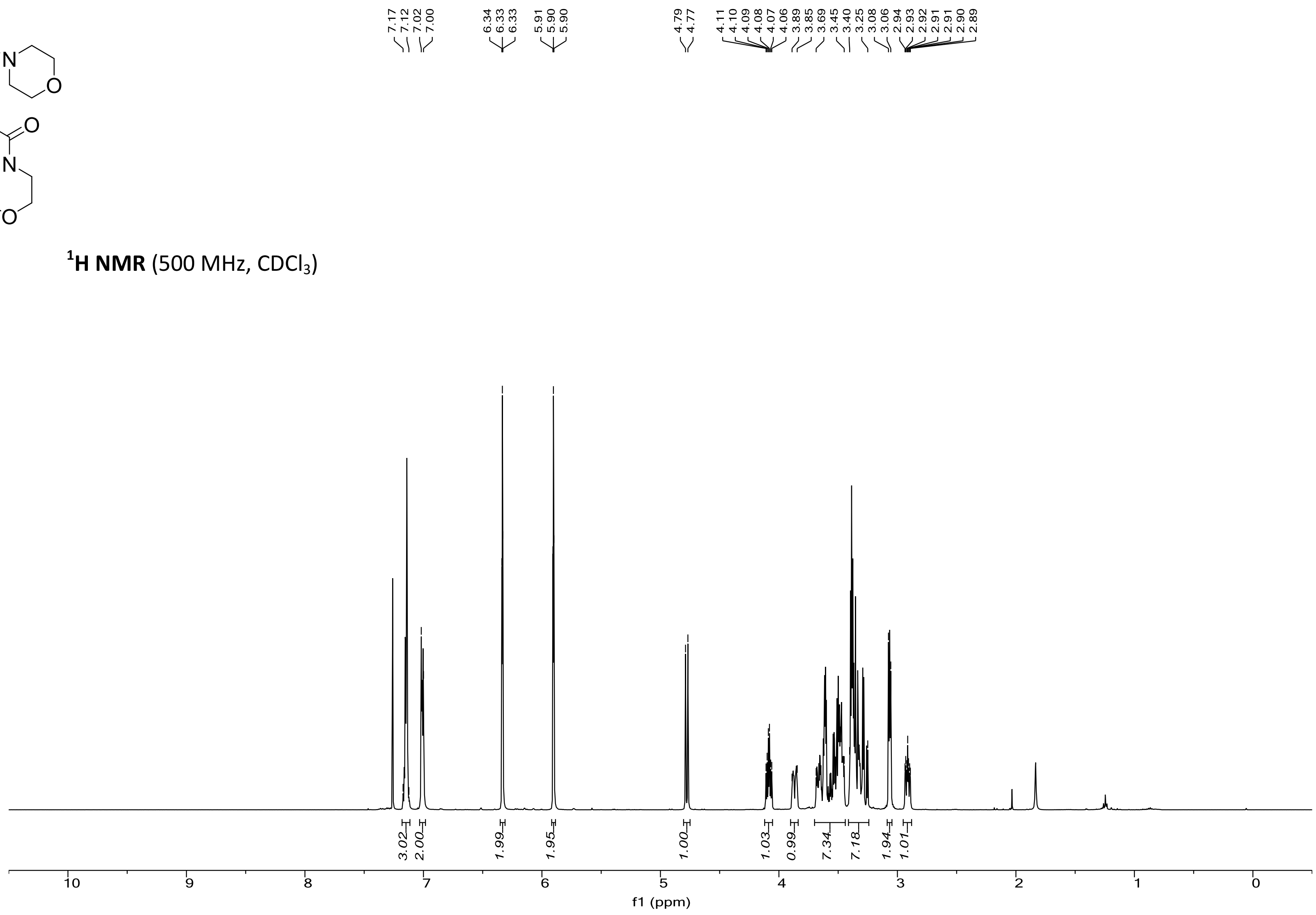

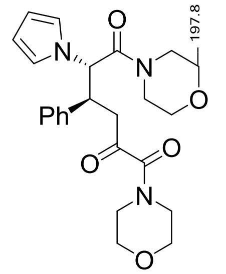

31

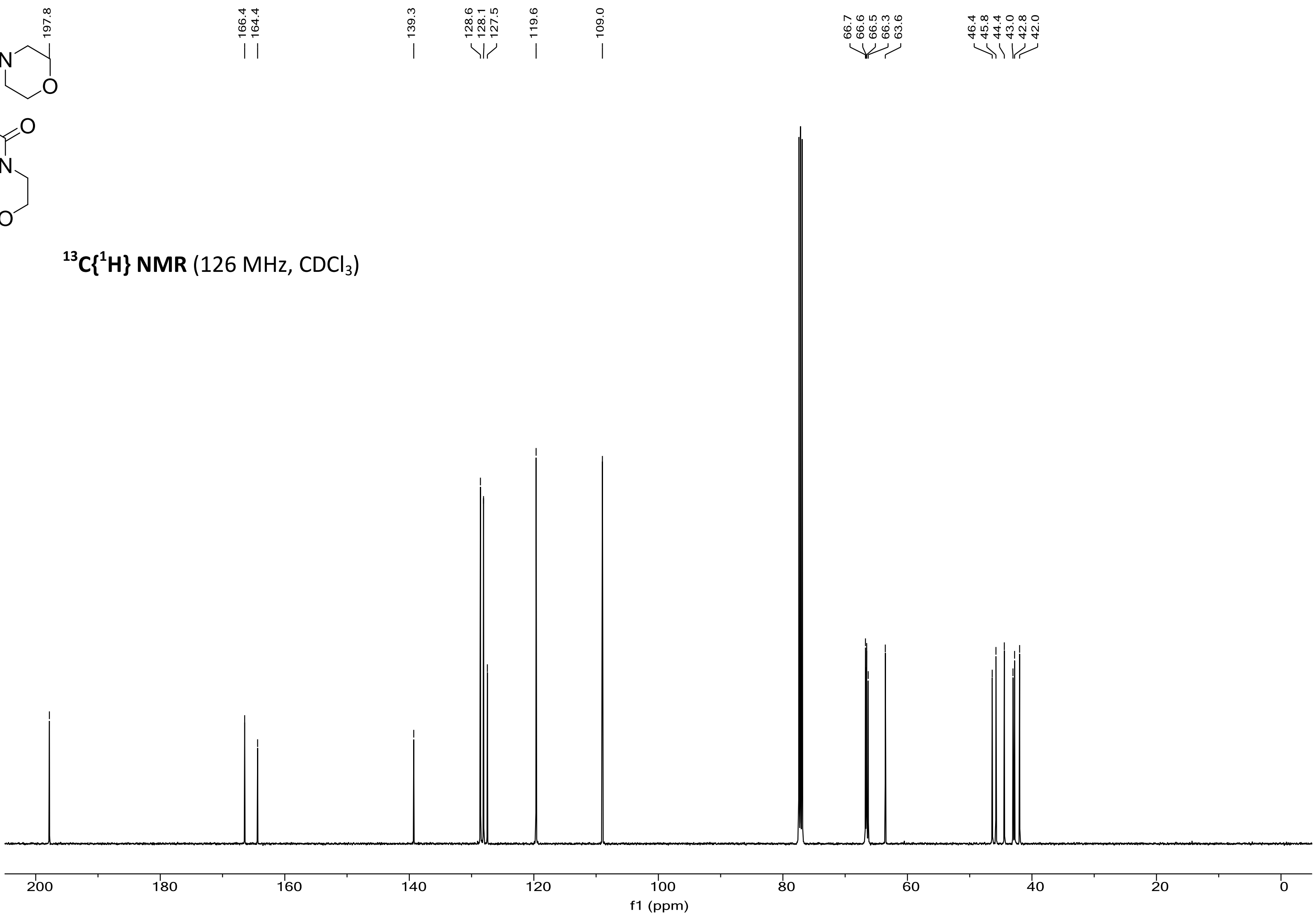

^{13}C{^{1}H} NMR (126 MHz, $CDCl_3$)

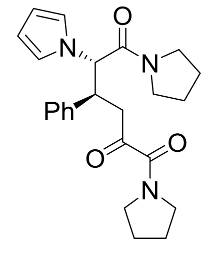

32

^{1}H NMR (500 MHz, $CDCl_3$)

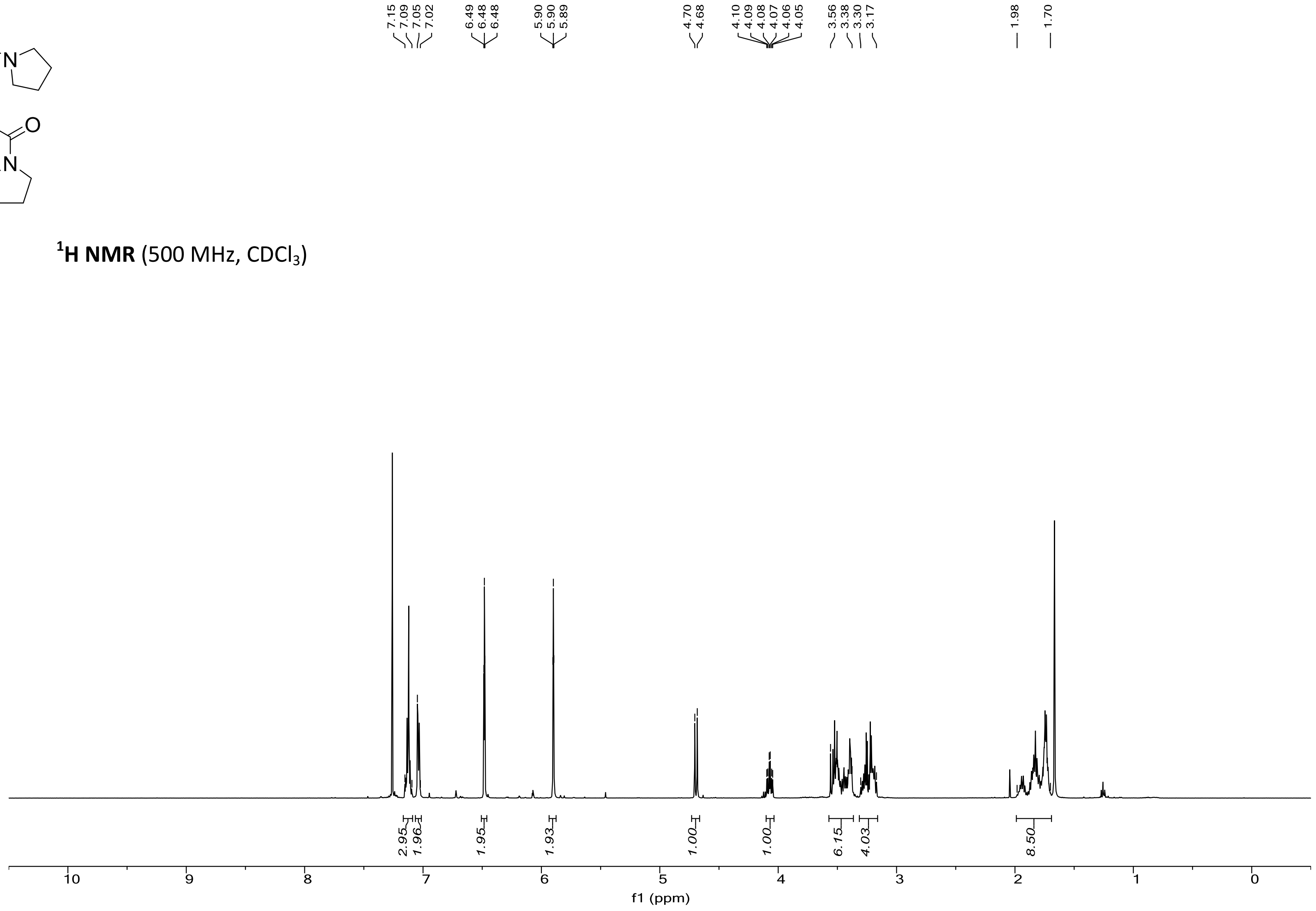

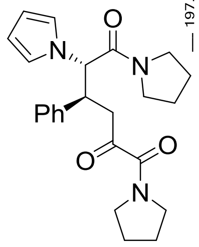

32

^{13}C{^{1}H} NMR (126 MHz, $CDCl_3$)

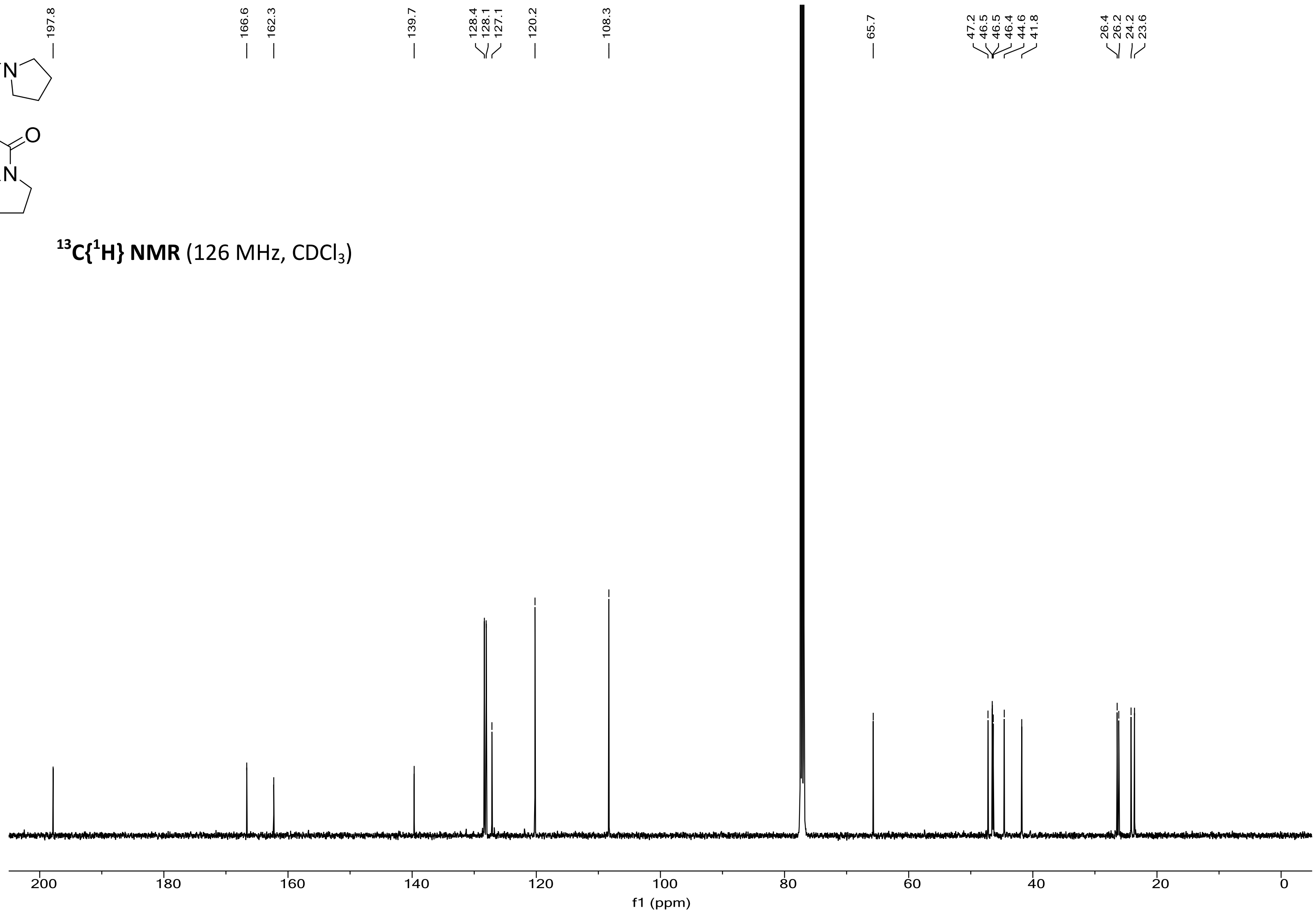

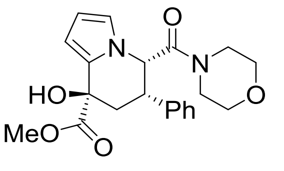

33

^{1}H NMR (500 MHz, $CDCl_3$)

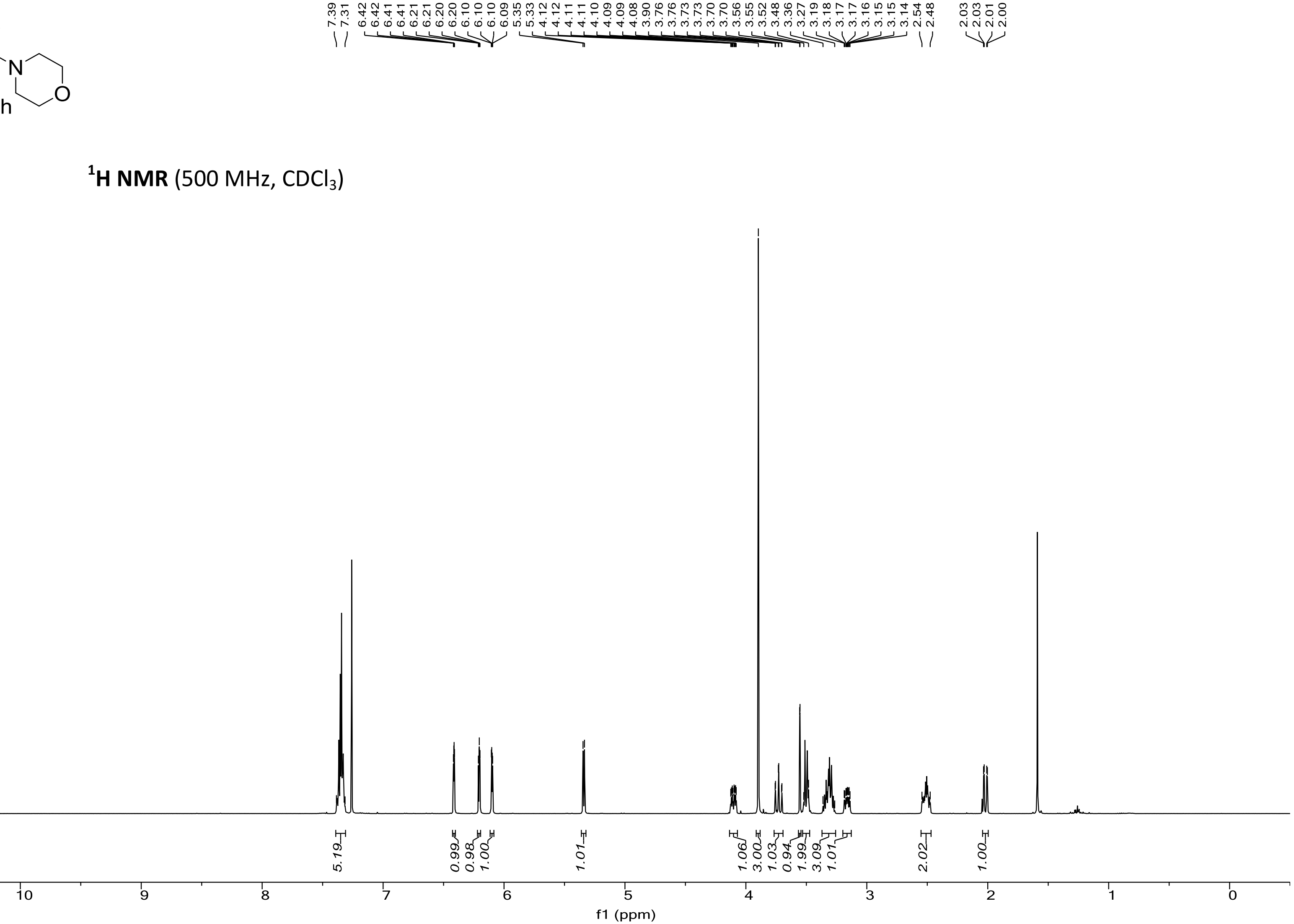

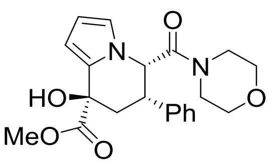

33

^{13}C{^{1}H} NMR (126 MHz, $CDCl_3$)

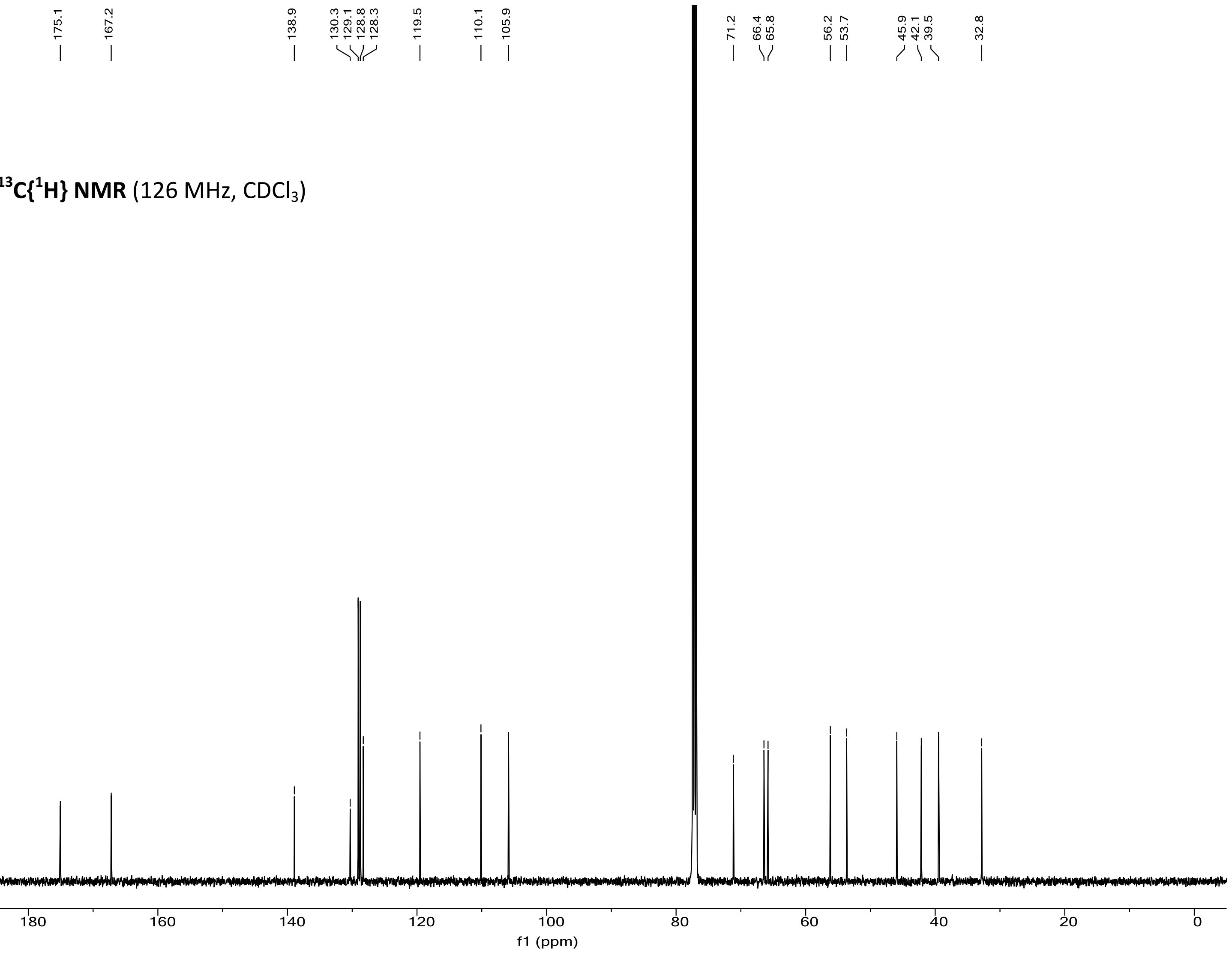

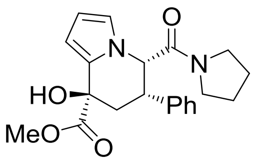

34

^{1}H NMR (500 MHz, CDCl$_3$)

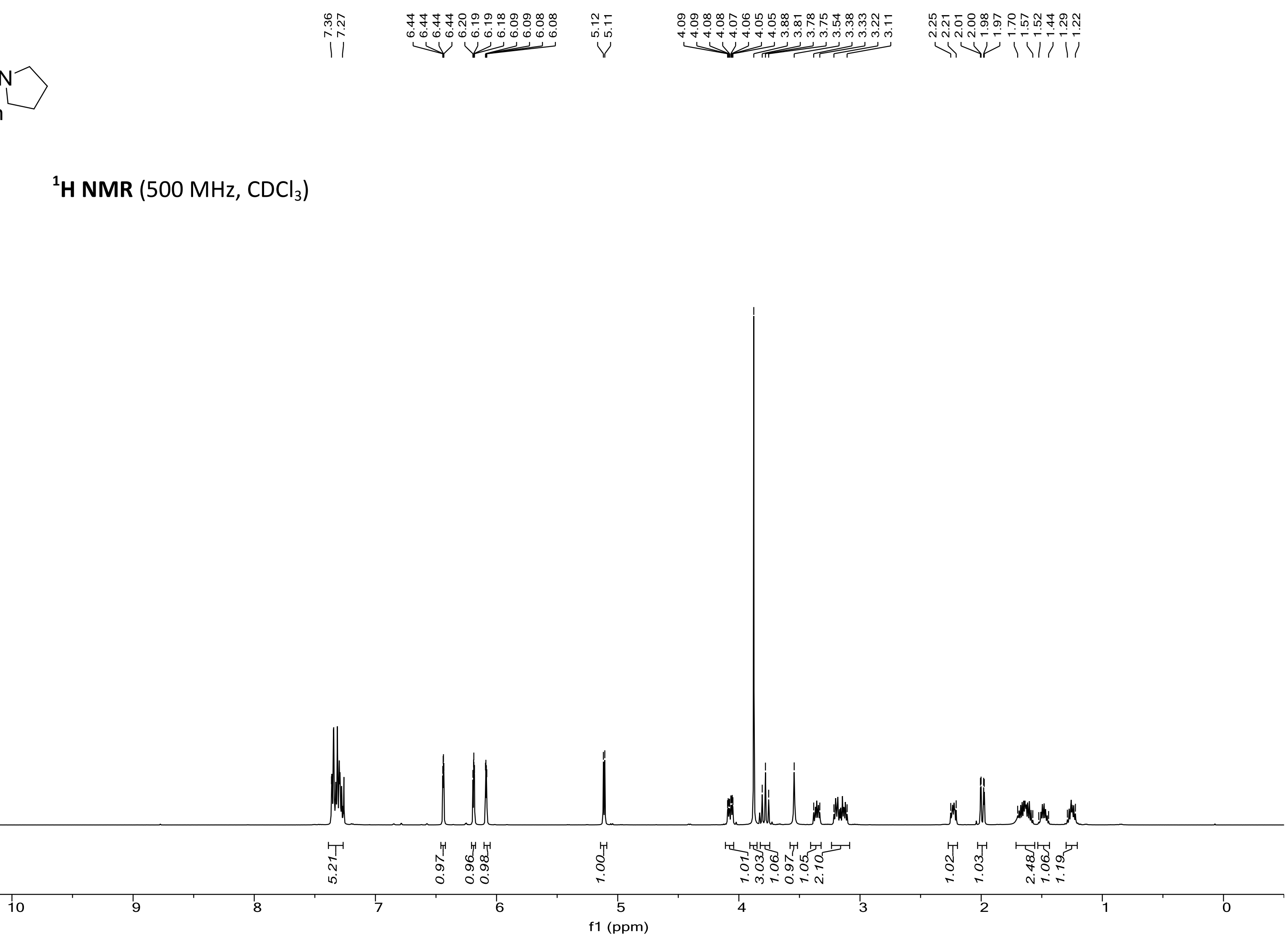

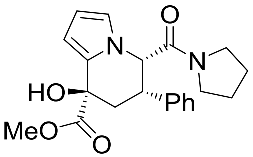

34

^{13}C{^{1}H} NMR (126 MHz, $CDCl_3$)

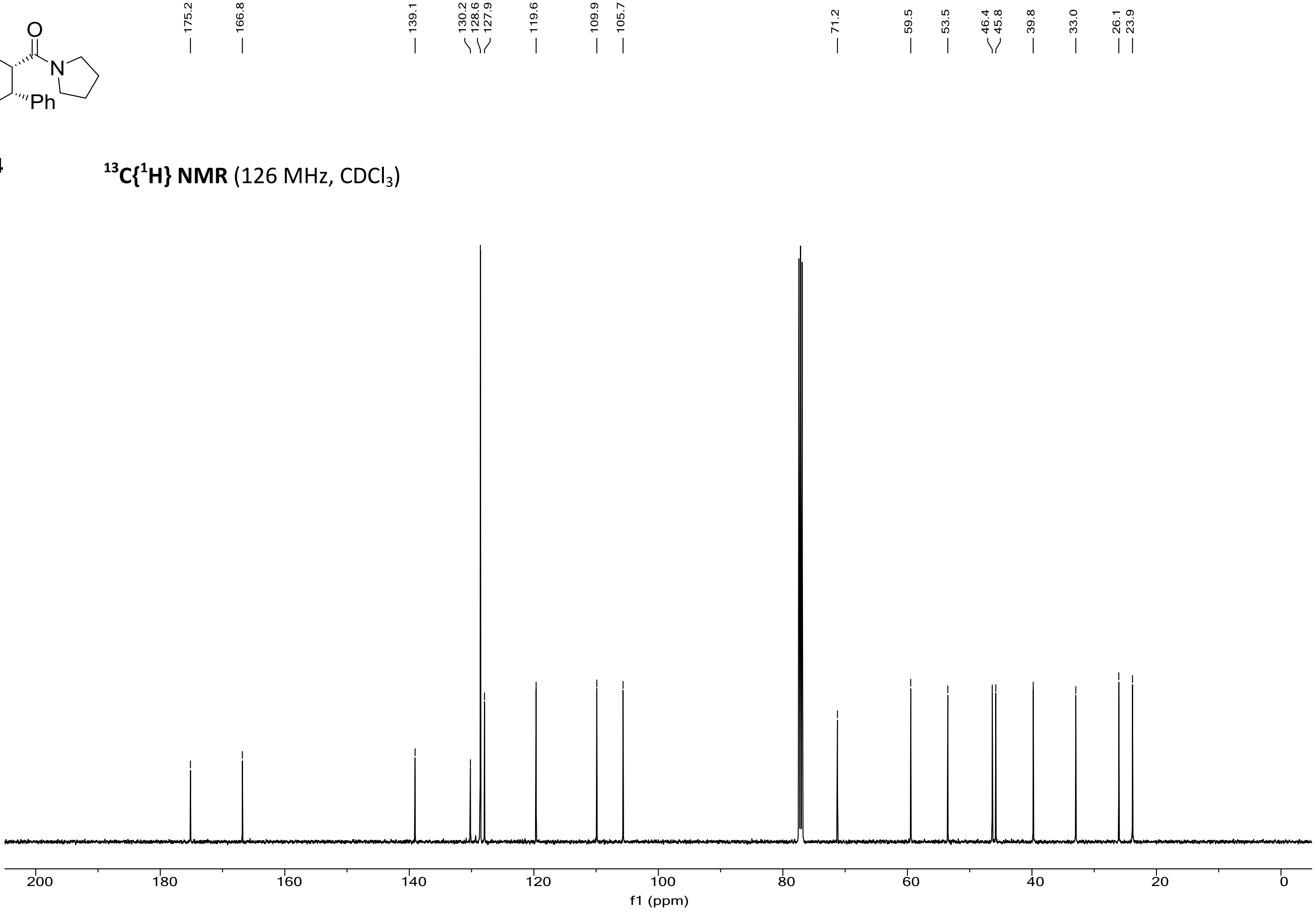

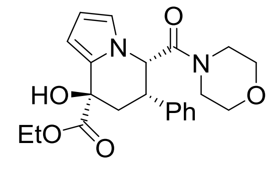

35

^{1}H NMR (500 MHz, $CDCl_3$)

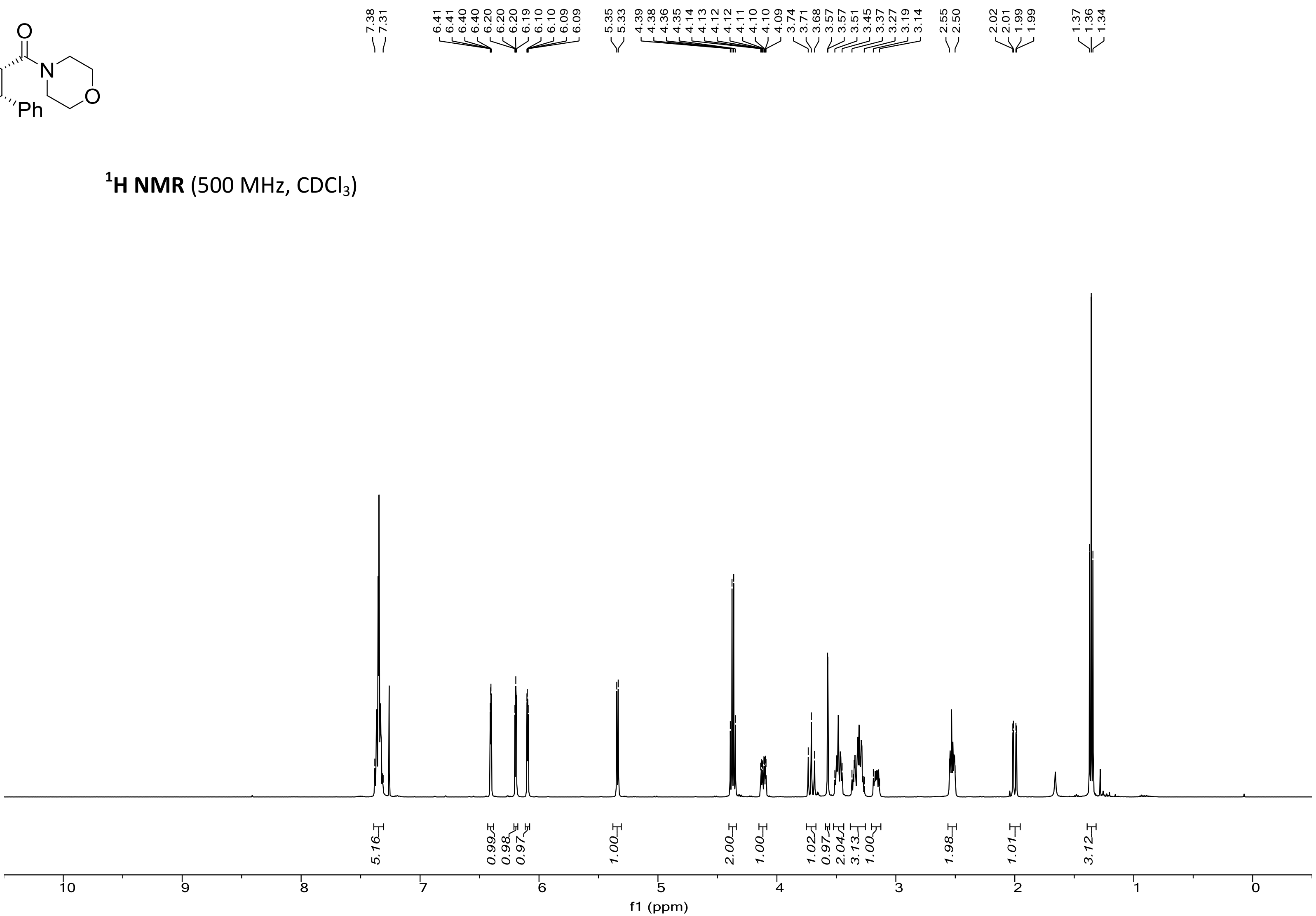

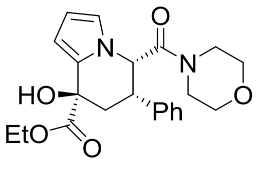

35

$^{13}C\{^{1}H\}$ NMR (126 MHz, $CDCl_3$)

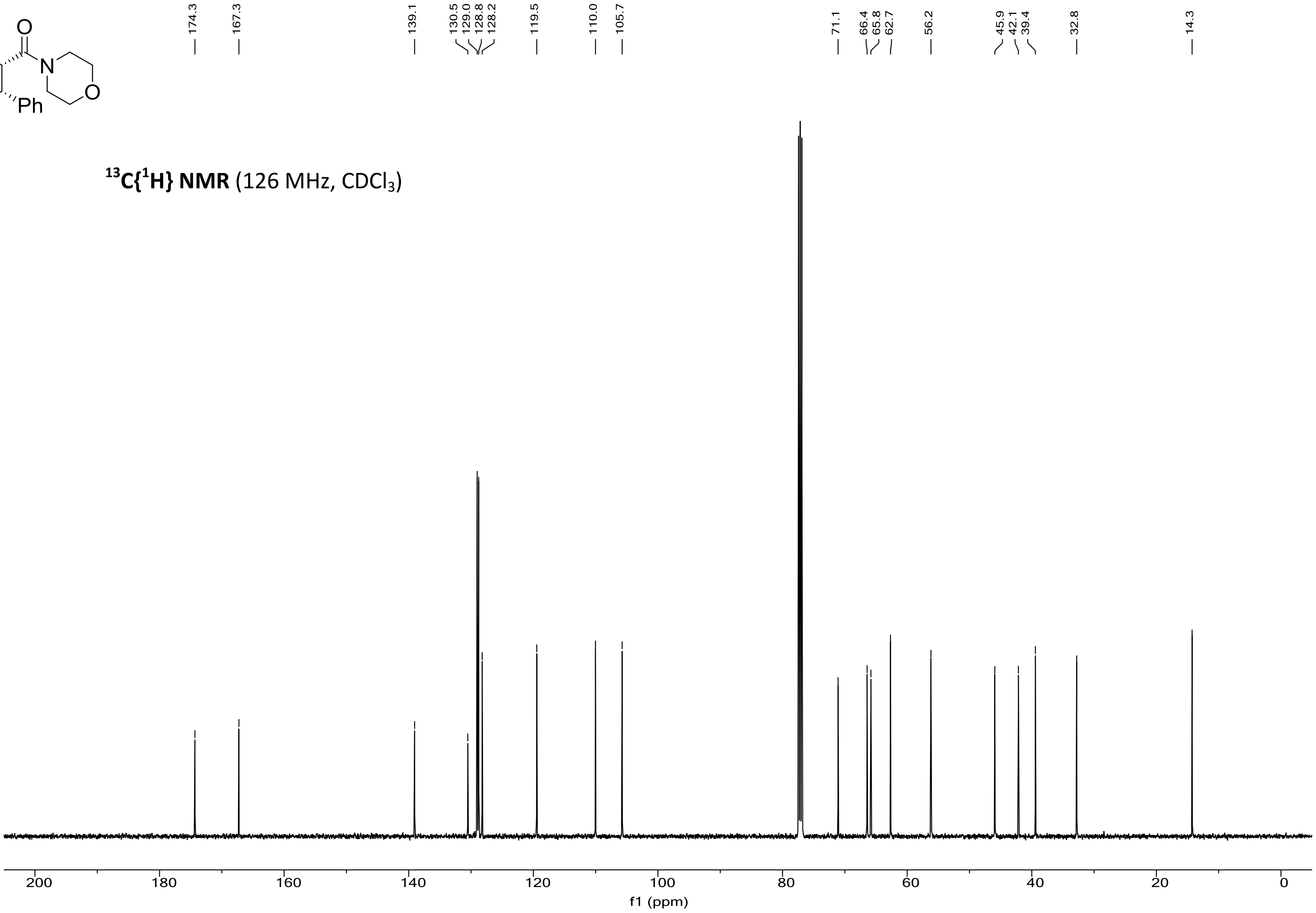

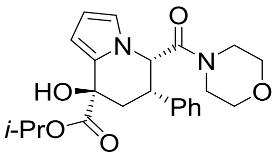

36

^{1}**H NMR** (400 MHz, $CDCl_3$)

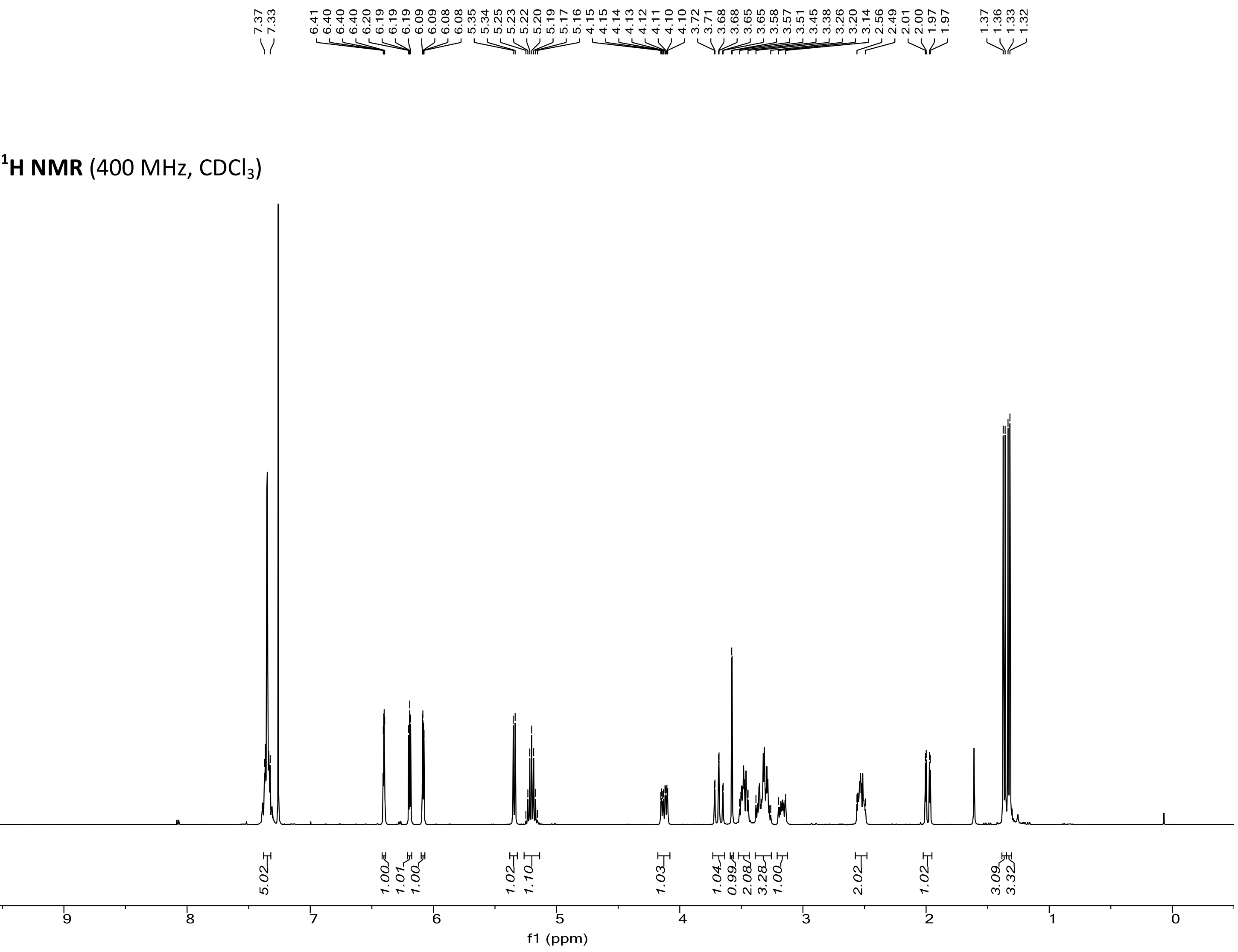

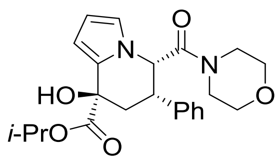

36

^{13}C{^{1}H} NMR (126 MHz, $CDCl_3$)

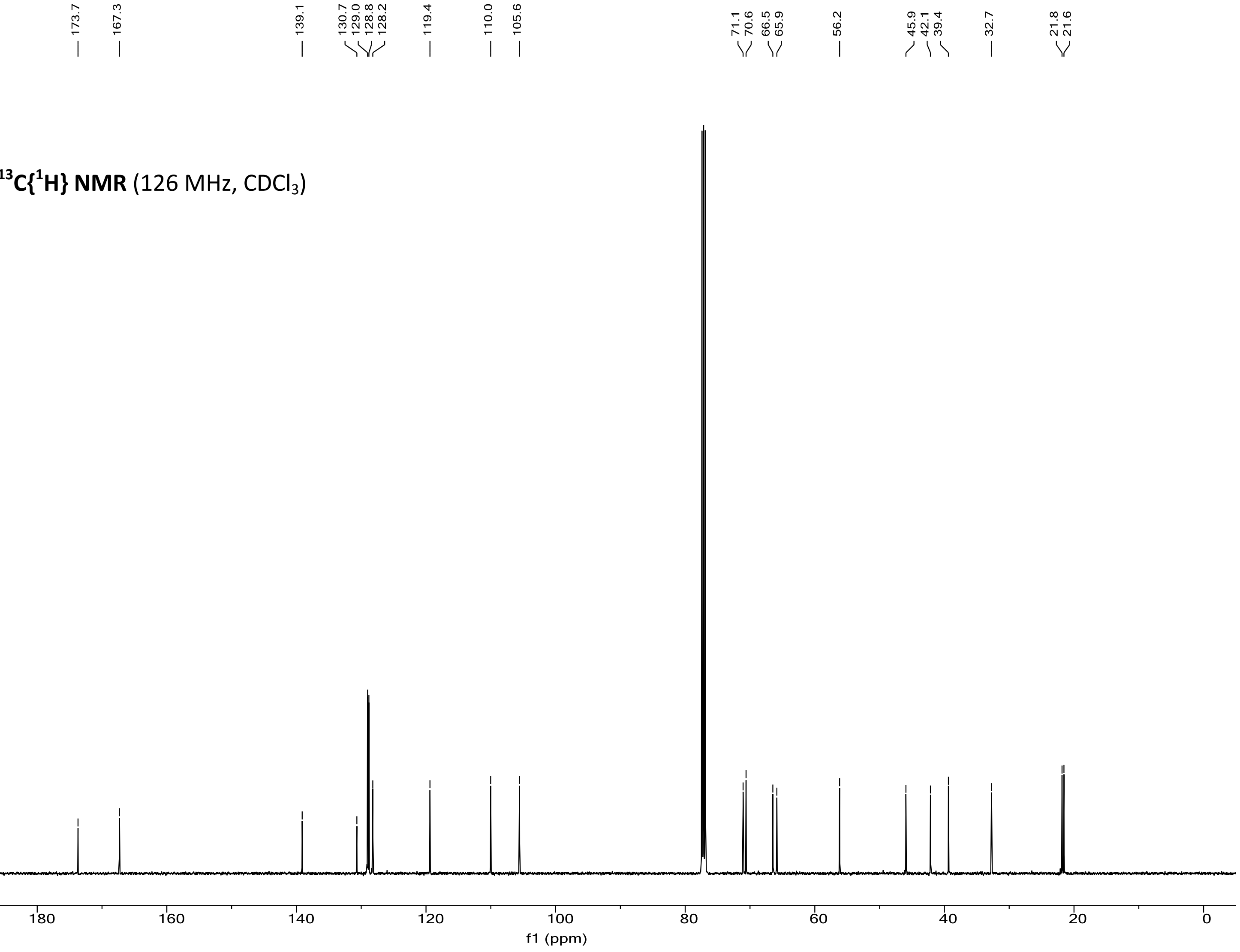

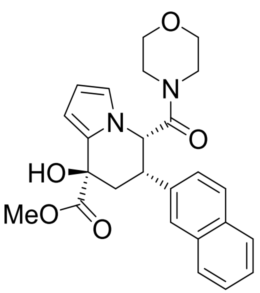

37

^{1}H NMR (500 MHz, $CDCl_3$)

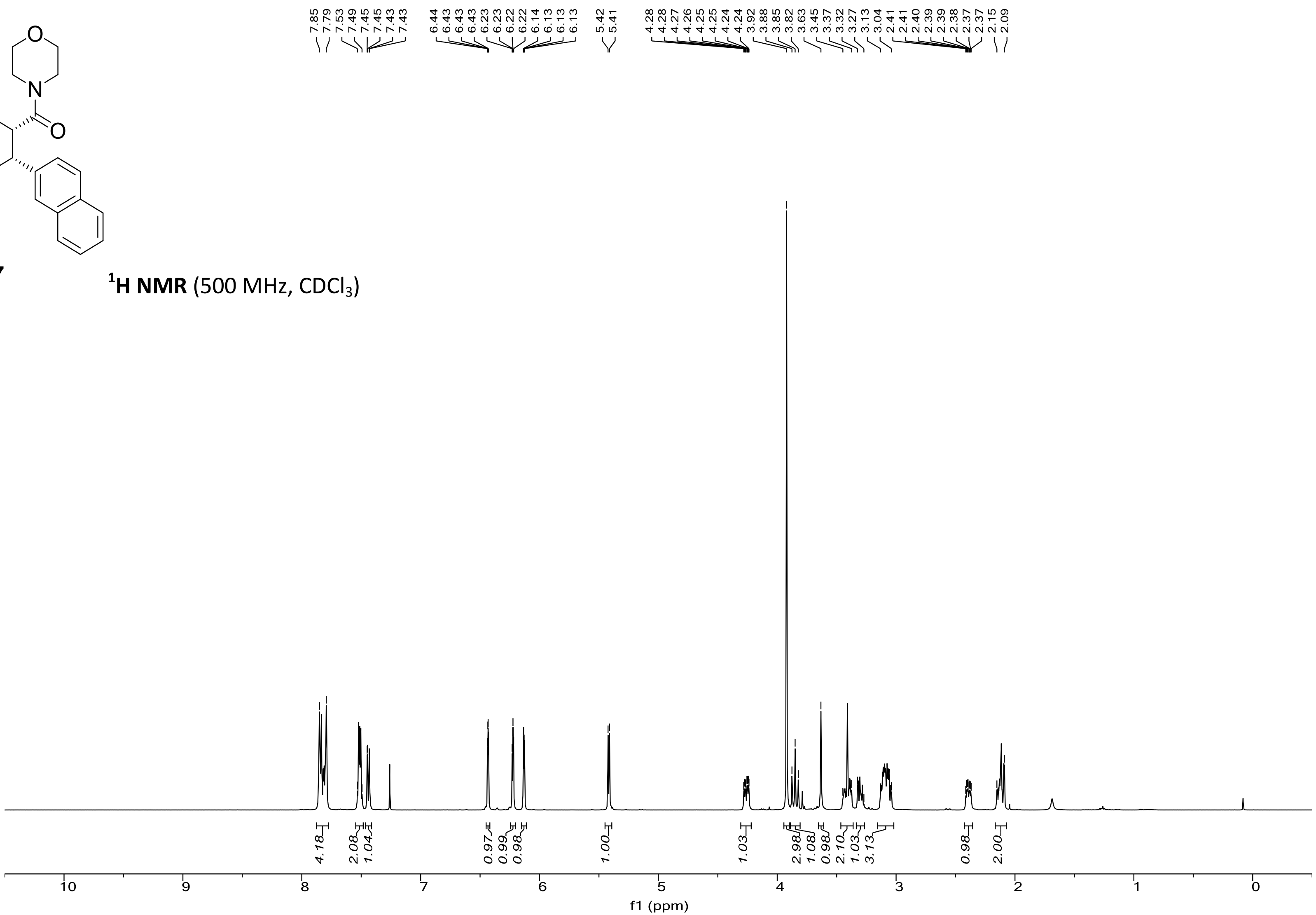

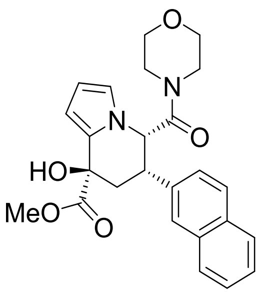

37

$^{13}C\{^1H\}$ **NMR** (126 MHz, $CDCl_3$)

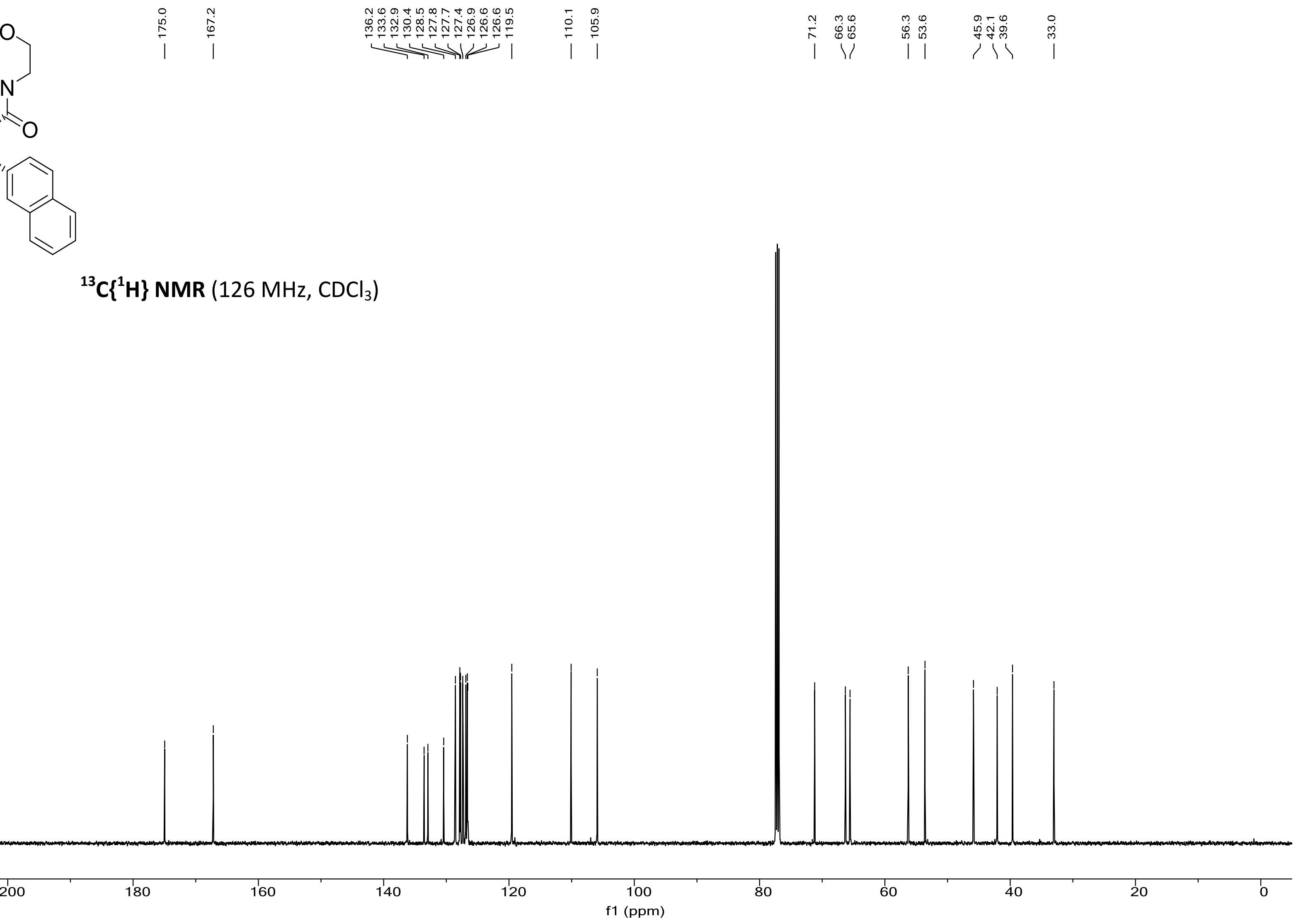

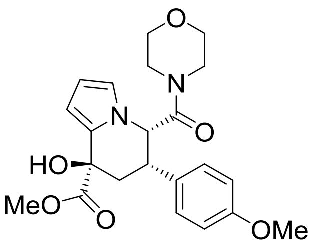

38

[1]**H NMR** (500 MHz, $CDCl_3$)

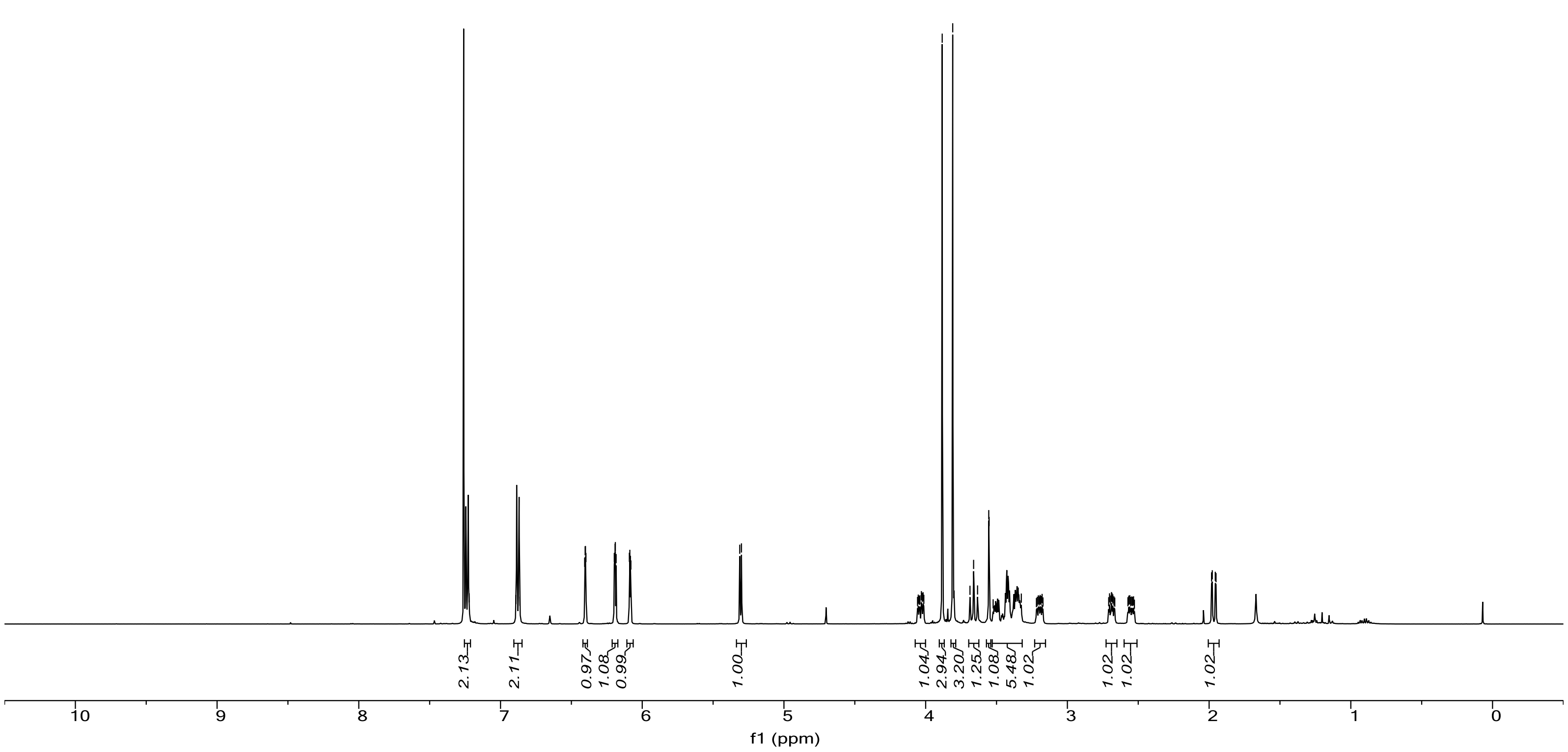

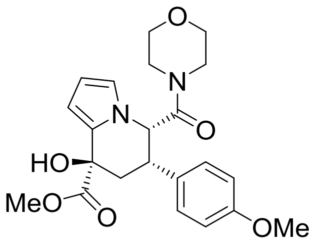

38

$^{13}C\{^{1}H\}$ NMR (126 MHz, $CDCl_3$)

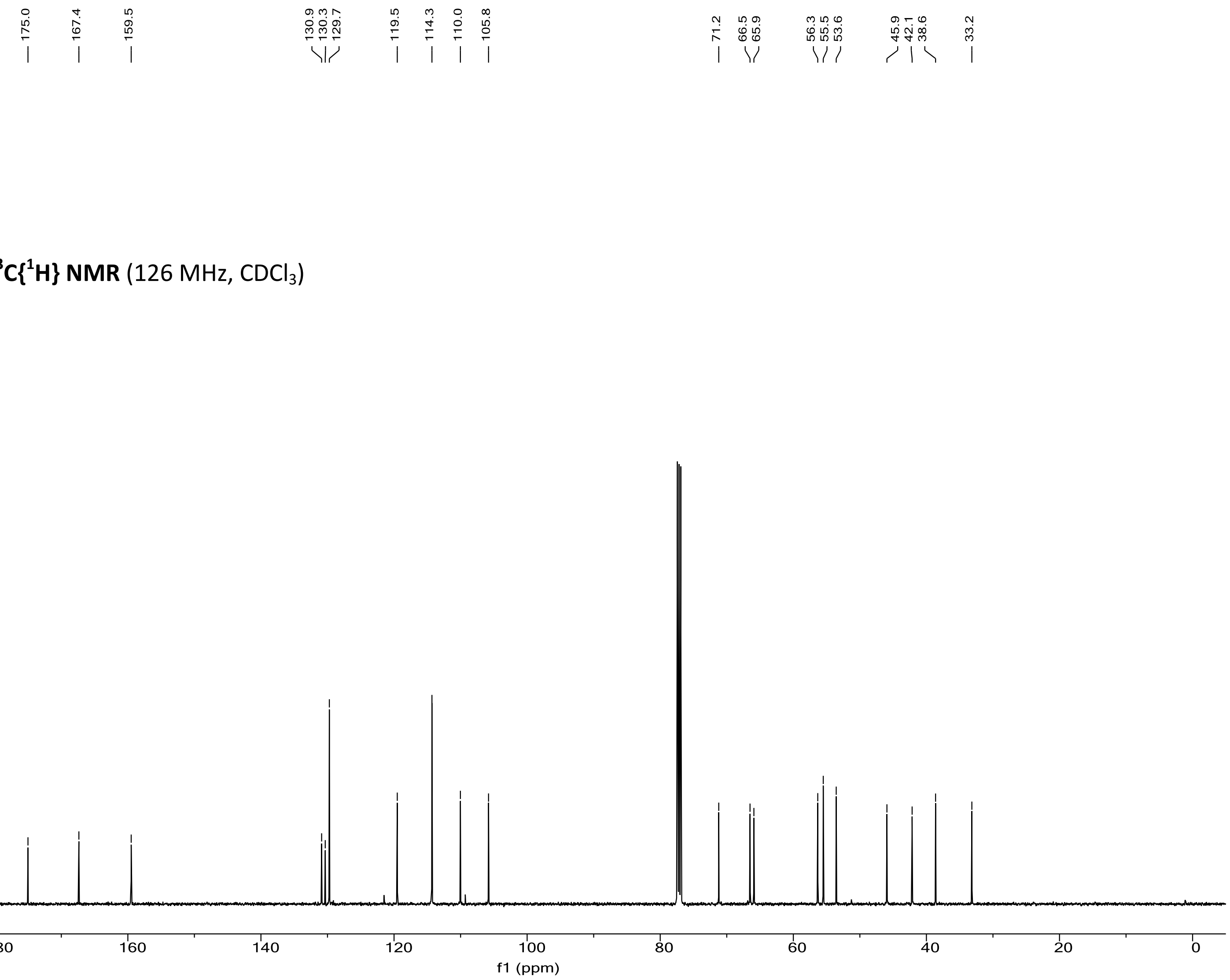

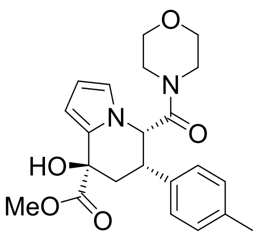

39

^{1}H NMR (500 MHz, $CDCl_3$)

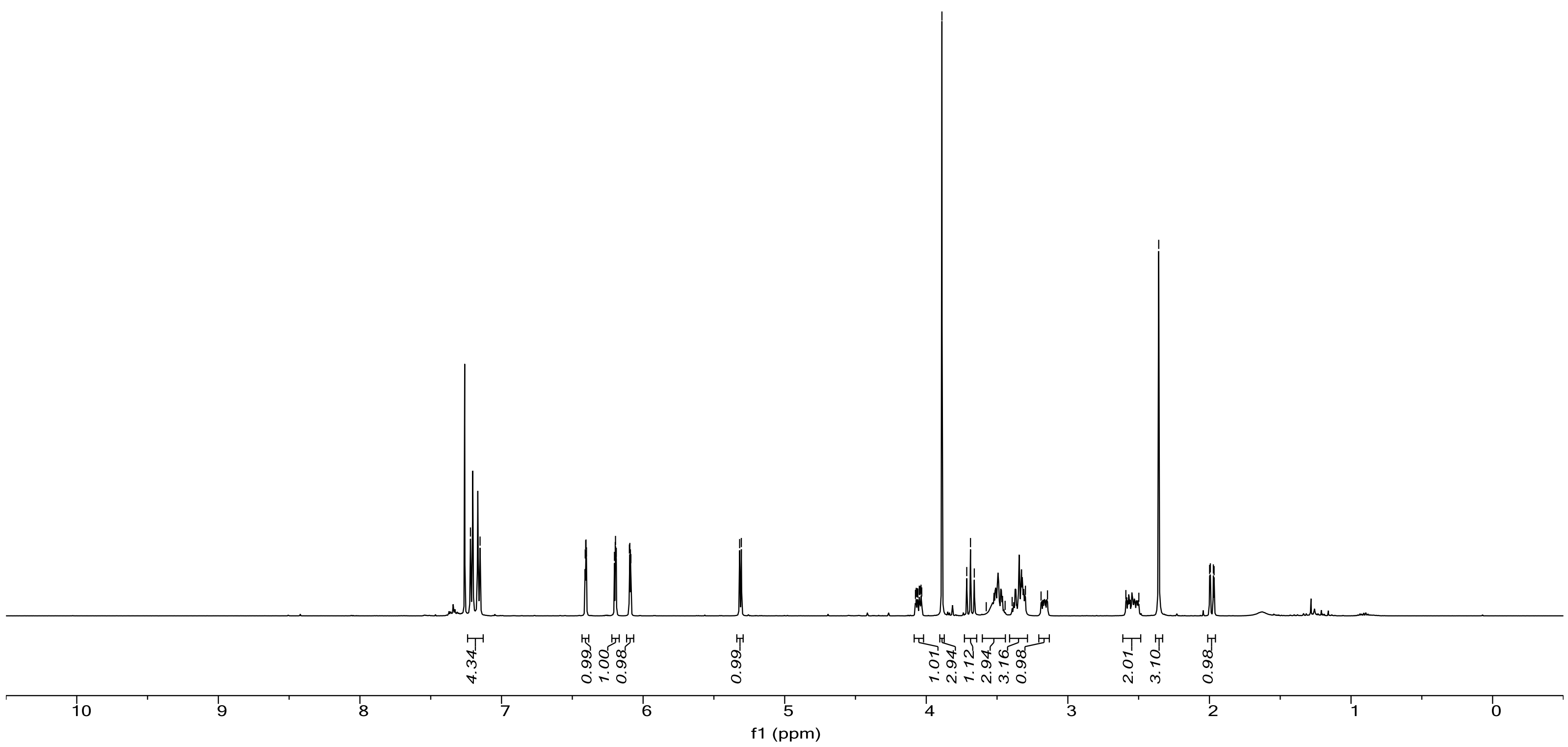

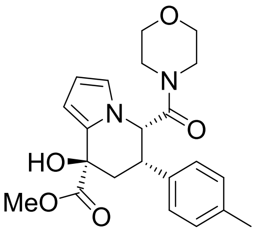

39

^{13}C{^{1}H} NMR (126 MHz, $CDCl_3$)

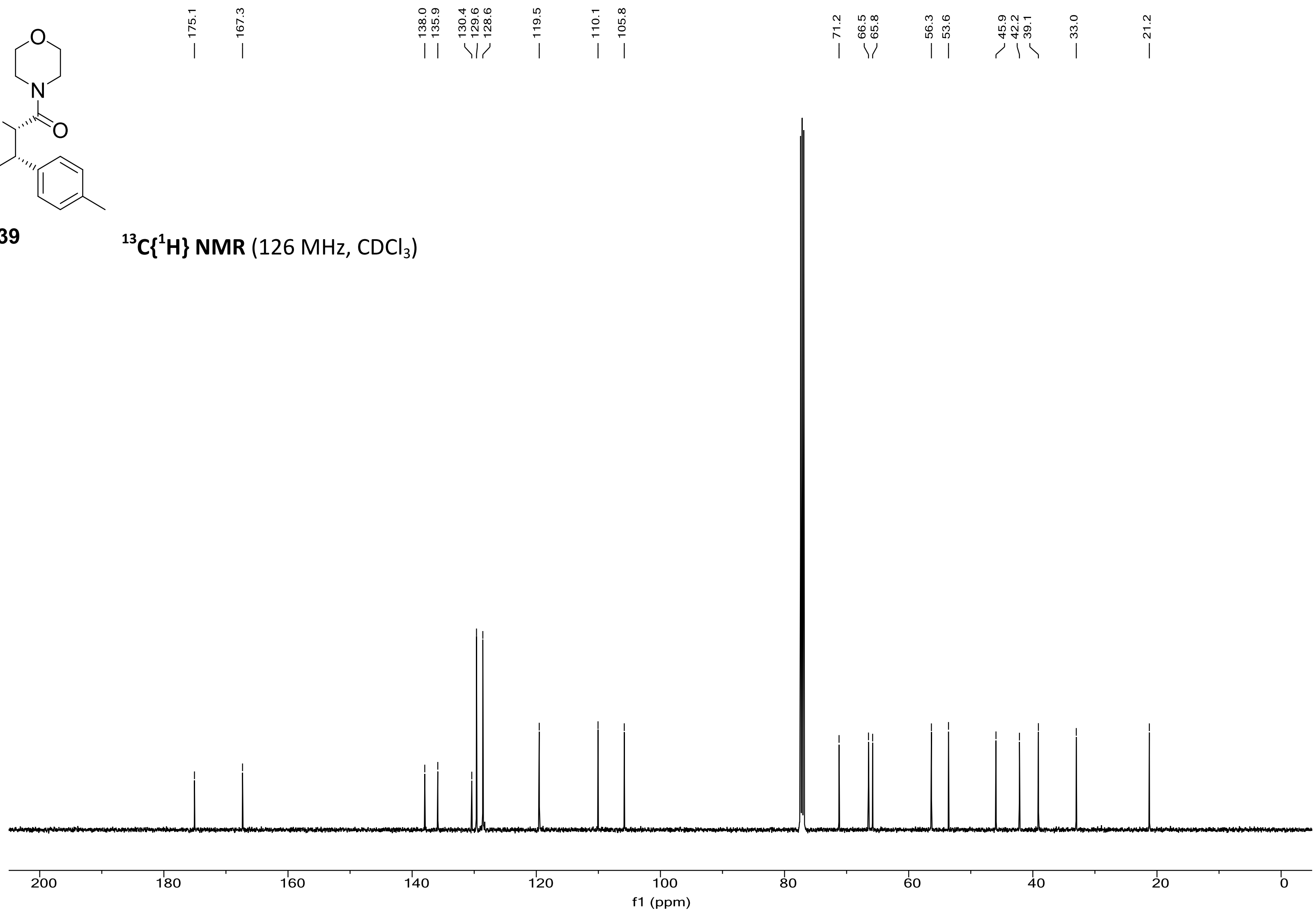

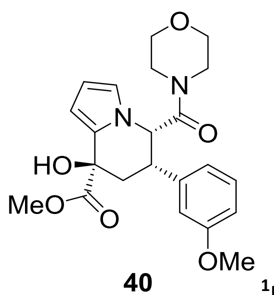

40

^{1}H NMR (500 MHz, $CDCl_3$)

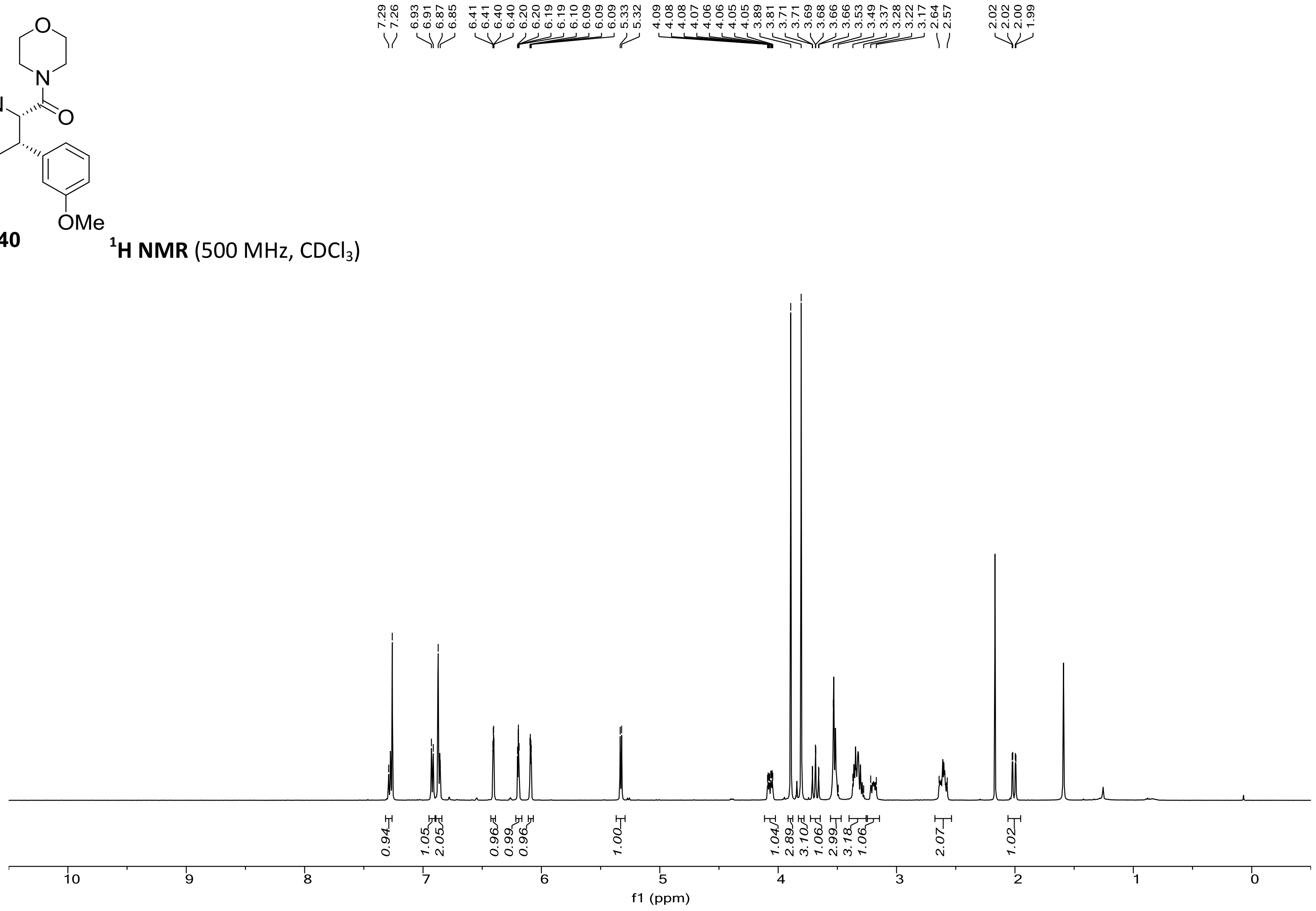

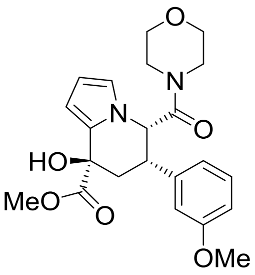

40

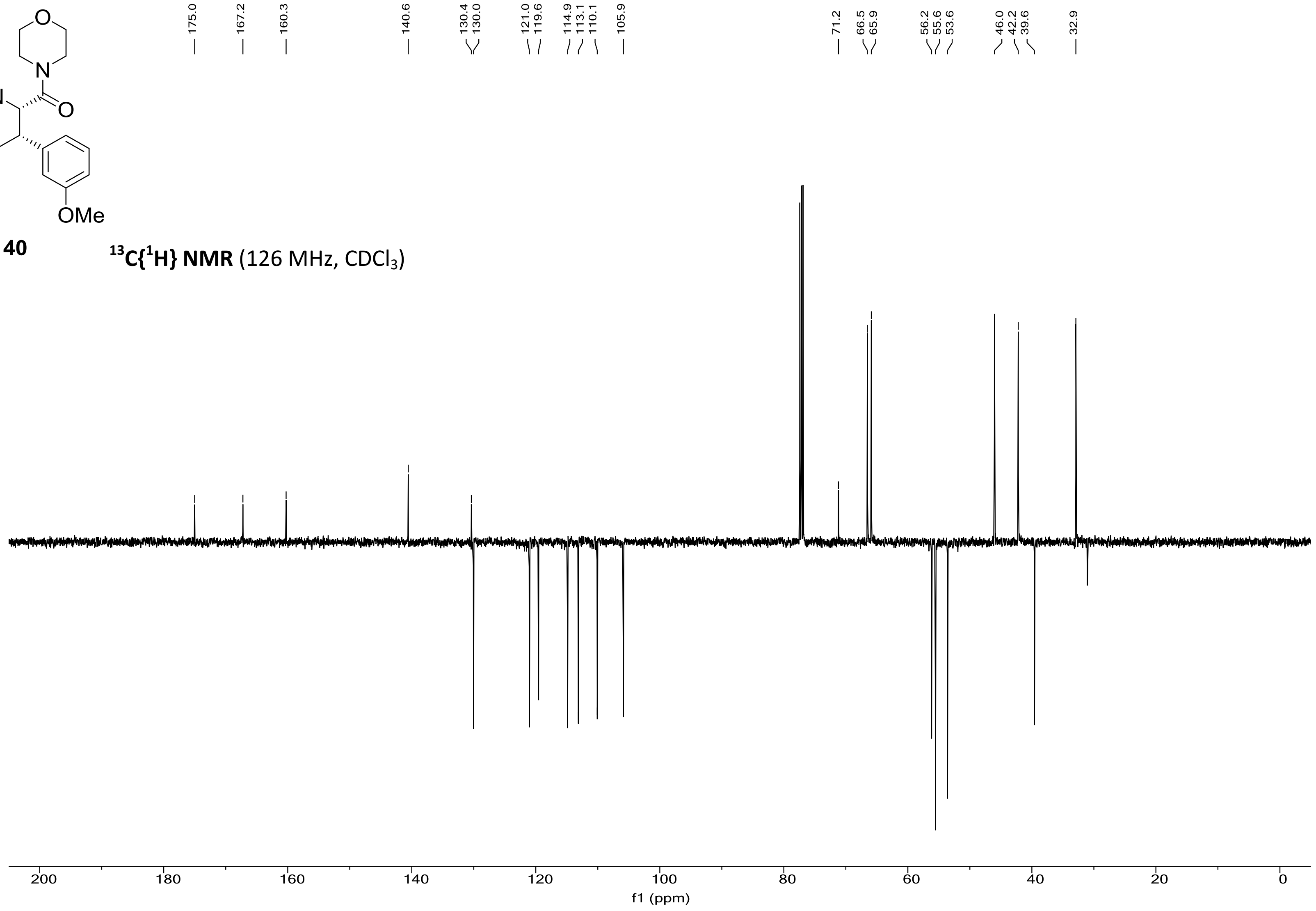

$^{13}C\{^{1}H\}$ NMR (126 MHz, $CDCl_3$)
175.0
167.2
160.3
140.6
130.4
130.0
121.0
119.6
114.9
113.1
110.1
105.9
71.2
66.5
65.9
56.2
55.6
53.6
46.0
42.2
39.6
32.9
200
180
160
140
120
100
80
60
40
20
0
f1 (ppm)

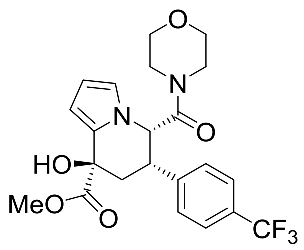

41

^{1}H NMR (500 MHz, $CDCl_3$)

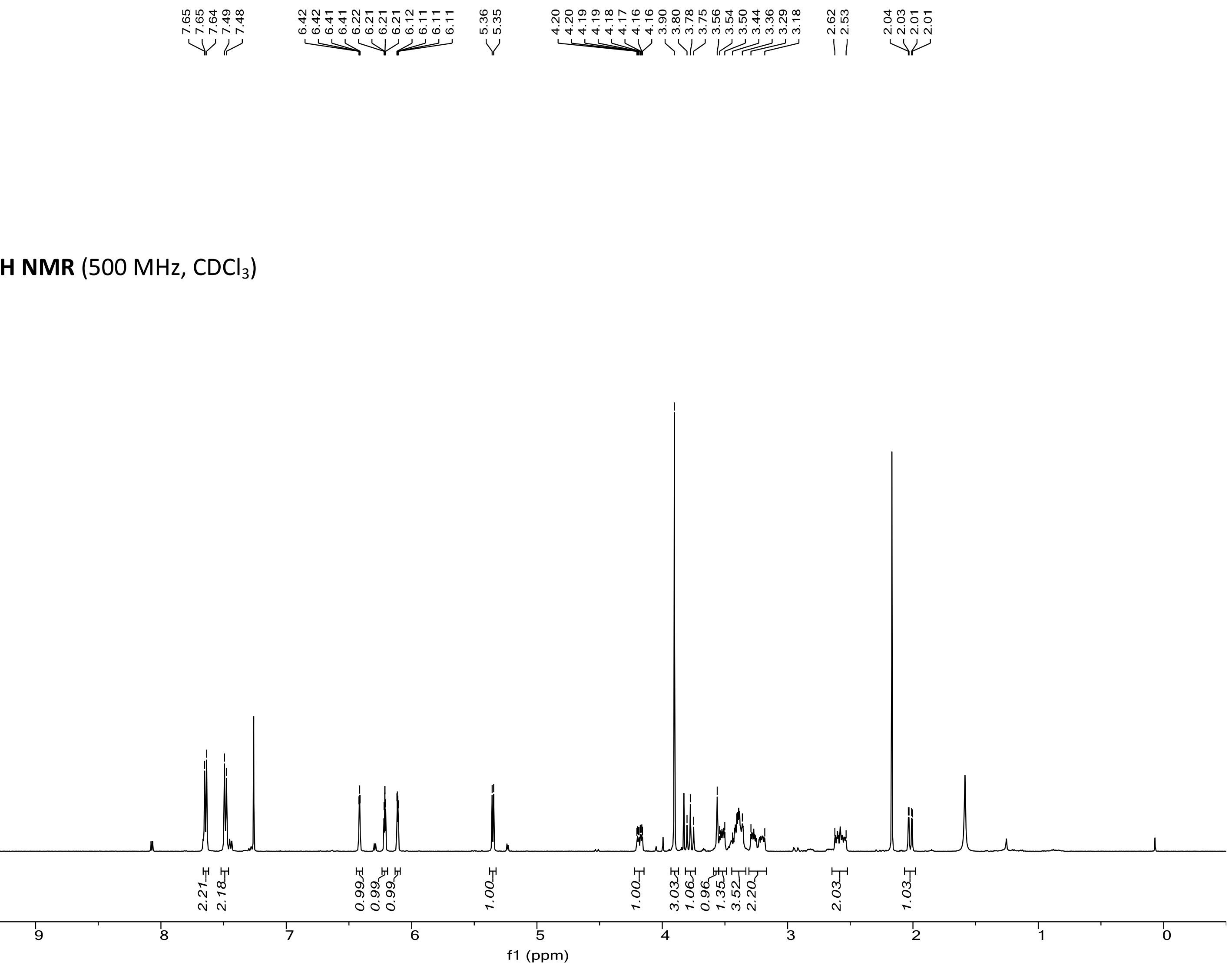

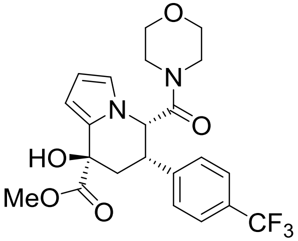

41

$^{13}C\{^{1}H\}$ **NMR** (126 MHz, $CDCl_3$)

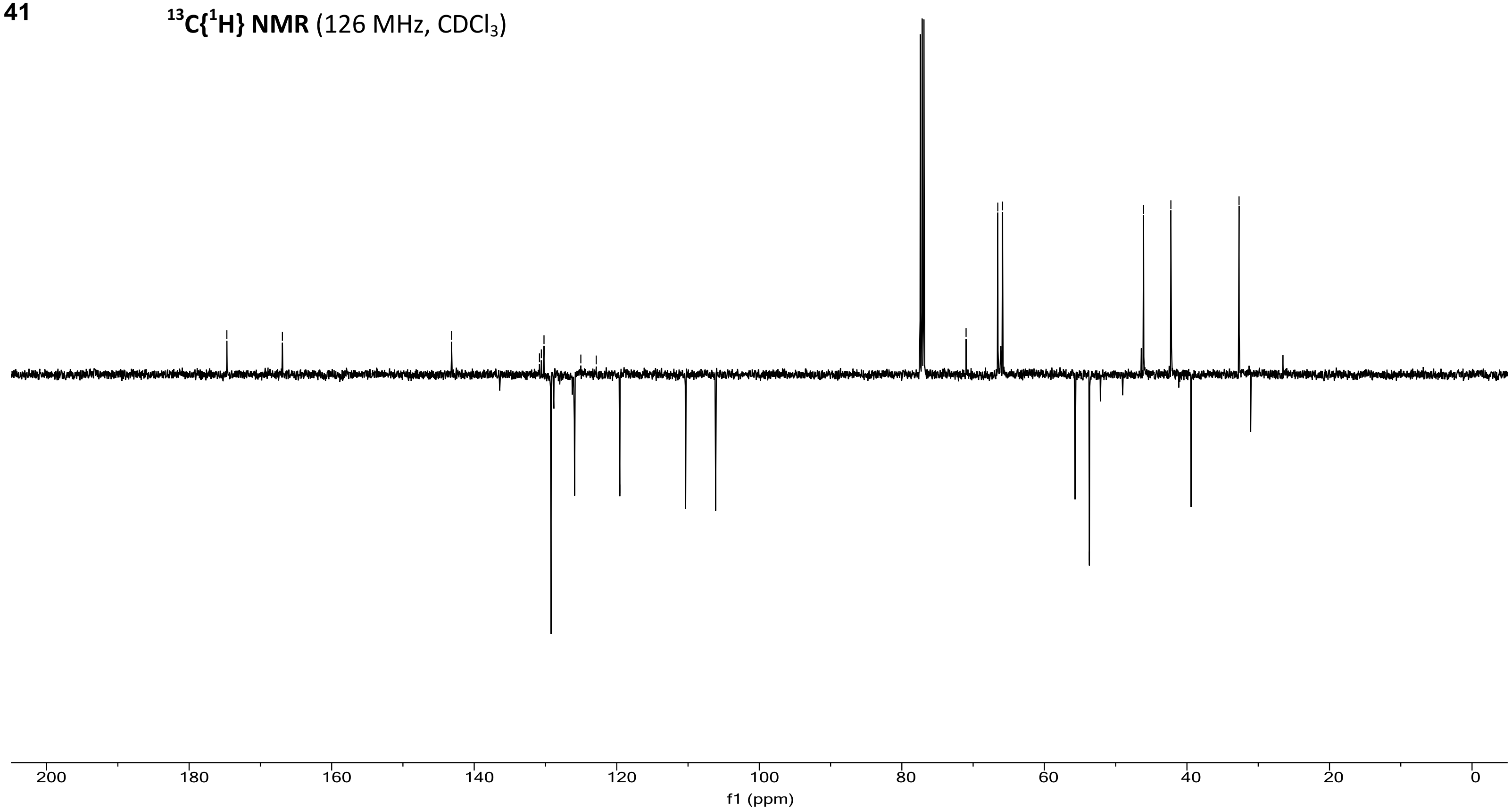

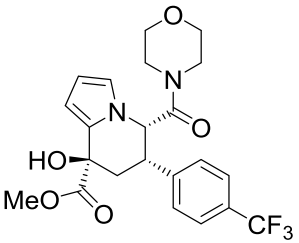

41

^{19}**F NMR** (471 MHz, $CDCl_3$)

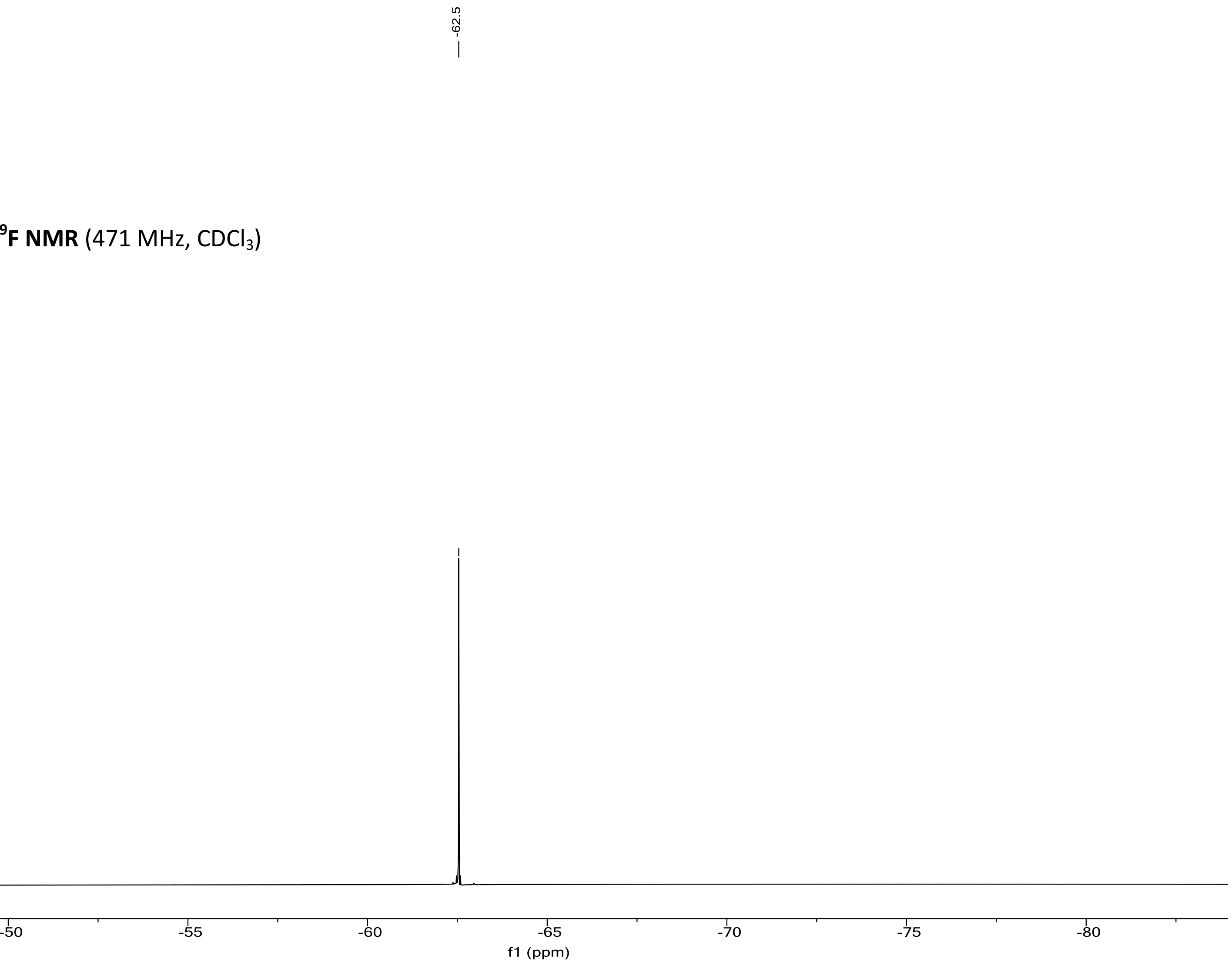

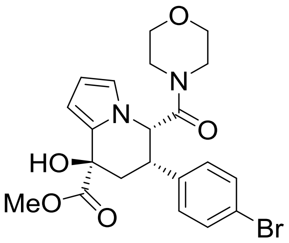

42

^{1}H NMR (500 MHz, $CDCl_3$)

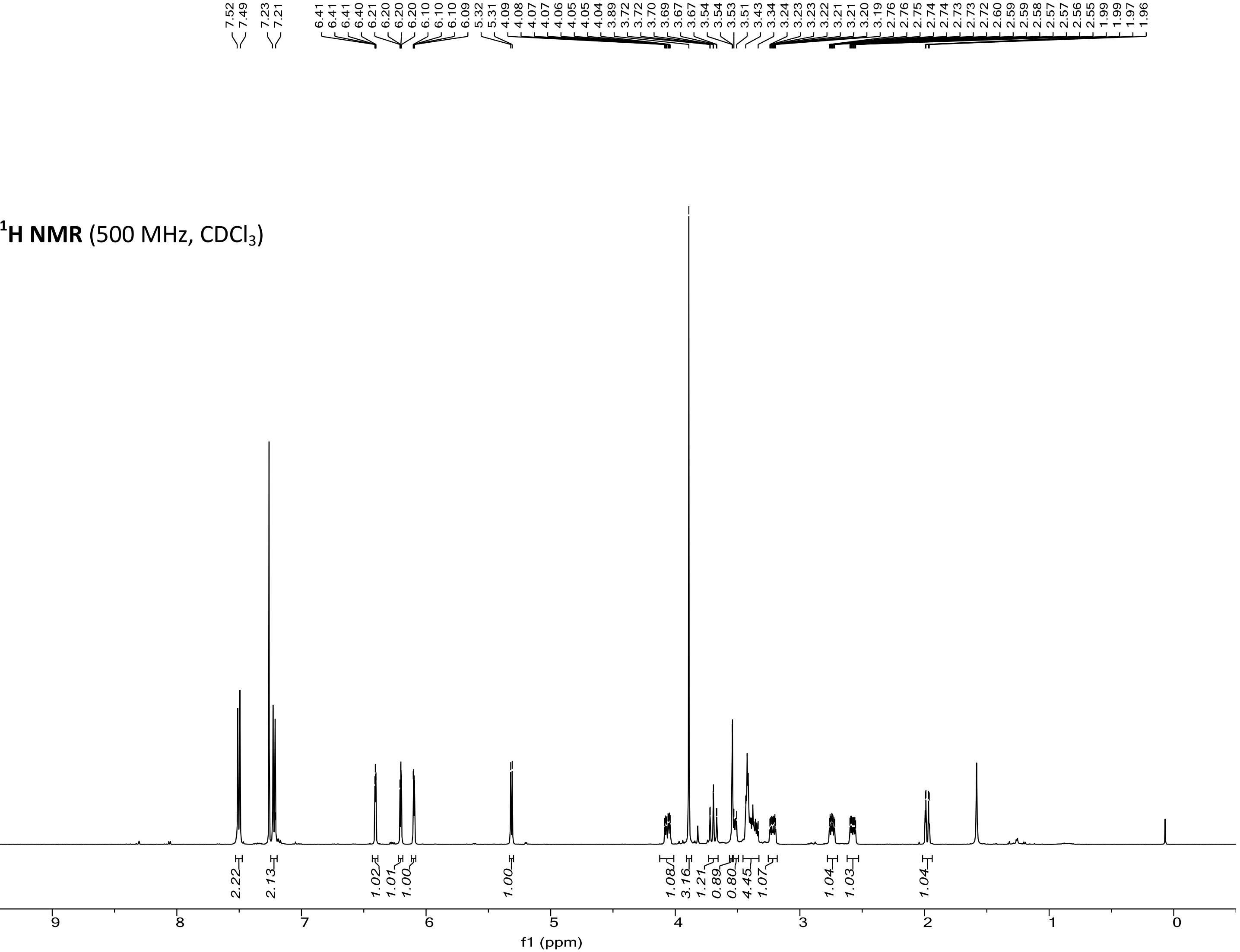

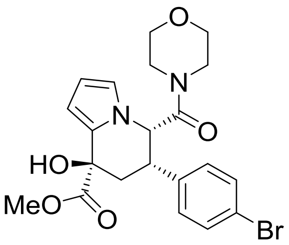

42

^{13}**C{^{1}H} NMR** (126 MHz, $CDCl_3$)

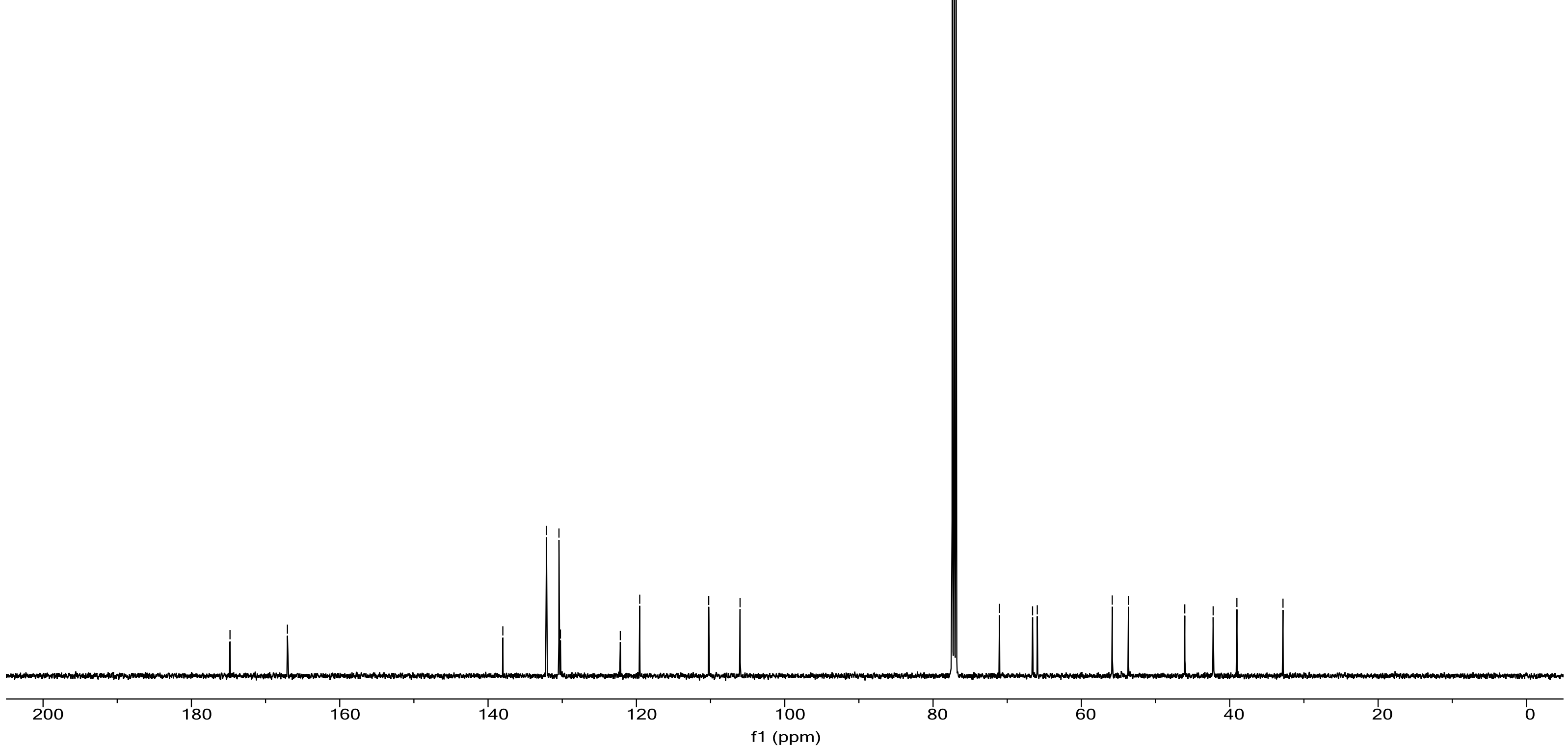

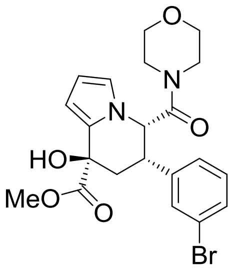

43

^{1}H NMR (500 MHz, $CDCl_3$)

~95% purity (grease contaminant from petroleum ether)

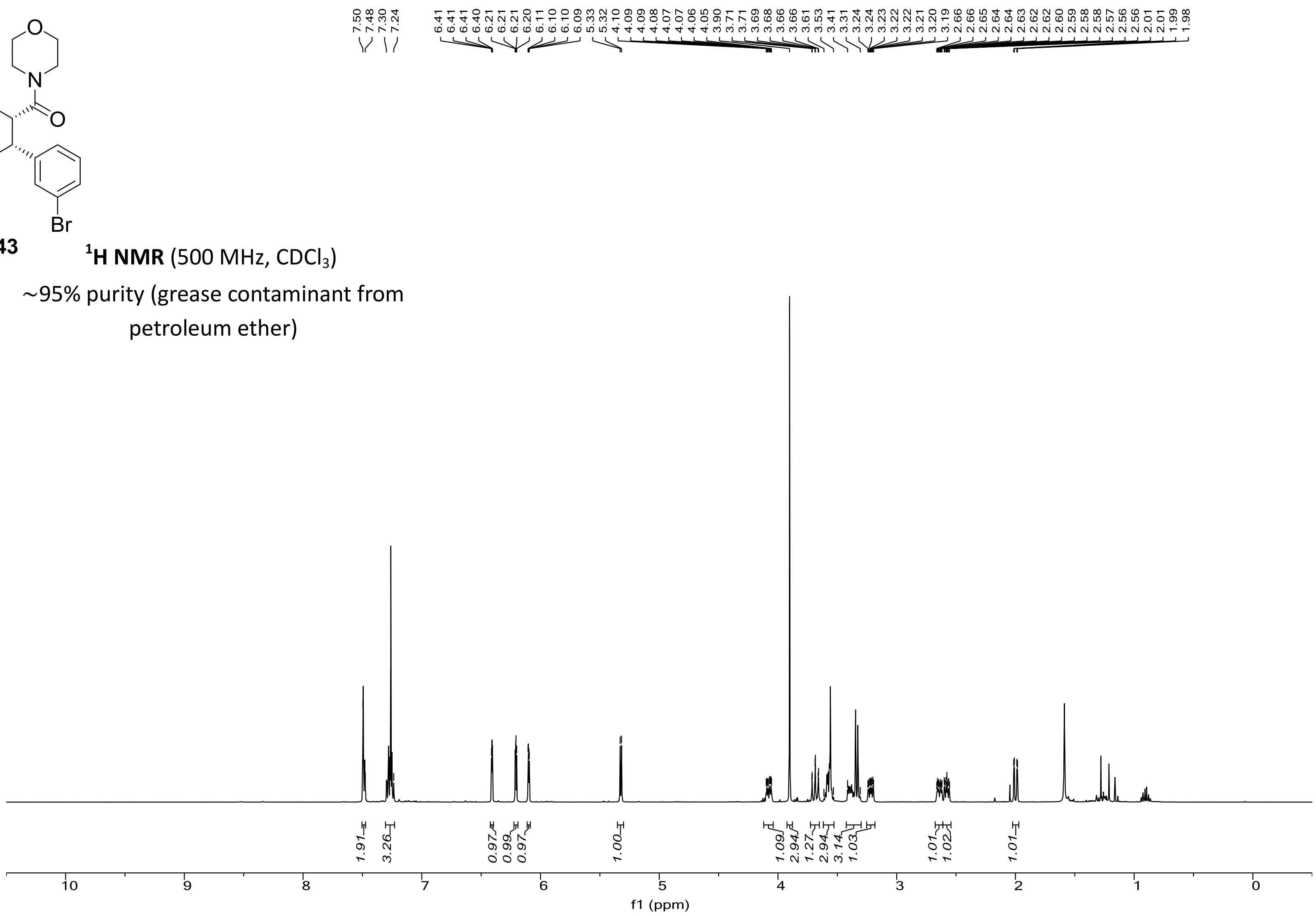

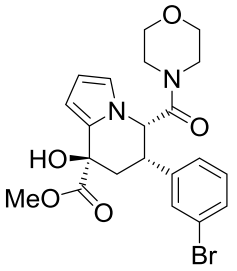

43

^{13}**C{^{1}H} NMR** (126 MHz, $CDCl_3$)

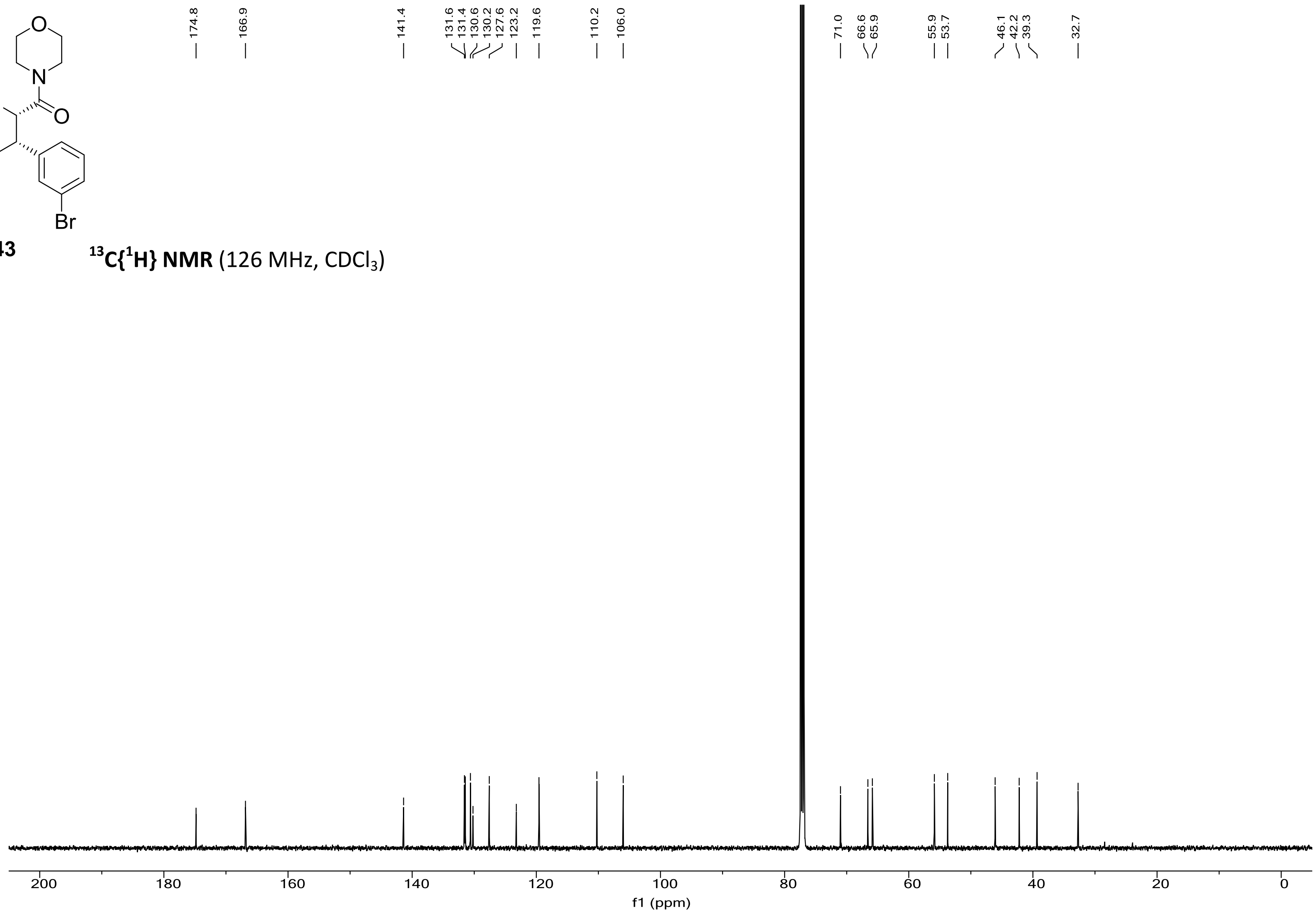

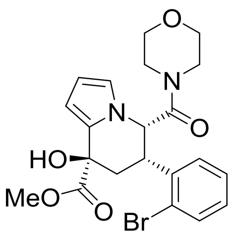

44

^{1}H NMR (500 MHz, $CDCl_3$)

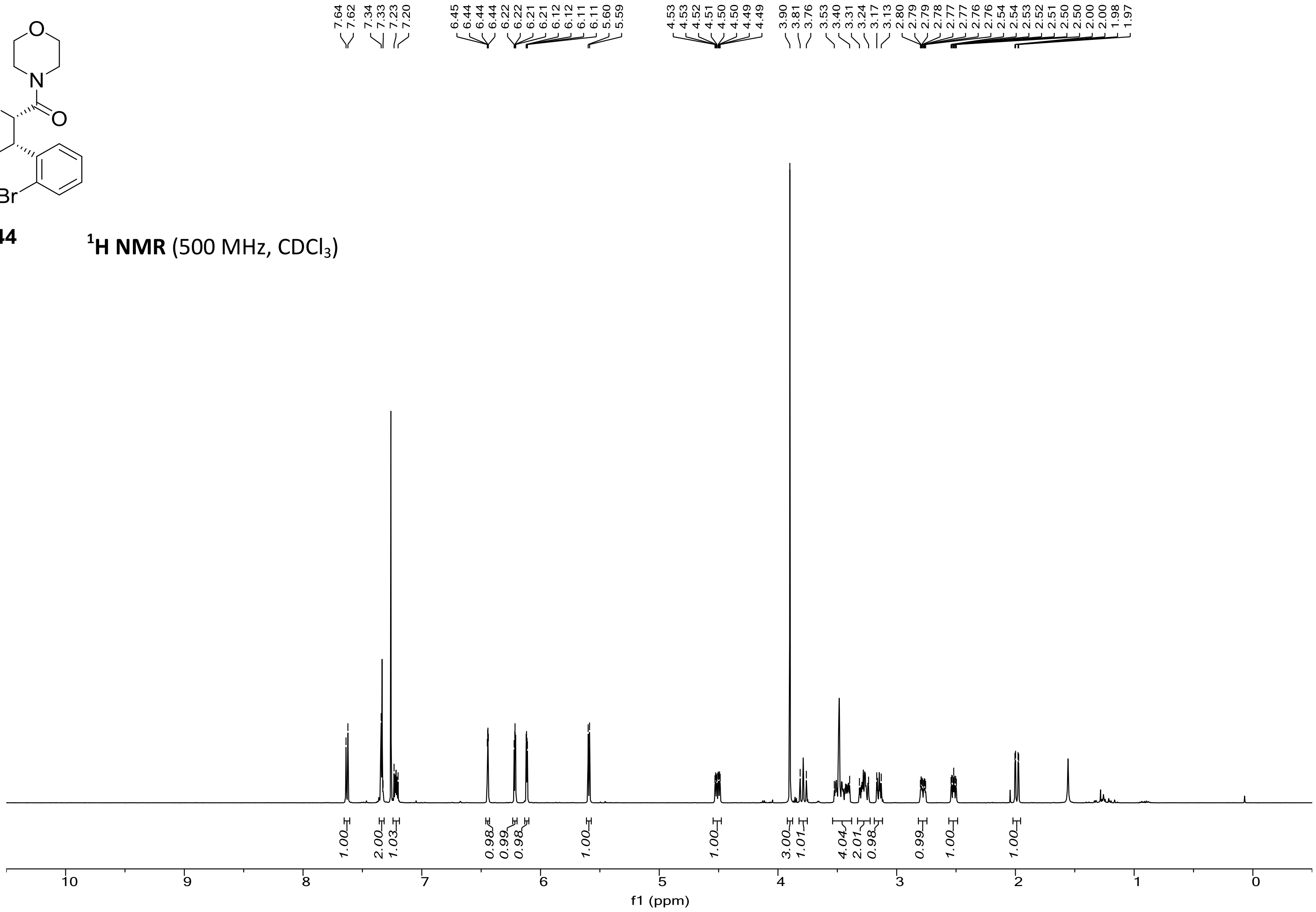

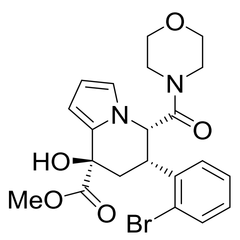

44

$^{13}C\{^{1}H\}$ NMR (126 MHz, $CDCl_3$)

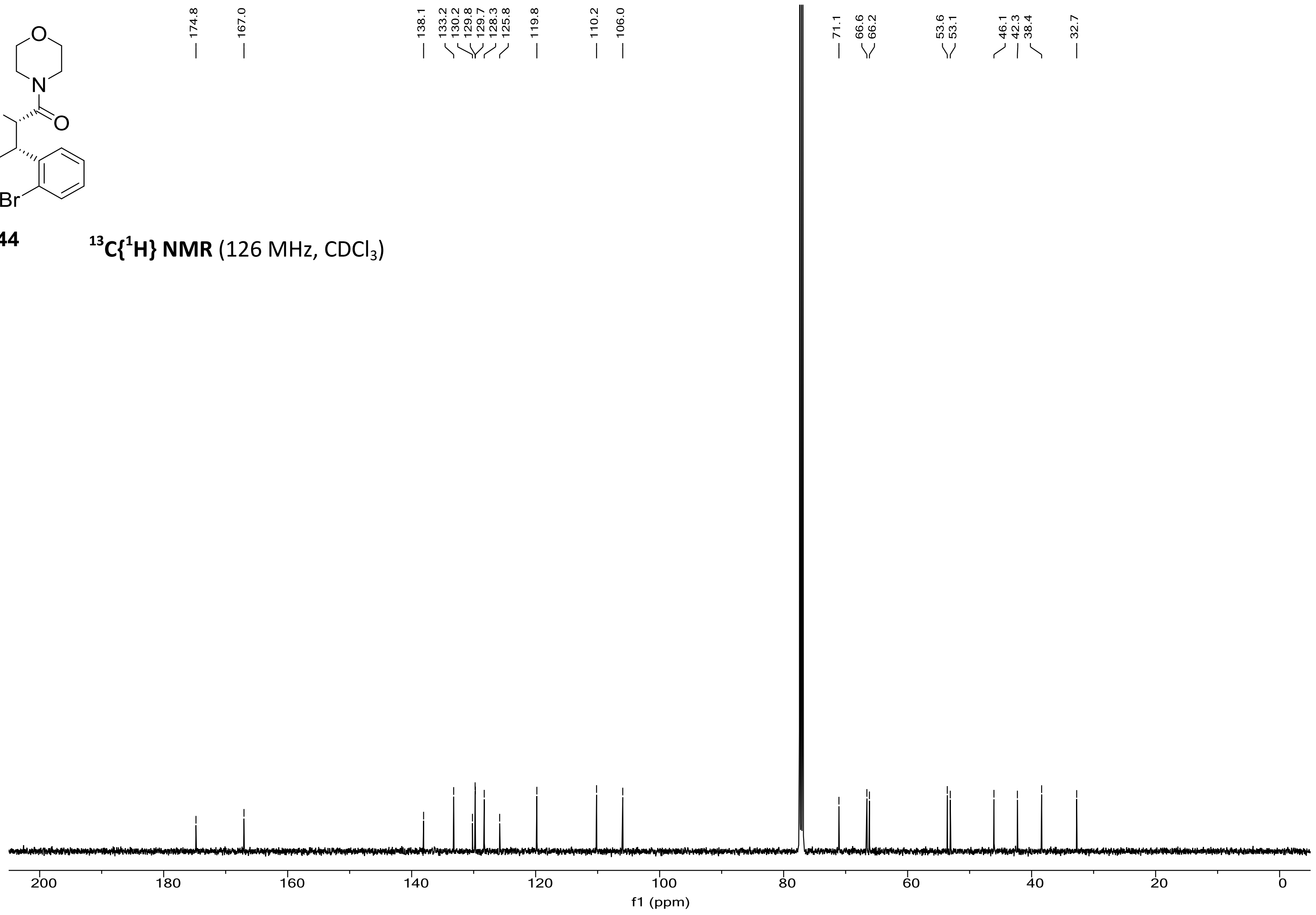

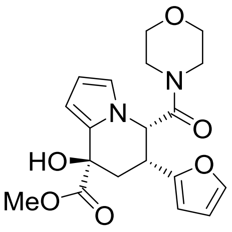

45

^{1}H NMR (500 MHz, $CDCl_3$)

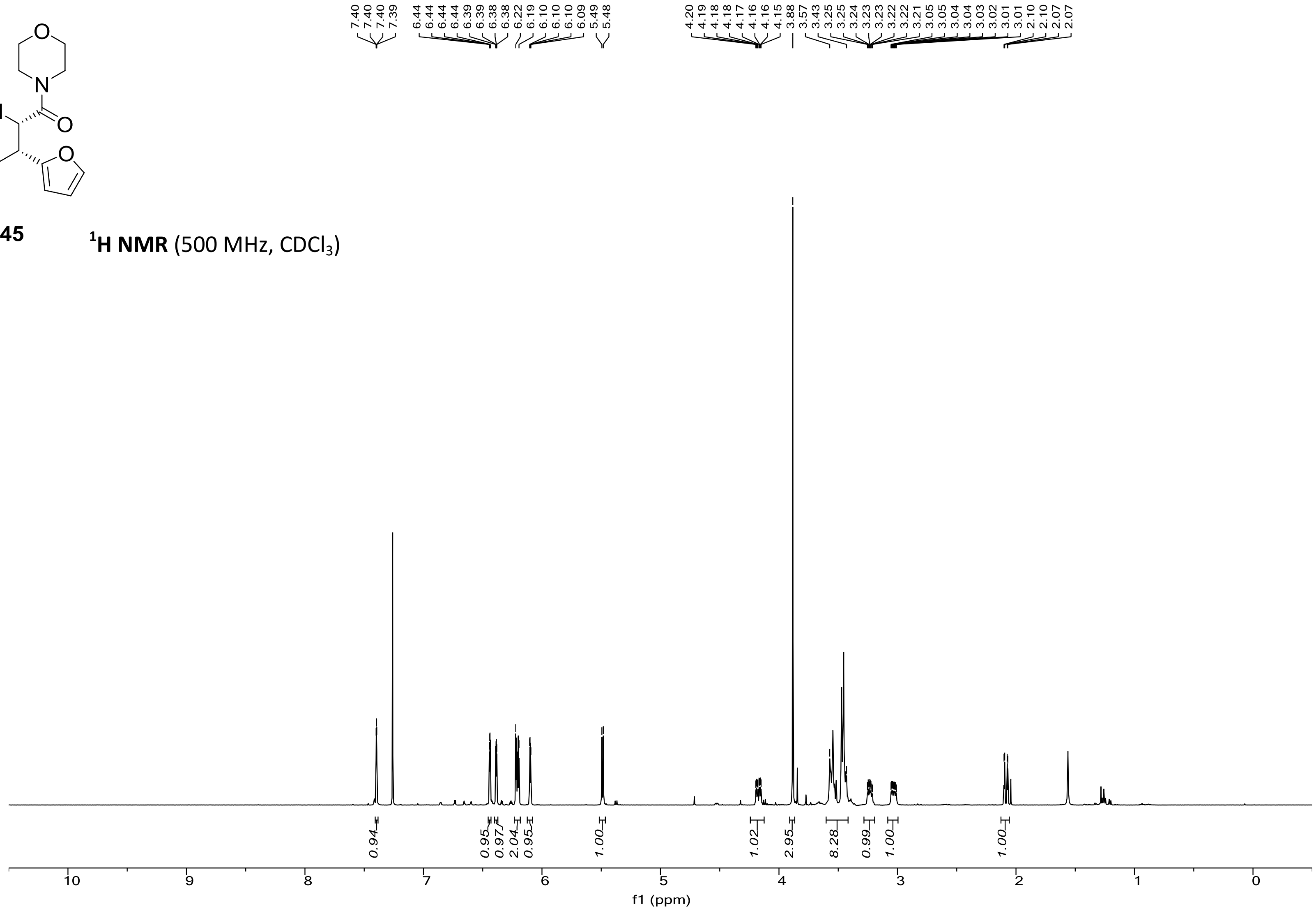

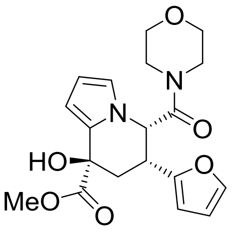

45

^{13}**C{^{1}H} NMR** (126 MHz, $CDCl_3$)

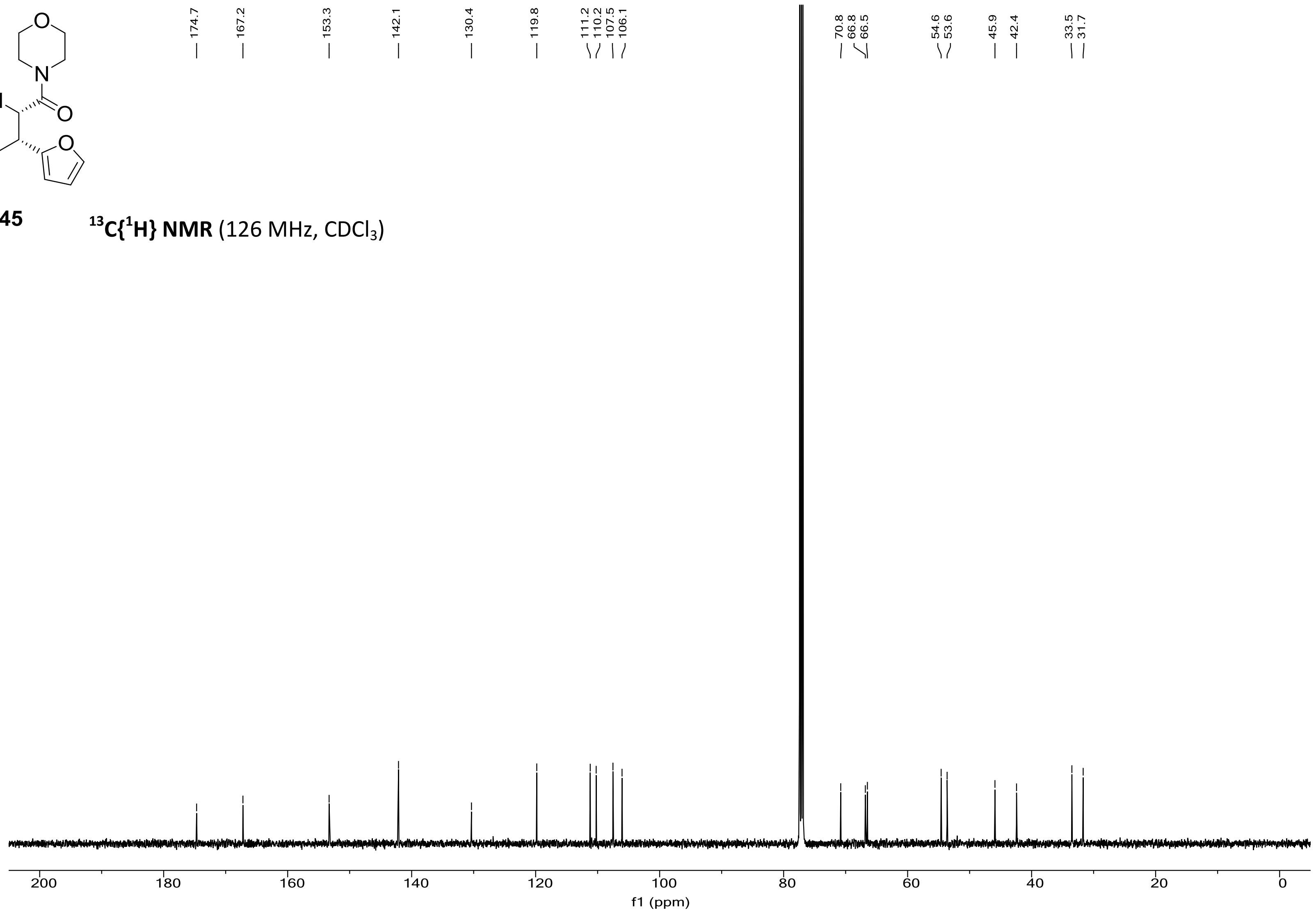

HPLC data for **8**: **Chiral HPLC analysis**, Chiralpak AD-H (97.5:2.5 hexane/*i*-PrOH, flow rate 1 mLmin^{-1}, 211 nm, 30 °C) t_R (major): 13.6 min, t_R (minor): 19.1 min, 95:5 er.
Minor diastereoisomer: **Chiral HPLC analysis**, Chiralpak AD-H (97.5:2.5 hexane/*i*-PrOH, flow rate 1 mLmin^{-1}, 211 nm, 30 °C) t_R (major): 15.3 min, t_R (minor): 22.3 min, 90:10 er.

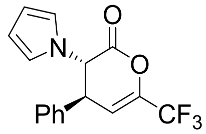

PDA Ch1 211nm

Peak#	Ret. Time	Area%
1	13.572	48.032
2	15.339	1.737
3	19.070	48.515
4	22.316	1.715
Total		100.000

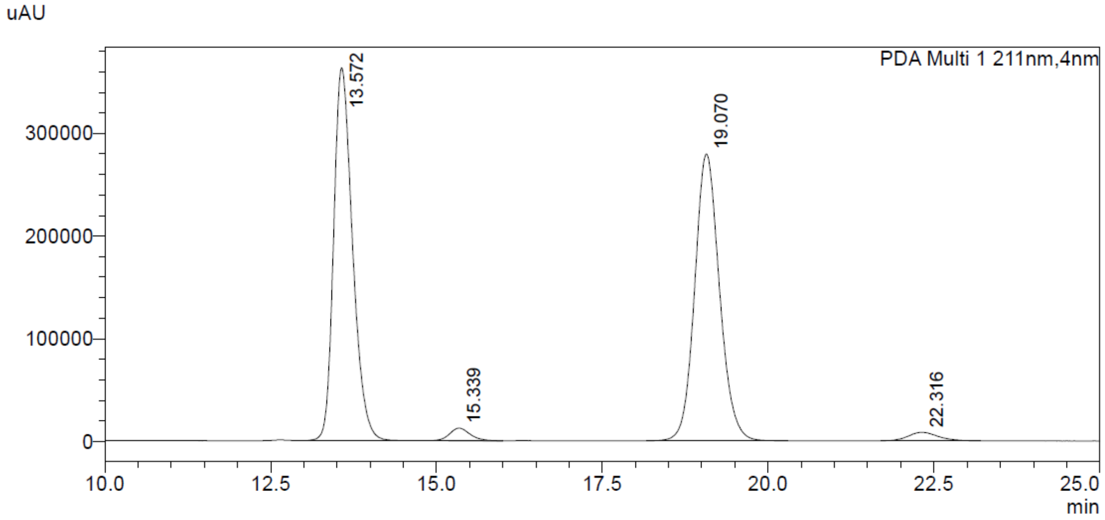

PDA Ch1 211nm

Peak#	Ret. Time	Area%
1	13.629	91.993
2	15.275	2.500
3	19.086	5.228
4	22.325	0.278
Total		100.000

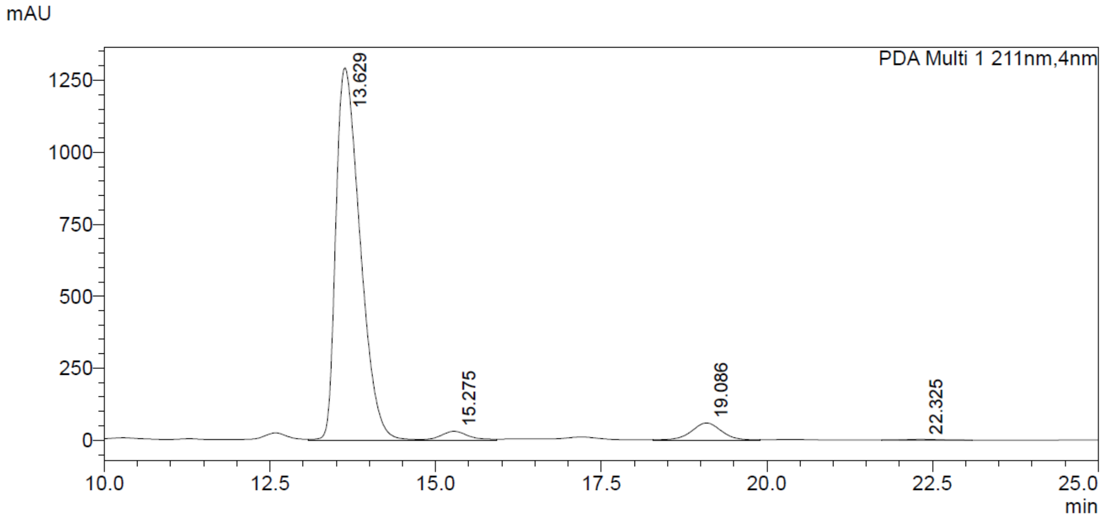

HPLC data for **9**: **Chiral HPLC analysis**, Chiralpak IB (99:1 hexane/*i*-PrOH, flow rate 1 mLmin^{-1}, 211 nm, 30 °C) t_R (major): 9.6 min, t_R (minor): 8.8 min, 96:4 er.

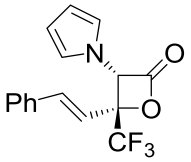

PDA Ch1 211nm

Peak#	Ret. Time	Area%
1	8.825	51.218
2	9.559	48.782
Total		100.000

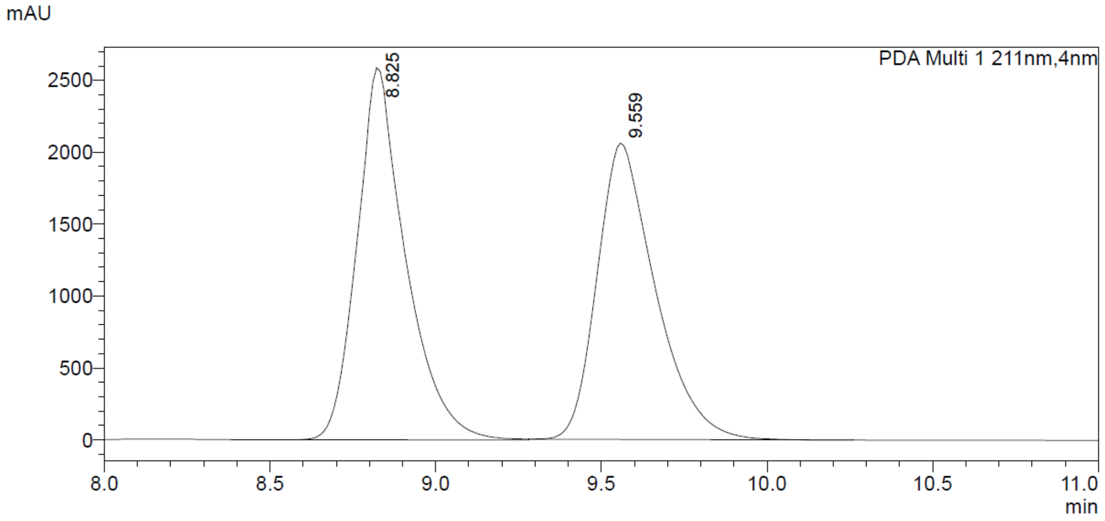

PDA Ch1 211nm

Peak#	Ret. Time	Area%
1	8.831	4.252
2	9.567	95.748
Total		100.000

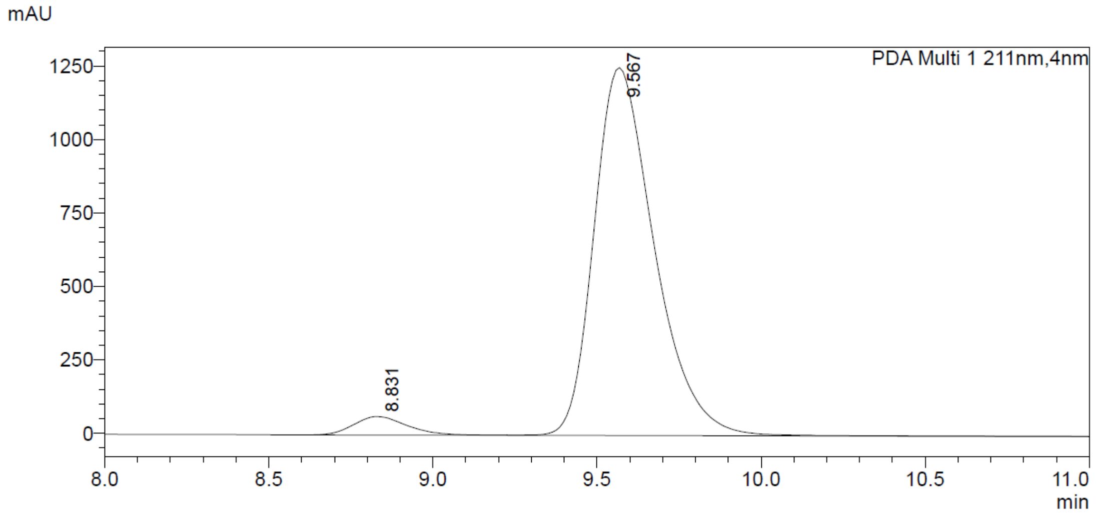

HPLC data for **10**: **Chiral HPLC analysis**, Chiralcel OD-H (90:10 hexane/*i*-PrOH, flow rate 1 mLmin^{-1}, 254 nm, 30 °C) t_R (major): 14.4 min, t_R (minor): 10.1 min, 80:20 er.

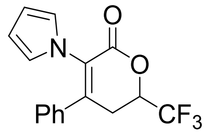

PDA Ch3 254nm

Peak#	Ret. Time	Area%
1	10.239	50.069
2	14.523	49.931
Total		100.000

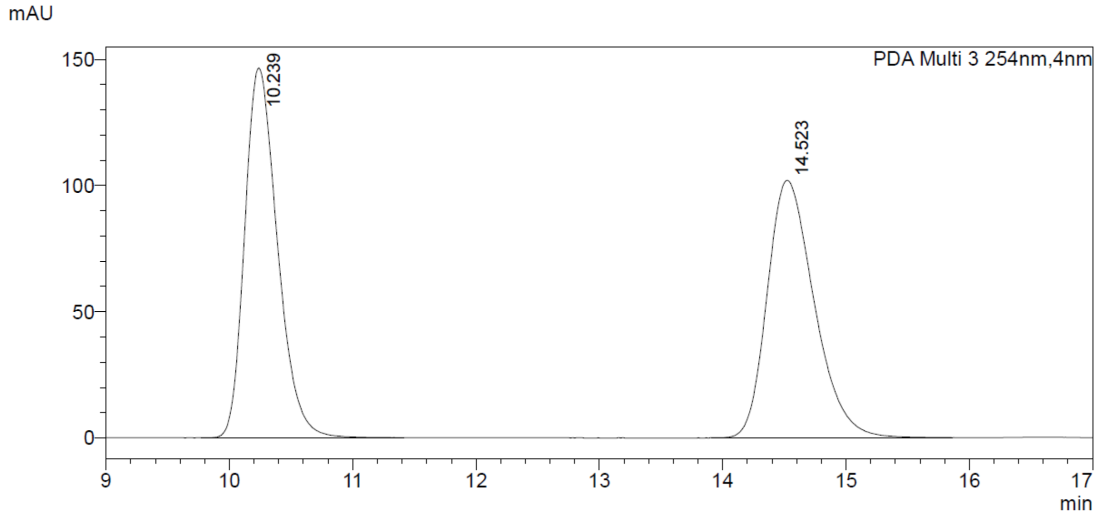

PDA Ch3 254nm

Peak#	Ret. Time	Area%
1	10.118	79.585
2	14.441	20.415
Total		100.000

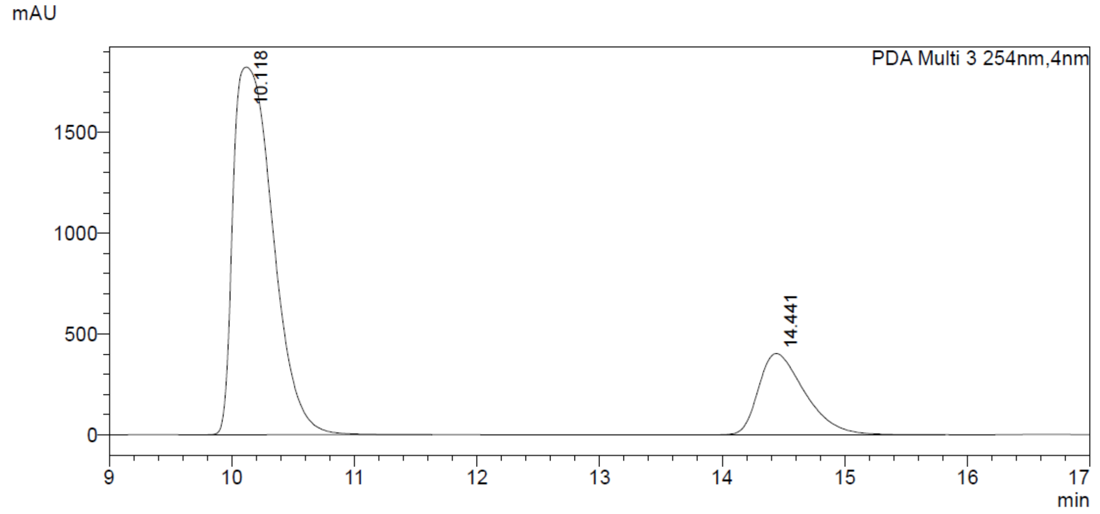

HPLC data for **14**: *major diastereoisomer*: **Chiral HPLC analysis**, Chiralpak AD-H (95:5 hexane/*i*-PrOH, flow rate 1 mLmin^{-1}, 220 nm, 30 °C) t_R (major): 19.5 min, t_R (minor): 28.4 min, 98:2 er. *minor diastereoisomer:* **Chiral HPLC analysis**, Chiralpak AD-H (95:5 hexane:*i*-PrOH, flow rate 1 mLmin^{-1}, 220 nm, 30 °C) t_R (major): 14.7 min, t_R (minor): 24.8 min, 96:4 er.

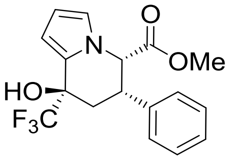

PDA Ch2 220nm

Peak#	Ret. Time	Area%
1	14.648	6.436
2	19.588	44.659
3	24.710	5.898
4	28.182	43.008
Total		100.000

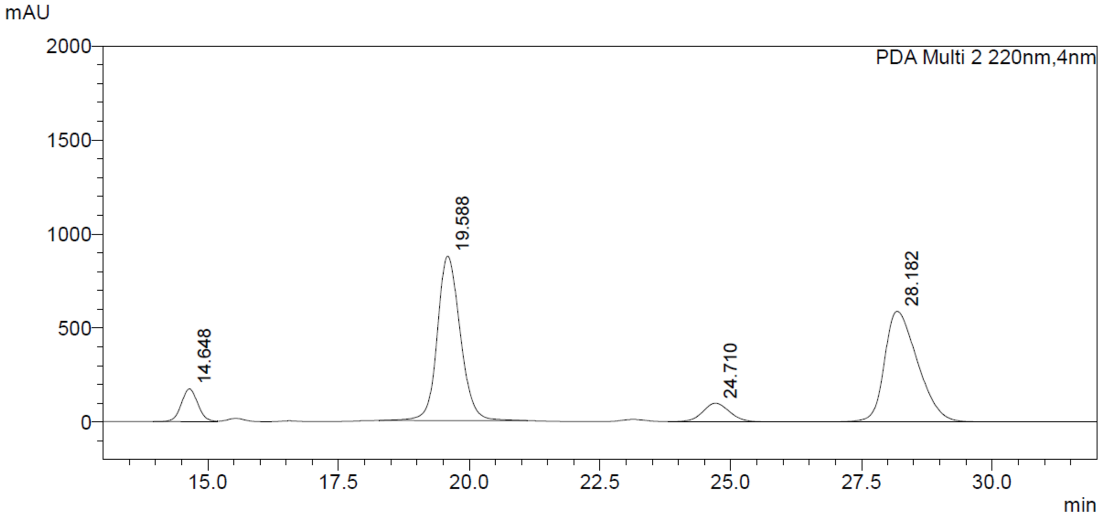

PDA Ch2 220nm

Peak#	Ret. Time	Area%
1	14.715	6.012
2	19.544	91.355
3	24.768	0.220
4	28.425	2.414
Total		100.000

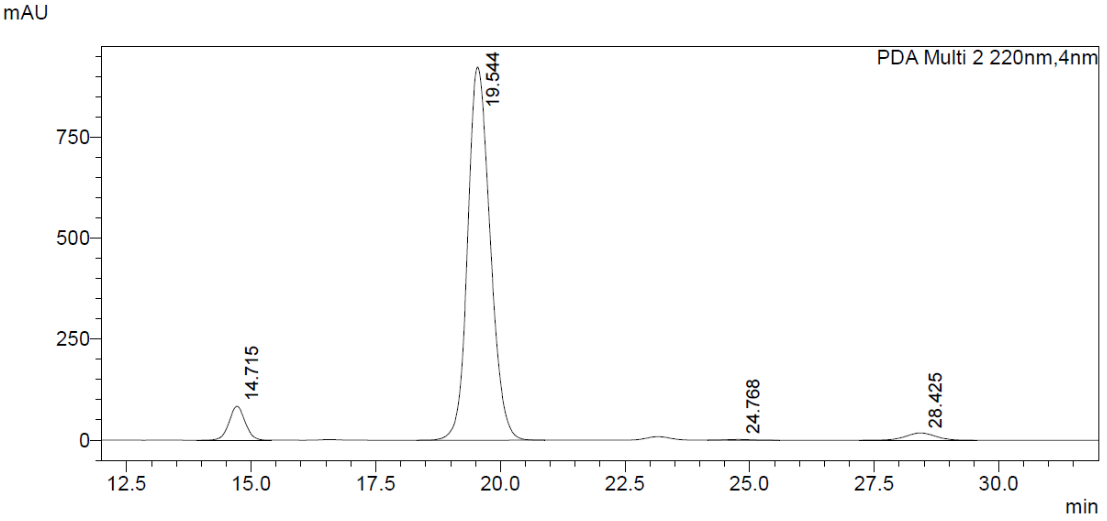

HPLC data for **16**: **Chiral HPLC analysis**, Chiralcel OD-H (80:20 hexane/*i*-PrOH, flow rate 1 mLmin^{-1}, 211 nm, 30 °C) t_R (major): 18.5 min, t_R (minor): 26.5 min, 97:3 er.

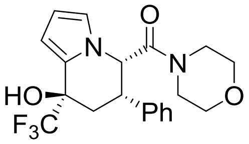

PDA Ch1 211nm

Peak#	Ret. Time	Area%
1	18.994	50.174
2	26.454	49.826
Total		100.000

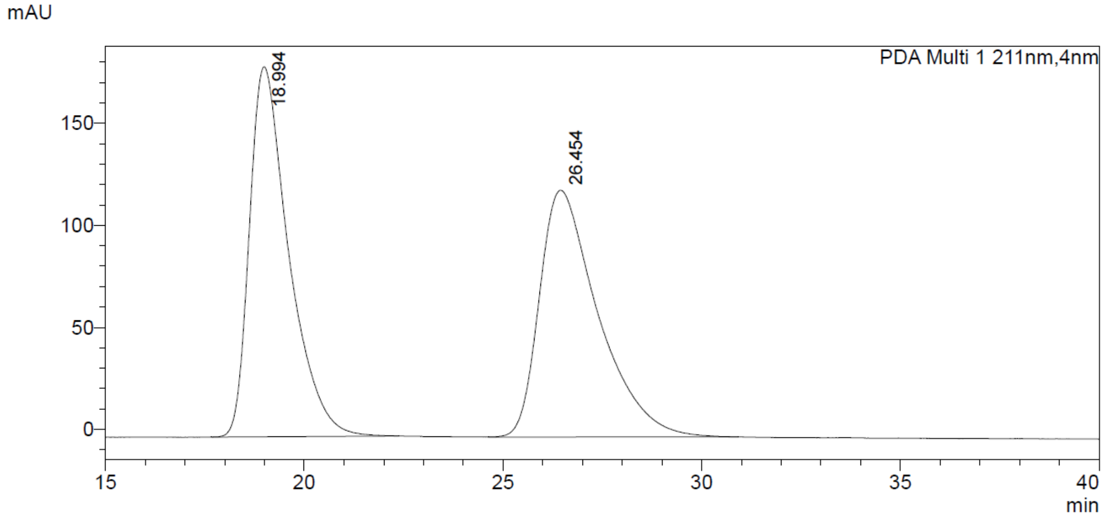

PDA Ch1 211nm

Peak#	Ret. Time	Area%
1	18.468	97.339
2	26.486	2.661
Total		100.000

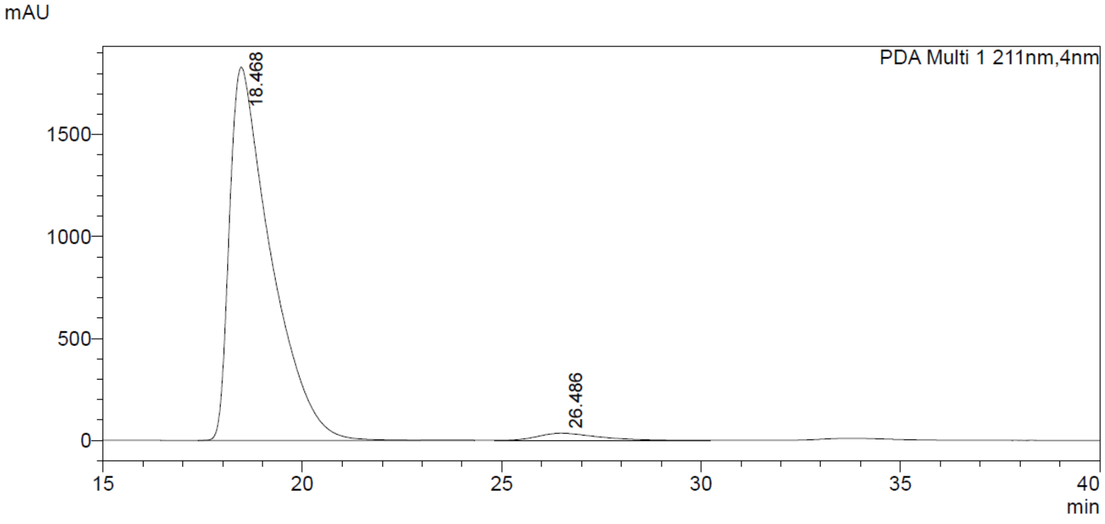

HPLC data for **17**: **Chiral HPLC analysis**, Chiralcel OD-H (80:20 hexane/*i*-PrOH, flow rate 1 mLmin^{-1}, 211 nm, 30 °C) t_R (major): 7.7 min, t_R (minor): 14.5 min, 98:2 er.

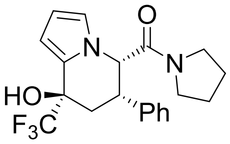

PDA Ch1 211nm

Peak#	Ret. Time	Area%
1	7.740	50.282
2	14.369	49.718
Total		100.000

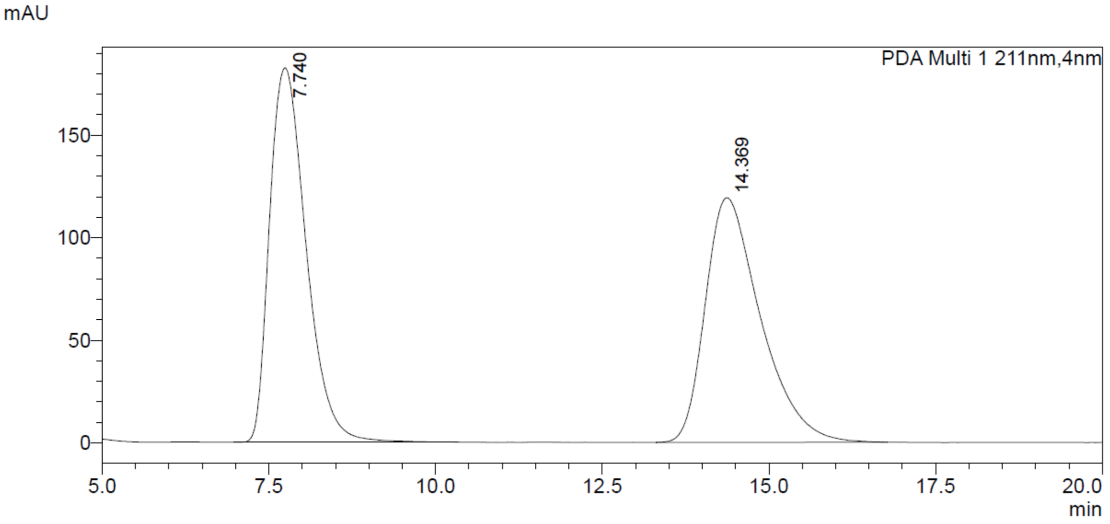

PDA Ch1 211nm

Peak#	Ret. Time	Area%
1	7.697	97.930
2	14.451	2.070
Total		100.000

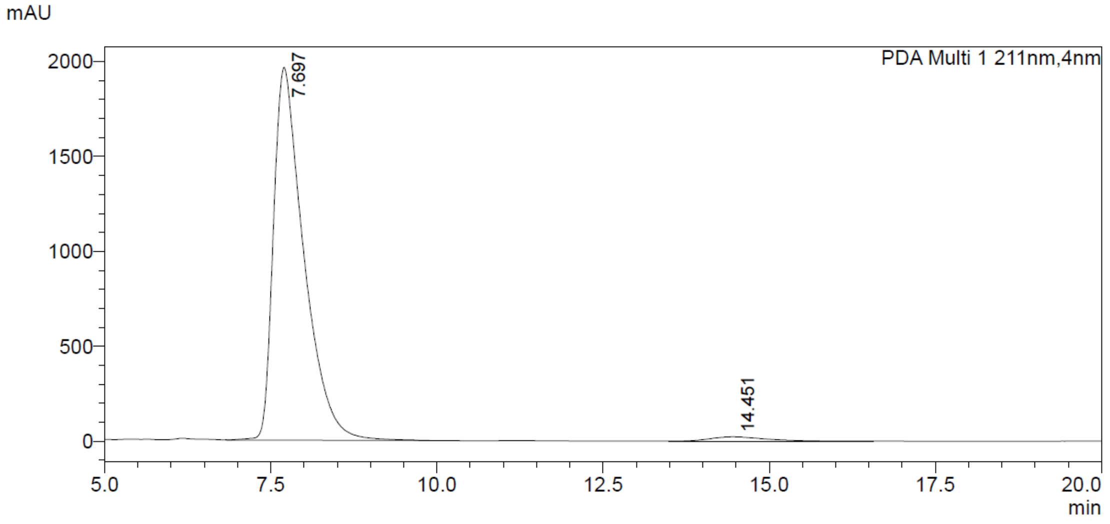

HPLC data for **18**: **Chiral HPLC analysis**, Chiralpak AD-H (97.5: 2.5 hexane/*i*-PrOH, flow rate 1 mLmin^{-1}, 220 nm, 30 °C) t_R (major): 52.0 min, t_R (minor): 73.7 min, 98:2 er.

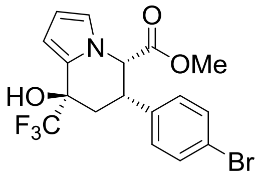

PDA Ch2 220nm

Peak#	Ret. Time	Area%
1	51.221	50.131
2	71.467	49.869
Total		100.000

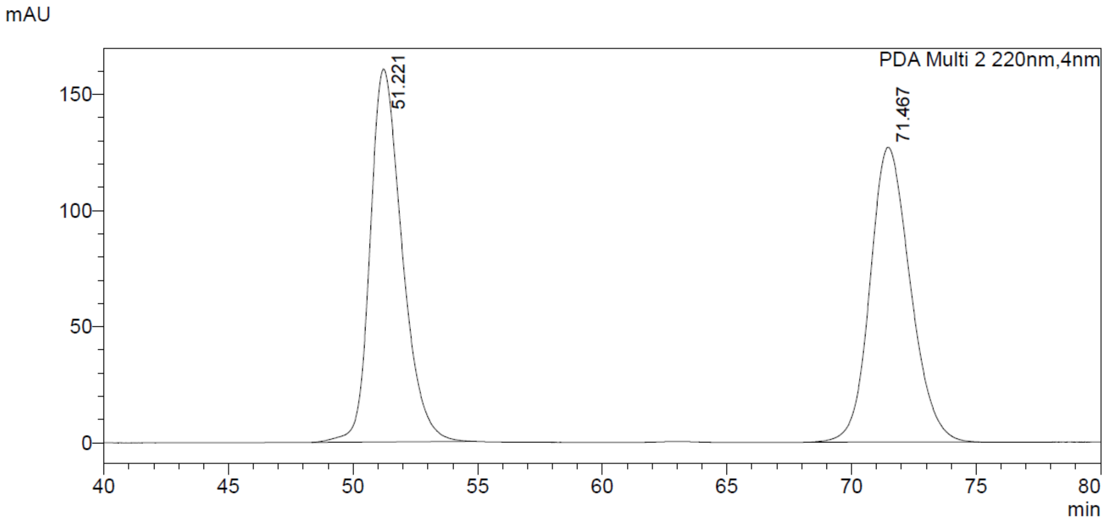

PDA Ch2 220nm

Peak#	Ret. Time	Area%
1	51.992	97.529
2	73.688	2.471
Total		100.000

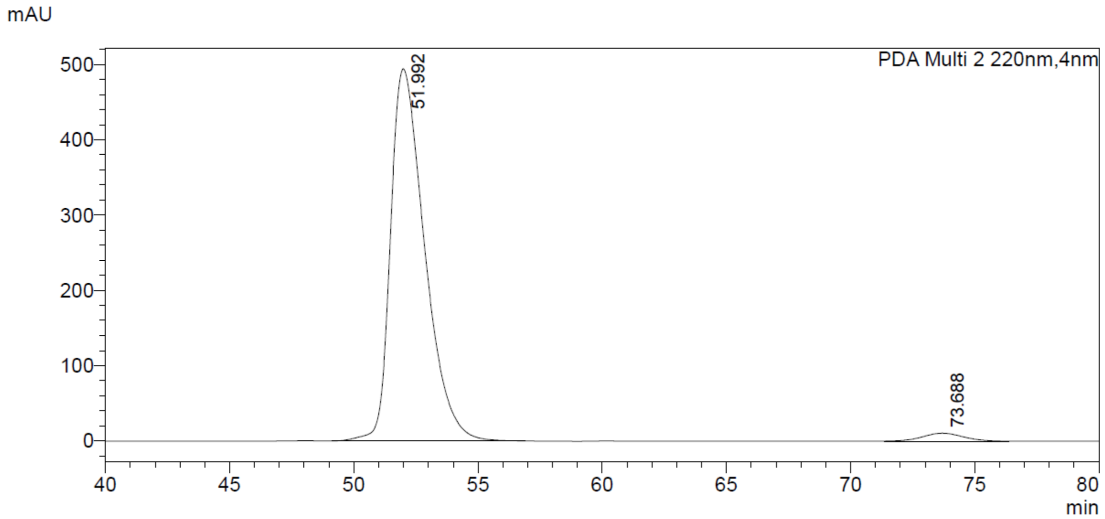

HPLC data for **19**: **Chiral HPLC analysis**, Chiralpak AD-H (97.5:2.5 hexane/IPA, flow rate 1 $mLmin^{-1}$, 211 nm, 30 °C) t_R (major): 45.6 min, t_R (minor): 62.9 min, 97:3 er.

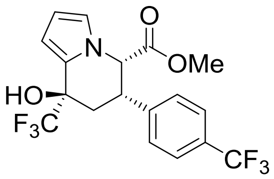

PDA Ch1 211nm

Peak#	Ret. Time	Area%
1	45.509	49.940
2	61.916	50.060
Total		100.000

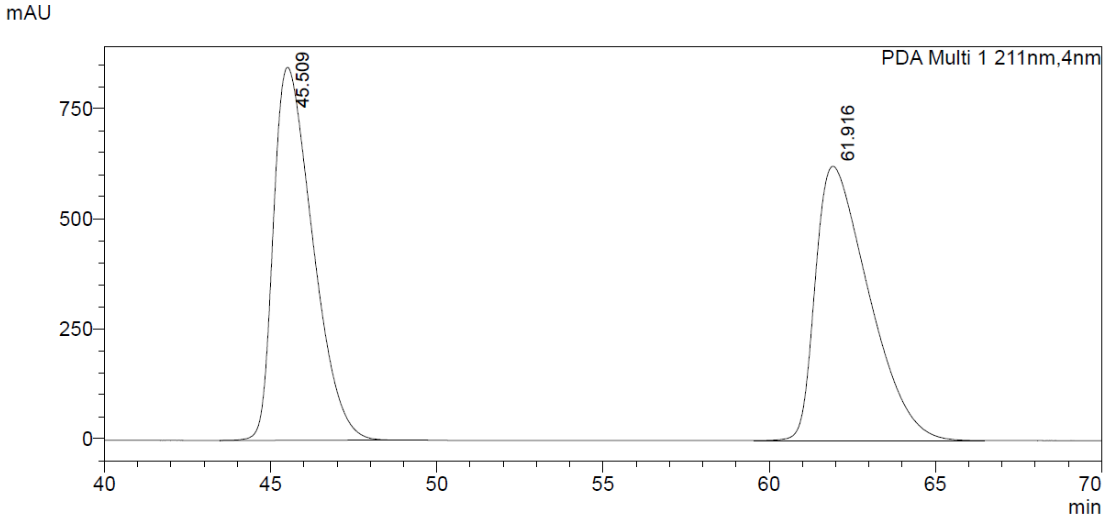

PDA Ch1 211nm

Peak#	Ret. Time	Area%
1	45.594	96.301
2	62.949	3.699
Total		100.000

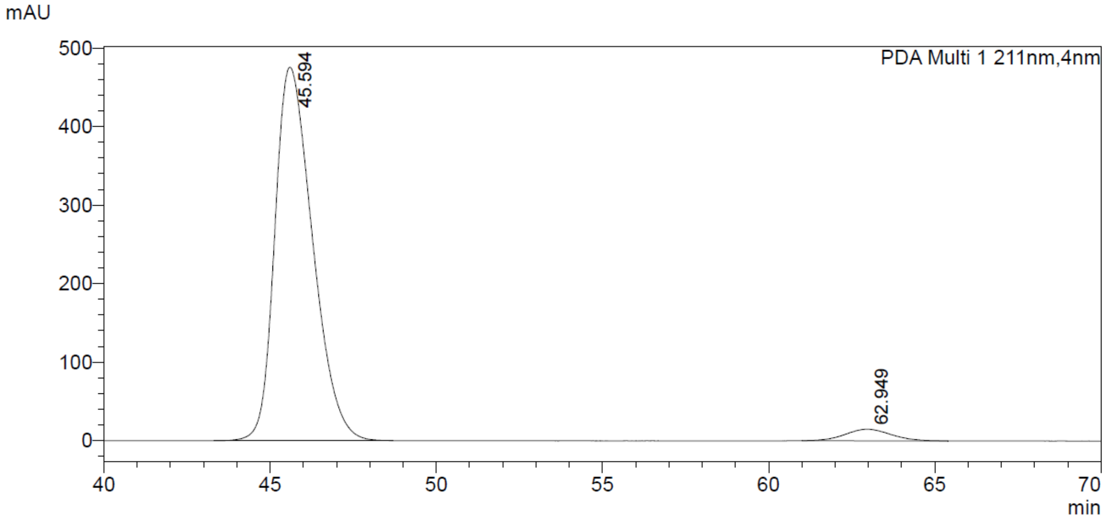

HPLC data for **20**: **Chiral HPLC analysis**, Chiralpak IB (85:15 hexane/IPA, flow rate 1 mLmin^{-1}, 211 nm, 30 °C) t_R (major): 9.0 min, t_R (minor): 23.6 min, 96:4 er.

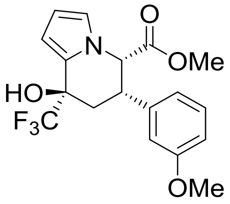

PDA Ch1 211nm

Peak#	Ret. Time	Area%
1	8.942	50.735
2	23.111	49.265
Total		100.000

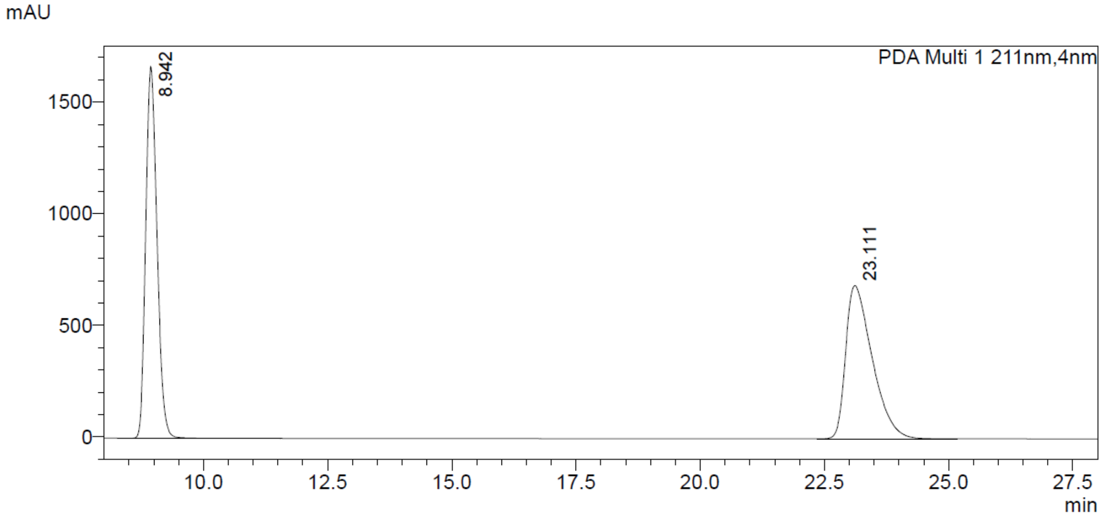

PDA Ch1 211nm

Peak#	Ret. Time	Area%
1	9.007	96.328
2	23.631	3.672
Total		100.000

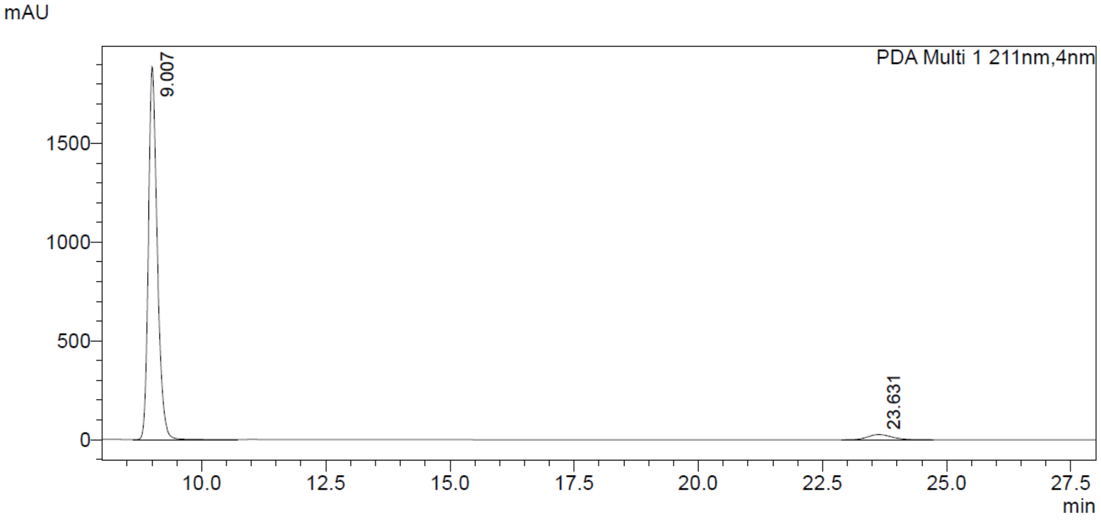

HPLC data for **21**: **Chiral HPLC analysis**, Chiralpak AD-H (97.5:2.5 hexane/IPA, flow rate 1 $mLmin^{-1}$, 211 nm, 30 °C) t_R (major): 59.1 min, t_R (minor): 104.6 min, 99:1 er.

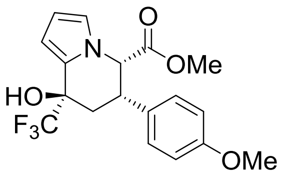

PDA Ch1 211nm

Peak#	Ret. Time	Area%
1	59.305	50.405
2	104.522	49.595
Total		100.000

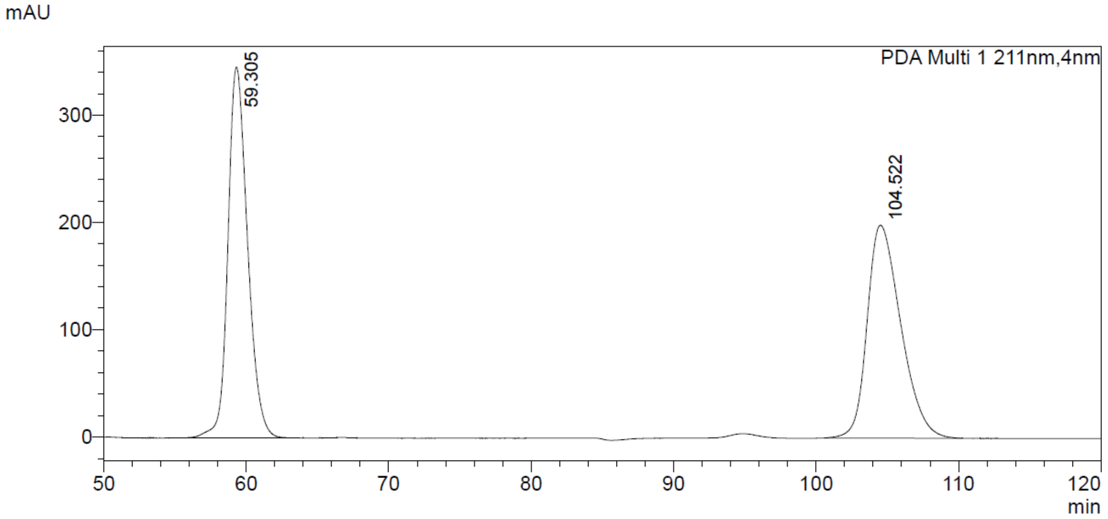

PDA Ch1 211nm

Peak#	Ret. Time	Area%
1	59.086	98.824
2	104.556	1.176
Total		100.000

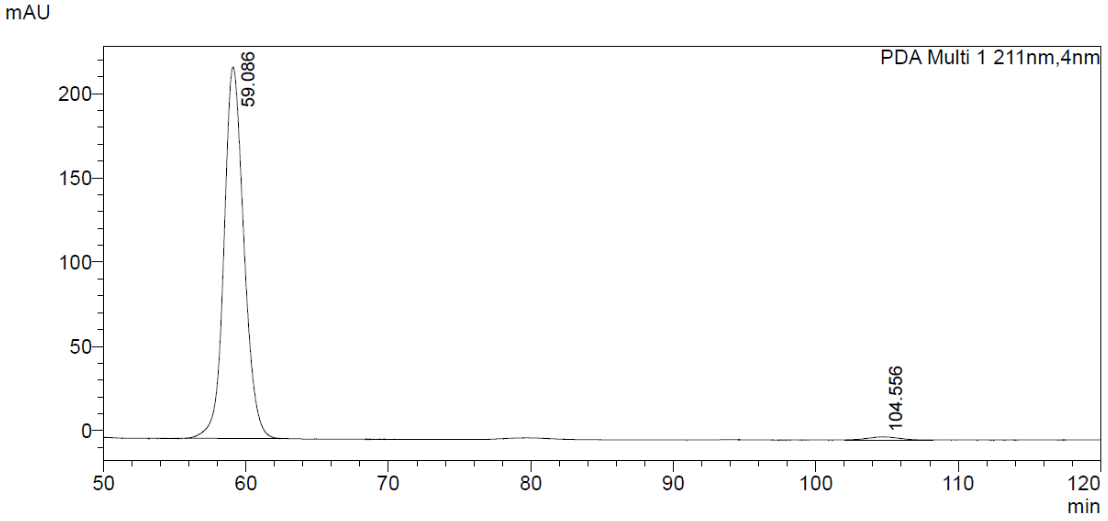

HPLC data for **22**: **Chiral HPLC analysis**, Chiralpak AD-H (97.5: 2.5 hexane/*i*-PrOH, flow rate 1 mLmin^{-1}, 220 nm, 30 °C) t_R (major): 38.7 min, t_R (minor): 59.7 min, 98:2 er.

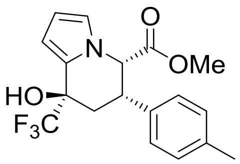

PDA Ch2 220nm

Peak#	Ret. Time	Area%
1	38.172	48.543
2	58.426	51.457
Total		100.000

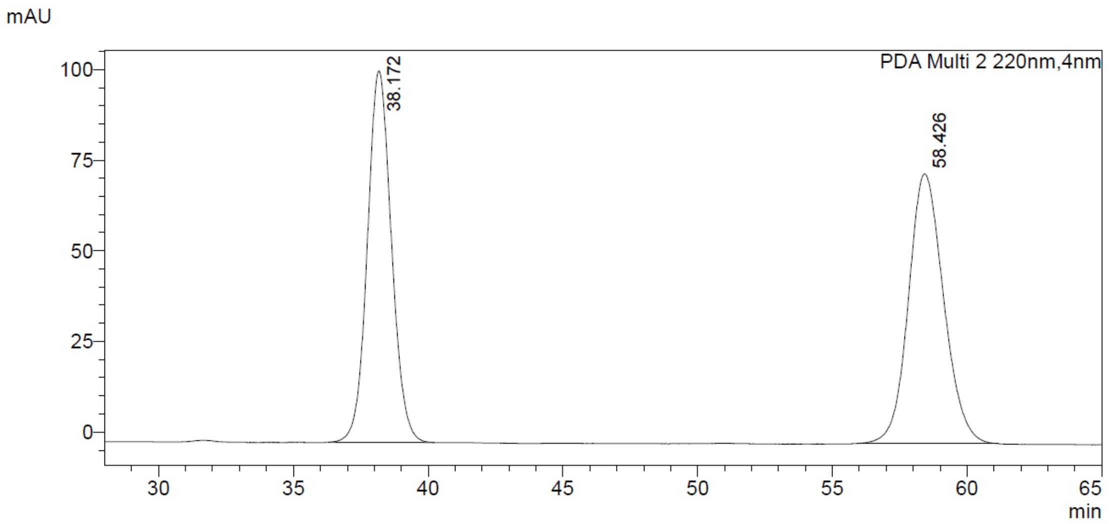

PDA Ch2 220nm

Peak#	Ret. Time	Area%
1	38.671	98.006
2	59.689	1.994
Total		100.000

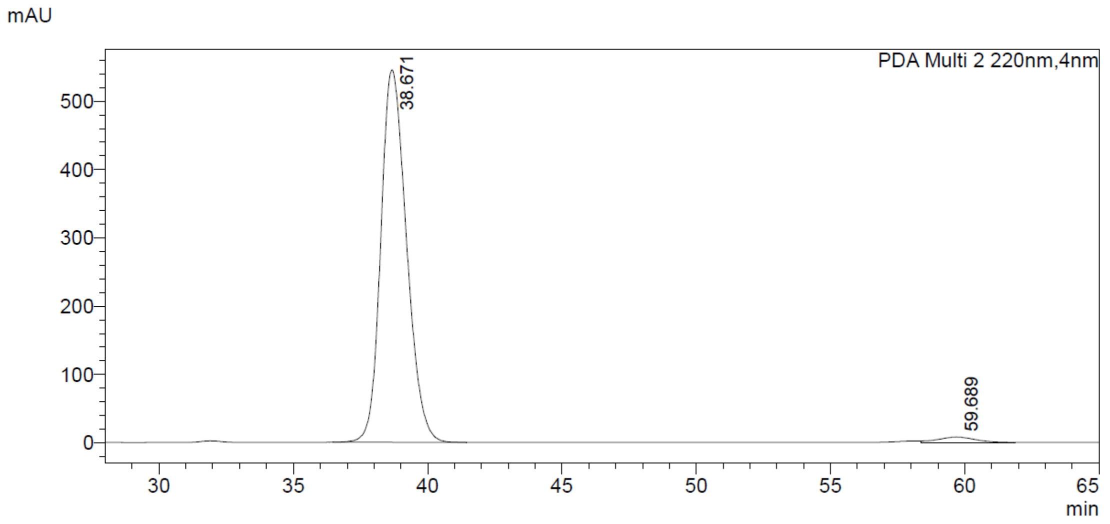

HPLC data for **23**: **Chiral HPLC analysis**, Chiralpak AD-H (97.5:2.5 hexane : IPA, flow rate 1 mLmin^{-1}, 211 nm, 30 °C) t_R (major): 17.6 min, t_R (minor): 77.3 min, > 99:1 er.

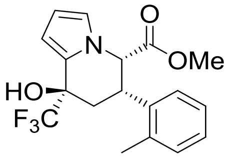

PDA Ch1 211nm

Peak#	Ret. Time	Area%
1	17.511	50.269
2	76.393	49.731
Total		100.000

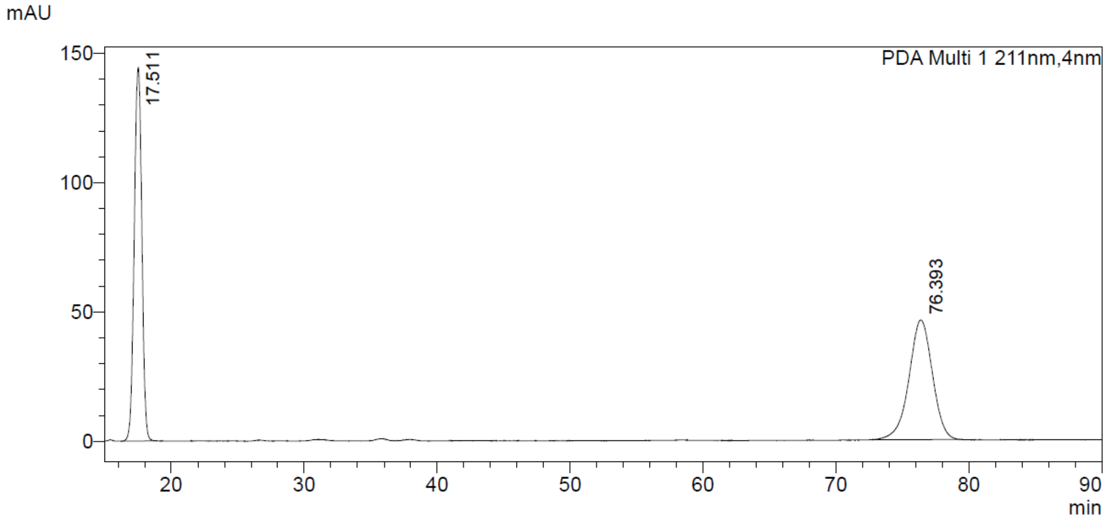

PDA Ch1 211nm

Peak#	Ret. Time	Area%
1	17.595	99.854
2	77.342	0.146
Total		100.000

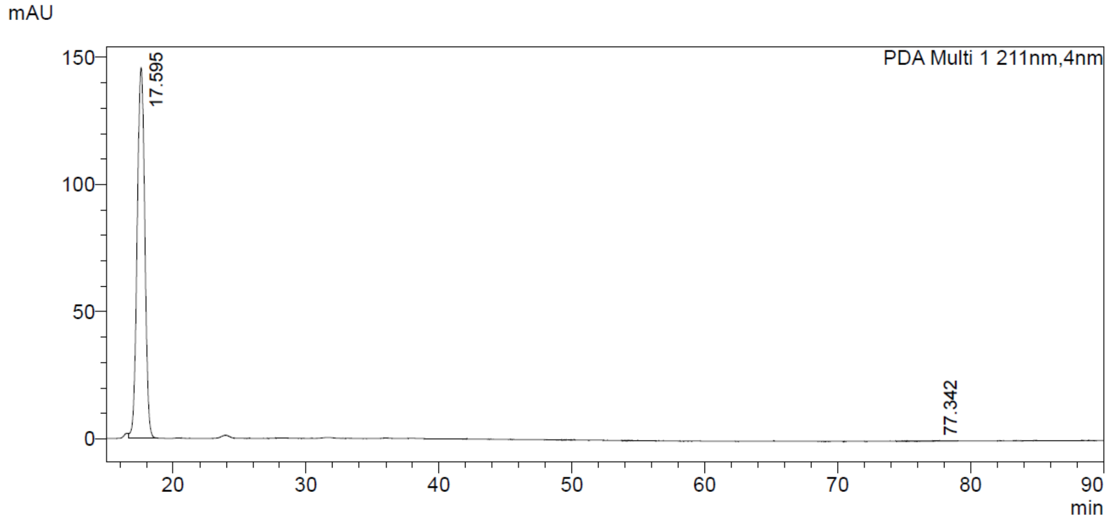

HPLC data for **24**: **Chiral HPLC analysis**, Chiralpak AD-H (97.5:2.5 hexane/IPA, flow rate 1 mLmin^{-1}, 211 nm, 30 °C) t_R (major): 52.9 min, t_R (minor): 45.2 min, 98:2 er.

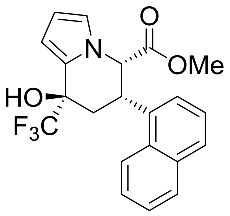

PDA Ch1 211nm

Peak#	Ret. Time	Area%
1	45.266	50.288
2	53.161	49.712
Total		100.000

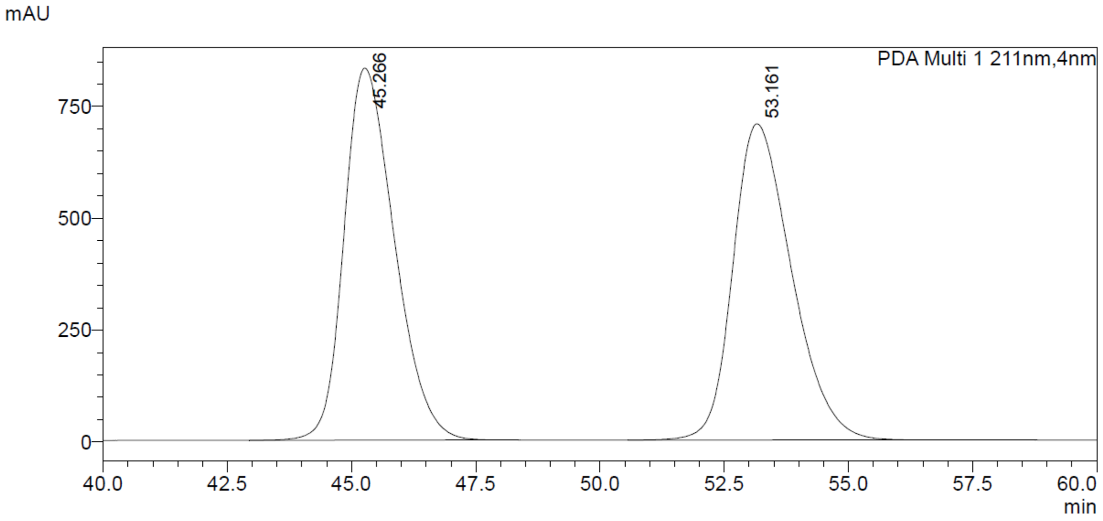

PDA Ch1 211nm

Peak#	Ret. Time	Area%
1	45.171	2.039
2	52.858	97.961
Total		100.000

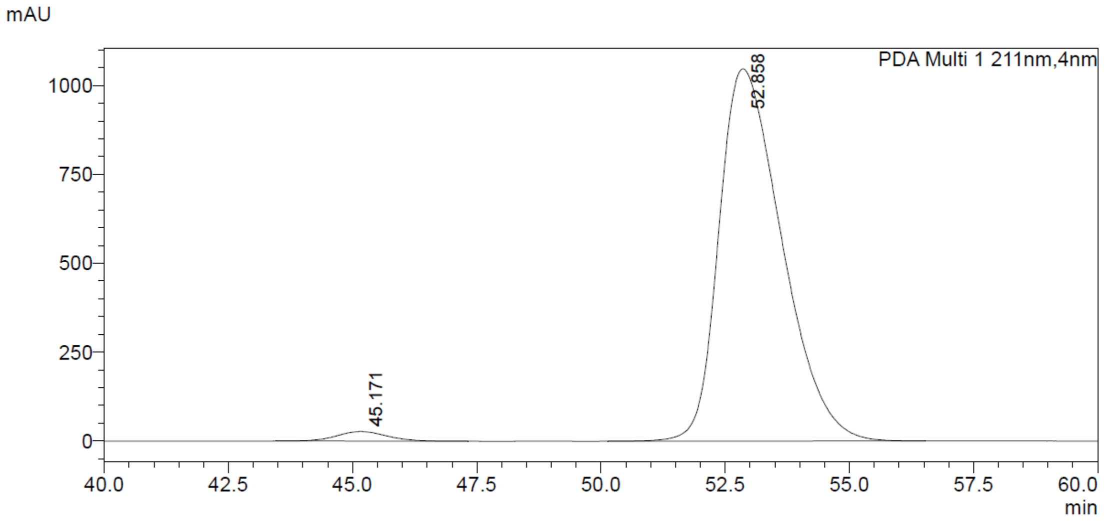

HPLC data for **25**: **Chiral HPLC analysis**, Chiralpak AD-H (97.5:2.5 hexane/IPA, flow rate 1 mLmin^{-1}, 254 nm, 30 °C) t_R (major): 69.1 min, t_R (minor): 91.9 min, 98:2 er.

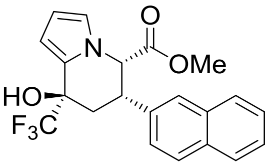

PDA Ch3 254nm

Peak#	Ret. Time	Area%
1	68.808	50.114
2	89.846	49.886
Total		100.000

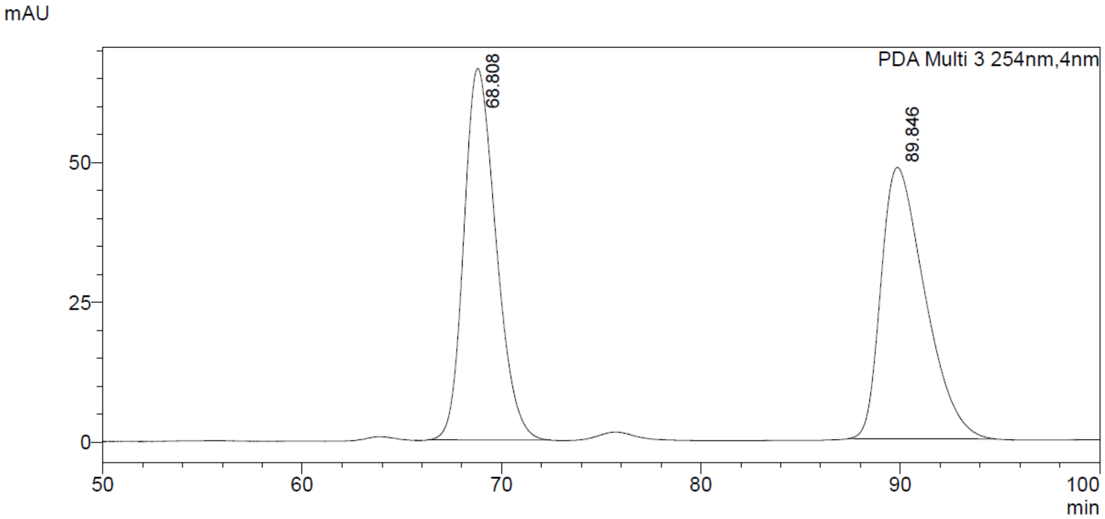

PDA Ch3 254nm

Peak#	Ret. Time	Area%
1	69.068	97.688
2	91.902	2.312
Total		100.000

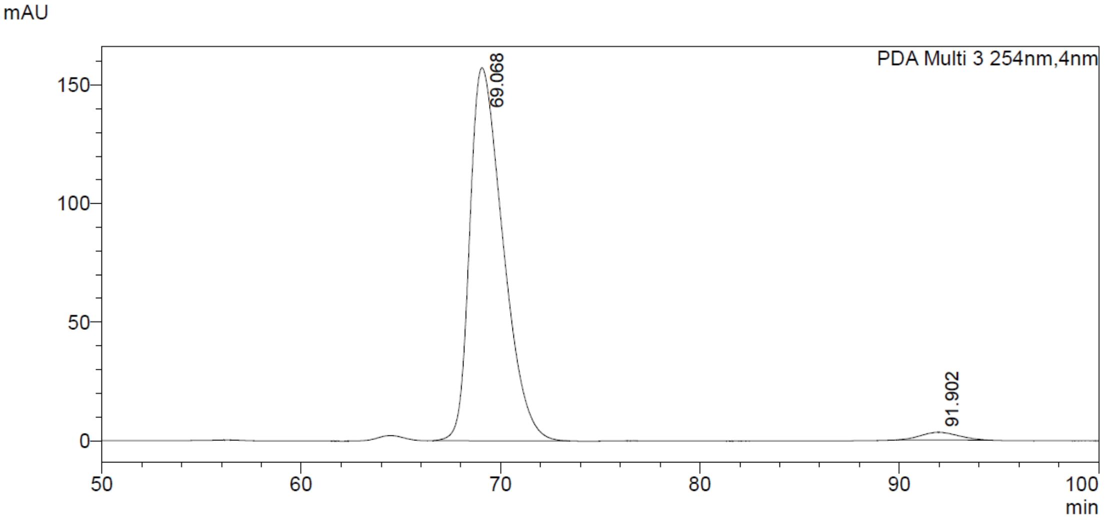

HPLC data for **26**: *major diastereoisomer*: **Chiral HPLC analysis**, Chiralpak AD-H (97.5:2.5 hexane/IPA, flow rate 1 mLmin^{-1}, 211 nm, 30 °C) t_R (major): 40.4 min, t_R (minor): 44.9 min, 97:3 er; *minor diastereoisomer*: **Chiral HPLC analysis**, Chiralpak AD-H (97.5:2.5 hexane/IPA, flow rate 1 mLmin^{-1}, 211 nm, 30 °C) t_R (major): 25.0 min, t_R (minor): 31.8 min, 92:8 er.

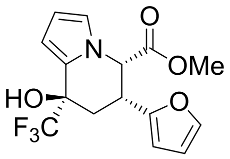

PDA Ch1 211nm

Peak#	Ret. Time	Area%
1	25.582	12.902
2	32.515	12.902
3	41.275	37.131
4	45.488	37.064
Total		100.000

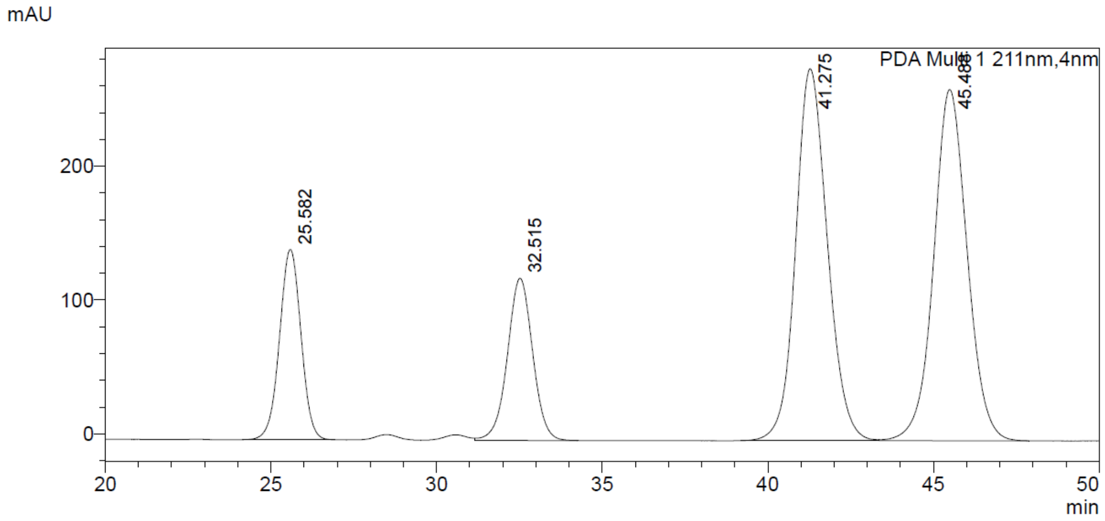

PDA Ch1 211nm

Peak#	Ret. Time	Area%
1	24.995	21.066
2	31.757	1.914
3	40.417	74.831
4	44.856	2.190
Total		100.000

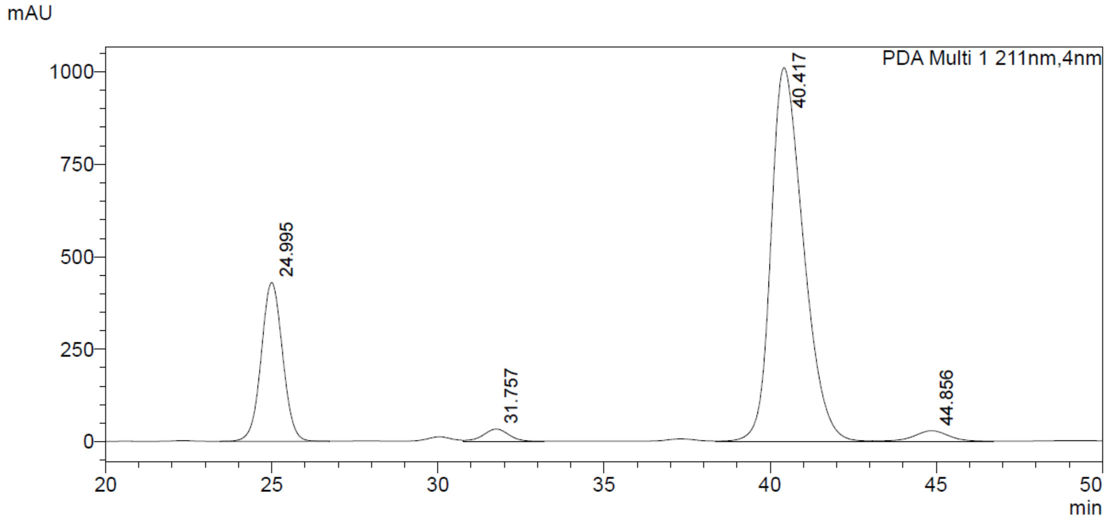

HPLC data for **31**: **Chiral HPLC analysis**, Chiralcel OD-H (80:20 hexane/*i*-PrOH, flow rate 1 mLmin^{-1}, 211 nm, 30 °C) t_R (major): 25.2 min, t_R (minor): 33.3 min, > 99:1 er.

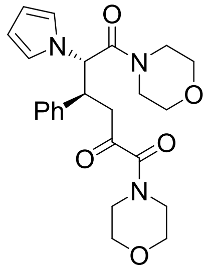

PDA Ch1 211nm

Peak#	Ret. Time	Area%
1	26.469	50.046
2	32.541	49.954
Total		100.000

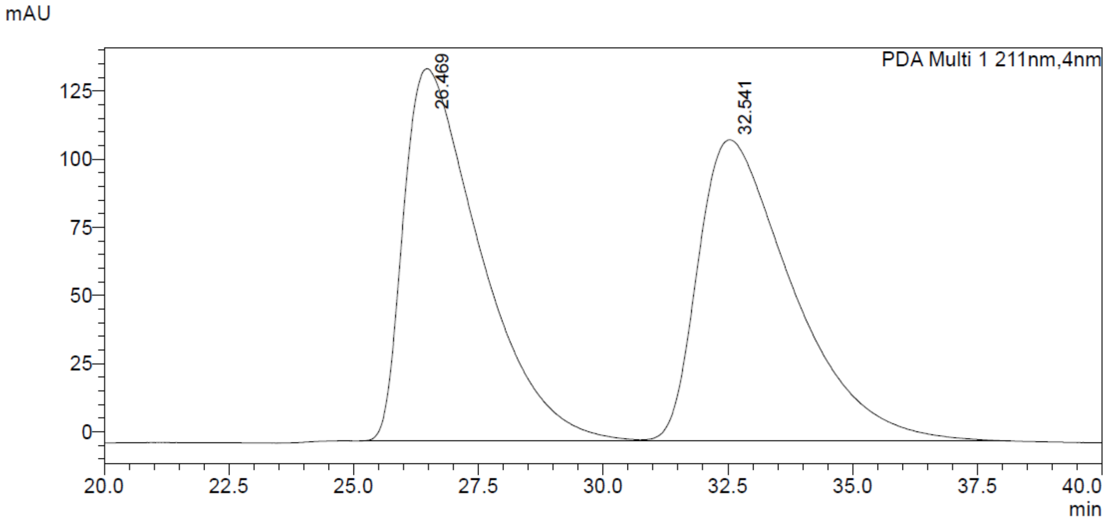

PDA Ch1 211nm

Peak#	Ret. Time	Area%
1	25.190	99.926
2	33.355	0.074
Total		100.000

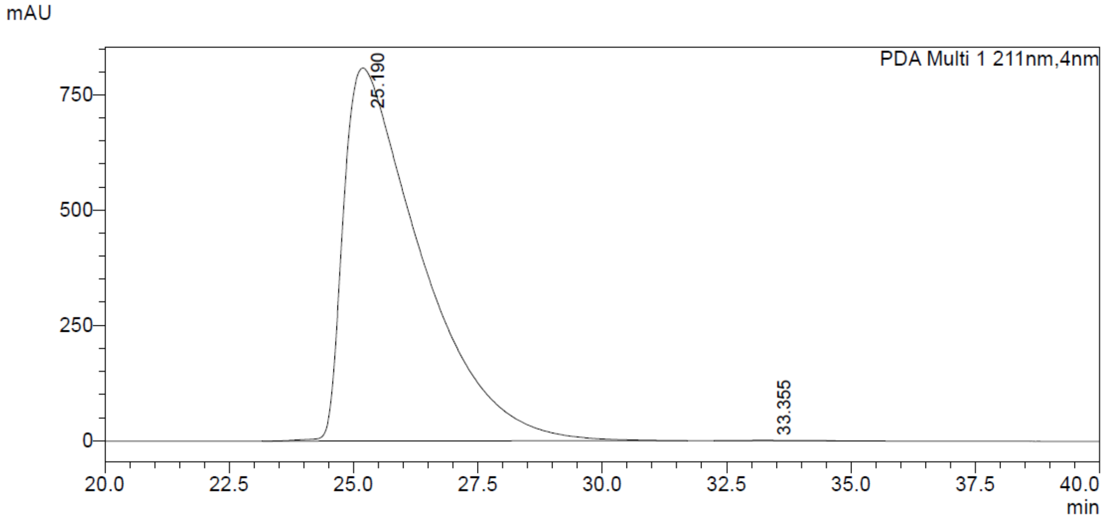

HPLC data for **32**: **Chiral HPLC analysis**, Chiralcel OD-H (90:10 hexane/*i*-PrOH, flow rate 1 mLmin^{-1}, 211 nm, 30 °C) t_R (major): 21.3 min, t_R (minor): 27.8 min, > 99:1 er.

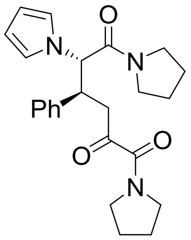

PDA Ch1 211nm

Peak#	Ret. Time	Area%
1	22.077	50.079
2	28.226	49.921
Total		100.000

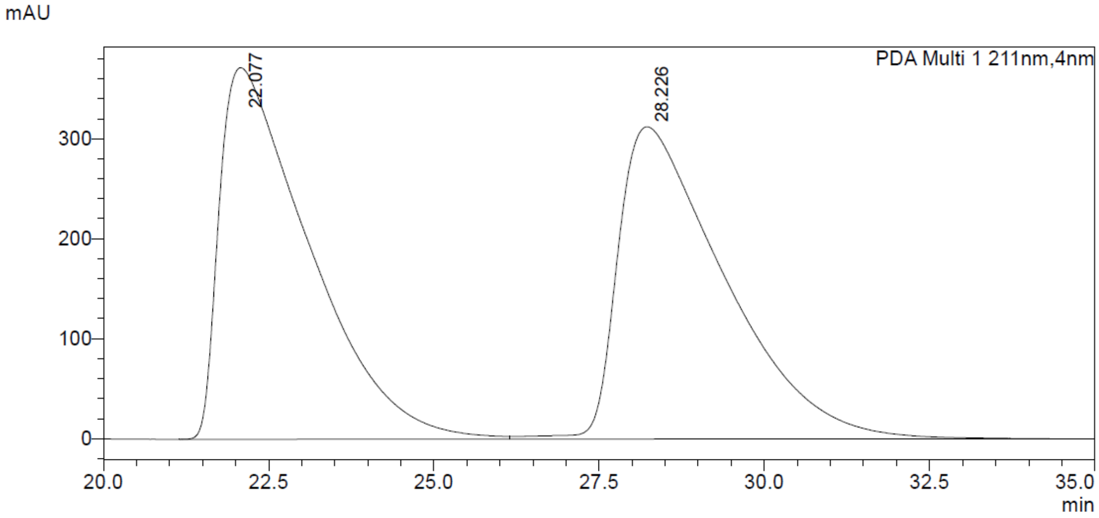

PDA Ch1 211nm

Peak#	Ret. Time	Area%
1	21.305	99.927
2	27.781	0.073
Total		100.000

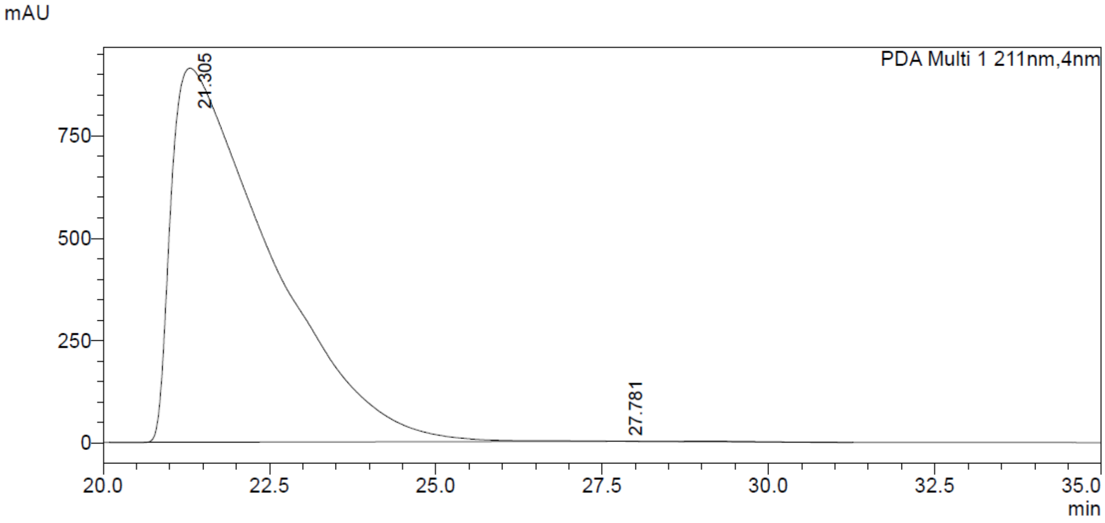

HPLC data for **33**: **Chiral HPLC analysis**, Chiralpak AD-H (80:20 hexane/IPA, flow rate 1 mLmin^{-1}, 211 nm, 30 °C) t_R (major): 18.8 min, t_R (minor): 31.9 min, > 99:1 er

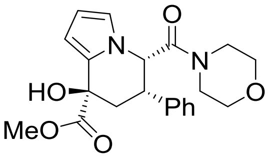

PDA Ch1 211nm

Peak#	Ret. Time	Area%
1	17.336	49.239
2	31.079	50.761
Total		100.000

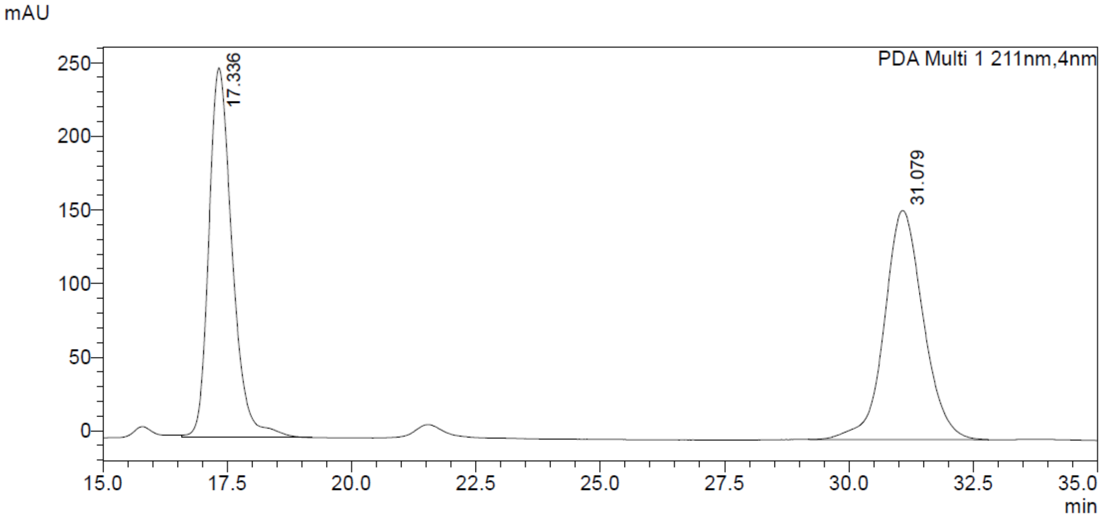

PDA Ch1 211nm

Peak#	Ret. Time	Area%
1	18.841	99.731
2	31.863	0.269
Total		100.000

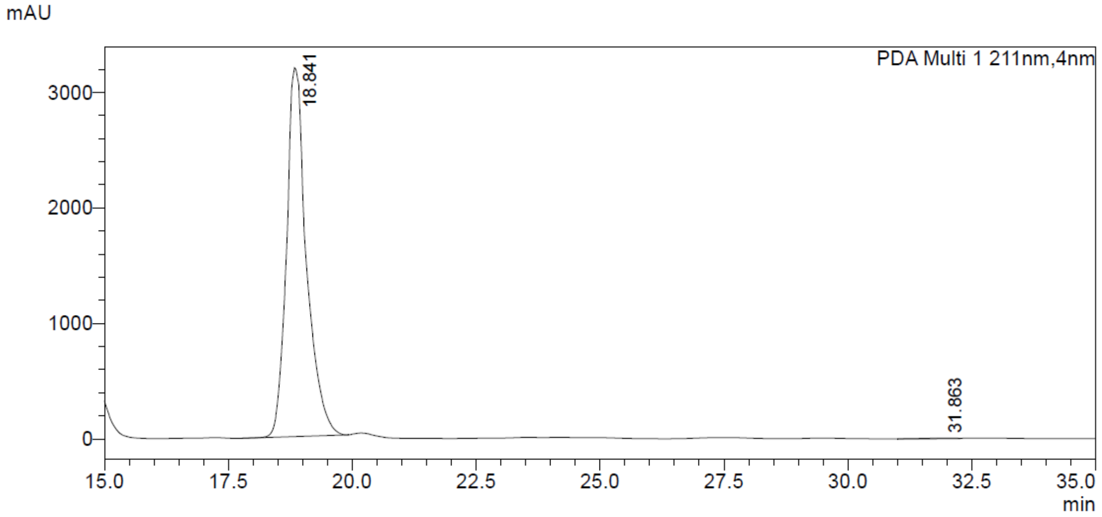

HPLC data for **34**: **Chiral HPLC analysis**, Chiralcel OD-H (80:20 hexane/IPA, flow rate 1 mLmin^{-1}, 211 nm, 30 °C) t_R (major): 21.2 min, t_R (minor): 29.3 min, > 99:1 er.

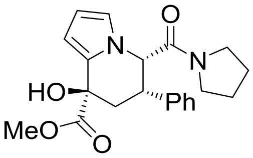

PDA Ch1 211nm

Peak#	Ret. Time	Area%
1	21.392	49.629
2	27.996	50.371
Total		100.000

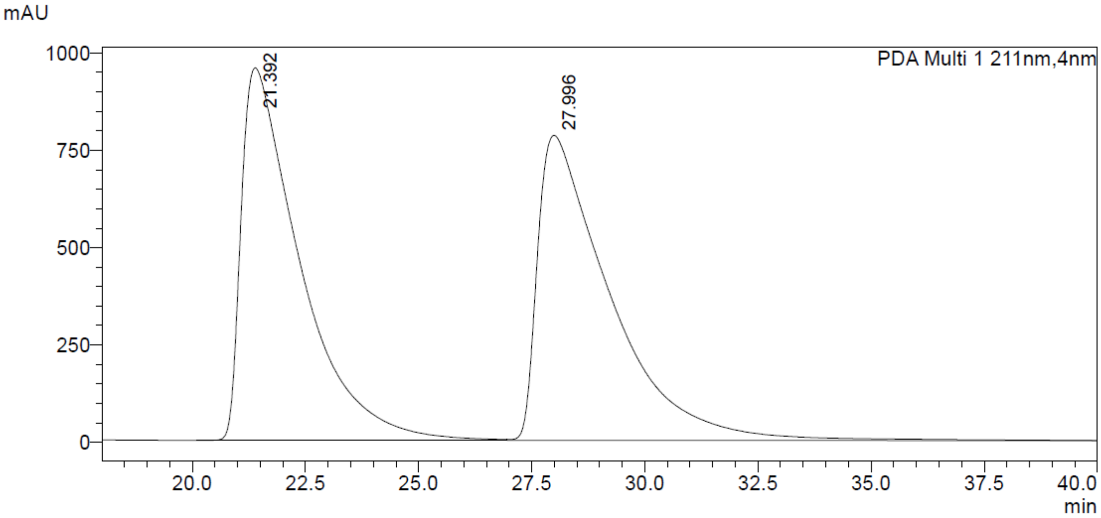

PDA Ch1 211nm

Peak#	Ret. Time	Area%
1	21.189	99.869
2	29.346	0.131
Total		100.000

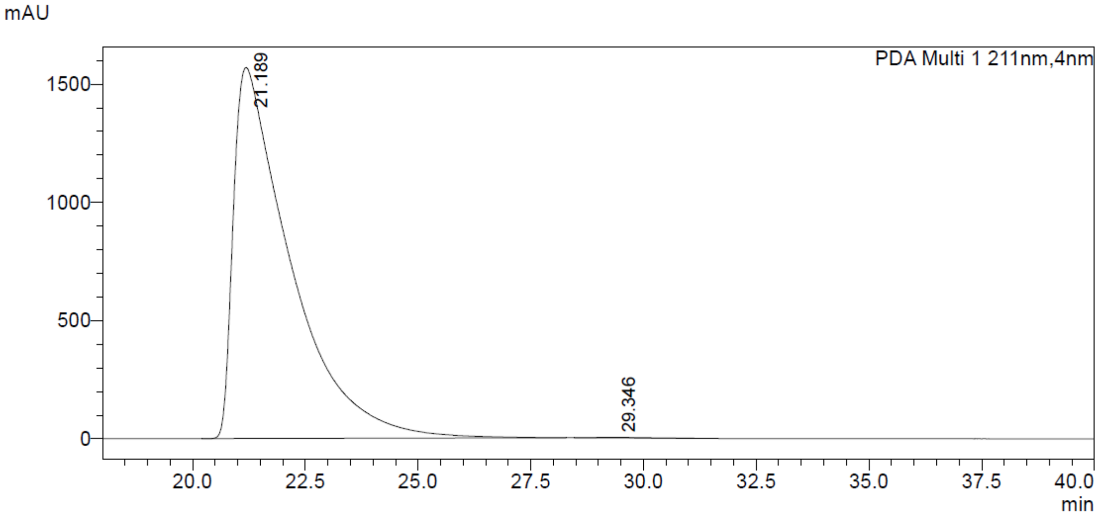

HPLC data for **35**: **Chiral HPLC analysis**, Chiralpak AD-H (90:10 hexane/IPA, flow rate 1 mLmin^{-1}, 211 nm, 30 °C) t_R (major): 31.4 min, t_R (minor): 63.7 min, > 99:1 er.

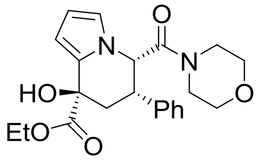

PDA Ch1 211nm

Peak#	Ret. Time	Area%
1	31.762	49.086
2	40.012	0.998
3	56.480	1.003
4	63.863	48.912
Total		100.000

Peaks at 40 and 56 min correspond to minor diastereoisomer

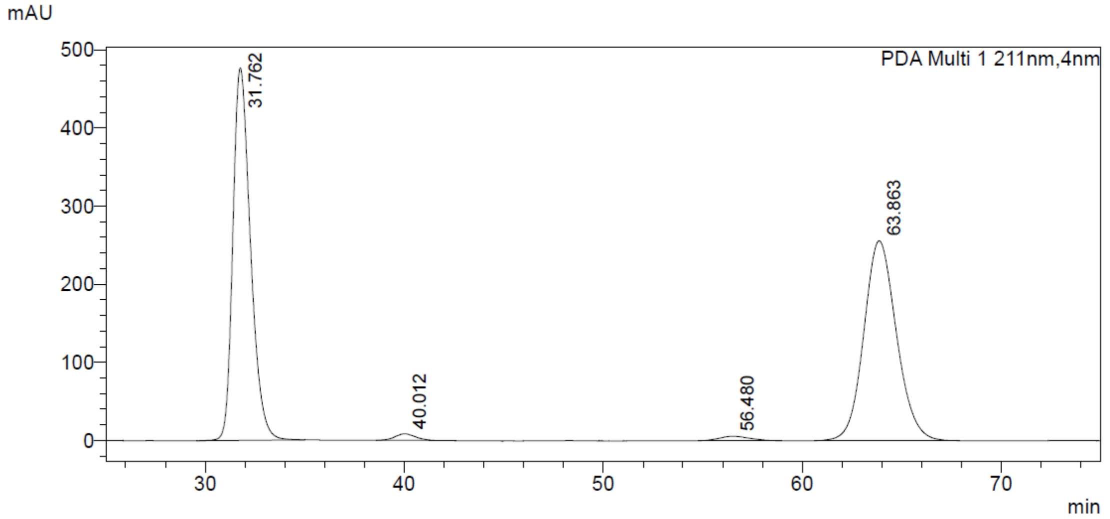

PDA Ch1 211nm

Peak#	Ret. Time	Area%
1	31.409	99.827
2	63.666	0.173
Total		100.000

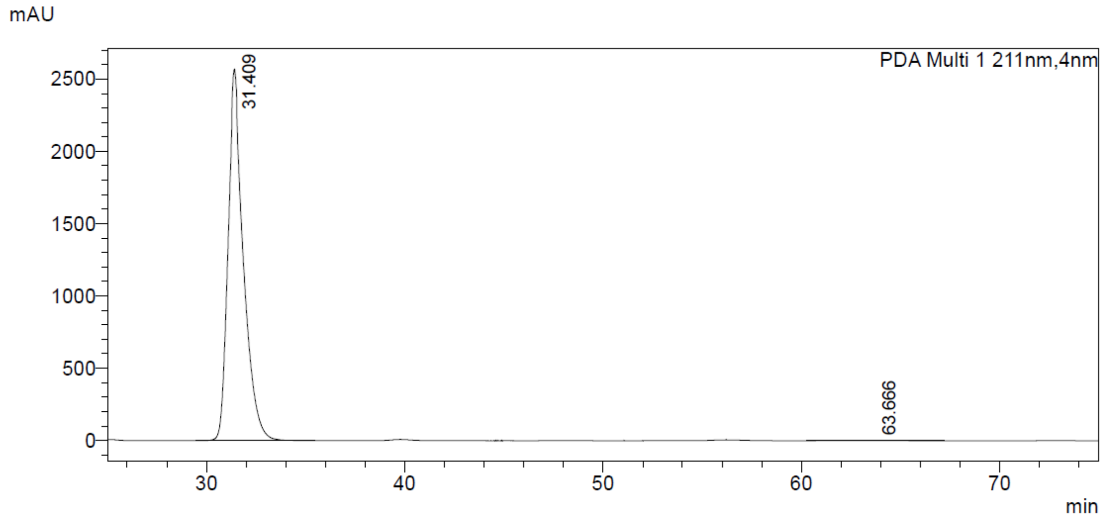

HPLC data for **36**: **Chiral HPLC analysis**, Chiralpak AD-H (90:10 hexane : IPA, flow rate 1 mLmin^{-1}, 211 nm, 30 °C) t_R (major): 27.3 min, t_R (minor): 56.9 min, > 99:1 er.

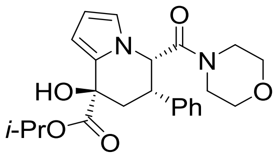

PDA Ch1 211nm

Peak#	Ret. Time	Area%
1	27.348	50.365
2	57.300	49.635
Total		100.000

Peaks at 32 and 46 min correspond to minor diastereoisomer

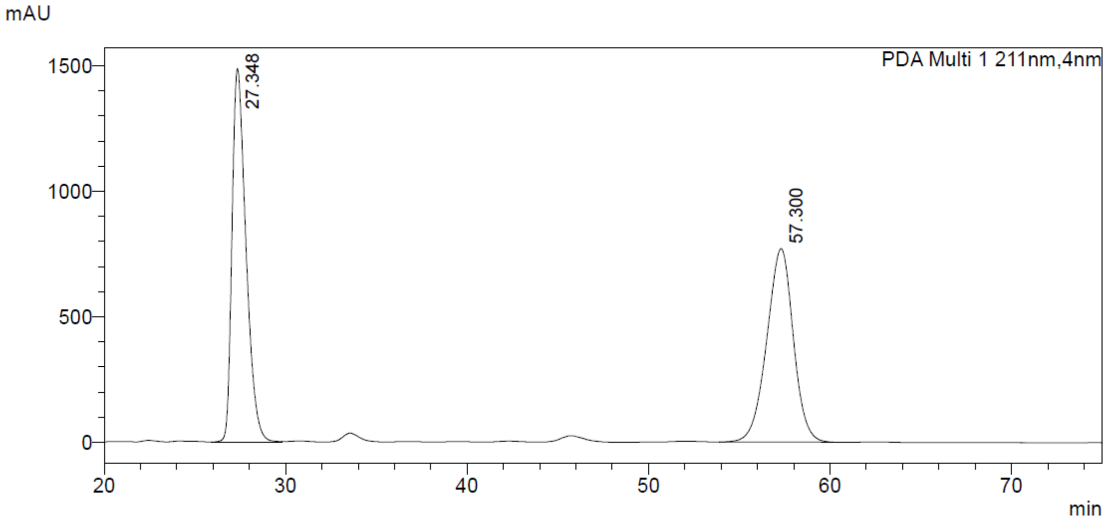

PDA Ch1 211nm

Peak#	Ret. Time	Area%
1	27.300	99.656
2	56.910	0.344
Total		100.000

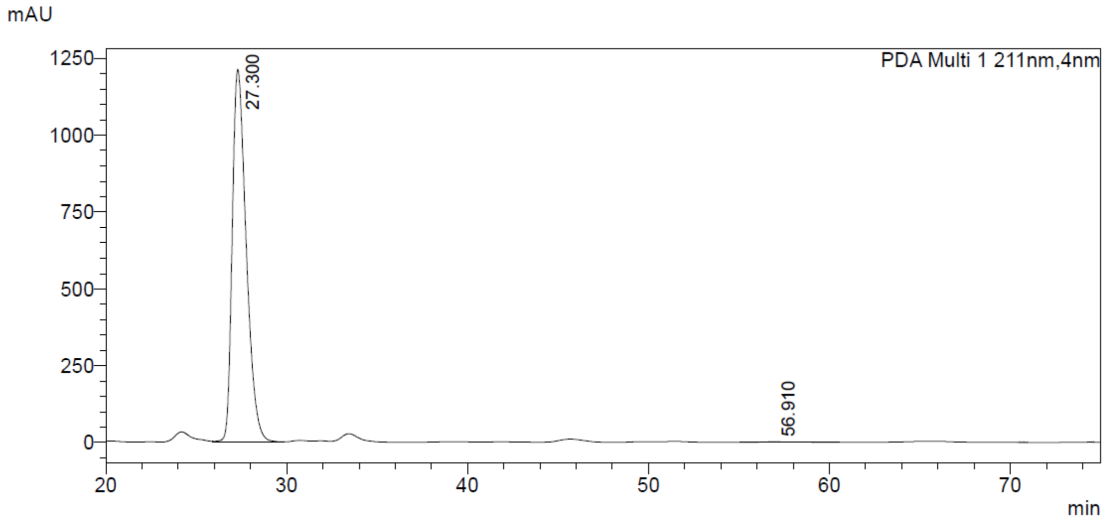

HPLC data for **37**: **Chiral HPLC analysis**, Chiralpak IB (80:20 hexane/IPA, flow rate 1 mLmin^{-1}, 211 nm, 30 °C) t_R (major): 50.6 min, t_R (minor): 65.4 min, > 99:1 er.

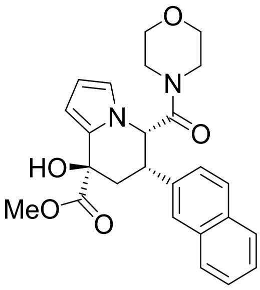

PDA Ch1 211nm

Peak#	Ret. Time	Area%
1	48.322	49.725
2	58.492	50.275
Total		100.000

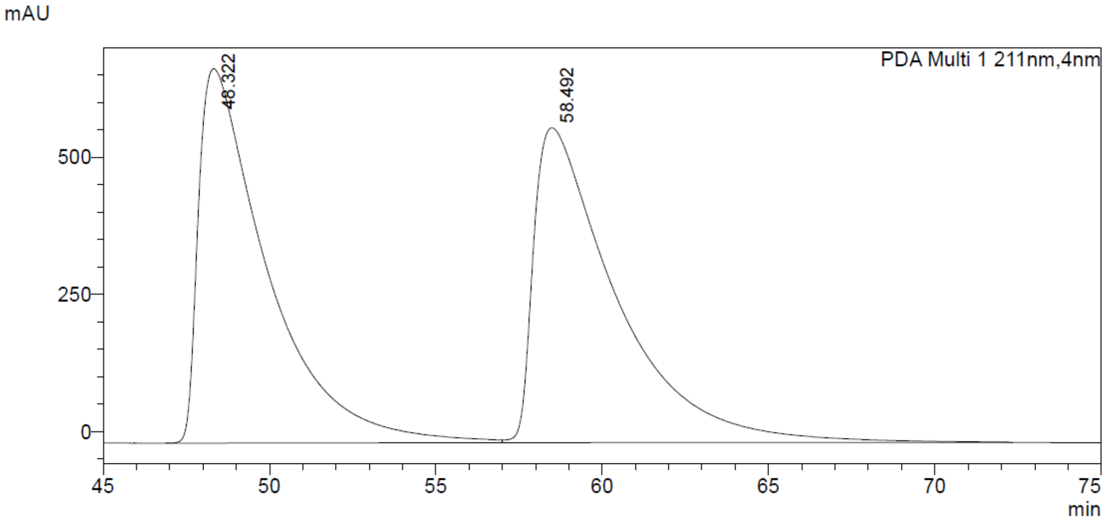

PDA Ch1 211nm

Peak#	Ret. Time	Area%
1	50.561	99.896
2	65.384	0.104
Total		100.000

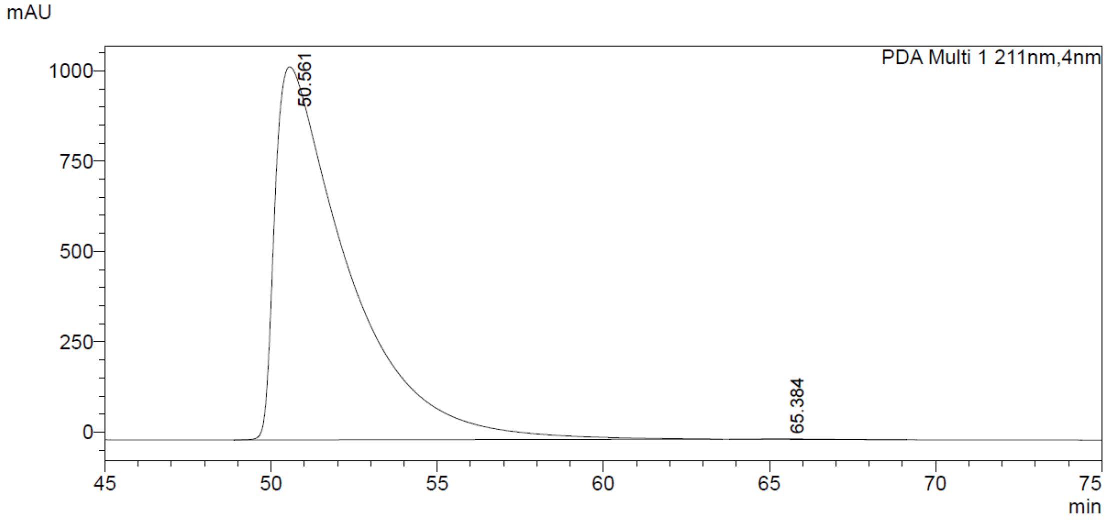

HPLC data for **38**: **Chiral HPLC analysis**, Chiralpak AD-H (90:10 hexane/IPA, flow rate 1 mLmin^{-1}, 211 nm, 30 °C) t_R (major): 56.4 min, t_R (minor): 80.8 min, > 99:1 er.

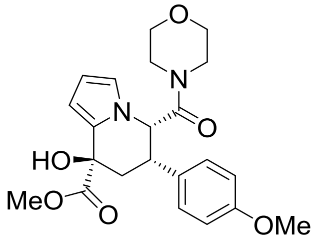

PDA Ch1 211nm

Peak#	Ret. Time	Area%
1	56.615	47.867
2	65.630	2.382
3	80.354	47.350
4	89.127	2.401
Total		100.000

Peaks at 66 and 89 min correspond to minor diastereoisomer

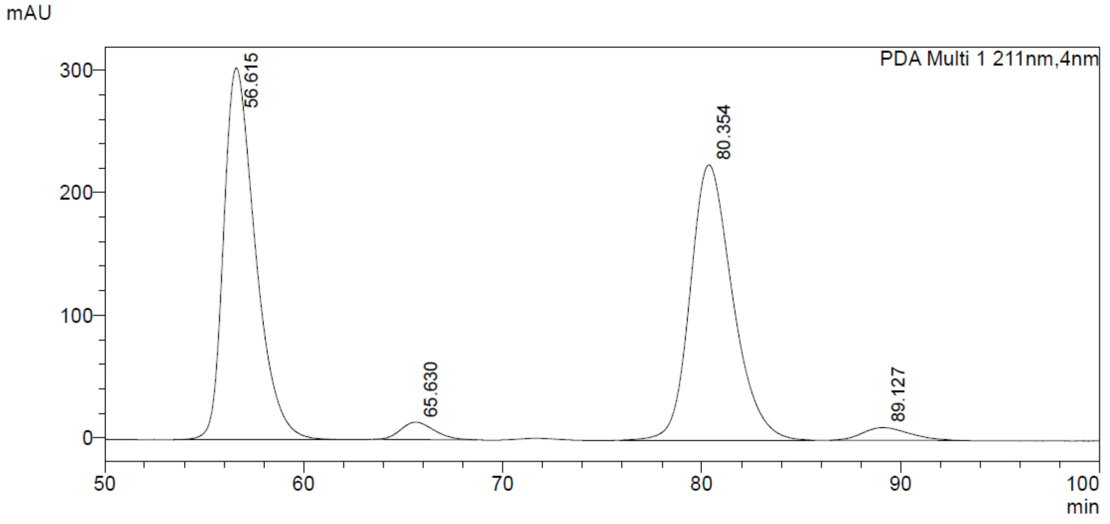

PDA Ch1 211nm

Peak#	Ret. Time	Area%
1	56.351	99.871
2	80.821	0.129
Total		100.000

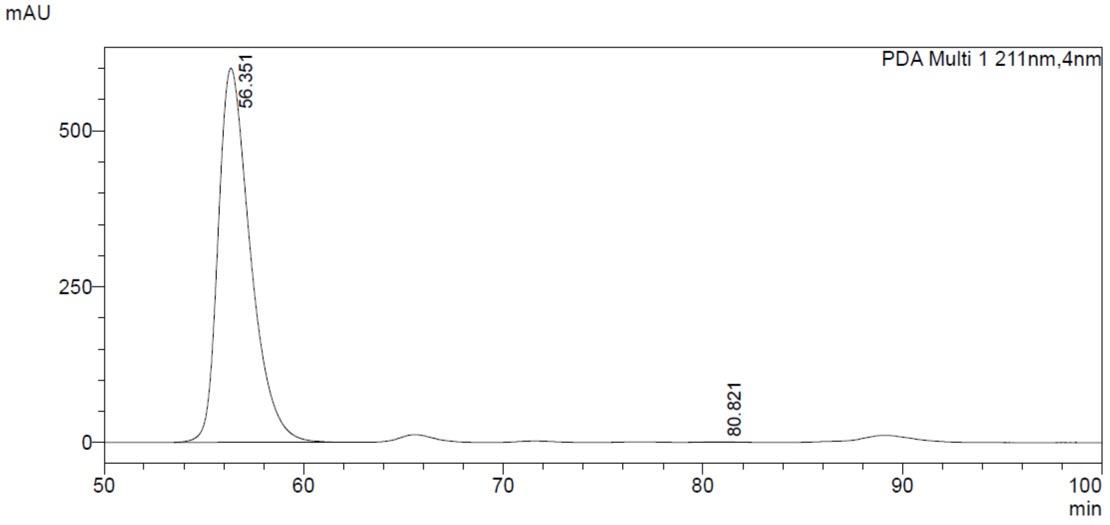

HPLC data for **39**: **Chiral HPLC analysis**, Chiralpak AD-H (90:10 hexane/IPA, flow rate 1 mLmin^{-1}, 211 nm, 30 °C) t_R (major): 38.5 min, t_R (minor): 55.0 min, > 99:1 er.

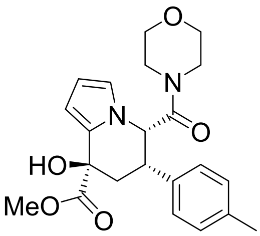

PDA Ch1 211nm

Peak#	Ret. Time	Area%
1	38.785	49.977
2	55.058	50.023
Total		100.000

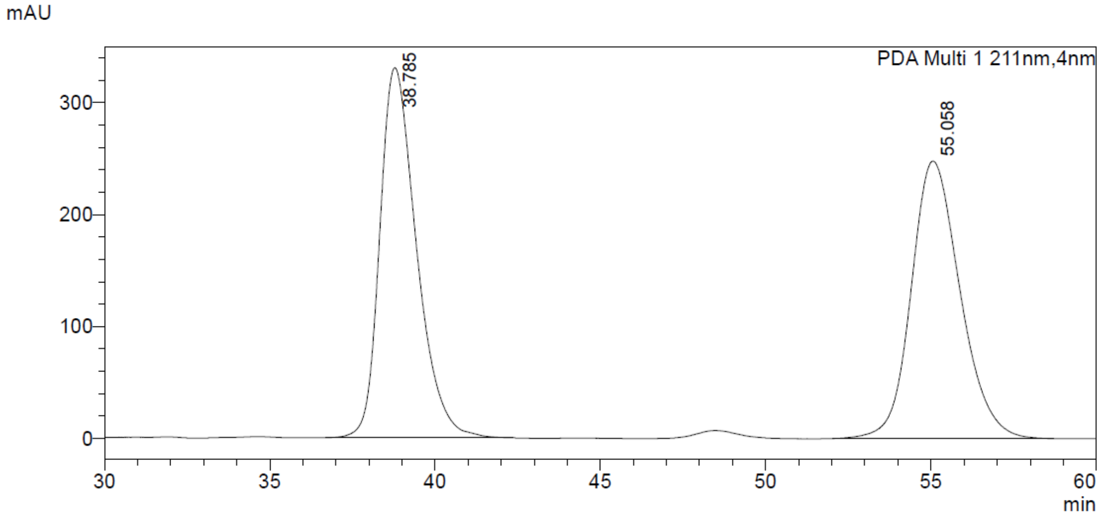

PDA Ch1 211nm

Peak#	Ret. Time	Area%
1	38.538	99.746
2	55.031	0.254
Total		100.000

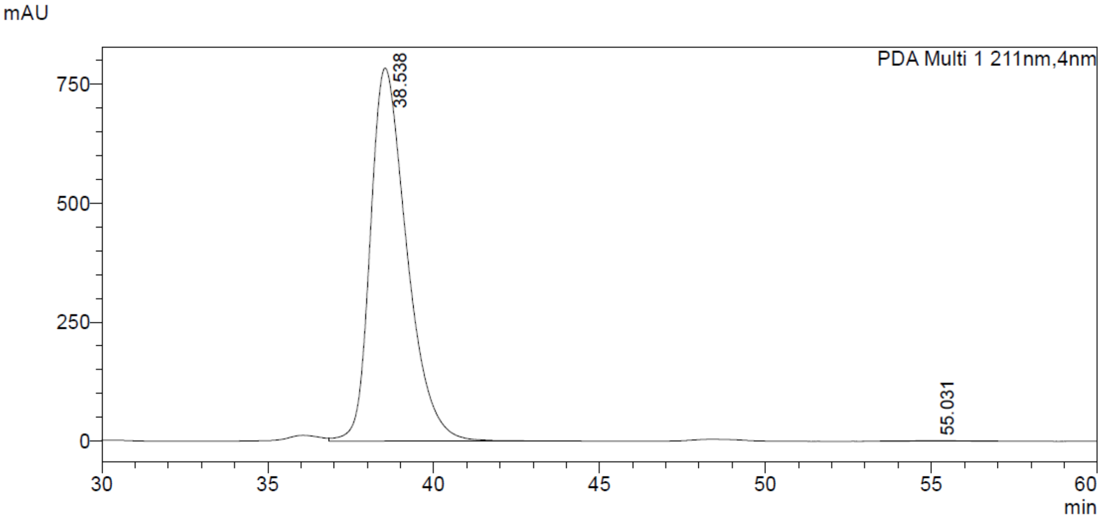

HPLC data for **40**: **Chiral HPLC analysis**, Chiralpak AD-H (90:10 hexane/IPA, flow rate 1 mLmin^{-1}, 211 nm, 30 °C) t_R (major): 46.8 min, t_R (minor): 68.2 min, > 99:1 er.

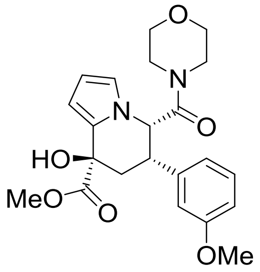

PDA Ch1 211nm

Peak#	Ret. Time	Area%
1	47.523	50.015
2	67.823	49.985
Total		100.000

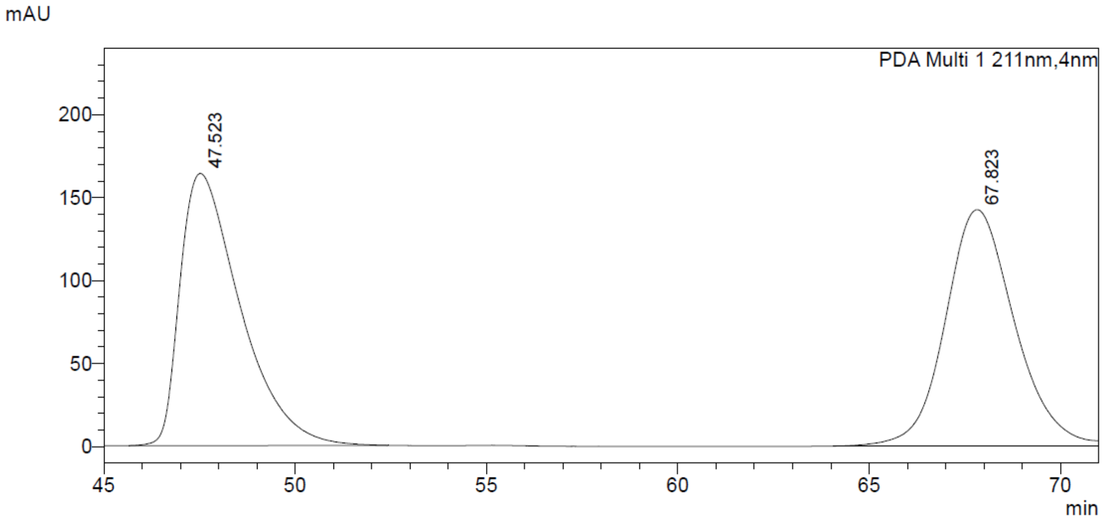

PDA Ch1 211nm

Peak#	Ret. Time	Area%
1	46.802	99.733
2	68.179	0.267
Total		100.000

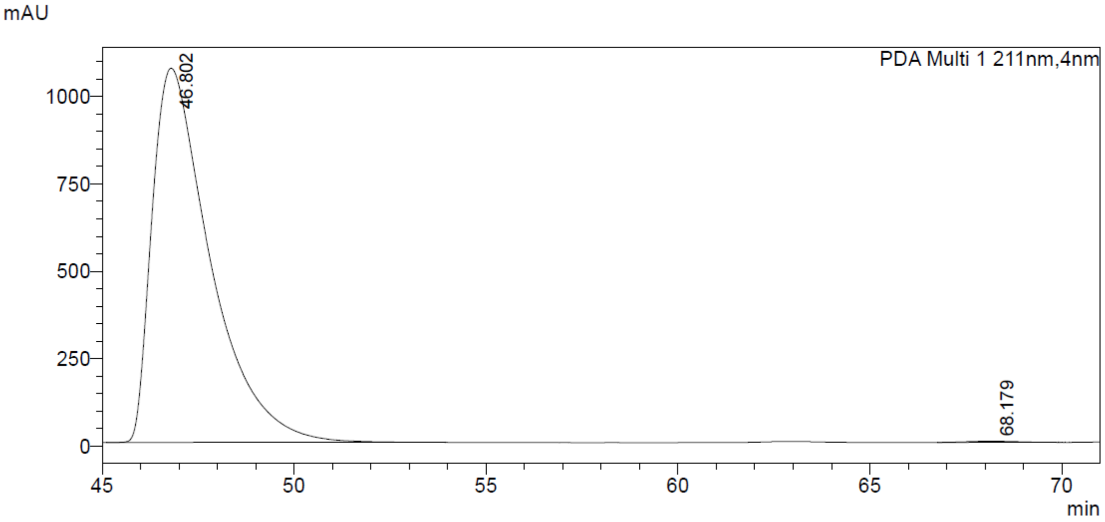

HPLC data for **41**: **Chiral HPLC analysis**, Chiralpak AD-H (90:10 hexane/IPA, flow rate 1 mLmin^{-1}, 211 nm, 30 °C) t_R (major): 88.3 min, t_R (minor): 134.8 min, > 99:1 er.

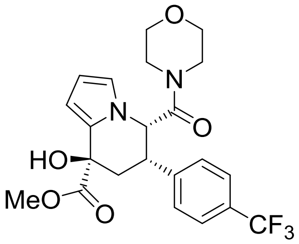

PDA Ch1 211nm

Peak#	Ret. Time	Area%
1	88.408	50.420
2	134.564	49.580
Total		100.000

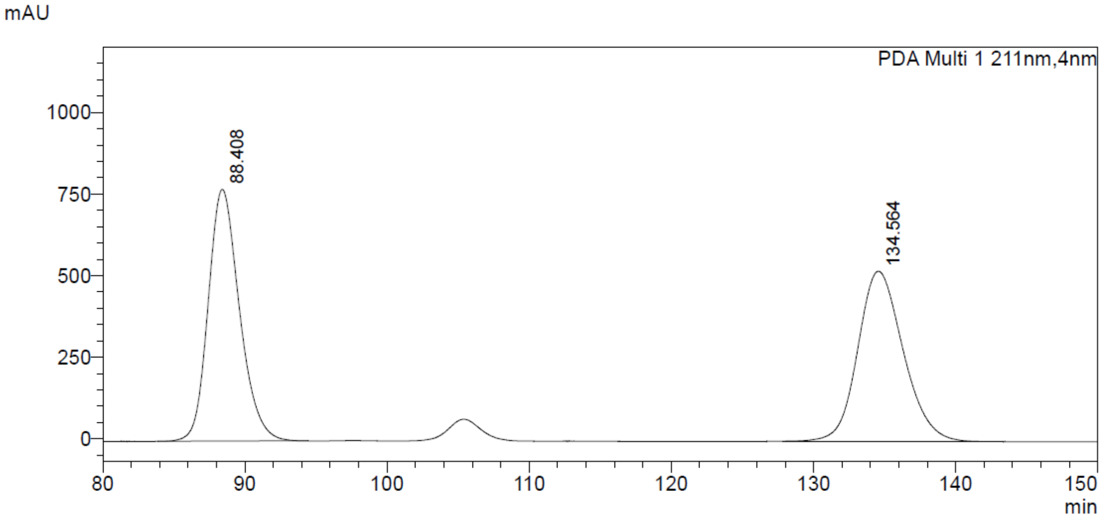

PDA Ch1 211nm

Peak#	Ret. Time	Area%
1	88.271	99.652
2	134.789	0.348
Total		100.000

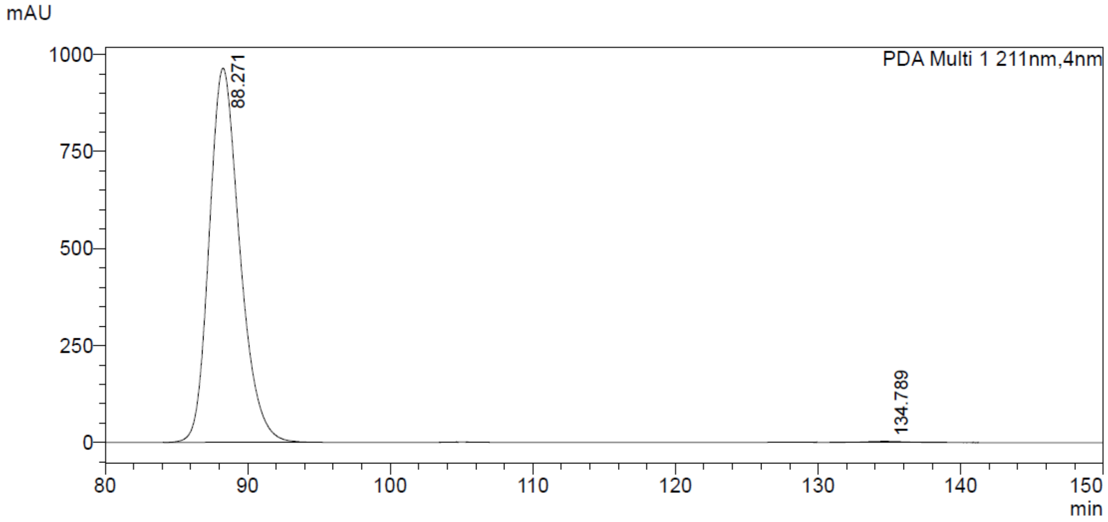

HPLC data for **42**: **Chiral HPLC analysis**, Chiralpak AD-H (80:20 hexane/IPA, flow rate 1 mLmin^{-1}, 220 nm, 30 °C) t_R (major): 41.5 min, t_R (minor): 63.8 min, > 99:1 er.

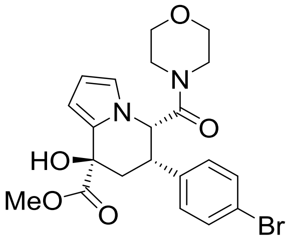

PDA Ch2 220nm

Peak#	Ret. Time	Area%
1	40.804	50.332
2	62.448	49.668
Total		100.000

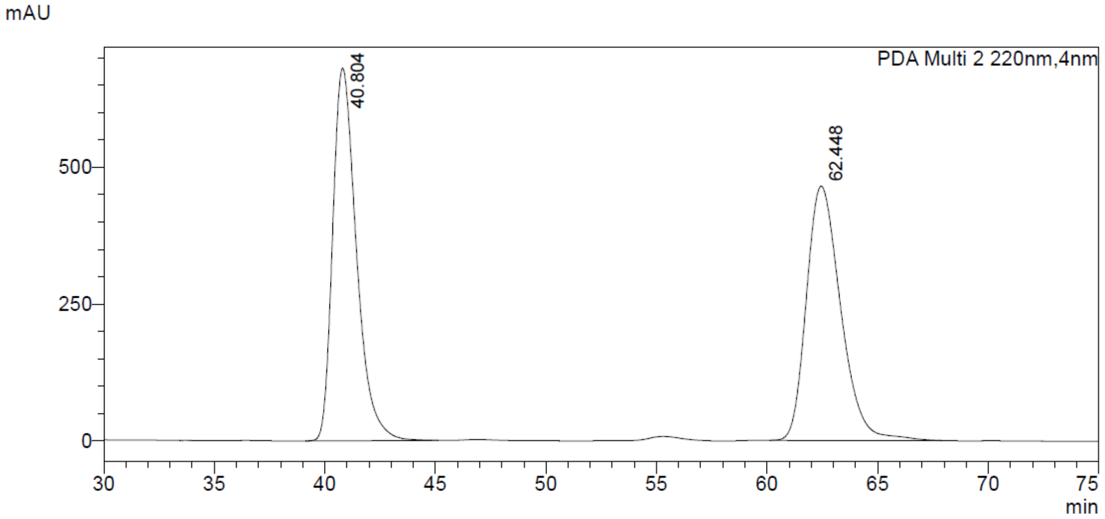

PDA Ch2 220nm

Peak#	Ret. Time	Area%
1	41.473	99.669
2	63.839	0.331
Total		100.000

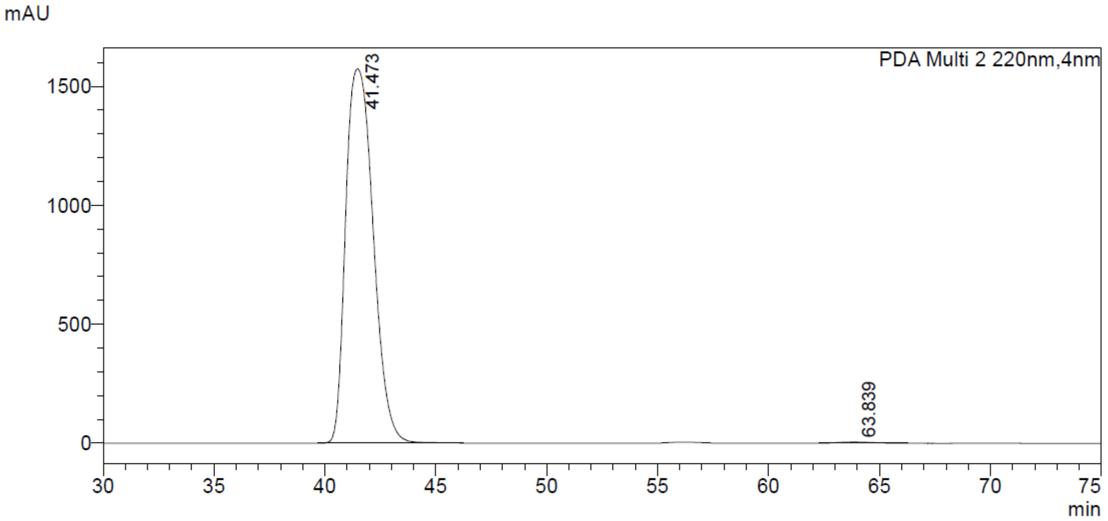

HPLC data for **43**: **Chiral HPLC analysis**, Chiralpak AD-H (90:10 hexane/IPA, flow rate 1 mLmin^{-1}, 211 nm, 30 °C) t_R (major): 63.3 min, t_R (minor): 87.9 min, > 99:1 er.

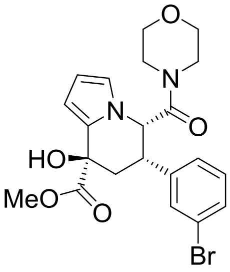

PDA Ch1 211nm

Peak#	Ret. Time	Area%
1	63.297	49.994
2	88.265	50.006
Total		100.000

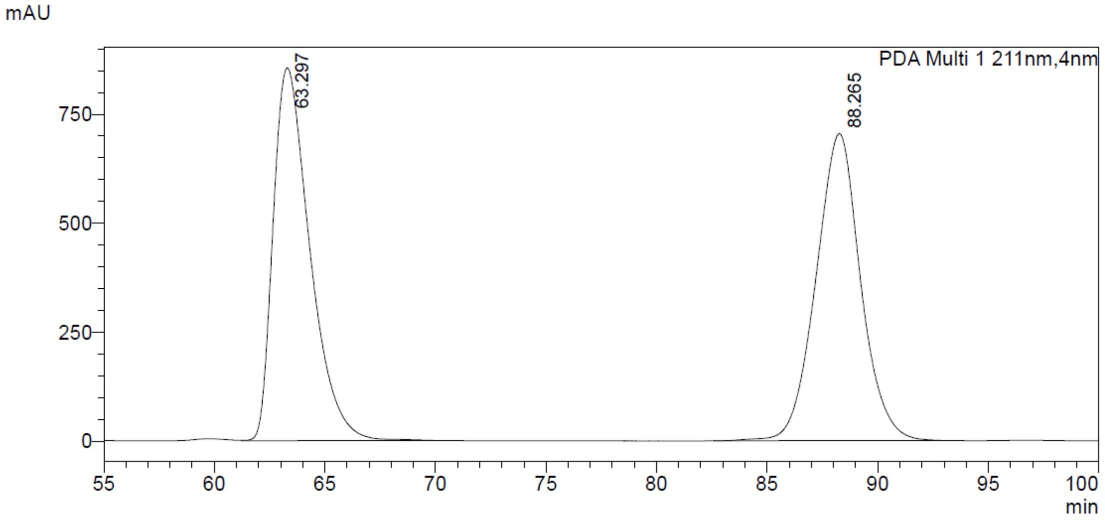

PDA Ch1 211nm

Peak#	Ret. Time	Area%
1	63.311	99.638
2	87.916	0.362
Total		100.000

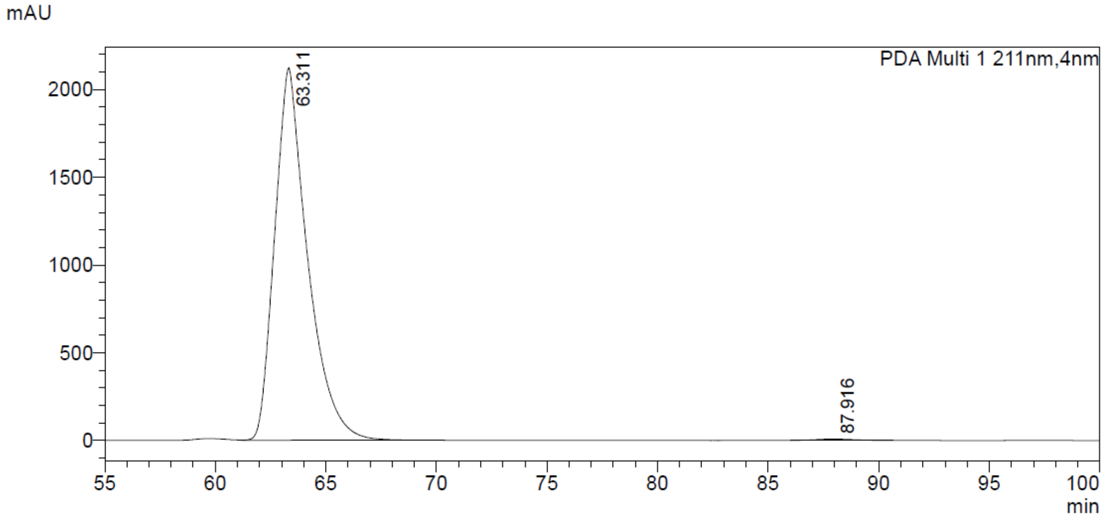

HPLC data for **44**: **Chiral HPLC analysis**, Chiralcel OD-H (90:10 hexane/IPA, flow rate 1 mLmin^{-1}, 220 nm, 30 °C) t_R (major): 37.6 min, t_R (minor): 58.4 min, > 99:1 er.

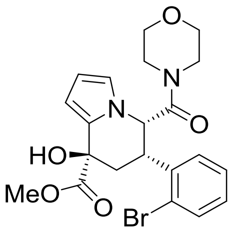

PDA Ch2 220nm

Peak#	Ret. Time	Area%
1	39.358	50.027
2	56.085	49.973
Total		100.000

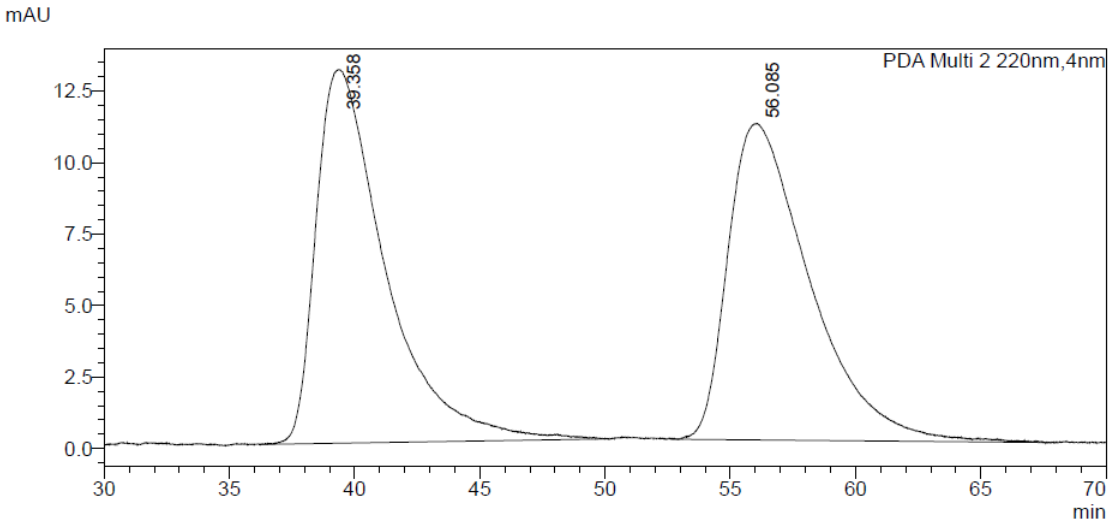

PDA Ch2 220nm

Peak#	Ret. Time	Area%
1	37.626	99.102
2	58.454	0.898
Total		100.000

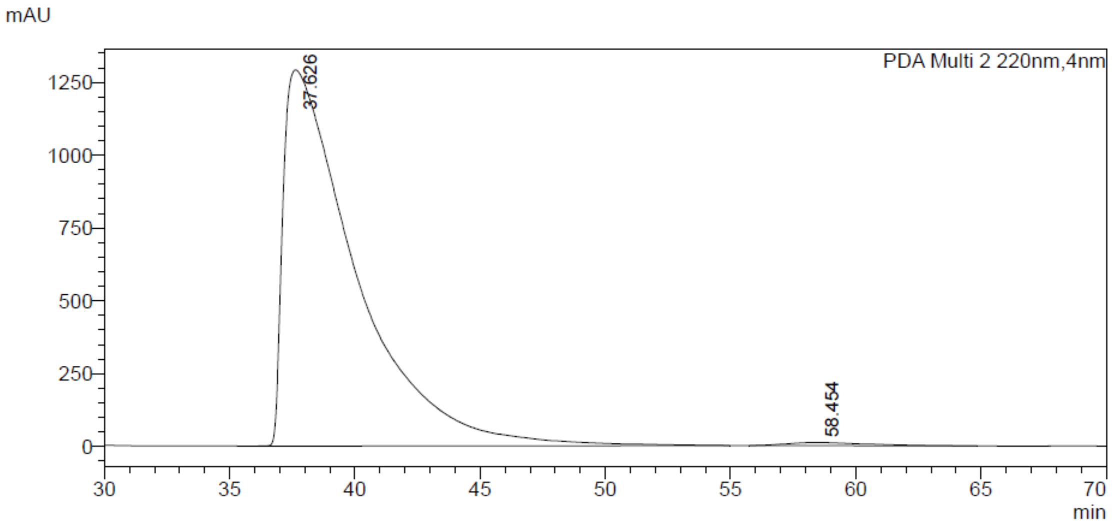

HPLC data for **45**: **Chiral HPLC analysis**, Chiralpak AD-H (90:10 hexane/IPA, flow rate 1 mLmin^{-1}, 220 nm, 30 °C) t_R (major): 89.3 min, t_R (minor): 53.1 min, > 99:1 er.

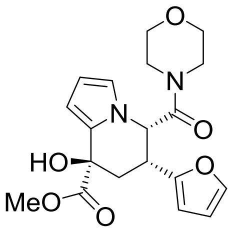

PDA Ch2 220nm

Peak#	Ret. Time	Area%
1	53.177	57.674
2	89.738	42.326
Total		100.000

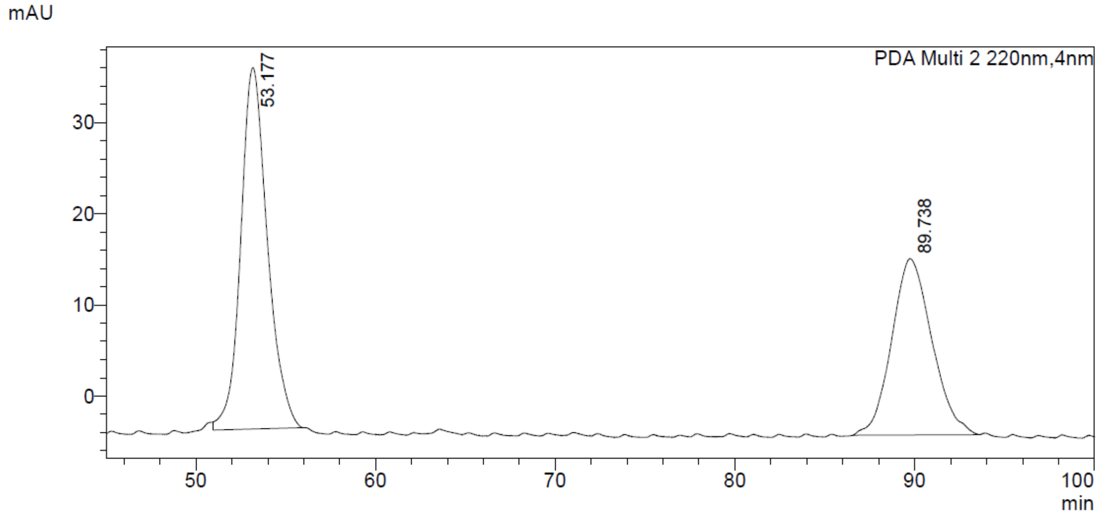

PDA Ch2 220nm

Peak#	Ret. Time	Area%
1	53.115	0.573
2	89.348	99.427
Total		100.000

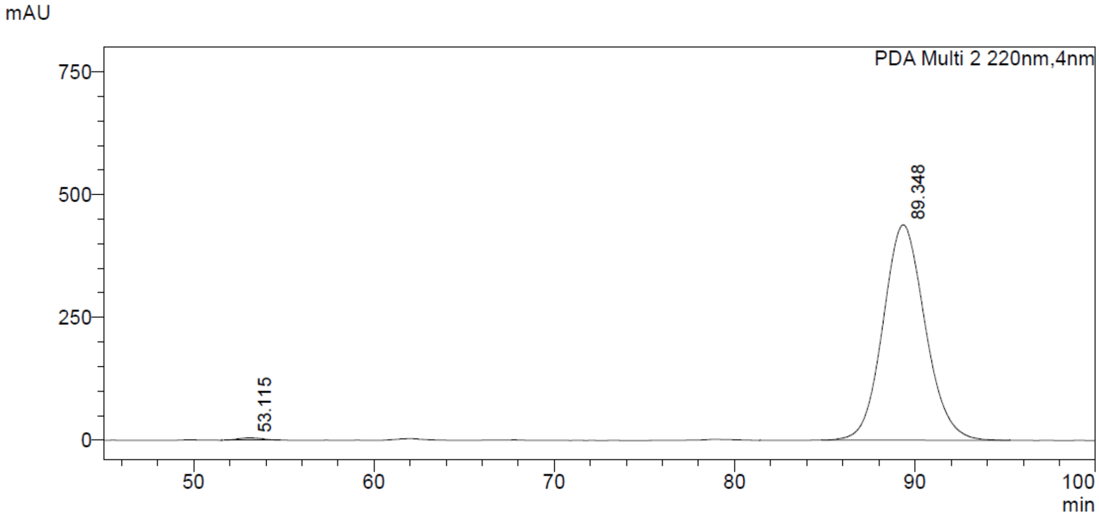

References

1 D. Belmessieri, L. C. Morrill, C. Simal, A. M. Z. Slawin and A. D. Smith, *J. Am. Chem. Soc.*, 2011, **133**, 2714–2720.

2 W. P. Unsworth, G. Coulthard, C. Kitsiou and R. J. K. Taylor, *J. Org. Chem.*, 2014, **79**, 1368–1376.

3 D. Wang, H. Nanding, N. Han, F. Chen and G. Zhao, *J. Agric. Food Chem.*, 2008, **56**, 1495–1500.

4 A. Sanz-Marco, G. Blay, M. C. Munoz and J. R. Pedro, *Chem. Commun.*, 2015, **51**, 8958–8961.

5 C. M. Young, J. E. Taylor and A. D. Smith, *Org. Biomol. Chem.*, 2019, **17**, 4747–4752.

6 Y. Wang, J. Han, J. Chen and W. Cao, *Tetrahedron*, 2015, **71**, 8256–8262.

7 T. A. Hamlin, C. B. Kelly and N. E. Leadbeater, *Eur. J. Org. Chem.*, 2013, **2013**, 3658–3661.

8 B. Ouyang, T. Yu, R. Luo and G. Lu, *Org. Biomol. Chem.*, 2014, **12**, 4172–4176.

9 Y.-Z. Hua, M.-M. Liu, P.-J. Huang, X. Song, M.-C. Wang and J.-B. Chang, *Chem. Eur. J.*, 2015, **21**, 11994–11998.

10 X. Tang, L. Tong, H. Liang, J. Liang, Y. Zou, X. Zhang, M. Yan and A. S. C. Chan, *Org. Biomol. Chem.*, 2018, **16**, 3560–3563.

11 *CrystalClear-SM Exper*t v2.1. Rigaku Americas, The Woodlands, Texas, USA, and Rigaku Corporation, Tokyo, Japan, 2015.

12 *CrysAlisPro* v1.171.38.46. Rigaku Oxford Diffraction, Rigaku Corporation, Oxford, U.K. 2015.

13 G. M. Sheldrick, *Acta Crystallogr., Sect. A*. 2015, **71**, 3–8.

14 M. C. Burla, R. Caliandro, M. Camalli, B. Carrozzini, G. L. Cascarano, C. Giacovazzo, M. Mallamo, A. Mazzone, G. Polidori and R. Spagna, *J. Appl. Cryst*. 2012, **45**, 357–361.

15 L. Palatinus and G. Chapuis, *J. Appl. Cryst*. 2007, **40**, 786–790.

16 G. M. Sheldrick, *Acta Crystallogr., Sect. C*. 2015, **71**, 3–8.

17 *CrystalStructure* v4.3.0. Rigaku Americas, The Woodlands, Texas, USA, and Rigaku Corporation, Tokyo, Japan, 2018.
